# Supplementary material for: Interactions between the protein barnase and co-solutes studied by NMR
Source: Commun Chem. 2024 Feb 28;7:44. doi: 10.1038/s42004-024-01127-0 (PMC10902301; doi:10.1038/s42004-024-01127-0)
Supplement: Supplementary file 2 — Supplementary material [file 42004_2024_1127_MOESM2_ESM.pdf]

Interactions between the protein barnase and co-solutes studied by NMR

Clare R. Trevitt, Yashwanth Kumar D. R., Nicholas J. Fowler and Mike P. Williamson

Supplementary Material

Supplementary Material contains a table of fitted data for all co-solutes as a separate text file (Supplementary Data 1), a figure showing chemical shift changes for N, H and C' on titration with a 1:1 mixture of thiocyanate and sulfate, a figure showing chemical shift changes for methyl H and C on titration with TMAO, a figure showing the hydrogen bonding network around L42 and R83 in barnase, and a figure showing the numbering of residues in barnase.

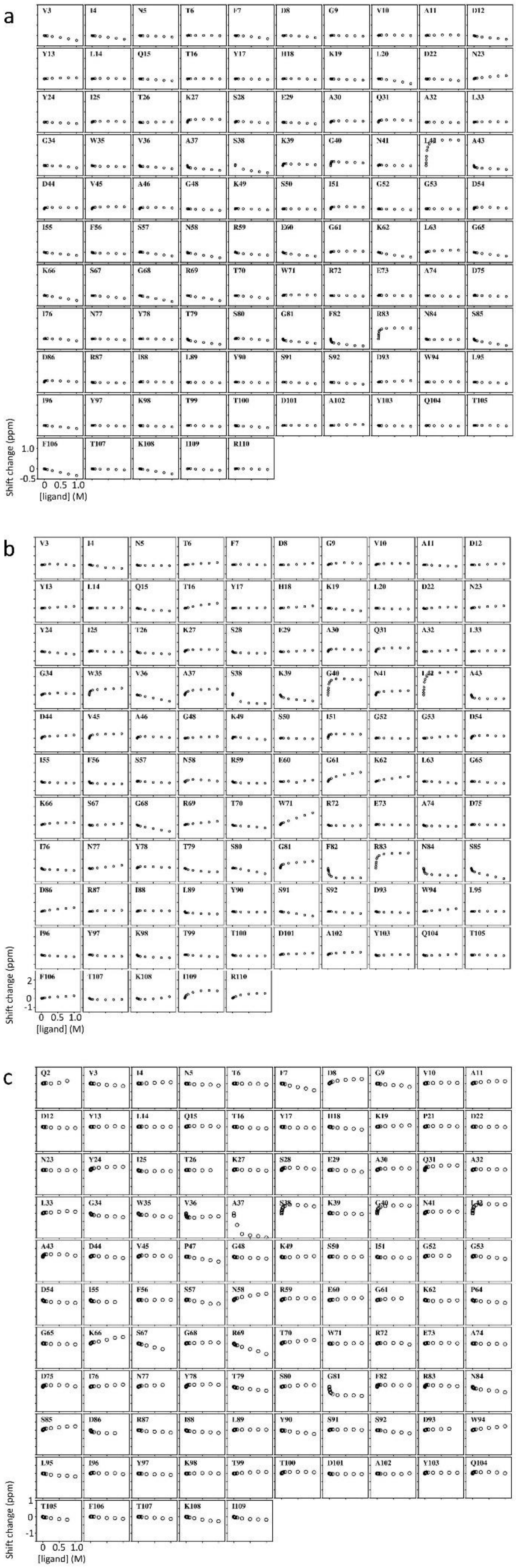
a
Shift change (ppm)
[ligand] (M)
b
Shift change (ppm)
[ligand] (M)
c
Shift change (ppm)
[ligand] (M)

Supplementary Figure 1. Chemical shift changes for (a) ^{1}H, (b) ^{15}N and (c) ^{13}C nuclei from backbone amides in barnase on addition of a 1:1 mixture of thiocyanate and sulfate. Chemical shift scale are given at the bottom left of each panel.

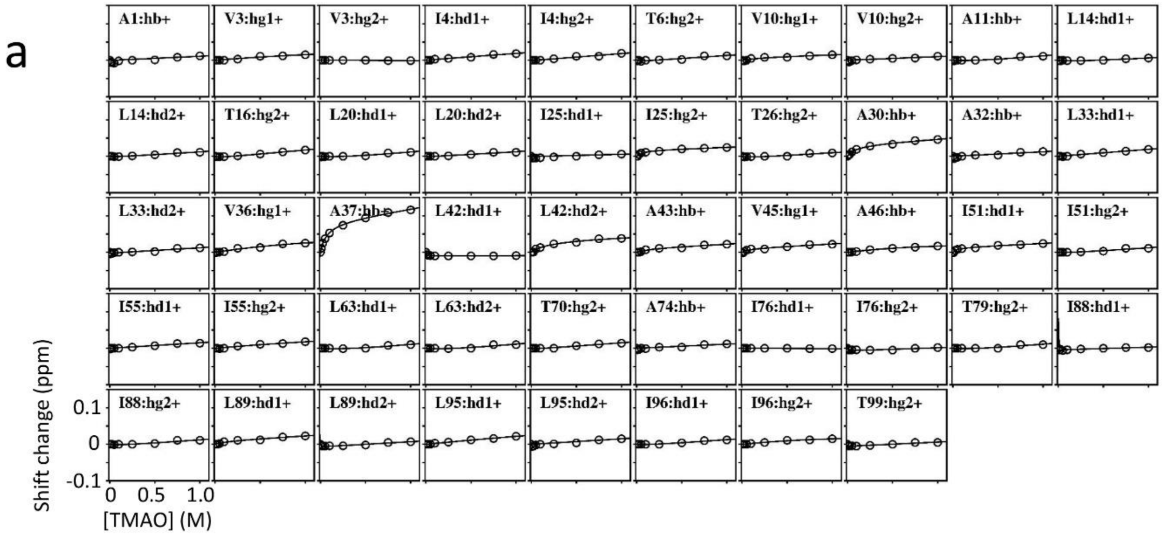

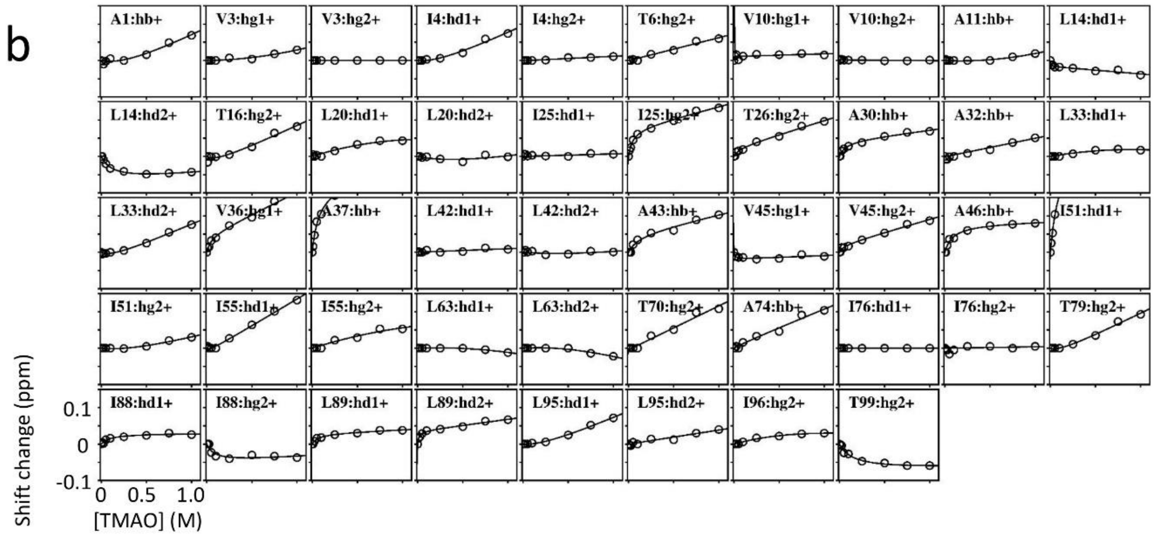

Supplementary Figure 2. Chemical shift changes for (a) ^{1}H and (b) ^{13}C nuclei from methyl groups in barnase on addition of TMAO. ^{1}H shifts show no indication of binding (ie no curvature), except for A30, A37 and L42. L42 is one of the two residues with unusual behaviour (Fig 2), and A37 carbonyl is part of the same hydrogen bonding network. The ^{13}C shifts show much more curvature, but predominantly for alanines.

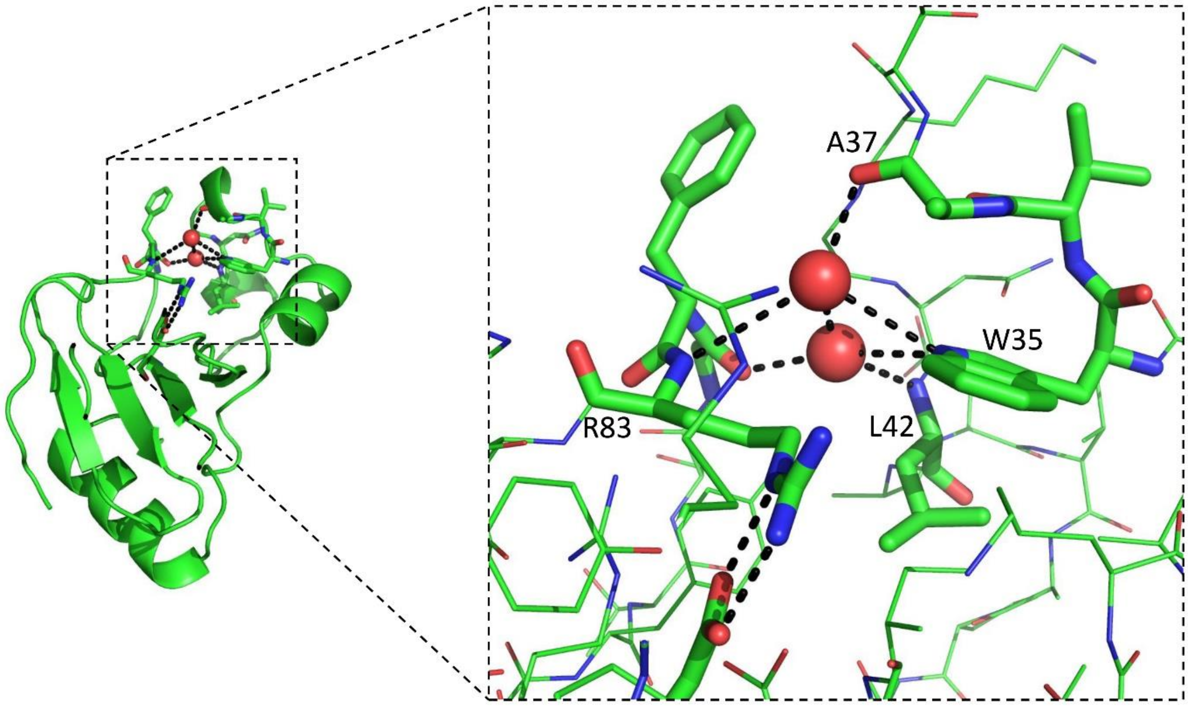

Supplementary Figure 3. Hydrogen bonding network in the barnase crystal structure (pdb ID 1a2p) involving two deeply buried water molecules, which form hydrogen bonds to the NH of R83, L42 and the W35 sidechain. The sidechain of R83 makes hydrogen bonds to the scissile phosphate of the substrate and is a key active site residue.

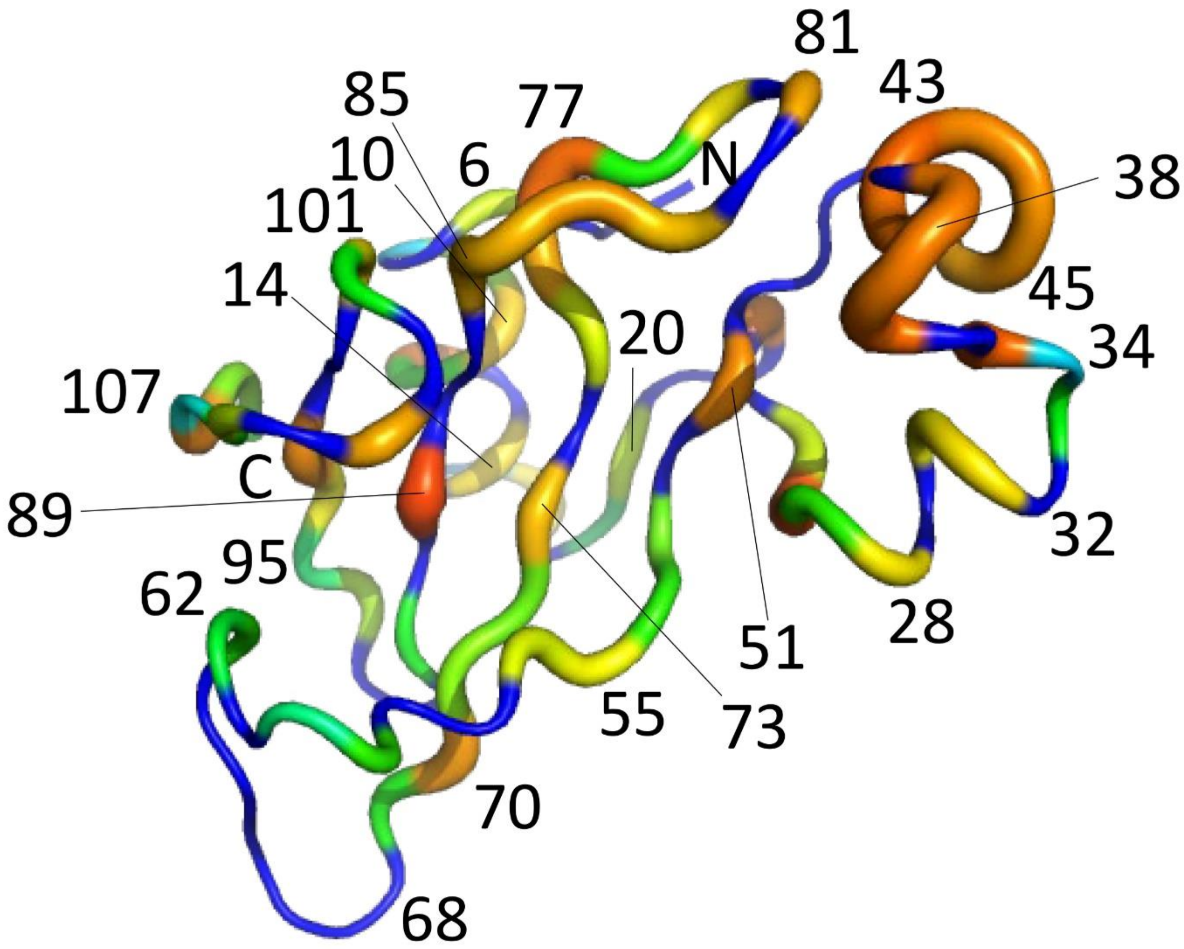

Fig. S4. The structure of barnase (using the structure colored as in Figure 2a) with residue numbers indicated.
