# Supplementary material for: Interactions between the protein barnase and co-solutes studied by NMR
Source: Commun Chem. 2024 Feb 28;7:44. doi: 10.1038/s42004-024-01127-0 (PMC10902301; doi:10.1038/s42004-024-01127-0)
Supplement: Supplementary file 3 — Description of Additional Supplementary Files [file 42004_2024_1127_MOESM3_ESM.pdf]

Description of Additional Supplementary Files

File name: Supplementary Data 1

Description: This file has a separate line for each nucleus (amide proton, amide nitrogen, and amide carbonyl) and each co-solute. Each line contains two sets of data. Columns 4-10 contain values fitted to the full equation (3), namely mL, $\Delta\delta$max, and Kd, together with their associated errors, and the χ 2 value for the fitting. Columns 14-18 contain values fitted to the simple linear equation $\Delta\delta$max, = mL[L] + c, again with the errors and χ 2 values. The choice as to which of these values to use was usually dictated by the criteria set out in the Methods section of the main text. Namely, the full fit was used, unless Kd was outside the range of 5 – 900 mM, or the fitted absolute value of $\Delta\delta$max was less than 0.03 ppm for 1H, or 0.06 ppm for 15N or 13C, in which case the simple linear equation was used.
