# Supplementary material for: Interactions between the protein barnase and co-solutes studied by NMR
Source: Commun Chem. 2024 Feb 28;7:44. doi: 10.1038/s42004-024-01127-0 (PMC10902301; doi:10.1038/s42004-024-01127-0)
Supplement: Supplementary file 4 — Supplementary Data 1 [file 42004_2024_1127_MOESM4_ESM.pdf]

res	ion	shift	m	m_er	ddmax	dd_er	kd	kd_er	chi2	res	ion	shift	lm	lm_er	lc	lc_er	lchi2
A102	bet	c	-69.69	72.6	-0.0318	0.2352	1000.00	5848.23	0.00013	A102	bet	c	-87.30	2.6	-0.00	0.00	0.00017
A102	bet	h	16.82	1.3	-0.0009	0.0008	1.23	18.41	0.00002	A102	bet	h	16.52	0.9	-0.00	0.00	0.00002
A102	bet	n	-81.09	97.0	-0.0237	0.3142	1000.00	10504.63	0.00023	A102	bet	n	-95.11	3.1	0.00	0.00	0.00024
A102	ect	c	17.15	158.1	-0.1354	0.5121	1000.00	2992.05	0.00061	A102	ect	c	-54.93	7.0	-0.00	0.00	0.00124
A102	ect	h	15.18	27.9	0.0141	0.0904	999.98	5070.54	0.00002	A102	ect	h	22.70	1.0	0.00	0.00	0.00003
A102	ect	n	-113.18	104.6	0.0238	0.3388	1000.00	11245.84	0.00027	A102	ect	n	-101.37	3.3	0.00	0.00	0.00027
A102	tmao	c	-72.69	176.0	0.0299	0.5699	1000.00	15067.70	0.00075	A102	tmao	c	-52.88	5.3	-0.00	0.00	0.00070
A102	tmao	h	17.05	14.6	0.0062	0.0260	352.92	1708.02	0.00006	A102	tmao	h	22.65	1.7	-0.00	0.00	0.00007
A102	tmao	n	-84.02	222.1	0.1599	0.7194	1000.00	3560.90	0.00120	A102	tmao	n	5.76	9.3	-0.00	0.00	0.00220
A11	bet	c	-0.01	20.5	0.0059	0.0665	1000.00	8847.03	0.00001	A11	bet	c	3.53	0.7	-0.00	0.00	0.00001
A11	bet	h	9.12	0.8	-0.0022	0.0005	1.00	4.79	0.00001	A11	bet	h	8.43	0.9	-0.00	0.00	0.00002
A11	bet	n	-5.38	52.0	0.0005	0.1683	1000.00	282980.50	0.00007	A11	bet	n	-3.67	1.4	-0.00	0.00	0.00005
A11	ect	c	20.23	92.8	0.0079	0.3006	1000.00	30284.62	0.00021	A11	ect	c	25.16	2.9	-0.00	0.00	0.00021
A11	ect	h	23.15	0.9	0.0009	0.0007	38.05	81.93	0.00000	A11	ect	h	23.86	0.4	0.00	0.00	0.00000
A11	ect	n	-58.39	71.6	0.0300	0.1827	683.79	3737.21	0.00034	A11	ect	n	-39.74	4.0	0.00	0.00	0.00040
A11	tmao	c	27.08	15.5	0.0418	0.0247	277.94	206.32	0.00011	A11	tmao	c	61.58	5.5	0.00	0.00	0.00076
A11	tmao	h	16.46	19.1	0.0231	0.0403	489.43	862.10	0.00005	A11	tmao	h	33.03	2.3	0.00	0.00	0.00013
A11	tmao	n	-19.78	4.2	-0.0078	0.0025	1.00	6.95	0.00022	A11	tmao	n	-22.16	3.8	-0.01	0.00	0.00037
A30	bet	c	22.77	36.1	0.0020	0.1168	1000.00	46305.95	0.00003	A30	bet	c	23.91	1.1	-0.00	0.00	0.00003
A30	bet	h	12.71	1.4	-0.0021	0.0008	1.20	8.76	0.00002	A30	bet	h	12.04	1.2	-0.00	0.00	0.00003
A30	bet	n	20.76	34.2	-0.0201	0.0904	725.99	2868.55	0.00007	A30	bet	n	9.49	1.6	-0.00	0.00	0.00007
A30	ect	c	60.48	4.6	-0.0029	0.0028	1.00	20.17	0.00027	A30	ect	c	59.67	3.4	-0.00	0.00	0.00028
A30	ect	h	15.98	10.2	0.0205	0.0303	879.35	1070.25	0.00000	A30	ect	h	27.32	0.9	0.00	0.00	0.00002
A30	ect	n	-54.83	94.9	0.1155	0.3074	1000.00	2106.49	0.00022	A30	ect	n	7.00	5.2	0.00	0.00	0.00069
A30	tmao	c	2.62	72.0	0.0949	0.2331	999.61	1942.86	0.00013	A30	tmao	c	53.17	4.2	0.00	0.00	0.00044
A30	tmao	h	19.77	4.6	0.0242	0.0048	94.55	35.40	0.00004	A30	tmao	h	40.42	5.0	0.01	0.00	0.00062
A30	tmao	n	-6.59	20.9	0.1868	0.0228	104.35	23.02	0.00078	A30	tmao	n	150.21	34.1	0.04	0.02	0.02946
A32	bet	c	-51.43	3.2	0.0024	0.0019	1.00	16.94	0.00013	A32	bet	c	-50.75	2.3	0.00	0.00	0.00014
A32	bet	h	1.64	1.1	-0.0010	0.0007	5.01	21.80	0.00001	A32	bet	h	1.19	0.8	-0.00	0.00	0.00001
A32	bet	n	-23.65	3.1	-0.0110	0.0037	137.98	76.85	0.00001	A32	bet	n	-33.14	2.0	-0.00	0.00	0.00010
A32	ect	c	-36.42	92.6	0.0332	0.3000	1000.00	7151.45	0.00021	A32	ect	c	-18.56	3.1	0.00	0.00	0.00025
A32	ect	h	-9.35	16.5	0.0504	0.0534	1000.00	838.83	0.00001	A32	ect	h	16.80	1.7	0.00	0.00	0.00008
A32	ect	n	-52.61	62.7	0.0632	0.1345	503.61	1072.14	0.00054	A32	ect	n	-7.63	6.7	0.00	0.00	0.00112
A32	tmao	c	-11.58	15.1	0.1144	0.0160	93.97	24.66	0.00046	A32	tmao	c	84.61	22.3	0.03	0.01	0.01259
A32	tmao	h	17.09	4.0	0.0361	0.0045	113.83	24.95	0.00003	A32	tmao	h	47.69	6.5	0.01	0.00	0.00107
A32	tmao	n	8.68	7.4	0.1370	0.0075	83.09	8.92	0.00012	A32	tmao	n	120.97	26.1	0.04	0.01	0.01731
A37	bet	c	91.19	168.6	0.1192	0.5461	1000.00	3625.34	0.00069	A37	bet	c	154.98	6.9	0.00	0.00	0.00120
A37	bet	h	22.46	1.1	-0.0018	0.0007	1.35	8.33	0.00002	A37	bet	h	21.86	1.0	-0.00	0.00	0.00002
A37	bet	n	61.99	4.3	-0.0020	0.0034	26.75	131.46	0.00010	A37	bet	n	60.43	2.1	-0.00	0.00	0.00011
A37	ect	c	64.75	4.5	-0.0095	0.0037	32.71	34.74	0.00010	A37	ect	c	57.67	3.2	-0.00	0.00	0.00026
A37	ect	h	40.03	3.1	0.0009	0.0031	76.50	541.38	0.00002	A37	ect	h	40.96	1.0	0.00	0.00	0.00003
A37	ect	n	144.86	185.7	0.0610	0.6014	1000.00	7806.58	0.00084	A37	ect	n	179.59	6.2	-0.00	0.00	0.00098
A37	tmao	c	-77.35	25.3	-0.4383	0.0253	76.84	8.93	0.00157	A37	tmao	c	-434.24	85.9	-0.13	0.04	0.18727
A37	tmao	h	17.43	3.6	-0.0439	0.0032	47.25	8.25	0.00005	A37	tmao	h	-15.83	9.7	-0.02	0.00	0.00238
A37	tmao	n	94.34	5.7	0.1294	0.0060	94.46	8.21	0.00006	A37	tmao	n	202.26	24.1	0.03	0.01	0.01477
A43	bet	c	-32.64	2.6	-0.0032	0.0018	8.81	22.27	0.00006	A43	bet	c	-34.34	1.8	-0.00	0.00	0.00009
A43	bet	h	-0.24	0.5	-0.0028	0.0003	2.67	2.97	0.00000	A43	bet	h	-1.27	0.9	-0.00	0.00	0.00002
A43	bet	n	-3.66	54.4	-0.0267	0.1763	1000.00	5218.49	0.00007	A43	bet	n	-18.45	2.0	-0.00	0.00	0.00010
A43	ect	c	-37.72	67.9	0.0459	0.2199	1000.00	3787.81	0.00011	A43	ect	c	-11.80	2.8	-0.00	0.00	0.00019
A43	ect	h	-2.07	23.4	-0.0149	0.0720	922.26	3604.96	0.00002	A43	ect	h	-10.19	1.0	-0.00	0.00	0.00002
A43	ect	n	-43.58	6.3	-0.0087	0.0047	18.69	34.01	0.00026	A43	ect	n	-49.24	4.1	-0.00	0.00	0.00042
A43	tmao	c	-49.32	9.7	0.1379	0.0106	102.31	14.27	0.00017	A43	tmao	c	66.75	25.4	0.03	0.01	0.01638
A43	tmao	h	-17.26	4.5	-0.0760	0.0043	61.47	7.50	0.00006	A43	tmao	h	-77.33	15.7	-0.02	0.01	0.00629
A43	tmao	n	11.22	4.4	-0.2392	0.0045	81.09	3.01	0.00005	A43	tmao	n	-185.17	46.3	-0.07	0.02	0.05450
A46	bet	h	-2.11	0.9	-0.0007	0.0006	2.32	20.42	0.00001	A46	bet	h	-2.36	0.7	-0.00	0.00	0.00001
A46	bet	n	-72.67	1.6	-0.0014	0.0010	1.00	14.97	0.00003	A46	bet	n	-73.08	1.2	-0.00	0.00	0.00004

A46	ect	h	-1.04	28.8	0.0241	0.0932	1000.00	3061.07	0.00002	A46	ect	h	11.30	1.1	0.00	0.00	0.00003
A46	ect	n	-96.37	11.9	0.0427	0.0215	359.32	205.80	0.00004	A46	ect	n	-64.14	4.0	0.00	0.00	0.00041
A46	tmao	h	0.84	9.3	0.0345	0.0146	269.88	145.11	0.00004	A46	tmao	h	29.06	4.3	0.00	0.00	0.00047
A46	tmao	n	-115.67	35.4	0.1207	0.0605	322.27	191.44	0.00043	A46	tmao	n	-19.92	13.4	0.01	0.01	0.00459
A74	bet	c	0.13	72.4	0.0301	0.2344	998.18	6148.61	0.00013	A74	bet	c	15.37	2.4	0.00	0.00	0.00014
A74	bet	h	-0.34	0.7	-0.0055	0.0005	13.92	5.05	0.00000	A74	bet	h	-3.48	1.5	-0.00	0.00	0.00006
A74	bet	n	19.55	8.1	-0.0642	0.0076	60.23	15.70	0.00020	A74	bet	n	-31.70	14.0	-0.02	0.01	0.00501
A74	ect	c	45.50	3.5	-0.0034	0.0028	27.54	64.50	0.00006	A74	ect	c	43.06	1.8	-0.00	0.00	0.00009
A74	ect	h	1.91	7.0	-0.0063	0.0072	86.86	191.75	0.00011	A74	ect	h	-3.60	2.5	-0.00	0.00	0.00015
A74	ect	n	-25.36	31.3	-0.0441	0.0466	239.61	336.29	0.00057	A74	ect	n	-60.57	6.8	-0.01	0.00	0.00118
A74	tmao	c	4.27	8.8	0.0174	0.0075	40.59	44.30	0.00032	A74	tmao	c	17.87	5.7	0.01	0.00	0.00084
A74	tmao	h	3.07	0.9	-0.0029	0.0006	9.36	8.91	0.00001	A74	tmao	h	1.56	1.0	-0.00	0.00	0.00002
A74	tmao	n	-18.70	5.8	-0.0880	0.0052	49.56	6.82	0.00012	A74	tmao	n	-86.44	19.5	-0.03	0.01	0.00969
D101	bet	c	-41.85	6.3	0.0051	0.0061	69.22	172.24	0.00011	D101	bet	c	-37.73	2.3	0.00	0.00	0.00013
D101	ect	c	-73.38	196.7	0.0383	0.6369	1000.00	13151.85	0.00094	D101	ect	c	-51.47	6.3	-0.00	0.00	0.00099
D101	ect	h	1.58	2.6	0.0030	0.0023	51.64	91.83	0.00002	D101	ect	h	3.96	1.2	0.00	0.00	0.00003
D101	ect	n	-66.10	12.7	-0.0176	0.0084	6.59	16.93	0.00159	D101	ect	n	-74.33	9.4	-0.01	0.00	0.00226
D101	tmao	c	-43.74	18.1	-0.0271	0.0096	41.34	29.10	0.00016	D101	tmao	c	-85.99	15.7	-0.01	0.00	0.00123
D101	tmao	h	11.38	2.5	-0.0034	0.0015	1.00	9.51	0.00008	D101	tmao	h	10.36	2.0	-0.00	0.00	0.00010
D101	tmao	n	-124.78	19.2	0.1329	0.0209	103.09	29.46	0.00067	D101	tmao	n	-16.13	22.7	0.03	0.01	0.01305
D12	bet	c	10.49	1.6	-0.0020	0.0013	31.72	59.97	0.00001	D12	bet	c	9.04	0.9	-0.00	0.00	0.00002
D12	bet	h	5.27	0.9	-0.0014	0.0006	1.06	8.29	0.00001	D12	bet	h	4.82	0.8	-0.00	0.00	0.00002
D12	bet	n	-15.17	4.3	-0.0057	0.0047	111.15	164.45	0.00003	D12	bet	n	-19.99	1.5	-0.00	0.00	0.00006
D12	ect	c	-23.59	97.0	0.0699	0.3139	998.92	3551.35	0.00023	D12	ect	c	13.15	3.9	0.00	0.00	0.00039
D12	ect	h	-1.41	1.4	0.0013	0.0012	30.61	73.79	0.00001	D12	ect	h	-0.43	0.7	0.00	0.00	0.00001
D12	ect	n	-14.00	6.9	-0.0029	0.0053	21.31	124.02	0.00030	D12	ect	n	-16.04	3.5	-0.00	0.00	0.00032
D12	tmao	c	2.46	5.4	-0.0674	0.0040	17.60	3.58	0.00020	D12	tmao	c	-38.36	17.5	-0.04	0.01	0.00776
D12	tmao	h	6.51	1.7	-0.0111	0.0014	33.47	11.34	0.00001	D12	tmao	h	-1.26	2.6	-0.00	0.00	0.00017
D12	tmao	n	0.39	7.0	-0.0647	0.0055	25.13	6.37	0.00027	D12	tmao	n	-42.66	16.3	-0.03	0.01	0.00673
D22	bet	c	-14.76	4.0	-0.0039	0.0031	23.95	59.00	0.00009	D22	bet	c	-17.34	2.1	-0.00	0.00	0.00012
D22	bet	h	12.22	1.0	-0.0014	0.0006	1.00	8.97	0.00001	D22	bet	h	11.80	0.8	-0.00	0.00	0.00002
D22	bet	n	-98.97	151.9	-0.0382	0.4920	1000.00	10193.89	0.00056	D22	bet	n	-116.85	4.5	-0.00	0.00	0.00052
D22	ect	c	15.94	7.6	-0.0037	0.0064	38.17	169.80	0.00025	D22	ect	c	12.91	3.3	-0.00	0.00	0.00028
D22	ect	h	14.45	3.6	0.0095	0.0039	100.74	75.63	0.00002	D22	ect	h	22.63	2.1	0.00	0.00	0.00011
D22	ect	n	-259.71	150.0	0.1533	0.4859	1000.00	2508.18	0.00055	D22	ect	n	-178.67	7.2	0.00	0.00	0.00132
D22	tmao	c	-0.82	4.5	-0.0040	0.0027	1.00	14.15	0.00025	D22	tmao	c	-2.00	3.3	-0.00	0.00	0.00028
D22	tmao	h	12.85	1.6	-0.0027	0.0010	1.00	7.55	0.00003	D22	tmao	h	12.03	1.4	-0.00	0.00	0.00005
D22	tmao	n	-93.56	6.2	-0.0831	0.0052	35.55	5.88	0.00017	D22	tmao	n	-153.31	19.5	-0.03	0.01	0.00965
D44	bet	c	-9.65	1.5	0.0024	0.0009	1.00	8.22	0.00003	D44	bet	c	-8.94	1.3	0.00	0.00	0.00004
D44	bet	h	10.15	0.6	-0.0017	0.0004	2.24	5.27	0.00000	D44	bet	h	9.53	0.7	-0.00	0.00	0.00001
D44	bet	n	38.00	22.6	-0.0185	0.0732	1000.00	3125.53	0.00001	D44	bet	n	28.60	0.9	-0.00	0.00	0.00002
D44	ect	c	-17.36	1.3	0.0020	0.0011	30.78	46.31	0.00001	D44	ect	c	-15.87	0.8	0.00	0.00	0.00002
D44	ect	h	20.45	4.3	0.0049	0.0047	97.59	169.92	0.00004	D44	ect	h	24.87	1.6	0.00	0.00	0.00007
D44	ect	n	50.15	6.9	-0.0054	0.0044	3.75	22.61	0.00052	D44	ect	n	47.98	4.8	-0.00	0.00	0.00059
D44	tmao	c	-53.56	159.4	0.0772	0.5159	998.79	5281.03	0.00062	D44	tmao	c	-8.76	5.7	-0.00	0.00	0.00083
D44	tmao	h	17.46	3.8	0.0473	0.0041	94.66	15.26	0.00003	D44	tmao	h	57.09	9.0	0.01	0.00	0.00205
D44	tmao	n	-12.00	23.6	0.1047	0.0302	164.57	72.56	0.00057	D44	tmao	n	78.19	17.3	0.02	0.01	0.00757
D54	bet	c	11.02	20.8	-0.0227	0.0512	642.62	1331.11	0.00003	D54	bet	c	-3.16	1.6	-0.00	0.00	0.00007
D54	bet	h	17.27	1.2	-0.0016	0.0007	1.00	9.77	0.00002	D54	bet	h	16.78	1.0	-0.00	0.00	0.00002
D54	bet	n	-48.33	1.4	-0.0032	0.0008	1.00	5.52	0.00002	D54	bet	n	-49.32	1.4	-0.00	0.00	0.00005
D54	ect	c	-18.76	79.2	0.0634	0.2563	998.93	3194.07	0.00015	D54	ect	c	13.93	3.2	0.00	0.00	0.00026
D54	ect	h	45.11	25.5	0.0336	0.0827	1000.00	1945.46	0.00002	D54	ect	h	63.09	1.5	0.00	0.00	0.00006
D54	ect	n	-10.09	163.4	0.1260	0.5292	1000.00	3324.08	0.00065	D54	ect	n	59.16	7.1	0.00	0.00	0.00127
D54	tmao	c	-42.87	6.2	-0.0142	0.0053	38.83	37.12	0.00016	D54	tmao	c	-53.63	4.4	-0.01	0.00	0.00048
D54	tmao	h	28.49	2.2	0.0574	0.0021	66.66	5.23	0.00001	D54	tmao	h	74.62	11.8	0.02	0.01	0.00354
D54	tmao	n	44.22	8.5	0.1825	0.0085	76.89	7.17	0.00018	D54	tmao	n	193.78	36.4	0.05	0.02	0.03357
D75	bet	c	1.17	22.3	0.0148	0.0721	1000.00	3843.28	0.00001	D75	bet	c	9.24	0.9	0.00	0.00	0.00002

D75	bet	h	9.01	1.3	-0.0010	0.0008	3.60	23.03	0.00002	D75	bet	h	8.63	0.9	-0.00	0.00	0.00002
D75	bet	n	28.37	48.2	-0.0223	0.1563	1000.00	5552.03	0.00006	D75	bet	n	15.96	1.7	0.00	0.00	0.00008
D75	ect	c	17.60	60.9	0.0642	0.1971	999.25	2429.64	0.00009	D75	ect	c	51.69	3.0	0.00	0.00	0.00023
D75	ect	h	10.99	2.6	0.0014	0.0024	61.38	229.63	0.00002	D75	ect	h	12.31	1.0	0.00	0.00	0.00002
D75	ect	n	21.05	123.4	0.0232	0.3995	1000.00	13636.30	0.00037	D75	ect	n	32.89	3.9	0.00	0.00	0.00038
D75	tmao	c	22.04	3.3	0.1010	0.0032	66.62	4.45	0.00003	D75	tmao	c	103.33	20.8	0.03	0.01	0.01102
D75	tmao	h	4.67	4.2	0.0250	0.0057	186.67	61.62	0.00002	D75	tmao	h	25.87	3.7	0.00	0.00	0.00035
D75	tmao	n	43.37	9.5	0.0304	0.0082	41.95	28.15	0.00036	D75	tmao	n	67.05	8.6	0.01	0.00	0.00186
D86	bet	c	-8.39	51.8	-0.0563	0.1678	1000.00	2356.42	0.00007	D86	bet	c	-38.80	2.7	-0.00	0.00	0.00018
D86	bet	h	6.49	0.9	-0.0022	0.0005	2.87	6.51	0.00001	D86	bet	h	5.67	0.9	-0.00	0.00	0.00002
D86	bet	n	-12.33	74.1	-0.0934	0.2401	1000.00	2034.30	0.00013	D86	bet	n	-61.99	4.1	-0.00	0.00	0.00043
D86	ect	c	-189.01	180.3	0.2400	0.5841	1000.00	1925.50	0.00079	D86	ect	c	-65.11	9.5	0.01	0.00	0.00227
D86	ect	h	35.69	3.0	-0.0054	0.0032	98.60	107.44	0.00002	D86	ect	h	31.15	1.3	-0.00	0.00	0.00004
D86	ect	n	-207.01	65.0	0.2087	0.1500	574.31	393.71	0.00043	D86	ect	n	-69.69	13.1	0.01	0.01	0.00433
D86	tmao	c	-54.96	13.0	-0.3084	0.0103	25.81	2.55	0.00094	D86	tmao	c	-261.63	76.1	-0.15	0.03	0.14692
D86	tmao	h	23.49	2.5	0.1185	0.0022	39.82	1.85	0.00003	D86	tmao	h	111.11	27.4	0.05	0.01	0.01903
D86	tmao	n	-18.24	11.7	-0.3283	0.0089	20.72	1.82	0.00086	D86	tmao	n	-226.82	83.7	-0.17	0.04	0.17773
D8	bet	c	8.16	51.1	0.0042	0.1654	1000.00	31225.82	0.00006	D8	bet	c	10.26	1.6	0.00	0.00	0.00006
D8	bet	h	33.13	1.5	-0.0018	0.0009	1.00	10.50	0.00003	D8	bet	h	32.57	1.2	-0.00	0.00	0.00003
D8	bet	n	44.24	2.2	-0.0048	0.0015	12.85	16.10	0.00004	D8	bet	n	41.54	1.8	-0.00	0.00	0.00008
D8	ect	c	76.80	3.6	-0.0074	0.0030	32.73	36.23	0.00006	D8	ect	c	71.48	2.4	-0.00	0.00	0.00015
D8	ect	h	48.08	17.9	0.0034	0.0549	922.75	12205.10	0.00001	D8	ect	h	49.86	0.6	0.00	0.00	0.00001
D8	ect	n	70.09	3.7	-0.0065	0.0024	4.48	10.85	0.00015	D8	ect	n	67.35	3.1	-0.00	0.00	0.00024
D8	tmao	c	35.37	9.9	0.0512	0.0114	121.36	46.11	0.00015	D8	tmao	c	79.43	9.5	0.01	0.00	0.00230
D8	tmao	h	25.52	3.9	0.0145	0.0049	153.57	80.95	0.00002	D8	tmao	h	37.88	2.4	0.00	0.00	0.00015
D8	tmao	n	55.73	37.1	0.0506	0.1202	1000.00	1879.30	0.00003	D8	tmao	n	83.11	2.3	0.00	0.00	0.00013
D93	bet	c	-42.20	4.3	0.0034	0.0036	36.69	101.84	0.00008	D93	bet	c	-39.74	2.0	0.00	0.00	0.00010
D93	bet	h	-5.22	0.6	-0.0010	0.0005	28.40	40.99	0.00000	D93	bet	h	-5.94	0.4	-0.00	0.00	0.00000
D93	bet	n	-43.25	38.9	0.0017	0.1260	1000.00	59195.31	0.00004	D93	bet	n	-42.21	1.2	-0.00	0.00	0.00004
D93	ect	c	-12.24	61.1	0.0460	0.1979	1000.00	3401.39	0.00009	D93	ect	c	11.70	2.4	0.00	0.00	0.00015
D93	ect	h	-21.03	28.6	0.0302	0.0927	999.53	2430.04	0.00002	D93	ect	h	-4.89	1.5	0.00	0.00	0.00005
D93	ect	n	-64.70	6.2	-0.0068	0.0043	10.16	27.70	0.00033	D93	ect	n	-68.47	4.2	-0.00	0.00	0.00044
D93	tmao	c	12.19	11.6	0.0135	0.0102	45.29	82.86	0.00052	D93	tmao	c	23.03	5.7	0.00	0.00	0.00082
D93	tmao	h	5.07	8.6	0.0159	0.0134	264.50	284.82	0.00004	D93	tmao	h	18.61	2.4	0.00	0.00	0.00015
D93	tmao	n	-45.04	2.8	-0.0009	0.0017	1.00	39.48	0.00010	D93	tmao	n	-45.29	2.0	-0.00	0.00	0.00010
E29	bet	c	-27.72	1.0	0.0015	0.0006	4.42	11.94	0.00001	E29	bet	c	-27.08	0.8	0.00	0.00	0.00002
E29	bet	h	-6.22	0.7	-0.0018	0.0004	1.00	4.84	0.00001	E29	bet	h	-6.78	0.7	-0.00	0.00	0.00001
E29	bet	n	-64.87	19.6	-0.0297	0.0317	288.67	381.94	0.00016	E29	bet	n	-87.04	3.4	-0.00	0.00	0.00030
E29	ect	c	-96.76	23.7	0.0470	0.0451	401.44	419.82	0.00013	E29	ect	c	-61.99	4.5	0.00	0.00	0.00052
E29	ect	h	-6.85	29.2	0.0150	0.0944	999.28	4989.66	0.00002	E29	ect	h	1.18	1.1	0.00	0.00	0.00003
E29	ect	n	-98.13	59.2	0.0544	0.1917	1000.00	2785.70	0.00008	E29	ect	n	-69.26	2.7	0.00	0.00	0.00018
E29	tmao	c	-23.54	4.6	-0.0860	0.0035	20.50	2.68	0.00013	E29	tmao	c	-78.19	22.2	-0.04	0.01	0.01247
E29	tmao	h	-3.73	3.6	0.0164	0.0040	107.87	47.18	0.00002	E29	tmao	h	10.08	3.1	0.00	0.00	0.00024
E29	tmao	n	-26.30	6.2	-0.0782	0.0052	35.30	6.21	0.00018	E29	tmao	n	-83.33	19.1	-0.03	0.01	0.00928
E60	bet	c	-40.43	19.6	0.0291	0.0475	626.42	945.73	0.00003	E60	bet	c	-21.57	2.0	0.00	0.00	0.00011
E60	bet	h	-5.58	0.8	-0.0022	0.0006	5.58	8.24	0.00001	E60	bet	h	-6.56	0.8	-0.00	0.00	0.00002
E60	bet	n	-128.94	193.9	-0.1536	0.6274	998.51	3228.47	0.00091	E60	bet	n	-207.81	7.7	-0.00	0.00	0.00150
E60	ect	c	19.65	4.1	-0.0047	0.0032	22.96	48.06	0.00010	E60	ect	c	16.29	2.4	-0.00	0.00	0.00015
E60	ect	h	37.83	6.7	0.0072	0.0115	326.99	612.66	0.00001	E60	ect	h	43.62	1.1	0.00	0.00	0.00003
E60	ect	n	-146.48	124.8	0.0617	0.4038	999.06	5171.33	0.00038	E60	ect	n	-115.42	4.2	0.00	0.00	0.00044
E60	tmao	c	-12.04	6.9	-0.0219	0.0068	75.07	47.39	0.00012	E60	tmao	c	-29.55	4.6	-0.01	0.00	0.00054
E60	tmao	h	0.74	2.4	-0.0165	0.0019	24.44	8.36	0.00003	E60	tmao	h	-10.14	4.2	-0.01	0.00	0.00045
E60	tmao	n	-114.68	3.8	-0.0292	0.0032	38.05	10.73	0.00006	E60	tmao	n	-136.05	7.0	-0.01	0.00	0.00123
E73	bet	c	6.48	2.2	-0.0007	0.0016	13.08	119.80	0.00004	E73	bet	c	6.07	1.3	-0.00	0.00	0.00004
E73	bet	h	5.47	1.0	-0.0014	0.0006	8.93	19.03	0.00001	E73	bet	h	4.74	0.7	-0.00	0.00	0.00001
E73	bet	n	32.09	12.2	-0.0088	0.0209	324.73	907.35	0.00005	E73	bet	n	25.00	1.7	-0.00	0.00	0.00007
E73	ect	c	-57.65	172.3	0.1331	0.5579	1000.00	3317.50	0.00072	E73	ect	c	14.44	7.4	0.00	0.00	0.00138

E73	ect	h	8.77	1.7	0.0036	0.0017	65.70	65.07	0.00001	E73	ect	h	11.77	1.0	0.00	0.00	0.00003
E73	ect	n	32.81	130.4	0.0418	0.3683	807.63	6028.95	0.00074	E73	ect	n	58.97	5.8	-0.00	0.00	0.00085
E73	tmao	c	28.50	4.8	0.0115	0.0055	124.08	101.73	0.00003	E73	tmao	c	38.30	2.3	0.00	0.00	0.00014
E73	tmao	h	8.97	10.7	0.0202	0.0170	275.39	292.92	0.00005	E73	tmao	h	25.87	2.9	0.00	0.00	0.00022
E73	tmao	n	16.55	8.0	0.0418	0.0090	113.97	42.73	0.00010	E73	tmao	n	52.20	7.8	0.01	0.00	0.00155
F106	bet	c	-33.21	64.1	-0.0387	0.2075	998.16	4231.67	0.00010	F106	bet	c	-53.16	2.3	-0.00	0.00	0.00014
F106	bet	h	5.28	0.9	-0.0020	0.0006	1.72	6.46	0.00001	F106	bet	h	4.61	0.9	-0.00	0.00	0.00002
F106	bet	n	4.32	1.8	-0.0020	0.0011	1.00	11.69	0.00004	F106	bet	n	3.70	1.4	-0.00	0.00	0.00005
F106	ect	c	-2.19	181.5	-0.0648	0.5879	1000.00	7185.02	0.00080	F106	ect	c	-37.82	6.2	-0.00	0.00	0.00096
F106	ect	h	13.34	1.6	0.0012	0.0013	29.43	84.65	0.00001	F106	ect	h	14.25	0.8	0.00	0.00	0.00002
F106	ect	n	-22.55	111.2	0.0393	0.3601	1000.00	7248.31	0.00030	F106	ect	n	-0.92	3.8	0.00	0.00	0.00036
F106	tmao	c	-45.12	132.8	0.0041	0.4299	999.99	82303.12	0.00043	F106	tmao	c	-39.53	3.6	-0.00	0.00	0.00033
F106	tmao	h	-12.88	7.5	0.0230	0.0124	299.91	196.89	0.00002	F106	tmao	h	5.32	2.6	0.00	0.00	0.00017
F106	tmao	n	95.18	13.3	0.0665	0.0174	175.18	68.71	0.00017	F106	tmao	n	151.75	10.3	0.01	0.00	0.00270
F56	bet	c	2.53	47.1	-0.0198	0.1524	1000.00	6088.27	0.00005	F56	bet	c	-8.99	1.6	0.00	0.00	0.00007
F56	bet	h	-13.59	0.5	-0.0021	0.0003	1.00	3.29	0.00000	F56	bet	h	-14.24	0.8	-0.00	0.00	0.00001
F56	bet	n	-78.35	3.4	0.0036	0.0024	11.63	31.79	0.00010	F56	bet	n	-76.41	2.2	0.00	0.00	0.00012
F56	ect	c	-70.88	213.9	0.1289	0.6927	1000.00	4253.73	0.00111	F56	ect	c	0.28	8.3	0.00	0.00	0.00176
F56	ect	h	-37.96	30.4	0.0313	0.0984	998.28	2485.50	0.00002	F56	ect	h	-21.67	1.4	0.00	0.00	0.00005
F56	ect	n	-195.75	84.2	0.0892	0.2726	998.84	2415.40	0.00017	F56	ect	n	-149.43	3.9	0.00	0.00	0.00039
F56	tmao	c	-36.15	10.6	0.0028	0.0080	19.89	183.52	0.00072	F56	tmao	c	-34.14	5.4	0.00	0.00	0.00074
F56	tmao	h	-10.46	2.6	-0.0195	0.0023	42.26	12.18	0.00003	F56	tmao	h	-24.90	4.4	-0.01	0.00	0.00050
F56	tmao	n	-115.93	8.0	-0.1121	0.0068	40.13	6.18	0.00026	F56	tmao	n	-198.66	25.8	-0.04	0.01	0.01689
F7	bet	c	-54.78	51.0	0.0887	0.1550	905.39	1291.03	0.00008	F7	bet	c	-5.40	4.0	0.00	0.00	0.00041
F7	bet	h	23.47	1.3	-0.0019	0.0008	1.54	9.52	0.00002	F7	bet	h	22.83	1.1	-0.00	0.00	0.00003
F7	bet	n	43.80	3.4	-0.0022	0.0024	11.86	51.99	0.00009	F7	bet	n	42.56	2.0	-0.00	0.00	0.00010
F7	ect	c	13.23	6.3	-0.0041	0.0038	1.00	19.36	0.00049	F7	ect	c	11.94	4.6	-0.00	0.00	0.00054
F7	ect	h	34.33	2.1	0.0013	0.0021	81.29	252.24	0.00001	F7	ect	h	35.54	0.7	0.00	0.00	0.00001
F7	ect	n	57.49	6.5	-0.0031	0.0046	13.35	76.52	0.00032	F7	ect	n	55.67	3.7	-0.00	0.00	0.00035
F7	tmao	c	-18.11	111.0	0.0468	0.3595	1000.00	6085.36	0.00030	F7	tmao	c	9.04	3.8	-0.00	0.00	0.00037
F7	tmao	h	4.26	1.5	-0.0026	0.0009	1.00	7.16	0.00003	F7	tmao	h	3.44	1.3	-0.00	0.00	0.00004
F7	tmao	n	51.78	5.7	-0.0048	0.0040	11.32	39.41	0.00027	F7	tmao	n	49.13	3.6	-0.00	0.00	0.00032
F82	bet	c	-3.47	663.4	-0.1092	2.1487	1000.00	15567.92	0.01066	F82	bet	c	-76.68	19.7	0.01	0.01	0.00981
F82	bet	h	16.20	1.3	-0.0023	0.0008	3.60	9.59	0.00002	F82	bet	h	15.28	1.1	-0.00	0.00	0.00003
F82	bet	n	-79.76	172.4	-0.1788	0.5584	1000.00	2471.50	0.00072	F82	bet	n	-174.57	8.4	-0.00	0.00	0.00180
F82	ect	c	-19.73	41.0	0.0090	0.0526	165.73	1482.13	0.00171	F82	ect	c	-10.18	8.4	0.00	0.00	0.00179
F82	ect	h	35.93	23.1	-0.0761	0.0749	999.56	778.65	0.00001	F82	ect	h	-3.45	2.6	-0.00	0.00	0.00017
F82	ect	n	-177.11	17.6	-0.0531	0.0235	183.28	119.53	0.00027	F82	ect	n	-222.55	8.6	-0.01	0.00	0.00189
F82	tmao	c	16.86	37.1	0.1980	0.0360	69.48	26.37	0.00368	F82	tmao	c	173.82	39.8	0.06	0.02	0.04013
F82	tmao	h	-0.95	4.1	-0.1110	0.0040	74.79	5.53	0.00004	F82	tmao	h	-91.10	21.9	-0.03	0.01	0.01216
F82	tmao	n	-77.06	9.1	-0.3638	0.0091	76.74	3.87	0.00020	F82	tmao	n	-373.40	71.2	-0.11	0.03	0.12860
G34	bet	c	-68.56	64.1	-0.0345	0.2075	1000.00	4763.88	0.00010	G34	bet	c	-88.15	2.4	0.00	0.00	0.00014
G34	bet	h	4.50	0.7	-0.0019	0.0005	3.59	6.80	0.00001	G34	bet	h	3.76	0.7	-0.00	0.00	0.00001
G34	bet	n	-80.66	130.2	-0.0964	0.4216	1000.00	3459.82	0.00041	G34	bet	n	-130.33	5.0	-0.00	0.00	0.00064
G34	ect	c	-93.52	5.5	-0.0043	0.0039	13.27	46.95	0.00023	G34	ect	c	-96.04	3.3	-0.00	0.00	0.00027
G34	ect	h	4.90	28.4	0.0120	0.0921	1000.00	6065.49	0.00002	G34	ect	h	11.61	1.0	-0.00	0.00	0.00003
G34	ect	n	-103.80	5.9	-0.0030	0.0044	16.48	85.82	0.00025	G34	ect	n	-105.79	3.3	-0.00	0.00	0.00027
G34	tmao	c	-43.47	17.7	-0.0825	0.0286	285.92	122.83	0.00013	G34	tmao	c	-107.76	8.5	-0.01	0.00	0.00185
G34	tmao	h	11.60	1.1	-0.0033	0.0007	2.37	5.18	0.00001	G34	tmao	h	10.42	1.3	-0.00	0.00	0.00004
G34	tmao	n	-103.39	15.9	0.0890	0.0329	470.28	178.44	0.00004	G34	tmao	n	-41.13	6.3	0.01	0.00	0.00102
G40	bet	c	-79.06	36.4	-0.0189	0.0733	448.24	1818.61	0.00023	G40	bet	c	-92.14	3.3	-0.00	0.00	0.00027
G40	bet	h	-5.90	0.8	-0.0025	0.0006	17.51	13.95	0.00000	G40	bet	h	-7.42	0.8	-0.00	0.00	0.00001
G40	bet	n	-168.96	252.5	-0.2460	0.8171	999.05	2626.50	0.00155	G40	bet	n	-297.71	11.6	-0.01	0.01	0.00344
G40	ect	c	-95.56	85.2	0.1486	0.2757	998.44	1465.97	0.00018	G40	ect	c	-16.21	6.2	0.00	0.00	0.00098
G40	ect	h	15.26	2.5	0.0101	0.0043	339.97	167.97	0.00000	G40	ect	h	23.13	1.0	0.00	0.00	0.00003
G40	ect	n	-136.15	101.8	0.2035	0.3296	999.38	1280.82	0.00025	G40	ect	n	-29.08	7.9	0.00	0.00	0.00157
G40	tmao	c	-38.42	14.4	0.3031	0.0148	86.13	8.14	0.00045	G40	tmao	c	212.16	58.0	0.08	0.03	0.08539

G40	tmao	h	18.08	6.6	0.0628	0.0074	114.23	23.54	0.00007	G40	tmao	h	71.43	11.3	0.01	0.01	0.00325
G40	tmao	n	-45.99	17.8	0.6850	0.0188	93.24	4.82	0.00064	G40	tmao	n	523.13	127.1	0.18	0.06	0.40970
G48	bet	c	-25.54	3.5	0.0033	0.0032	50.04	111.97	0.00004	G48	bet	c	-22.99	1.5	0.00	0.00	0.00006
G48	bet	h	5.85	0.8	-0.0018	0.0005	2.63	6.57	0.00001	G48	bet	h	5.19	0.7	-0.00	0.00	0.00001
G48	bet	n	-22.07	49.2	-0.0090	0.1592	999.39	14053.06	0.00006	G48	bet	n	-27.51	1.5	0.00	0.00	0.00006
G48	ect	c	-64.20	74.2	0.0205	0.2404	1000.00	9257.69	0.00013	G48	ect	c	-53.79	2.4	0.00	0.00	0.00014
G48	ect	h	14.90	26.9	0.0045	0.0871	1000.00	15331.62	0.00002	G48	ect	h	17.32	0.8	0.00	0.00	0.00002
G48	ect	n	-54.46	122.8	0.0389	0.3977	1000.00	8082.63	0.00037	G48	ect	n	-31.19	4.0	-0.00	0.00	0.00040
G48	tmao	c	57.12	90.6	-0.1827	0.2934	1000.00	1271.08	0.00020	G48	tmao	c	-38.02	6.8	-0.00	0.00	0.00116
G48	tmao	h	11.35	1.7	-0.0025	0.0010	1.00	8.82	0.00004	G48	tmao	h	10.63	1.4	-0.00	0.00	0.00005
G48	tmao	n	-4.10	1.9	-0.0017	0.0011	1.00	14.35	0.00004	G48	tmao	n	-4.61	1.4	-0.00	0.00	0.00005
G52	bet	c	-37.41	34.1	0.0352	0.1104	1000.00	2481.53	0.00003	G52	bet	c	-18.36	1.7	0.00	0.00	0.00007
G52	bet	h	-11.12	1.3	-0.0021	0.0008	4.28	12.00	0.00002	G52	bet	h	-11.97	1.0	-0.00	0.00	0.00003
G52	bet	n	27.95	4.3	-0.0062	0.0026	1.00	8.79	0.00023	G52	bet	n	26.07	3.5	-0.00	0.00	0.00032
G52	ect	c	-27.20	6.7	0.0228	0.0078	130.20	74.51	0.00006	G52	ect	c	-8.54	3.7	0.01	0.00	0.00035
G52	ect	h	1.55	60.6	-0.0153	0.1961	1000.00	10140.75	0.00009	G52	ect	h	-7.43	1.9	0.00	0.00	0.00010
G52	ect	n	61.43	130.8	0.0165	0.4237	1000.00	20337.17	0.00041	G52	ect	n	72.67	3.9	-0.00	0.00	0.00039
G52	tmao	c	-1.50	5.7	0.0554	0.0056	72.02	14.87	0.00008	G52	tmao	c	43.92	11.6	0.02	0.01	0.00341
G52	tmao	h	-12.18	8.8	0.0353	0.0156	346.55	176.61	0.00002	G52	tmao	h	15.20	3.6	0.00	0.00	0.00033
G52	tmao	n	37.64	7.1	0.0339	0.0068	62.50	27.01	0.00015	G52	tmao	n	65.46	8.0	0.01	0.00	0.00163
G53	bet	c	-16.53	3.2	-0.0036	0.0019	1.00	11.18	0.00013	G53	bet	c	-17.62	2.5	-0.00	0.00	0.00016
G53	bet	h	-3.58	0.6	-0.0003	0.0003	1.11	24.41	0.00000	G53	bet	h	-3.67	0.4	-0.00	0.00	0.00000
G53	bet	n	-14.58	5.8	-0.0177	0.0080	194.43	126.43	0.00003	G53	bet	n	-29.52	2.7	-0.00	0.00	0.00019
G53	ect	c	28.61	120.8	0.0334	0.3912	1000.00	9261.89	0.00035	G53	ect	c	47.40	4.0	-0.00	0.00	0.00040
G53	ect	h	-5.06	2.2	0.0088	0.0027	132.33	66.16	0.00001	G53	ect	h	2.49	1.6	0.00	0.00	0.00006
G53	ect	n	-183.91	147.6	0.1130	0.4780	1000.00	3346.26	0.00053	G53	ect	n	-123.78	6.2	0.00	0.00	0.00097
G53	tmao	c	36.94	6.1	0.0219	0.0050	31.49	19.88	0.00018	G53	tmao	c	52.64	6.1	0.01	0.00	0.00095
G53	tmao	h	-6.55	19.8	0.0235	0.0454	568.84	1050.12	0.00004	G53	tmao	h	9.74	2.1	0.00	0.00	0.00011
G53	tmao	n	-53.26	14.7	0.0313	0.0203	202.31	186.32	0.00016	G53	tmao	n	-26.96	5.0	0.00	0.00	0.00064
G61	bet	c	-54.69	52.1	-0.0531	0.1684	998.34	2508.37	0.00007	G61	bet	c	-82.37	2.4	-0.00	0.00	0.00015
G61	bet	h	3.10	0.9	-0.0014	0.0005	1.00	7.62	0.00001	G61	bet	h	2.65	0.8	-0.00	0.00	0.00001
G61	bet	n	-104.77	152.5	-0.0380	0.4939	1000.00	10278.05	0.00056	G61	bet	n	-126.50	4.9	0.00	0.00	0.00062
G61	ect	c	-59.49	4.1	0.0028	0.0031	17.56	66.45	0.00012	G61	ect	c	-57.75	2.3	0.00	0.00	0.00013
G61	ect	h	8.39	6.8	0.0064	0.0104	256.41	538.68	0.00002	G61	ect	h	13.93	1.3	0.00	0.00	0.00004
G61	ect	n	-112.36	117.5	0.0573	0.3806	1000.00	5254.92	0.00033	G61	ect	n	-81.34	4.2	0.00	0.00	0.00046
G61	tmao	c	-60.97	41.4	0.0539	0.0904	519.83	861.80	0.00022	G61	tmao	c	-21.94	5.1	0.00	0.00	0.00067
G61	tmao	h	6.10	15.0	0.0398	0.0299	440.46	347.67	0.00004	G61	tmao	h	35.22	3.5	0.00	0.00	0.00031
G61	tmao	n	-75.77	28.9	0.2206	0.0394	192.96	49.69	0.00068	G61	tmao	n	108.59	30.9	0.03	0.01	0.02415
G65	bet	c	-11.97	44.1	-0.0418	0.1427	1000.00	2699.07	0.00005	G65	bet	c	-33.22	1.8	-0.00	0.00	0.00008
G65	bet	h	14.07	1.2	-0.0018	0.0007	1.82	9.21	0.00002	G65	bet	h	13.46	1.0	-0.00	0.00	0.00002
G65	bet	n	100.93	83.1	0.0145	0.2692	1000.00	14647.87	0.00017	G65	bet	n	109.21	2.6	-0.00	0.00	0.00018
G65	ect	c	-4.62	60.1	-0.0098	0.1818	900.26	13708.55	0.00012	G65	ect	c	-10.89	2.2	0.00	0.00	0.00012
G65	ect	h	18.59	4.4	0.0061	0.0059	186.85	263.38	0.00002	G65	ect	h	23.82	1.2	0.00	0.00	0.00004
G65	ect	n	106.09	3.9	-0.0018	0.0023	1.00	28.10	0.00019	G65	ect	n	105.55	2.8	-0.00	0.00	0.00020
G65	tmao	c	-16.38	6.2	0.0084	0.0055	47.67	73.73	0.00014	G65	tmao	c	-9.59	3.2	0.00	0.00	0.00025
G65	tmao	h	11.03	2.0	-0.0070	0.0020	82.15	46.17	0.00001	G65	tmao	h	5.37	1.4	-0.00	0.00	0.00005
G65	tmao	n	78.00	8.1	0.0498	0.0094	124.84	40.04	0.00010	G65	tmao	n	120.56	8.9	0.01	0.00	0.00201
G68	bet	c	-52.77	141.2	-0.0086	0.4574	1000.00	42092.84	0.00048	G68	bet	c	-59.34	4.3	0.00	0.00	0.00046
G68	bet	h	27.71	1.6	-0.0021	0.0009	1.00	9.60	0.00003	G68	bet	h	27.05	1.3	-0.00	0.00	0.00004
G68	bet	n	-75.67	8.9	-0.0017	0.0080	48.31	552.21	0.00029	G68	bet	n	-77.21	3.4	-0.00	0.00	0.00030
G68	ect	c	-82.42	73.5	0.0929	0.2380	1000.00	2028.05	0.00013	G68	ect	c	-33.12	4.1	0.00	0.00	0.00042
G68	ect	h	36.24	18.7	0.0136	0.0606	1000.00	3520.29	0.00001	G68	ect	h	43.44	0.8	0.00	0.00	0.00001
G68	ect	n	-108.84	108.4	0.0905	0.3512	1000.00	3070.19	0.00028	G68	ect	n	-61.25	4.6	0.00	0.00	0.00054
G68	tmao	c	-39.40	16.2	0.0185	0.0207	165.75	283.22	0.00027	G68	tmao	c	-23.87	4.2	0.00	0.00	0.00045
G68	tmao	h	33.86	1.6	-0.0013	0.0010	1.00	16.08	0.00003	G68	tmao	h	33.49	1.2	-0.00	0.00	0.00003
G68	tmao	n	-28.95	104.2	0.0916	0.2968	818.35	2237.47	0.00045	G68	tmao	n	26.15	6.2	0.00	0.00	0.00097
G81	bet	c	-20.37	13.3	0.0146	0.0122	54.76	103.92	0.00058	G81	bet	c	-8.16	6.0	0.00	0.00	0.00093

G81	bet	h	-1.94	0.6	-0.0021	0.0004	1.00	3.77	0.00000	G81	bet	h	-2.60	0.8	-0.00	0.00	0.00002
G81	bet	n	-81.83	33.1	0.0073	0.0575	335.54	3090.29	0.00035	G81	bet	n	-74.44	3.8	-0.00	0.00	0.00036
G81	ect	c	-236.07	1275.5	0.2138	4.1310	1000.00	15292.72	0.03942	G81	ect	c	-115.15	40.3	-0.00	0.02	0.04119
G81	ect	h	-14.28	21.6	0.0027	0.0701	1000.00	20867.03	0.00001	G81	ect	h	-12.63	0.7	-0.00	0.00	0.00001
G81	ect	n	-168.62	103.3	0.2303	0.3346	999.49	1149.28	0.00026	G81	ect	n	-47.02	9.0	0.00	0.00	0.00206
G81	tmao	c	-60.08	24.1	-0.2010	0.0199	33.64	8.98	0.00272	G81	tmao	c	-203.97	49.2	-0.09	0.02	0.06140
G81	tmao	h	-20.19	3.1	-0.0681	0.0030	69.98	6.47	0.00003	G81	tmao	h	-75.03	13.7	-0.02	0.01	0.00474
G81	tmao	n	-105.69	7.5	0.2831	0.0077	86.25	4.56	0.00012	G81	tmao	n	128.03	53.8	0.08	0.02	0.07352
G9	bet	h	7.09	0.5	-0.0020	0.0003	4.29	5.09	0.00000	G9	bet	h	6.25	0.7	-0.00	0.00	0.00001
G9	bet	n	11.89	2.7	-0.0033	0.0018	6.49	18.70	0.00007	G9	bet	n	10.36	1.9	-0.00	0.00	0.00009
G9	ect	c	-395.66	562.1	0.3135	1.2736	556.35	2180.87	0.03448	G9	ect	c	-183.23	42.3	0.01	0.02	0.04535
G9	ect	h	26.07	0.9	-0.0015	0.0006	1.00	7.67	0.00001	G9	ect	h	25.62	0.8	-0.00	0.00	0.00002
G9	ect	n	115.63	10.4	-0.0158	0.0079	21.49	34.43	0.00066	G9	ect	n	105.32	6.6	-0.01	0.00	0.00110
G9	tmao	c	-47.50	4.9	0.0094	0.0037	20.11	25.80	0.00015	G9	tmao	c	-41.46	3.5	0.00	0.00	0.00031
G9	tmao	h	-0.30	2.9	0.0164	0.0030	80.09	28.66	0.00002	G9	tmao	h	13.35	3.4	0.00	0.00	0.00030
G9	tmao	n	-10.42	5.0	0.0982	0.0043	43.46	4.73	0.00010	G9	tmao	n	63.76	22.7	0.04	0.01	0.01303
H18	bet	c	-79.40	102.6	-0.0091	0.3323	1000.00	28968.59	0.00026	H18	bet	c	-84.38	3.2	-0.00	0.00	0.00026
H18	bet	h	6.47	0.6	-0.0020	0.0004	1.00	3.85	0.00000	H18	bet	h	5.87	0.7	-0.00	0.00	0.00001
H18	bet	n	-48.15	61.8	-0.0332	0.2000	1000.00	4764.66	0.00009	H18	bet	n	-66.32	2.3	-0.00	0.00	0.00014
H18	ect	c	-138.89	6.1	0.0228	0.0068	112.44	58.84	0.00006	H18	ect	c	-119.63	4.3	0.01	0.00	0.00047
H18	ect	h	20.89	3.3	0.0046	0.0040	144.72	202.98	0.00001	H18	ect	h	24.88	1.1	0.00	0.00	0.00003
H18	ect	n	-66.59	5.6	0.0082	0.0051	55.98	78.27	0.00010	H18	ect	n	-59.90	2.8	0.00	0.00	0.00019
H18	tmao	c	-69.80	10.7	-0.0188	0.0073	8.76	15.82	0.00104	H18	tmao	c	-79.21	8.3	-0.01	0.00	0.00174
H18	tmao	h	9.74	2.7	0.0148	0.0028	88.39	31.55	0.00002	H18	tmao	h	22.16	3.0	0.00	0.00	0.00023
H18	tmao	n	-86.90	5.9	-0.0112	0.0036	1.00	6.76	0.00044	H18	tmao	n	-90.32	5.4	-0.01	0.00	0.00073
I109	bet	c	12.90	102.2	-0.0411	0.3308	1000.00	6369.48	0.00025	I109	bet	c	-9.33	3.5	-0.00	0.00	0.00032
I109	bet	h	-22.43	0.9	-0.0014	0.0006	1.00	8.72	0.00001	I109	bet	h	-22.86	0.8	-0.00	0.00	0.00002
I109	bet	n	-287.66	21.6	0.0159	0.0318	232.91	625.99	0.00028	I109	bet	n	-275.97	3.4	0.00	0.00	0.00030
I109	ect	c	51.70	116.1	-0.1071	0.3761	1000.00	2779.64	0.00033	I109	ect	c	-3.21	4.8	-0.00	0.00	0.00059
I109	ect	h	-26.65	23.7	0.0226	0.0768	997.90	2679.30	0.00001	I109	ect	h	-14.79	1.1	0.00	0.00	0.00003
I109	ect	n	-66.67	22.4	-0.0047	0.0324	225.03	2126.25	0.00032	I109	ect	n	-71.87	3.6	0.00	0.00	0.00034
I109	tmao	c	-11.53	3.0	-0.0188	0.0020	5.50	3.43	0.00009	I109	tmao	c	-19.75	5.7	-0.01	0.00	0.00083
I109	tmao	h	4.61	4.8	0.0205	0.0047	72.53	34.03	0.00006	I109	tmao	h	21.53	4.6	0.01	0.00	0.00053
I109	tmao	n	-9.98	29.7	0.3898	0.0289	70.00	10.79	0.00234	I109	tmao	n	302.63	77.6	0.12	0.04	0.15287
I25	bet	c	-13.51	6.1	-0.0094	0.0097	278.43	360.22	0.00002	I25	bet	c	-21.21	1.4	-0.00	0.00	0.00005
I25	bet	h	-5.37	1.1	-0.0020	0.0007	1.00	6.97	0.00001	I25	bet	h	-5.97	1.0	-0.00	0.00	0.00002
I25	bet	n	-171.77	8.6	0.0075	0.0063	16.21	48.47	0.00052	I25	bet	n	-167.29	4.9	0.00	0.00	0.00062
I25	ect	c	-106.92	126.2	0.1606	0.4083	998.47	2009.78	0.00039	I25	ect	c	-22.76	6.8	0.00	0.00	0.00118
I25	ect	h	139.93	150.3	-0.2191	0.4869	1000.00	1758.10	0.00055	I25	ect	h	24.18	9.1	-0.00	0.00	0.00212
I25	ect	n	258.30	41.5	-0.2278	0.0902	515.67	202.51	0.00022	I25	ect	n	101.16	15.9	-0.01	0.01	0.00645
I25	tmao	c	29.64	102.1	-0.0967	0.3306	1000.00	2704.62	0.00025	I25	tmao	c	-23.84	4.9	-0.00	0.00	0.00062
I25	tmao	h	7.48	2.7	0.0497	0.0027	74.54	8.17	0.00002	I25	tmao	h	48.18	10.1	0.01	0.00	0.00258
I25	tmao	n	-105.33	5.5	-0.0689	0.0038	11.64	2.65	0.00025	I25	tmao	n	-142.83	19.0	-0.04	0.01	0.00919
I4	bet	c	6.87	1.6	-0.0029	0.0010	1.00	7.08	0.00003	I4	bet	c	6.00	1.4	-0.00	0.00	0.00005
I4	bet	h	-33.64	1.6	-0.0021	0.0010	1.00	9.76	0.00003	I4	bet	h	-34.31	1.3	-0.00	0.00	0.00004
I4	bet	n	-133.81	7.3	-0.0035	0.0054	18.16	96.00	0.00036	I4	bet	n	-136.12	3.9	-0.00	0.00	0.00039
I4	ect	c	-18.17	111.6	0.0417	0.3614	1000.00	6854.39	0.00030	I4	ect	c	5.73	3.8	-0.00	0.00	0.00037
I4	ect	h	-47.30	14.2	0.0463	0.0459	999.47	783.21	0.00000	I4	ect	h	-23.12	1.6	0.00	0.00	0.00007
I4	ect	n	-235.60	21.1	0.1198	0.0442	483.74	181.29	0.00007	I4	ect	n	-151.64	8.7	0.01	0.00	0.00193
I4	tmao	c	128.63	86.1	-0.1209	0.2786	999.07	1821.86	0.00018	I4	tmao	c	63.72	5.4	-0.00	0.00	0.00073
I4	tmao	h	-44.30	5.2	0.0249	0.0069	174.94	72.22	0.00003	I4	tmao	h	-23.45	3.7	0.00	0.00	0.00035
I4	tmao	n	-117.99	7.1	-0.0103	0.0046	6.17	15.44	0.00050	I4	tmao	n	-122.76	5.4	-0.01	0.00	0.00074
I51	bet	c	13.29	23.7	-0.0168	0.0766	998.49	3609.19	0.00001	I51	bet	c	4.39	1.0	-0.00	0.00	0.00002
I51	bet	h	16.95	48.8	0.0067	0.1580	1000.00	18671.07	0.00006	I51	bet	h	21.91	1.4	-0.00	0.00	0.00005
I51	bet	n	61.70	3.4	-0.0021	0.0021	1.00	20.65	0.00015	I51	bet	n	61.05	2.5	-0.00	0.00	0.00016
I51	ect	c	14.11	68.3	-0.0038	0.2213	1000.00	46552.83	0.00011	I51	ect	c	11.82	2.1	0.00	0.00	0.00011
I51	ect	h	41.44	2.5	0.0052	0.0019	16.04	20.34	0.00005	I51	ect	h	44.57	1.9	0.00	0.00	0.00010

I51	ect	n	110.31	147.2	0.0296	0.4768	1000.00	12758.63	0.00053	I51	ect	n	125.49	4.6	0.00	0.00	0.00055
I51	tmao	c	-13.99	6.0	-0.0312	0.0049	33.44	14.32	0.00017	I51	tmao	c	-35.78	7.5	-0.01	0.00	0.00141
I51	tmao	h	11.92	12.7	0.0876	0.0139	104.65	30.04	0.00029	I51	tmao	h	86.83	16.9	0.02	0.01	0.00726
I51	tmao	n	72.11	12.9	0.2569	0.0125	67.76	6.94	0.00046	I51	tmao	n	280.29	53.5	0.08	0.02	0.07275
I55	bet	c	-58.86	76.5	-0.0077	0.2478	1000.00	25474.34	0.00014	I55	bet	c	-64.10	2.3	0.00	0.00	0.00014
I55	bet	h	9.99	0.8	-0.0017	0.0005	2.25	7.21	0.00001	I55	bet	h	9.39	0.7	-0.00	0.00	0.00001
I55	bet	n	-48.78	40.7	-0.0172	0.1318	1000.00	6056.10	0.00004	I55	bet	n	-57.73	1.4	-0.00	0.00	0.00005
I55	ect	c	-560413.41	932483.2	839.3772	3020.0055	1000.00	2846.98	21067.86413	I55	ect	c	-113966.90	42240.1	14.37	19.35	45262.23620
I55	ect	h	5.49	20.0	0.0113	0.0647	1000.00	4533.02	0.00001	I55	ect	h	11.66	0.8	0.00	0.00	0.00001
I55	ect	n	-106.00	127.9	0.0467	0.4141	1000.00	7013.41	0.00040	I55	ect	n	-79.69	4.4	-0.00	0.00	0.00048
I55	tmao	c	-65.79	2.6	-0.0042	0.0016	1.00	7.96	0.00009	I55	tmao	c	-67.06	2.2	-0.00	0.00	0.00013
I55	tmao	h	-3.26	2.0	-0.0107	0.0016	26.47	11.85	0.00002	I55	tmao	h	-10.40	2.7	-0.01	0.00	0.00019
I55	tmao	n	-12.29	3.4	-0.0494	0.0030	45.62	6.63	0.00004	I55	tmao	n	-50.04	11.4	-0.02	0.01	0.00330
I76	bet	c	11.91	47.3	-0.0045	0.1531	1000.00	27046.18	0.00005	I76	bet	c	8.68	1.4	0.00	0.00	0.00005
I76	bet	h	1.17	1.2	-0.0019	0.0007	1.00	7.64	0.00002	I76	bet	h	0.58	1.0	-0.00	0.00	0.00003
I76	bet	n	-4.71	33.0	0.0244	0.1068	1000.00	3457.53	0.00003	I76	bet	n	8.30	1.4	0.00	0.00	0.00005
I76	ect	c	44.73	6.1	-0.0049	0.0038	3.05	20.57	0.00043	I76	ect	c	42.86	4.4	-0.00	0.00	0.00048
I76	ect	h	2.21	2.2	0.0048	0.0022	68.73	64.67	0.00001	I76	ect	h	6.35	1.3	0.00	0.00	0.00005
I76	ect	n	-50.57	5.4	0.0117	0.0038	11.85	15.58	0.00024	I76	ect	n	-44.26	4.4	0.01	0.00	0.00048
I76	tmao	c	22.02	5.2	0.0575	0.0043	32.47	6.65	0.00013	I76	tmao	c	62.97	14.1	0.02	0.01	0.00506
I76	tmao	h	3.82	2.8	-0.0312	0.0020	16.12	3.77	0.00006	I76	tmao	h	-14.65	8.2	-0.02	0.00	0.00172
I76	tmao	n	-84.18	11.9	-0.3008	0.0099	35.56	3.11	0.00064	I76	tmao	n	-301.11	70.7	-0.13	0.03	0.12669
I88	bet	c	-5.60	2.3	0.0023	0.0014	1.00	12.89	0.00007	I88	bet	c	-4.89	1.8	0.00	0.00	0.00008
I88	bet	h	0.47	1.0	-0.0027	0.0006	5.98	7.91	0.00001	I88	bet	h	-0.75	1.0	-0.00	0.00	0.00002
I88	bet	n	35.01	3.9	0.0022	0.0024	2.30	26.51	0.00018	I88	bet	n	35.79	2.7	0.00	0.00	0.00019
I88	ect	c	2.30	8.8	-0.0055	0.0065	17.83	72.18	0.00053	I88	ect	c	-1.12	4.8	-0.00	0.00	0.00058
I88	ect	h	5.16	1.8	0.0016	0.0013	18.31	49.84	0.00002	I88	ect	h	6.22	1.0	0.00	0.00	0.00003
I88	ect	n	3.77	217.5	0.0798	0.7045	1000.00	6984.30	0.00115	I88	ect	n	47.98	7.4	0.00	0.00	0.00140
I88	tmao	c	-2.99	9.5	-0.0543	0.0087	56.80	20.49	0.00029	I88	tmao	c	-44.74	11.4	-0.02	0.01	0.00329
I88	tmao	h	6.67	1.7	0.0454	0.0017	73.16	5.62	0.00001	I88	tmao	h	43.69	9.2	0.01	0.00	0.00213
I88	tmao	n	42.92	5.8	0.0848	0.0046	26.71	4.24	0.00018	I88	tmao	n	100.29	21.0	0.04	0.01	0.01117
I96	bet	c	-1.41	2.3	-0.0037	0.0014	1.26	8.23	0.00007	I96	bet	c	-2.62	2.0	-0.00	0.00	0.00010
I96	bet	h	-8.10	1.1	-0.0009	0.0007	1.00	15.49	0.00002	I96	bet	h	-8.38	0.8	-0.00	0.00	0.00002
I96	bet	n	-38.66	5.9	0.0013	0.0061	86.17	809.36	0.00008	I96	bet	n	-37.34	1.8	0.00	0.00	0.00008
I96	ect	c	18.09	27.9	0.0048	0.0670	618.39	8008.10	0.00007	I96	ect	c	21.44	1.6	0.00	0.00	0.00007
I96	ect	h	-5.51	23.0	0.0207	0.0745	998.61	2846.47	0.00001	I96	ect	h	5.75	1.1	0.00	0.00	0.00003
I96	ect	n	-54.73	130.0	0.0064	0.4211	1000.00	52461.35	0.00041	I96	ect	n	-49.13	3.8	-0.00	0.00	0.00037
I96	tmao	c	29.72	13.0	0.0200	0.0127	70.54	92.82	0.00045	I96	tmao	c	46.63	6.2	0.01	0.00	0.00096
I96	tmao	h	-26.17	3.4	0.0041	0.0033	65.79	113.33	0.00003	I96	tmao	h	-22.67	1.5	0.00	0.00	0.00006
I96	tmao	n	-76.08	5.4	-0.0504	0.0048	49.48	11.18	0.00010	I96	tmao	n	-114.59	11.1	-0.02	0.01	0.00313
K108	bet	c	-29.12	3.5	0.0030	0.0028	27.56	76.53	0.00007	K108	bet	c	-27.00	1.8	0.00	0.00	0.00008
K108	bet	h	28.34	1.5	-0.0017	0.0009	1.00	11.55	0.00003	K108	bet	h	27.82	1.2	-0.00	0.00	0.00004
K108	bet	n	118.53	4.4	-0.0091	0.0033	20.89	24.62	0.00012	K108	bet	n	112.65	3.2	-0.00	0.00	0.00026
K108	ect	c	-421518.29	441868.1	345.6433	1000.9553	556.04	1554.04	21337.10946	K108	ect	c	-187417.64	36877.4	16.65	16.89	34499.01926
K108	ect	h	39.36	24.6	-0.0043	0.0797	1000.00	14603.76	0.00001	K108	ect	h	37.01	0.8	-0.00	0.00	0.00002
K108	ect	n	66.18	44.4	0.0098	0.1243	795.13	8564.50	0.00009	K108	ect	n	73.00	1.9	-0.00	0.00	0.00009
K108	tmao	c	-31.09	3.6	-0.0058	0.0022	1.00	7.89	0.00016	K108	tmao	c	-32.86	3.1	-0.00	0.00	0.00024
K108	tmao	h	16.18	1.3	-0.0012	0.0008	1.00	13.77	0.00002	K108	tmao	h	15.83	1.0	-0.00	0.00	0.00002
K108	tmao	n	113.96	11.1	-0.1098	0.0095	39.76	8.71	0.00051	K108	tmao	n	33.68	25.0	-0.04	0.01	0.01589
K19	bet	c	19.79	4.2	-0.0011	0.0039	57.78	469.92	0.00006	K19	bet	c	18.86	1.5	-0.00	0.00	0.00006
K19	bet	h	-13.64	1.0	-0.0023	0.0006	6.33	9.78	0.00001	K19	bet	h	-14.69	0.9	-0.00	0.00	0.00002
K19	bet	n	-161.06	167.8	-0.0307	0.5433	1000.00	14012.58	0.00068	K19	bet	n	-178.75	5.3	0.00	0.00	0.00072
K19	ect	c	42.37	3.1	-0.0035	0.0026	35.98	70.15	0.00004	K19	ect	c	39.74	1.6	-0.00	0.00	0.00006
K19	ect	h	-15.68	35.2	0.0131	0.1140	1000.00	6885.02	0.00003	K19	ect	h	-7.99	1.2	-0.00	0.00	0.00004
K19	ect	n	-193.51	17.4	0.1084	0.0322	381.26	125.99	0.00008	K19	ect	n	-113.16	9.2	0.01	0.00	0.00217

K19	tmao	c	50.61	8.0	0.0016	0.0079	72.96	741.06	0.00016	K19	tmao	c	51.79	2.6	0.00	0.00	0.00017
K19	tmao	h	-5.80	43.2	0.0044	0.1398	1000.00	25184.62	0.00005	K19	tmao	h	-2.26	1.2	-0.00	0.00	0.00004
K19	tmao	n	-149.63	74.0	0.1312	0.2070	794.46	1068.00	0.00025	K19	tmao	n	-72.18	6.9	0.00	0.00	0.00120
K27	bet	c	28.89	2.3	-0.0007	0.0014	2.46	48.81	0.00006	K27	bet	c	28.62	1.6	-0.00	0.00	0.00006
K27	bet	h	22.04	1.6	-0.0011	0.0010	1.34	20.45	0.00003	K27	bet	h	21.69	1.2	-0.00	0.00	0.00003
K27	bet	n	22.57	44.1	-0.0280	0.1427	1000.00	4035.55	0.00005	K27	bet	n	7.22	1.7	-0.00	0.00	0.00008
K27	ect	c	37.04	18.4	0.0085	0.0258	209.18	892.04	0.00024	K27	ect	c	44.94	3.4	0.00	0.00	0.00029
K27	ect	h	45.09	33.8	0.0054	0.1095	1000.00	16082.23	0.00003	K27	ect	h	48.67	1.0	-0.00	0.00	0.00003
K27	ect	n	12.03	99.9	0.1039	0.3234	999.41	2461.27	0.00024	K27	ect	n	68.09	5.1	0.00	0.00	0.00065
K27	tmao	c	55.50	101.8	-0.0643	0.3296	1000.00	4057.43	0.00025	K27	tmao	c	23.31	3.5	-0.00	0.00	0.00031
K27	tmao	h	45.45	4.0	0.0752	0.0040	78.41	8.30	0.00004	K27	tmao	h	107.10	14.8	0.02	0.01	0.00559
K27	tmao	n	64.24	9.8	0.2436	0.0094	65.55	5.40	0.00027	K27	tmao	n	259.18	50.0	0.08	0.02	0.06341
K39	bet	c	1.62	16.2	0.0254	0.0346	500.69	685.43	0.00004	K39	bet	c	19.03	2.1	0.00	0.00	0.00011
K39	bet	h	-0.79	0.9	-0.0018	0.0006	1.58	7.10	0.00001	K39	bet	h	-1.39	0.8	-0.00	0.00	0.00002
K39	bet	n	-48.77	87.3	-0.0656	0.2825	997.77	3401.91	0.00019	K39	bet	n	-82.29	3.3	-0.00	0.00	0.00028
K39	ect	c	66.72	7.1	-0.0058	0.0047	6.52	28.73	0.00050	K39	ect	c	63.98	4.8	-0.00	0.00	0.00058
K39	ect	h	-10.39	14.5	0.0405	0.0470	1000.00	919.02	0.00001	K39	ect	h	10.61	1.4	0.00	0.00	0.00005
K39	ect	n	-160.80	7.6	-0.0119	0.0059	23.64	36.11	0.00034	K39	ect	n	-169.07	5.0	-0.01	0.00	0.00062
K39	tmao	c	19.99	10.4	0.0916	0.0112	96.82	21.87	0.00021	K39	tmao	c	97.13	17.5	0.02	0.01	0.00781
K39	tmao	h	5.00	5.5	0.0634	0.0061	113.42	19.23	0.00005	K39	tmao	h	58.88	11.4	0.01	0.01	0.00332
K39	tmao	n	-168.74	9.7	-0.1073	0.0093	68.07	12.45	0.00025	K39	tmao	n	-256.24	22.8	-0.03	0.01	0.01323
K49	bet	c	-25.90	28.8	0.0279	0.0932	998.20	2636.23	0.00002	K49	bet	c	-11.40	1.3	0.00	0.00	0.00004
K49	ect	c	44.50	222.9	-0.1021	0.7219	1000.00	5592.17	0.00120	K49	ect	c	-12.34	8.0	0.00	0.00	0.00161
K49	ect	h	10.94	2.6	0.0017	0.0018	7.90	40.65	0.00006	K49	ect	h	11.79	1.7	0.00	0.00	0.00007
K49	ect	n	-18.94	12.7	0.0163	0.0188	233.26	360.57	0.00010	K49	ect	n	-5.60	2.8	0.00	0.00	0.00020
K49	tmao	c	1.37	7.3	-0.0529	0.0076	86.30	23.79	0.00012	K49	tmao	c	-42.41	10.3	-0.01	0.00	0.00271
K49	tmao	h	8.29	1.9	0.0151	0.0019	76.69	19.65	0.00001	K49	tmao	h	20.80	3.2	0.00	0.00	0.00025
K49	tmao	n	-8.97	2.1	-0.0056	0.0014	10.68	11.70	0.00004	K49	tmao	n	-11.96	2.0	-0.00	0.00	0.00010
K62	bet	c	-49.92	73.9	-0.0037	0.2394	1000.00	51885.12	0.00013	K62	bet	c	-52.91	2.2	0.00	0.00	0.00013
K62	bet	h	8.42	1.0	-0.0016	0.0006	1.71	8.57	0.00001	K62	bet	h	7.87	0.9	-0.00	0.00	0.00002
K62	bet	n	-68.56	117.0	-0.1189	0.3790	1000.00	2521.32	0.00033	K62	bet	n	-130.26	5.3	-0.00	0.00	0.00072
K62	ect	c	-53.86	6.9	-0.0015	0.0063	56.59	529.64	0.00015	K62	ect	c	-55.45	2.5	-0.00	0.00	0.00016
K62	ect	h	17.02	5.2	0.0075	0.0081	261.27	365.42	0.00001	K62	ect	h	23.24	1.2	0.00	0.00	0.00004
K62	ect	n	-108.97	6.0	-0.0058	0.0036	1.00	13.38	0.00046	K62	ect	n	-110.72	4.6	-0.00	0.00	0.00054
K62	tmao	c	-47.59	6.7	0.0061	0.0050	17.25	48.62	0.00031	K62	tmao	c	-43.69	3.9	0.00	0.00	0.00039
K62	tmao	h	8.37	1.4	-0.0034	0.0009	1.00	5.39	0.00003	K62	tmao	h	7.34	1.4	-0.00	0.00	0.00005
K62	tmao	n	-90.10	125.9	0.1023	0.4078	1000.00	3154.01	0.00038	K62	tmao	n	-32.67	5.6	-0.00	0.00	0.00079
K66	bet	c	7.85	57.4	0.0197	0.1663	841.81	5954.77	0.00013	K66	bet	c	19.13	2.4	0.00	0.00	0.00015
K66	bet	h	11.26	1.3	-0.0019	0.0009	6.24	15.95	0.00002	K66	bet	h	10.40	1.0	-0.00	0.00	0.00002
K66	bet	n	-66.09	79.1	-0.0841	0.2560	1000.00	2408.29	0.00015	K66	bet	n	-111.53	4.0	-0.00	0.00	0.00041
K66	ect	c	53.10	15.6	-0.0110	0.0159	82.69	234.81	0.00056	K66	ect	c	43.74	5.2	-0.00	0.00	0.00069
K66	ect	h	13.33	8.2	0.0119	0.0181	529.83	793.88	0.00001	K66	ect	h	21.32	0.9	0.00	0.00	0.00002
K66	ect	n	-185.16	86.3	0.1832	0.2619	904.79	1056.28	0.00024	K66	ect	n	-84.85	7.6	0.01	0.00	0.00146
K66	tmao	c	16.26	100.9	-0.0070	0.2719	748.56	25367.77	0.00054	K66	tmao	c	13.79	4.5	-0.00	0.00	0.00050
K66	tmao	h	16.87	27.4	0.0169	0.0580	490.57	1704.37	0.00011	K66	tmao	h	30.10	2.5	0.00	0.00	0.00016
K66	tmao	n	-97.99	85.7	0.1058	0.2775	999.86	2074.40	0.00018	K66	tmao	n	-41.05	4.8	0.00	0.00	0.00059
K98	bet	c	0.00	0.0	-0.0000	0.0000	500.50	0.00	0.00000	K98	bet	c	0.00	0.0	0.00	0.00	0.00000
K98	bet	h	4.49	1.0	-0.0008	0.0006	1.13	17.36	0.00001	K98	bet	h	4.24	0.8	-0.00	0.00	0.00001
K98	bet	n	46.85	51.5	-0.0640	0.1668	999.16	2060.05	0.00006	K98	bet	n	13.71	2.7	-0.00	0.00	0.00018
K98	ect	c	44.54	191.5	-0.0779	0.6201	1000.00	6302.44	0.00089	K98	ect	c	1.32	6.7	-0.00	0.00	0.00113
K98	ect	h	-5.93	25.7	0.0165	0.0831	999.48	3986.77	0.00002	K98	ect	h	2.34	0.9	0.00	0.00	0.00002
K98	ect	n	-14.69	13.8	-0.0044	0.0147	96.19	594.31	0.00037	K98	ect	n	-18.76	4.0	-0.00	0.00	0.00040
K98	tmao	c	-24.06	102.3	0.0320	0.3315	1000.00	8189.17	0.00025	K98	tmao	c	-7.01	3.4	0.00	0.00	0.00029
K98	tmao	h	11.05	1.0	-0.0037	0.0006	1.00	3.62	0.00001	K98	tmao	h	9.92	1.3	-0.00	0.00	0.00005
K98	tmao	n	66.42	9.2	-0.1459	0.0091	73.74	9.40	0.00022	K98	tmao	n	-51.40	28.6	-0.04	0.01	0.02080
L14	bet	c	-2.18	1.2	0.0006	0.0008	11.38	68.69	0.00001	L14	bet	c	-1.84	0.7	0.00	0.00	0.00001
L14	bet	h	0.64	1.0	-0.0019	0.0006	1.00	6.83	0.00001	L14	bet	h	0.08	0.9	-0.00	0.00	0.00002

L14	bet	n	-11.85	23.9	0.0700	0.0462	414.27	294.53	0.00012	L14	bet	n	39.37	6.1	0.01	0.00	0.00094
L14	ect	c	30.43	3.8	-0.0029	0.0037	67.93	183.12	0.00004	L14	ect	c	27.72	1.5	-0.00	0.00	0.00006
L14	ect	h	10.63	1.6	0.0019	0.0011	12.80	29.59	0.00002	L14	ect	h	11.74	1.0	0.00	0.00	0.00003
L14	ect	n	-52.43	24.5	0.0023	0.0285	126.80	2645.63	0.00086	L14	ect	n	-50.00	5.8	0.00	0.00	0.00087
L14	tmao	c	27.81	50.2	-0.0047	0.1626	1000.00	27248.58	0.00006	L14	tmao	c	25.08	1.6	0.00	0.00	0.00006
L14	tmao	h	0.93	7.3	0.0289	0.0091	153.70	76.25	0.00006	L14	tmao	h	25.75	4.9	0.00	0.00	0.00061
L14	tmao	n	-6.79	5.7	0.0375	0.0055	66.06	20.39	0.00009	L14	tmao	n	24.05	8.4	0.01	0.00	0.00180
L20	bet	h	21.97	1.4	-0.0019	0.0009	1.00	9.44	0.00002	L20	bet	h	21.37	1.2	-0.00	0.00	0.00003
L20	bet	n	10.18	64.6	0.0181	0.2091	1000.00	9142.60	0.00010	L20	bet	n	20.89	2.1	-0.00	0.00	0.00011
L20	ect	h	12.22	13.2	0.0214	0.0348	719.32	1033.04	0.00001	L20	ect	h	25.20	1.2	0.00	0.00	0.00004
L20	ect	n	78.33	4.3	-0.0050	0.0032	19.37	40.85	0.00012	L20	ect	n	75.05	2.6	-0.00	0.00	0.00017
L20	tmao	h	16.73	61.7	0.0057	0.1999	1000.00	27704.93	0.00009	L20	tmao	h	21.39	1.7	-0.00	0.00	0.00007
L20	tmao	n	103.00	2.9	-0.0086	0.0019	6.46	7.84	0.00008	L20	tmao	n	99.09	3.0	-0.01	0.00	0.00023
L33	bet	c	-40.72	27.7	-0.0053	0.0798	835.40	10593.10	0.00003	L33	bet	c	-43.96	1.1	0.00	0.00	0.00003
L33	bet	h	0.42	0.5	-0.0023	0.0003	1.00	2.56	0.00000	L33	bet	h	-0.30	0.8	-0.00	0.00	0.00002
L33	bet	n	-68.05	137.7	-0.1584	0.4457	998.69	2224.44	0.00046	L33	bet	n	-149.75	6.7	-0.00	0.00	0.00112
L33	ect	c	-48.58	1.5	0.0076	0.0015	73.48	29.06	0.00001	L33	ect	c	-42.22	1.7	0.00	0.00	0.00007
L33	ect	h	11.82	2.5	0.0054	0.0027	104.72	95.84	0.00001	L33	ect	h	16.53	1.3	0.00	0.00	0.00004
L33	ect	n	-141.81	6.2	0.0165	0.0067	99.59	74.53	0.00007	L33	ect	n	-128.14	3.4	0.00	0.00	0.00029
L33	tmao	c	-17.55	13.1	0.0321	0.0175	182.97	147.05	0.00015	L33	tmao	c	9.55	5.3	0.00	0.00	0.00070
L33	tmao	h	13.31	6.5	0.0306	0.0077	134.91	55.71	0.00006	L33	tmao	h	39.56	5.4	0.01	0.00	0.00074
L33	tmao	n	-122.94	11.1	0.0935	0.0129	124.83	29.18	0.00018	L33	tmao	n	-43.74	16.1	0.02	0.01	0.00656
L42	bet	c	30.33	13.9	0.0034	0.0241	333.55	2765.89	0.00006	L42	bet	c	33.59	1.6	-0.00	0.00	0.00007
L42	bet	h	56.98	1.8	-0.0017	0.0011	1.00	14.29	0.00004	L42	bet	h	56.47	1.4	-0.00	0.00	0.00005
L42	bet	n	113.68	6.9	-0.0039	0.0056	30.55	122.93	0.00024	L42	bet	n	110.84	3.2	-0.00	0.00	0.00026
L42	ect	c	31.95	72.0	0.1105	0.2329	998.86	1667.39	0.00013	L42	ect	c	90.10	4.6	0.00	0.00	0.00053
L42	ect	h	99.97	13.2	0.0807	0.0239	362.84	121.81	0.00005	L42	ect	h	160.72	7.3	0.01	0.00	0.00135
L42	ect	n	169.00	96.7	0.2486	0.2558	725.53	656.01	0.00053	L42	ect	n	319.67	13.0	0.01	0.01	0.00428
L42	tmao	c	63.93	14.6	0.2973	0.0152	88.46	8.64	0.00045	L42	tmao	c	311.63	57.3	0.08	0.03	0.08339
L42	tmao	h	119.85	12.2	0.6574	0.0124	80.10	2.99	0.00035	L42	tmao	h	659.41	128.2	0.19	0.06	0.41686
L42	tmao	n	260.89	22.3	1.2933	0.0225	79.53	2.75	0.00118	L42	tmao	n	1320.95	252.3	0.37	0.12	1.61420
L63	bet	h	-6.27	1.0	-0.0022	0.0006	1.00	5.69	0.00001	L63	bet	h	-6.95	1.0	-0.00	0.00	0.00002
L63	bet	n	-104.62	61.9	-0.0550	0.2003	998.94	2877.74	0.00009	L63	bet	n	-133.36	2.7	-0.00	0.00	0.00019
L63	ect	h	-9.64	25.6	0.0230	0.0830	999.65	2851.32	0.00002	L63	ect	h	2.20	1.1	0.00	0.00	0.00003
L63	ect	n	-112.68	149.6	0.0602	0.4844	1000.00	6369.21	0.00054	L63	ect	n	-78.83	5.2	-0.00	0.00	0.00068
L63	tmao	h	5.57	2.9	0.0181	0.0034	123.83	39.11	0.00001	L63	tmao	h	21.09	3.3	0.00	0.00	0.00027
L63	tmao	n	-104.92	3.6	-0.0053	0.0022	1.00	8.67	0.00016	L63	tmao	n	-106.56	3.0	-0.00	0.00	0.00023
L89	bet	c	-6.44	1.9	-0.0027	0.0014	17.91	33.23	0.00003	L89	bet	c	-8.12	1.2	-0.00	0.00	0.00004
L89	bet	h	-4.25	0.9	-0.0021	0.0005	4.40	7.79	0.00001	L89	bet	h	-5.13	0.8	-0.00	0.00	0.00002
L89	bet	n	23.16	3.2	-0.0043	0.0042	177.32	260.27	0.00001	L89	bet	n	19.40	0.9	-0.00	0.00	0.00002
L89	ect	c	-6.11	118.3	0.0405	0.3827	998.60	7467.77	0.00034	L89	ect	c	15.38	4.0	0.00	0.00	0.00040
L89	ect	h	-1.88	40.0	0.0063	0.1294	1000.00	16311.34	0.00004	L89	ect	h	1.98	1.2	-0.00	0.00	0.00004
L89	ect	n	-8.76	9.7	-0.0128	0.0069	13.58	28.08	0.00072	L89	ect	n	-16.33	6.5	-0.01	0.00	0.00107
L89	tmao	c	-136.93	547.5	0.2759	1.4509	999.20	3438.89	0.00014	L89	tmao	c	52.61	11.7	0.00	0.00	0.00069
L89	tmao	h	3.82	5.0	0.0010	0.0050	77.43	780.92	0.00006	L89	tmao	h	5.19	1.6	-0.00	0.00	0.00006
L89	tmao	n	4.64	4.4	-0.0785	0.0044	78.13	8.77	0.00005	L89	tmao	n	-59.67	15.5	-0.02	0.01	0.00609
L95	bet	c	-18.42	4.1	0.0065	0.0047	119.65	147.45	0.00003	L95	bet	c	-13.12	1.4	0.00	0.00	0.00005
L95	bet	h	-4.94	0.6	-0.0006	0.0004	1.00	12.54	0.00000	L95	bet	h	-5.12	0.4	-0.00	0.00	0.00001
L95	bet	n	-89.74	89.3	-0.0419	0.2894	1000.00	5463.46	0.00019	L95	bet	n	-111.27	3.0	-0.00	0.00	0.00024
L95	ect	c	86.73	270.2	-0.1136	0.8752	1000.00	6098.18	0.00177	L95	ect	c	26.11	9.4	-0.00	0.00	0.00222
L95	ect	h	-1.04	6.2	0.0074	0.0095	254.55	421.69	0.00002	L95	ect	h	4.84	1.2	0.00	0.00	0.00004
L95	ect	n	-168.74	126.5	0.1035	0.4097	1000.00	3131.38	0.00039	L95	ect	n	-115.68	5.0	0.00	0.00	0.00064
L95	tmao	c	-29.69	36.9	0.0119	0.0276	114.74	354.24	0.00018	L95	tmao	c	-9.42	7.7	0.00	0.00	0.00030
L95	tmao	h	-3.65	33.8	0.0249	0.1095	999.53	3474.84	0.00003	L95	tmao	h	9.79	1.4	0.00	0.00	0.00005
L95	tmao	n	-183.61	30.4	0.2292	0.0985	1000.00	340.03	0.00002	L95	tmao	n	-64.77	7.6	0.01	0.00	0.00145
N23	bet	c	-51.70	2.4	0.0020	0.0014	1.00	15.23	0.00007	N23	bet	c	-51.07	1.8	0.00	0.00	0.00008
N23	bet	h	-20.76	1.2	-0.0012	0.0007	1.87	14.27	0.00002	N23	bet	h	-21.17	0.9	-0.00	0.00	0.00002

N23	bet	n	-49.32	2.2	-0.0089	0.0021	59.13	30.37	0.00002	N23	bet	n	-56.50	2.1	-0.00	0.00	0.00012
N23	ect	c	-22.12	60.5	0.0084	0.1958	1000.00	18359.48	0.00009	N23	ect	c	-17.37	1.9	-0.00	0.00	0.00009
N23	ect	h	-36.49	20.8	0.0335	0.0672	1000.00	1586.52	0.00001	N23	ect	h	-18.63	1.4	0.00	0.00	0.00005
N23	ect	n	5.51	8.8	-0.0090	0.0060	9.31	28.13	0.00069	N23	ect	n	0.71	5.9	-0.01	0.00	0.00088
N23	tmao	c	-1.08	8.3	0.0386	0.0080	67.00	29.40	0.00019	N23	tmao	c	30.68	8.8	0.01	0.00	0.00198
N23	tmao	h	7.03	2.1	0.0350	0.0021	70.68	8.62	0.00001	N23	tmao	h	35.37	7.1	0.01	0.00	0.00128
N23	tmao	n	30.27	6.7	0.1043	0.0059	44.47	6.09	0.00017	N23	tmao	n	108.61	23.4	0.04	0.01	0.01385
N41	bet	c	18.46	1.0	0.0018	0.0007	9.16	16.48	0.00001	N41	bet	c	19.37	0.8	0.00	0.00	0.00002
N41	bet	h	-6.59	1.1	-0.0034	0.0008	9.48	9.73	0.00001	N41	bet	h	-8.35	1.2	-0.00	0.00	0.00003
N41	bet	n	-74.95	90.5	-0.0130	0.2931	1000.00	17804.77	0.00020	N41	bet	n	-83.48	2.7	0.00	0.00	0.00019
N41	ect	c	46.52	6.3	-0.0040	0.0042	7.29	39.48	0.00039	N41	ect	c	44.54	4.1	-0.00	0.00	0.00042
N41	ect	h	-29.69	23.1	0.0435	0.0748	1000.00	1359.60	0.00001	N41	ect	h	-7.05	1.6	0.00	0.00	0.00007
N41	ect	n	-186.29	62.3	0.1772	0.2018	999.54	901.02	0.00009	N41	ect	n	-94.14	6.3	0.00	0.00	0.00102
N41	tmao	c	37.49	8.2	0.0592	0.0074	51.11	14.78	0.00023	N41	tmao	c	83.53	13.5	0.02	0.01	0.00464
N41	tmao	h	-2.07	4.5	0.0509	0.0057	159.13	27.77	0.00002	N41	tmao	h	41.05	7.9	0.01	0.00	0.00157
N41	tmao	n	-115.60	8.0	0.1479	0.0084	90.84	9.76	0.00013	N41	tmao	n	7.07	27.8	0.04	0.01	0.01958
N58	bet	c	-29.14	1.3	0.0008	0.0008	2.86	25.45	0.00002	N58	bet	c	-28.83	0.9	0.00	0.00	0.00002
N58	bet	h	12.13	1.2	-0.0020	0.0008	1.72	8.60	0.00002	N58	bet	h	11.45	1.1	-0.00	0.00	0.00003
N58	bet	n	-9.42	49.5	0.0056	0.1604	1000.00	22564.13	0.00006	N58	bet	n	-5.72	1.5	-0.00	0.00	0.00006
N58	ect	c	-11.60	5.9	-0.0034	0.0040	8.58	46.92	0.00032	N58	ect	c	-13.32	3.7	-0.00	0.00	0.00034
N58	ect	h	2.64	39.5	0.0217	0.1281	1000.00	4678.67	0.00004	N58	ect	h	13.98	1.4	0.00	0.00	0.00005
N58	ect	n	79.78	6.8	-0.0067	0.0046	8.76	28.15	0.00042	N58	ect	n	76.30	4.5	-0.00	0.00	0.00052
N58	tmao	c	-26.95	38.5	0.0544	0.0783	456.88	682.47	0.00025	N58	tmao	c	12.82	5.5	0.00	0.00	0.00076
N58	tmao	h	38.53	3.4	-0.0145	0.0021	3.40	4.05	0.00013	N58	tmao	h	32.91	4.9	-0.01	0.00	0.00060
N58	tmao	n	-79.73	227.7	1.1013	0.7375	999.94	529.84	0.00126	N58	tmao	n	494.95	38.1	0.03	0.02	0.03677
N5	bet	c	-2.43	2.2	0.0011	0.0015	10.13	60.36	0.00004	N5	bet	c	-1.84	1.3	0.00	0.00	0.00004
N5	bet	h	39.82	1.5	-0.0028	0.0010	3.42	9.52	0.00003	N5	bet	h	38.73	1.3	-0.00	0.00	0.00004
N5	bet	n	61.72	3.8	-0.0024	0.0029	20.22	79.43	0.00009	N5	bet	n	60.07	2.1	-0.00	0.00	0.00011
N5	ect	c	12.34	6.3	-0.0042	0.0053	34.93	116.42	0.00018	N5	ect	c	9.08	2.9	-0.00	0.00	0.00022
N5	ect	h	53.28	22.5	-0.0135	0.0730	1000.00	4262.74	0.00001	N5	ect	h	46.24	0.8	-0.00	0.00	0.00002
N5	ect	n	60.36	4.5	-0.0023	0.0030	6.86	47.94	0.00020	N5	ect	n	59.27	2.9	-0.00	0.00	0.00021
N5	tmao	c	21.77	88.3	-0.0481	0.2861	1000.00	4706.37	0.00019	N5	tmao	c	-3.99	3.3	-0.00	0.00	0.00027
N5	tmao	h	21.79	35.6	-0.0249	0.1152	999.09	3665.65	0.00003	N5	tmao	h	9.42	1.2	-0.00	0.00	0.00004
N5	tmao	n	54.91	4.2	-0.0694	0.0036	43.06	5.56	0.00007	N5	tmao	n	2.83	15.8	-0.03	0.01	0.00636
N77	bet	c	-38.91	34.6	0.0477	0.1120	1000.00	1856.37	0.00003	N77	bet	c	-13.23	2.1	0.00	0.00	0.00011
N77	bet	h	-2.27	0.6	-0.0014	0.0004	1.00	5.76	0.00000	N77	bet	h	-2.70	0.6	-0.00	0.00	0.00001
N77	bet	n	-15.25	36.2	0.0081	0.1172	1000.00	11429.57	0.00003	N77	bet	n	-10.97	1.2	0.00	0.00	0.00003
N77	ect	c	-185.96	243.3	0.3193	0.7879	1000.00	1952.41	0.00143	N77	ect	c	-15.86	14.0	0.01	0.01	0.00497
N77	ect	h	6.06	1.7	0.0016	0.0017	75.95	161.25	0.00001	N77	ect	h	7.37	0.6	0.00	0.00	0.00001
N77	ect	n	29.68	17.6	-0.0418	0.0201	120.62	99.46	0.00047	N77	ect	n	-6.58	8.8	-0.01	0.00	0.00196
N77	tmao	c	16.62	38.7	0.0190	0.0759	426.45	1815.50	0.00029	N77	tmao	c	32.39	3.9	0.00	0.00	0.00039
N77	tmao	h	8.43	3.6	0.0140	0.0032	44.14	24.48	0.00005	N77	tmao	h	18.97	3.5	0.01	0.00	0.00030
N77	tmao	n	11.28	9.0	0.1768	0.0066	17.37	2.26	0.00055	N77	tmao	n	118.48	46.1	0.09	0.02	0.05400
N84	bet	c	33.37	2.7	-0.0008	0.0025	55.50	417.95	0.00002	N84	bet	c	32.70	1.0	-0.00	0.00	0.00003
N84	bet	h	-13.87	1.0	-0.0035	0.0007	14.18	10.96	0.00001	N84	bet	h	-15.90	1.1	-0.00	0.00	0.00003
N84	bet	n	-235.09	388.9	-0.5441	1.2590	999.49	1830.30	0.00367	N84	bet	n	-520.29	22.7	-0.01	0.01	0.01311
N84	ect	c	24.45	4.7	-0.0035	0.0029	2.95	21.58	0.00025	N84	ect	c	23.10	3.3	-0.00	0.00	0.00028
N84	ect	h	-1.36	35.2	0.0037	0.1138	999.61	24654.82	0.00003	N84	ect	h	1.16	1.1	-0.00	0.00	0.00003
N84	ect	n	-487.94	16.3	-0.0648	0.0164	78.12	39.52	0.00064	N84	ect	n	-539.68	12.8	-0.02	0.01	0.00414
N84	tmao	c	3.92	18.7	-0.1197	0.0197	92.16	28.64	0.00072	N84	tmao	c	-93.58	21.6	-0.03	0.01	0.01184
N84	tmao	h	-4.53	2.6	-0.0415	0.0024	58.89	7.67	0.00002	N84	tmao	h	-37.07	8.7	-0.01	0.00	0.00190
N84	tmao	n	-338.79	11.4	-0.5426	0.0103	52.07	2.29	0.00044	N84	tmao	n	-759.70	118.5	-0.19	0.05	0.35607
P21	bet	c	-110.56	3.6	0.0020	0.0022	1.00	22.96	0.00016	P21	bet	c	-109.96	2.6	0.00	0.00	0.00017
P21	ect	c	-53.74	8.4	-0.0087	0.0093	109.88	208.90	0.00012	P21	ect	c	-61.56	2.8	-0.00	0.00	0.00020
P21	tmao	c	-15.94	1.7	0.0353	0.0013	22.70	2.61	0.00002	P21	tmao	c	7.12	9.0	0.02	0.00	0.00206
P47	bet	c	-39.31	4.7	0.0014	0.0033	12.84	117.11	0.00017	P47	bet	c	-38.52	2.6	0.00	0.00	0.00018
P47	ect	c	-76.49	75.1	0.0656	0.2431	1000.00	2934.48	0.00014	P47	ect	c	-41.80	3.3	0.00	0.00	0.00028

P47	tmao	c	-47.08	6.8	0.0260	0.0061	51.80	28.30	0.00016	P47	tmao	c	-26.37	6.6	0.01	0.00	0.00110
P64	bet	c	-35.45	129.2	-0.0399	0.4184	1000.00	8298.72	0.00040	P64	bet	c	-58.14	4.3	0.00	0.00	0.00047
P64	ect	c	-34.03	2.3	0.0019	0.0014	3.66	20.99	0.00006	P64	ect	c	-33.25	1.6	0.00	0.00	0.00007
P64	tmao	c	8.42	3.3	-0.0189	0.0024	15.76	7.34	0.00008	P64	tmao	c	-2.75	5.3	-0.01	0.00	0.00071
Q104	bet	c	-32.07	2.2	0.0014	0.0013	1.00	20.12	0.00006	Q104	bet	c	-31.63	1.6	0.00	0.00	0.00007
Q104	bet	h	-2.18	9.2	0.0044	0.0297	999.80	5343.22	0.00000	Q104	bet	h	0.19	0.3	0.00	0.00	0.00000
Q104	bet	n	0.00	0.0	-0.0000	0.0000	500.50	0.00	0.00000	Q104	bet	n	0.00	0.0	0.00	0.00	0.00000
Q104	ect	c	-37.66	74.3	0.0448	0.2406	999.72	4250.91	0.00013	Q104	ect	c	-15.28	2.5	0.00	0.00	0.00016
Q104	ect	h	5.86	3.1	0.0033	0.0043	202.31	373.57	0.00001	Q104	ect	h	8.69	0.7	0.00	0.00	0.00001
Q104	ect	n	14.75	5.4	-0.0026	0.0035	5.79	45.18	0.00029	Q104	ect	n	13.55	3.5	-0.00	0.00	0.00031
Q104	tmao	c	-15.93	8.6	0.0663	0.0082	65.13	17.19	0.00021	Q104	tmao	c	37.34	14.1	0.02	0.01	0.00502
Q104	tmao	h	16.26	1.6	-0.0068	0.0011	9.93	7.23	0.00002	Q104	tmao	h	12.79	2.1	-0.00	0.00	0.00011
Q104	tmao	n	60.90	33.7	-0.1741	0.0710	488.08	201.49	0.00017	Q104	tmao	n	-60.47	12.5	-0.01	0.01	0.00396
Q15	bet	c	-39.79	5.9	0.0002	0.0067	117.64	7030.72	0.00005	Q15	bet	c	-39.08	1.4	-0.00	0.00	0.00005
Q15	bet	h	5.83	0.6	-0.0005	0.0003	2.61	18.32	0.00000	Q15	bet	h	5.65	0.4	-0.00	0.00	0.00000
Q15	bet	n	-68.60	89.9	-0.0204	0.2911	1000.00	11319.29	0.00020	Q15	bet	n	-80.90	2.8	0.00	0.00	0.00020
Q15	ect	c	15.38	6.6	-0.0073	0.0056	39.56	77.73	0.00018	Q15	ect	c	9.37	3.4	-0.00	0.00	0.00030
Q15	ect	h	24.84	1.2	-0.0009	0.0007	1.00	17.31	0.00002	Q15	ect	h	24.57	0.9	-0.00	0.00	0.00002
Q15	ect	n	-18.96	20.2	-0.0322	0.0185	54.19	70.83	0.00135	Q15	ect	n	-45.83	10.9	-0.01	0.01	0.00303
Q15	tmao	c	-24.79	91.0	0.0275	0.2373	709.01	5414.03	0.00050	Q15	tmao	c	-4.87	4.6	-0.00	0.00	0.00055
Q15	tmao	h	9.09	20.8	0.0079	0.0675	1000.00	6730.90	0.00001	Q15	tmao	h	13.57	0.7	-0.00	0.00	0.00001
Q15	tmao	n	-145.09	6.5	0.0155	0.0039	1.00	5.38	0.00053	Q15	tmao	n	-140.33	6.6	0.01	0.00	0.00111
Q2	bet	c	23.26	120.0	-0.0812	0.3888	1000.00	3787.21	0.00035	Q2	bet	c	-22.70	4.9	0.00	0.00	0.00060
Q2	ect	c	-197.52	79.2	0.1546	0.0669	37.41	41.99	0.02738	Q2	ect	c	-79.99	51.5	0.06	0.02	0.06733
Q2	tmao	c	33.91	5.3	-0.1572	0.0033	2.03	0.49	0.00034	Q2	tmao	c	19.33	8.8	-0.15	0.00	0.00185
Q31	bet	c	5.92	2.2	-0.0008	0.0018	37.31	223.75	0.00002	Q31	bet	c	5.18	0.9	-0.00	0.00	0.00002
Q31	bet	h	9.99	45.3	0.0032	0.1468	1000.00	36130.84	0.00005	Q31	bet	h	13.05	1.2	-0.00	0.00	0.00004
Q31	bet	n	24.00	5.6	0.0099	0.0062	108.94	121.18	0.00005	Q31	bet	n	32.12	2.2	0.00	0.00	0.00012
Q31	ect	c	34.92	104.6	0.0344	0.3387	1000.00	7788.60	0.00027	Q31	ect	c	53.55	3.5	0.00	0.00	0.00031
Q31	ect	h	4.82	19.6	0.0498	0.0634	1000.00	1007.60	0.00001	Q31	ect	h	30.93	1.8	0.00	0.00	0.00009
Q31	ect	n	22.90	150.1	0.0424	0.4862	1000.00	9080.38	0.00055	Q31	ect	n	47.51	4.9	-0.00	0.00	0.00061
Q31	tmao	c	38.32	15.7	0.1616	0.0175	109.66	21.03	0.00042	Q31	tmao	c	173.87	28.6	0.04	0.01	0.02075
Q31	tmao	h	12.46	4.0	0.0634	0.0048	135.98	16.77	0.00002	Q31	tmao	h	65.96	10.3	0.01	0.00	0.00268
Q31	tmao	n	-17.48	77.4	0.1201	0.1812	589.43	839.99	0.00057	Q31	tmao	n	64.53	9.4	0.00	0.00	0.00225
R110	bet	h	-8.76	0.9	-0.0020	0.0006	7.05	10.20	0.00001	R110	bet	h	-9.73	0.8	-0.00	0.00	0.00002
R110	bet	n	95.09	77.9	0.0239	0.2522	1000.00	8353.93	0.00015	R110	bet	n	108.10	2.6	0.00	0.00	0.00017
R110	ect	h	-7.13	26.4	0.0184	0.0853	1000.00	3678.84	0.00002	R110	ect	h	2.89	1.1	0.00	0.00	0.00003
R110	ect	n	60.91	56.4	0.0657	0.1125	440.23	793.13	0.00058	R110	ect	n	107.17	6.6	0.01	0.00	0.00111
R110	tmao	h	5.61	2.5	0.0234	0.0025	73.39	16.06	0.00002	R110	tmao	h	24.82	4.9	0.01	0.00	0.00060
R110	tmao	n	269.91	4.7	-0.0373	0.0032	7.49	3.21	0.00021	R110	tmao	n	252.02	10.9	-0.02	0.01	0.00304
R59	bet	c	-2.42	2.0	-0.0041	0.0013	3.37	8.60	0.00005	R59	bet	c	-4.01	1.8	-0.00	0.00	0.00008
R59	ect	c	118.79	110.2	0.0508	0.3568	1000.00	5553.62	0.00029	R59	ect	c	147.58	3.9	-0.00	0.00	0.00039
R59	ect	h	147.22	3.5	0.0097	0.0039	113.35	80.91	0.00002	R59	ect	h	155.09	1.7	0.00	0.00	0.00008
R59	ect	n	30.67	81.4	0.2751	0.1832	549.47	354.69	0.00074	R59	ect	n	211.14	16.3	0.02	0.01	0.00672
R59	tmao	c	-14.83	37.6	0.1328	0.0846	548.76	339.20	0.00016	R59	tmao	c	75.03	9.0	0.01	0.00	0.00207
R59	tmao	h	11.94	1.4	0.0052	0.0010	11.69	8.78	0.00002	R59	tmao	h	14.79	1.6	0.00	0.00	0.00007
R59	tmao	n	-40.89	14.3	-0.0591	0.0149	89.36	42.95	0.00043	R59	tmao	n	-91.43	12.8	-0.01	0.01	0.00414
R69	bet	c	-62.65	58.1	-0.0474	0.1880	999.54	3137.94	0.00008	R69	bet	c	-87.09	2.3	-0.00	0.00	0.00014
R69	bet	h	-14.17	0.8	-0.0013	0.0005	1.83	9.30	0.00001	R69	bet	h	-14.62	0.7	-0.00	0.00	0.00001
R69	bet	n	-17.52	2.1	-0.0037	0.0013	1.00	7.35	0.00006	R69	bet	n	-18.64	1.9	-0.00	0.00	0.00009
R69	ect	c	-90.04	1.0	-0.0039	0.0006	1.00	3.18	0.00001	R69	ect	c	-91.22	1.4	-0.00	0.00	0.00005
R69	ect	h	-22.87	31.0	0.0323	0.1003	999.71	2457.57	0.00002	R69	ect	h	-6.61	1.3	0.00	0.00	0.00004
R69	ect	n	-24.81	152.1	0.0749	0.4926	1000.00	5205.00	0.00056	R69	ect	n	16.63	5.5	0.00	0.00	0.00078
R69	tmao	c	-110.78	4.6	-0.0065	0.0030	6.31	16.14	0.00021	R69	tmao	c	-113.77	3.4	-0.00	0.00	0.00030
R69	tmao	h	-4.44	47.7	0.0089	0.1544	1000.00	13677.22	0.00006	R69	tmao	h	1.47	1.4	-0.00	0.00	0.00005
R69	tmao	n	25.09	5.5	0.0202	0.0049	49.80	28.52	0.00011	R69	tmao	n	40.30	4.6	0.01	0.00	0.00055
R72	bet	c	23.26	17.8	-0.0236	0.0297	306.21	466.56	0.00012	R72	bet	c	5.11	3.1	-0.00	0.00	0.00025

R72	bet	h	1.75	0.7	-0.0026	0.0005	7.06	6.83	0.00001	R72	bet	h	0.50	0.9	-0.00	0.00	0.00002
R72	bet	n	23.39	5.1	0.0006	0.0044	40.00	692.72	0.00011	R72	bet	n	23.97	2.1	0.00	0.00	0.00011
R72	ect	c	-24.25	108.5	0.0623	0.3515	1000.00	4464.86	0.00029	R72	ect	c	9.84	4.1	0.00	0.00	0.00044
R72	ect	h	1.15	34.9	0.0061	0.1130	1000.00	14743.81	0.00003	R72	ect	h	4.57	1.1	-0.00	0.00	0.00003
R72	ect	n	-11.20	70.2	0.0397	0.2274	1000.00	4537.28	0.00012	R72	ect	n	10.35	2.7	0.00	0.00	0.00018
R72	tmao	c	34.91	10.5	0.0202	0.0094	48.97	53.73	0.00040	R72	tmao	c	50.50	6.0	0.01	0.00	0.00092
R72	tmao	h	12.01	1.0	-0.0021	0.0006	1.00	6.23	0.00001	R72	tmao	h	11.38	0.9	-0.00	0.00	0.00002
R72	tmao	n	1.24	15.6	-0.0474	0.0174	111.93	72.27	0.00040	R72	tmao	n	-39.26	9.5	-0.01	0.00	0.00230
R83	bet	c	-113.18	287.7	-0.0776	0.9316	1000.00	9495.37	0.00200	R83	bet	c	-158.51	9.3	0.00	0.00	0.00220
R83	bet	h	116.39	202.4	0.1942	0.6554	1000.00	2670.64	0.00099	R83	bet	h	220.98	9.7	0.00	0.00	0.00238
R83	bet	n	25.36	70.1	-0.1059	0.2270	1000.00	1696.28	0.00012	R83	bet	n	-30.54	4.4	-0.00	0.00	0.00048
R83	ect	c	-9.66	410.7	-0.1235	1.3283	997.72	8497.79	0.00411	R83	ect	c	-74.76	13.6	-0.00	0.01	0.00467
R83	ect	h	227.47	16.7	0.1037	0.0262	269.49	86.53	0.00013	R83	ect	h	310.11	11.6	0.01	0.01	0.00340
R83	ect	n	217.01	33.6	0.3944	0.0658	423.81	75.49	0.00023	R83	ect	n	501.19	30.3	0.03	0.01	0.02334
R83	tmao	c	-28.55	10.0	0.1450	0.0097	68.01	9.57	0.00028	R83	tmao	c	87.93	29.5	0.04	0.01	0.02208
R83	tmao	h	142.76	11.6	0.5898	0.0118	81.53	3.22	0.00031	R83	tmao	h	627.71	114.4	0.16	0.05	0.33222
R83	tmao	n	446.39	35.6	2.0312	0.0355	76.45	2.70	0.00312	R83	tmao	n	2106.28	402.1	0.59	0.18	4.10085
R87	bet	c	8.86	32.1	-0.0118	0.1041	1000.00	6964.42	0.00003	R87	bet	c	2.04	1.1	0.00	0.00	0.00003
R87	bet	h	4.74	0.7	-0.0014	0.0004	1.00	6.64	0.00001	R87	bet	h	4.32	0.7	-0.00	0.00	0.00001
R87	bet	n	34.32	63.3	0.0406	0.2049	999.07	3988.45	0.00010	R87	bet	n	56.35	2.5	0.00	0.00	0.00016
R87	ect	c	-63.12	180.3	0.1472	0.5838	1000.00	3138.60	0.00079	R87	ect	c	15.55	7.8	0.00	0.00	0.00155
R87	ect	h	-2.59	30.1	0.0065	0.0974	1000.00	11905.14	0.00002	R87	ect	h	0.38	0.9	0.00	0.00	0.00002
R87	ect	n	51.22	9.9	-0.0186	0.0089	49.28	55.28	0.00035	R87	ect	n	36.85	5.6	-0.01	0.00	0.00079
R87	tmao	c	0.27	7.1	-0.0417	0.0052	15.23	6.86	0.00037	R87	tmao	c	-23.88	11.4	-0.02	0.01	0.00329
R87	tmao	h	4.03	1.5	-0.0044	0.0013	47.42	32.90	0.00001	R87	tmao	h	0.64	1.1	-0.00	0.00	0.00003
R87	tmao	n	53.78	8.2	0.1133	0.0062	20.40	3.63	0.00042	R87	tmao	n	125.55	29.2	0.06	0.01	0.02168
S28	bet	c	8.12	52.1	0.0099	0.1688	1000.00	13550.13	0.00007	S28	bet	c	13.19	1.6	0.00	0.00	0.00007
S28	bet	h	28.92	1.3	-0.0013	0.0008	5.52	20.60	0.00002	S28	bet	h	28.32	0.9	-0.00	0.00	0.00002
S28	bet	n	30.32	2.7	-0.0016	0.0016	1.00	21.76	0.00009	S28	bet	n	29.87	1.9	-0.00	0.00	0.00009
S28	ect	c	36.24	4.9	-0.0047	0.0030	2.64	16.64	0.00028	S28	ect	c	34.52	3.6	-0.00	0.00	0.00033
S28	ect	h	30.07	35.7	0.0311	0.1157	999.70	2940.09	0.00003	S28	ect	h	46.92	1.6	0.00	0.00	0.00007
S28	ect	n	-5.88	11.5	-0.0054	0.0111	67.55	293.11	0.00036	S28	ect	n	-10.59	4.0	-0.00	0.00	0.00041
S28	tmao	c	-16.22	14.4	0.1407	0.0210	228.65	46.13	0.00013	S28	tmao	c	99.50	17.8	0.02	0.01	0.00802
S28	tmao	h	22.83	2.8	0.0367	0.0029	90.08	13.62	0.00002	S28	tmao	h	53.50	7.1	0.01	0.00	0.00128
S28	tmao	n	-56.31	7.3	-0.0849	0.0061	35.11	6.68	0.00024	S28	tmao	n	-118.06	20.8	-0.04	0.01	0.01093
S38	bet	c	-23.08	64.0	-0.0656	0.2071	998.82	2495.58	0.00010	S38	bet	c	-57.40	3.0	-0.00	0.00	0.00023
S38	bet	h	44.10	61.1	0.0352	0.1978	1000.00	4443.37	0.00009	S38	bet	h	63.70	2.3	-0.00	0.00	0.00014
S38	bet	n	84.10	208.9	0.0499	0.6766	1000.00	10736.19	0.00106	S38	bet	n	113.01	6.7	-0.00	0.00	0.00114
S38	ect	c	-31.95	1.5	0.0023	0.0012	24.09	38.50	0.00001	S38	ect	c	-30.37	1.0	0.00	0.00	0.00002
S38	ect	h	74.14	41.3	-0.0124	0.1336	1000.00	8498.93	0.00004	S38	ect	h	67.90	1.3	-0.00	0.00	0.00004
S38	ect	n	97.42	81.3	-0.1017	0.2631	999.23	2045.22	0.00016	S38	ect	n	44.80	4.2	-0.00	0.00	0.00045
S38	tmao	c	-30.73	9.9	0.2819	0.0101	83.39	5.86	0.00022	S38	tmao	c	202.33	54.9	0.08	0.03	0.07645
S38	tmao	h	-7.85	2.8	-0.0637	0.0025	53.89	4.87	0.00003	S38	tmao	h	-57.42	13.7	-0.02	0.01	0.00478
S38	tmao	n	7.54	9.2	-0.3356	0.0094	82.29	4.53	0.00020	S38	tmao	n	-268.49	64.8	-0.09	0.03	0.10667
S50	bet	c	-6.34	31.2	0.0145	0.1010	1000.00	5530.06	0.00002	S50	bet	c	1.58	1.1	0.00	0.00	0.00003
S50	bet	h	-4.07	0.8	-0.0014	0.0005	1.56	7.52	0.00001	S50	bet	h	-4.55	0.7	-0.00	0.00	0.00001
S50	bet	n	-8.65	1.7	-0.0045	0.0013	17.50	16.98	0.00002	S50	bet	n	-11.34	1.4	-0.00	0.00	0.00005
S50	ect	c	-41.00	127.6	0.0794	0.4133	1000.00	4117.66	0.00039	S50	ect	c	1.68	5.0	0.00	0.00	0.00062
S50	ect	h	2.34	1.3	0.0006	0.0009	4.38	39.99	0.00002	S50	ect	h	2.62	0.9	0.00	0.00	0.00002
S50	ect	n	4.28	120.2	0.0080	0.3891	1000.00	38610.45	0.00035	S50	ect	n	9.92	3.7	-0.00	0.00	0.00034
S50	tmao	c	4.24	5.8	0.0265	0.0046	28.40	14.25	0.00017	S50	tmao	c	22.09	6.7	0.01	0.00	0.00113
S50	tmao	h	4.00	5.5	0.0105	0.0070	163.47	168.29	0.00003	S50	tmao	h	13.36	2.1	0.00	0.00	0.00011
S50	tmao	n	-20.37	105.4	0.0733	0.3412	999.43	3682.71	0.00027	S50	tmao	n	19.81	4.4	0.00	0.00	0.00048
S57	bet	c	-8.09	46.0	-0.0064	0.1488	999.80	18527.79	0.00005	S57	bet	c	-12.23	1.4	0.00	0.00	0.00005
S57	bet	h	53.52	2.5	-0.0028	0.0015	1.00	11.46	0.00008	S57	bet	h	52.67	1.9	-0.00	0.00	0.00010
S57	bet	n	97.10	108.4	0.0071	0.3512	1000.00	38914.36	0.00028	S57	bet	n	101.72	3.3	-0.00	0.00	0.00028

S57	ect	c	-417030.95437918.8	342.3582	992.8904	556.92	1557.88	20880.38989	S57	ect	c	-185276.86	36479.4	16.46	16.71	33758.31216
S57	ect	h	100.87	36.2	0.0092	0.1170	997.90	10065.90	0.00003	S57	ect	h	106.42	1.2	-0.00	0.00	0.00003
S57	ect	n	174.72	111.4	0.0201	0.3609	1000.00	14224.87	0.00030	S57	ect	n	185.65	3.5	0.00	0.00	0.00032
S57	tmao	c	-166.88	6.7	-0.0024	0.0041	1.00	35.75	0.00057	S57	tmao	c	-167.60	4.8	-0.00	0.00	0.00058
S57	tmao	h	-6.75	2.4	0.0006	0.0016	9.51	115.45	0.00005	S57	tmao	h	-6.44	1.4	0.00	0.00	0.00005
S57	tmao	n	26.71	8.2	0.0162	0.0068	32.80	37.25	0.00032	S57	tmao	n	38.79	5.6	0.01	0.00	0.00080
S67	bet	c	-9.14	3.2	-0.0056	0.0028	44.48	54.85	0.00004	S67	bet	c	-13.58	1.9	-0.00	0.00	0.00009
S67	bet	h	36.49	48.2	0.0090	0.1562	1000.00	13697.01	0.00006	S67	bet	h	42.18	1.5	-0.00	0.00	0.00006
S67	bet	n	3.92	2.2	-0.0037	0.0014	4.04	10.76	0.00005	S67	bet	n	2.42	1.8	-0.00	0.00	0.00008
S67	ect	c	-419597.58440534.7	344.4553	998.7124	556.81	1557.28	21140.15560	S67	ect	c	-186412.22	36706.3	16.57	16.81	34179.65662
S67	ect	h	68.13	4.4	0.0021	0.0054	149.27	616.05	0.00002	S67	ect	h	70.22	1.0	0.00	0.00	0.00003
S67	ect	n	85.99	1.1	-0.0014	0.0007	1.00	10.19	0.00002	S67	ect	n	85.56	0.9	-0.00	0.00	0.00002
S67	tmao	c	-23.69	17.8	0.0252	0.0197	108.92	151.19	0.00054	S67	tmao	c	-1.13	6.9	0.00	0.00	0.00122
S67	tmao	h	2.48	3.5	-0.0113	0.0037	95.61	58.39	0.00002	S67	tmao	h	-6.76	2.2	-0.00	0.00	0.00012
S67	tmao	n	-38.32	5.5	-0.0247	0.0041	17.99	10.17	0.00020	S67	tmao	n	-53.20	6.8	-0.01	0.00	0.00117
S80	bet	c	-29.05	39.2	-0.0042	0.1270	1000.00	24180.29	0.00004	S80	bet	c	-31.70	1.2	0.00	0.00	0.00004
S80	bet	h	13.51	0.6	-0.0013	0.0005	14.32	19.49	0.00000	S80	bet	h	12.76	0.5	-0.00	0.00	0.00001
S80	bet	n	-0.12	8.7	0.0050	0.0230	725.68	2932.56	0.00000	S80	bet	n	3.05	0.5	0.00	0.00	0.00001
S80	ect	c	-88.90	137.4	0.1830	0.4450	1000.00	1924.24	0.00046	S80	ect	c	6.20	7.4	0.00	0.00	0.00139
S80	ect	h	18.84	3.2	0.0052	0.0041	165.26	201.04	0.00001	S80	ect	h	23.36	1.1	0.00	0.00	0.00003
S80	ect	n	1.53	31.2	0.0164	0.0669	503.31	2053.40	0.00013	S80	ect	n	13.58	2.7	0.00	0.00	0.00018
S80	tmao	c	19.25	3.4	0.0080	0.0023	7.98	11.27	0.00011	S80	tmao	c	23.08	3.0	0.01	0.00	0.00023
S80	tmao	h	18.32	3.3	0.0293	0.0033	75.40	17.18	0.00003	S80	tmao	h	42.43	6.1	0.01	0.00	0.00093
S80	tmao	n	-88.12	9.4	0.0856	0.0113	137.65	29.71	0.00012	S80	tmao	n	-16.44	13.6	0.02	0.01	0.00468
S85	bet	c	-26.98	3.0	0.0026	0.0026	36.15	93.56	0.00004	S85	bet	c	-25.02	1.4	0.00	0.00	0.00005
S85	bet	h	3.20	0.6	-0.0034	0.0004	5.83	4.13	0.00000	S85	bet	h	1.68	1.1	-0.00	0.00	0.00003
S85	bet	n	44.09	3.8	-0.0059	0.0023	1.00	8.20	0.00018	S85	bet	n	42.24	3.3	-0.00	0.00	0.00027
S85	ect	c	36.15	10.4	-0.0072	0.0094	51.28	155.53	0.00038	S85	ect	c	29.78	4.4	-0.00	0.00	0.00048
S85	ect	h	-10.55	2.6	0.0023	0.0022	40.12	97.21	0.00003	S85	ect	h	-8.69	1.2	0.00	0.00	0.00004
S85	ect	n	-7.99	146.6	-0.0184	0.4748	1000.00	20464.01	0.00052	S85	ect	n	-19.54	4.5	0.00	0.00	0.00052
S85	tmao	c	9.77	6.6	0.1884	0.0055	34.14	2.67	0.00020	S85	tmao	c	144.64	44.7	0.08	0.02	0.05067
S85	tmao	h	-19.31	4.5	-0.0881	0.0039	41.88	4.60	0.00008	S85	tmao	h	-84.82	20.0	-0.03	0.01	0.01015
S85	tmao	n	-56.43	12.4	-0.4074	0.0113	52.43	3.34	0.00052	S85	tmao	n	-372.25	88.4	-0.14	0.04	0.19836
S91	bet	c	-29.24	43.3	0.0261	0.1399	998.14	4243.29	0.00005	S91	bet	c	-15.17	1.7	0.00	0.00	0.00007
S91	bet	h	-11.75	0.6	-0.0014	0.0004	2.81	6.98	0.00000	S91	bet	h	-12.26	0.6	-0.00	0.00	0.00001
S91	bet	n	-39.46	2.8	-0.0035	0.0017	2.31	11.96	0.00009	S91	bet	n	-40.73	2.2	-0.00	0.00	0.00012
S91	ect	c	8.01	5.5	0.0029	0.0033	1.00	24.55	0.00038	S91	ect	c	8.87	4.0	0.00	0.00	0.00040
S91	ect	h	-0.09	4.3	-0.0024	0.0069	288.80	1010.32	0.00001	S91	ect	h	-1.97	0.6	-0.00	0.00	0.00001
S91	ect	n	-50.36	5.9	-0.0046	0.0036	1.00	16.62	0.00044	S91	ect	n	-51.78	4.4	-0.00	0.00	0.00049
S91	tmao	c	42.90	100.8	-0.0394	0.3263	999.01	6543.45	0.00025	S91	tmao	c	23.22	3.2	-0.00	0.00	0.00027
S91	tmao	h	-1.45	3.2	0.0092	0.0034	86.80	60.85	0.00002	S91	tmao	h	6.43	2.1	0.00	0.00	0.00012
S91	tmao	n	-53.30	4.3	0.0201	0.0047	103.77	44.24	0.00003	S91	tmao	n	-36.07	4.0	0.00	0.00	0.00041
S92	bet	c	-65.83	2.7	0.0015	0.0018	7.28	44.33	0.00007	S92	bet	c	-65.10	1.7	0.00	0.00	0.00008
S92	bet	h	-10.85	0.8	-0.0016	0.0005	2.76	8.56	0.00001	S92	bet	h	-11.43	0.7	-0.00	0.00	0.00001
S92	bet	n	-44.02	6.4	0.0035	0.0068	95.80	349.10	0.00008	S92	bet	n	-41.01	1.9	0.00	0.00	0.00009
S92	ect	c	-107.09	134.5	0.0946	0.4355	1000.00	3642.91	0.00044	S92	ect	c	-56.47	5.5	0.00	0.00	0.00076
S92	ect	h	-4.21	1.8	0.0018	0.0013	19.71	47.92	0.00002	S92	ect	h	-3.01	1.0	0.00	0.00	0.00003
S92	ect	n	-40.56	113.6	0.0616	0.3675	998.97	4717.74	0.00031	S92	ect	n	-7.61	4.3	0.00	0.00	0.00046
S92	tmao	c	-66.78	10.3	0.0416	0.0126	146.86	71.20	0.00013	S92	tmao	c	-31.86	6.8	0.01	0.00	0.00117
S92	tmao	h	-23.98	42.7	0.0340	0.1382	997.57	3206.60	0.00004	S92	tmao	h	-5.14	1.9	0.00	0.00	0.00009
S92	tmao	n	-65.84	20.4	0.0625	0.0297	227.05	146.59	0.00026	S92	tmao	n	-13.22	9.0	0.01	0.00	0.00206
T100	bet	c	-23.32	77.4	-0.0277	0.2508	1000.00	7168.34	0.00015	T100	bet	c	-40.08	2.5	0.00	0.00	0.00016
T100	bet	h	2.80	0.5	-0.0018	0.0003	3.37	5.19	0.00000	T100	bet	h	2.10	0.7	-0.00	0.00	0.00001
T100	bet	n	-0.34	3.0	0.0002	0.0035	124.29	4216.32	0.00001	T100	bet	n	-0.24	0.7	0.00	0.00	0.00001
T100	ect	c	-108.10	94.3	0.1429	0.3055	1000.00	1691.13	0.00022	T100	ect	c	-33.49	5.7	0.00	0.00	0.00082

T100	ect	h	9.82	6.9	-0.0009	0.0137	433.87	7147.16	0.00001	T100	ect	h	8.94	0.6	0.00	0.00	0.00001
T100	ect	n	9.99	16.0	-0.0335	0.0173	101.05	95.27	0.00047	T100	ect	n	-19.08	8.0	-0.01	0.00	0.00161
T100	tmao	c	9.70	7.2	-0.0118	0.0044	1.00	7.79	0.00065	T100	tmao	c	6.10	6.2	-0.01	0.00	0.00098
T100	tmao	h	0.53	2.4	0.0257	0.0020	31.73	6.70	0.00003	T100	tmao	h	18.76	6.4	0.01	0.00	0.00103
T100	tmao	n	-14.39	7.0	0.0127	0.0042	1.00	7.03	0.00061	T100	tmao	n	-10.49	6.3	0.01	0.00	0.00100
T105	bet	c	-4.15	1.6	0.0038	0.0016	75.51	63.66	0.00001	T105	bet	c	-1.02	0.9	0.00	0.00	0.00002
T105	bet	h	12.09	1.5	-0.0010	0.0009	2.72	23.83	0.00003	T105	bet	h	11.72	1.0	-0.00	0.00	0.00003
T105	bet	n	73.93	56.1	-0.0789	0.1817	1000.00	1821.65	0.00008	T105	bet	n	33.51	2.9	-0.00	0.00	0.00022
T105	ect	c	-64.37	133.7	0.1325	0.4326	998.37	2580.29	0.00044	T105	ect	c	6.44	6.7	0.00	0.00	0.00113
T105	ect	h	14.16	4.3	0.0025	0.0043	81.47	273.76	0.00004	T105	ect	h	16.52	1.4	0.00	0.00	0.00005
T105	ect	n	-38.01	71.3	0.0576	0.1641	571.50	1556.31	0.00052	T105	ect	n	0.29	5.8	0.00	0.00	0.00085
T105	tmao	c	22.20	100.7	-0.0403	0.3262	1000.00	6407.28	0.00025	T105	tmao	c	-1.18	3.4	0.00	0.00	0.00030
T105	tmao	h	12.92	1.3	-0.0093	0.0009	10.76	4.24	0.00001	T105	tmao	h	8.00	2.6	-0.01	0.00	0.00017
T105	tmao	n	40.49	6.8	-0.0616	0.0053	24.95	6.47	0.00026	T105	tmao	n	-0.88	15.9	-0.03	0.01	0.00643
T107	bet	c	6.70	44.2	-0.0025	0.1430	1000.00	44490.32	0.00005	T107	bet	c	4.51	1.3	0.00	0.00	0.00004
T107	bet	h	-6.51	1.0	-0.0012	0.0007	7.87	20.82	0.00001	T107	bet	h	-7.12	0.7	-0.00	0.00	0.00001
T107	bet	n	-16.72	30.0	-0.0510	0.0971	998.45	1505.29	0.00002	T107	bet	n	-43.48	2.0	-0.00	0.00	0.00011
T107	ect	c	38.13	215.9	-0.0871	0.6993	1000.00	6351.85	0.00113	T107	ect	c	-9.70	7.5	-0.00	0.00	0.00143
T107	ect	h	-2.43	2.1	0.0052	0.0022	89.57	73.18	0.00001	T107	ect	h	1.93	1.2	0.00	0.00	0.00004
T107	ect	n	-87.49	99.3	0.1410	0.3212	998.29	1800.62	0.00024	T107	ect	n	-13.17	5.9	0.00	0.00	0.00088
T107	tmao	c	29.43	4.8	0.0197	0.0040	35.63	19.05	0.00010	T107	tmao	c	43.62	5.0	0.01	0.00	0.00064
T107	tmao	h	-0.67	1.3	-0.0049	0.0009	6.24	6.02	0.00002	T107	tmao	h	-2.89	1.6	-0.00	0.00	0.00007
T107	tmao	n	-55.89	6.6	-0.1045	0.0061	58.07	7.56	0.00014	T107	tmao	n	-137.97	22.1	-0.04	0.01	0.01237
T16	bet	c	-99.37	26.0	-0.0008	0.0584	545.72	36677.90	0.00008	T16	bet	c	-100.15	1.7	0.00	0.00	0.00008
T16	bet	h	-12.59	0.9	-0.0021	0.0005	1.00	5.47	0.00001	T16	bet	h	-13.23	0.9	-0.00	0.00	0.00002
T16	bet	n	-42.56	118.0	-0.0636	0.3818	997.97	4743.30	0.00034	T16	bet	n	-77.27	4.5	-0.00	0.00	0.00051
T16	ect	c	-56.12	27.5	-0.0491	0.0499	365.05	419.29	0.00020	T16	ect	c	-93.27	5.3	-0.00	0.00	0.00071
T16	ect	h	1.12	24.3	0.0038	0.0786	1000.00	16494.18	0.00001	T16	ect	h	3.39	0.8	-0.00	0.00	0.00001
T16	ect	n	-71.44	96.6	0.0512	0.3129	1000.00	4832.09	0.00023	T16	ect	n	-44.39	3.5	0.00	0.00	0.00031
T16	tmao	c	-93.60	9.7	0.0030	0.0090	56.76	374.80	0.00030	T16	tmao	c	-90.83	3.5	0.00	0.00	0.00032
T16	tmao	h	-7.59	1.7	-0.0024	0.0010	1.00	9.09	0.00004	T16	tmao	h	-8.32	1.4	-0.00	0.00	0.00005
T16	tmao	n	-53.26	2.5	-0.0066	0.0018	13.76	13.92	0.00005	T16	tmao	n	-57.04	2.2	-0.00	0.00	0.00013
T26	bet	c	6.25	37.9	0.0166	0.1226	1000.00	5845.93	0.00003	T26	bet	c	15.90	1.3	-0.00	0.00	0.00004
T26	bet	h	-11.16	1.3	-0.0024	0.0008	7.77	13.30	0.00001	T26	bet	h	-12.34	1.0	-0.00	0.00	0.00003
T26	bet	n	34.18	14.8	-0.0192	0.0276	384.24	613.66	0.00005	T26	bet	n	19.43	2.3	-0.00	0.00	0.00014
T26	ect	c	41.88	57.5	-0.0033	0.1862	1000.00	45226.25	0.00008	T26	ect	c	39.67	1.8	0.00	0.00	0.00008
T26	ect	h	-14.80	5.4	0.0183	0.0064	133.82	76.97	0.00004	T26	ect	h	0.84	3.3	0.00	0.00	0.00028
T26	ect	n	-3.72	9.5	0.0053	0.0071	17.54	79.83	0.00062	T26	ect	n	-0.40	5.1	0.00	0.00	0.00067
T26	tmao	c	42.88	87.8	-0.0681	0.2843	1000.00	3303.81	0.00019	T26	tmao	c	8.13	3.4	-0.00	0.00	0.00029
T26	tmao	h	11.52	2.2	-0.0782	0.0016	16.94	1.23	0.00003	T26	tmao	h	-35.57	20.4	-0.04	0.01	0.01053
T26	tmao	n	-58.47	11.4	-0.3335	0.0095	34.43	2.61	0.00060	T26	tmao	n	-297.32	78.7	-0.14	0.04	0.15725
T6	bet	c	6.86	2.8	-0.0029	0.0017	1.00	12.48	0.00010	T6	bet	c	5.98	2.2	-0.00	0.00	0.00012
T6	bet	h	22.91	1.1	-0.0022	0.0007	3.56	9.11	0.00001	T6	bet	h	22.04	1.0	-0.00	0.00	0.00003
T6	bet	n	90.67	5.3	-0.0059	0.0032	1.00	11.34	0.00035	T6	bet	n	88.83	4.1	-0.00	0.00	0.00043
T6	ect	c	-558933.77930561.4		837.3243	3013.7813	1000.00	2848.08	20981.10902	T6	ect	c	-113575.95	42144.9	14.33	19.30	45058.39975
T6	ect	h	39.18	1.4	-0.0009	0.0010	6.46	38.75	0.00002	T6	ect	h	38.78	0.9	-0.00	0.00	0.00002
T6	ect	n	123.95	6.4	-0.0129	0.0057	48.70	50.87	0.00015	T6	ect	n	113.77	3.9	-0.00	0.00	0.00039
T6	tmao	c	8.06	62.2	-0.0128	0.2009	997.37	12355.81	0.00009	T6	tmao	c	1.57	2.0	-0.00	0.00	0.00010
T6	tmao	h	24.70	2.9	0.0116	0.0030	81.90	41.54	0.00002	T6	tmao	h	34.56	2.6	0.00	0.00	0.00017
T6	tmao	n	140.45	9.4	0.0201	0.0079	37.37	38.23	0.00038	T6	tmao	n	156.01	6.6	0.01	0.00	0.00111
T70	bet	c	63.67	1.6	0.0011	0.0010	1.00	18.34	0.00003	T70	bet	c	64.00	1.2	0.00	0.00	0.00004
T70	bet	h	4.22	0.7	-0.0016	0.0004	1.12	6.06	0.00001	T70	bet	h	3.71	0.7	-0.00	0.00	0.00001
T70	bet	n	186.58	176.1	0.0175	0.5702	1000.00	25853.56	0.00075	T70	bet	n	197.11	5.5	-0.00	0.00	0.00076
T70	ect	c	127.99	190.0	-0.0655	0.6152	1000.00	7433.48	0.00087	T70	ect	c	95.10	6.1	-0.00	0.00	0.00094
T70	ect	h	9.18	1.3	0.0023	0.0011	38.70	49.26	0.00001	T70	ect	h	10.93	0.8	0.00	0.00	0.00002
T70	ect	n	244.06	132.0	-0.0514	0.4273	1000.00	6583.62	0.00042	T70	ect	n	218.40	4.2	-0.00	0.00	0.00045

T70	tmao	c	92.47	18.0	0.0293	0.0174	68.35	85.20	0.00088	T70	tmao	c	116.84	8.8	0.01	0.00	0.00194
T70	tmao	h	-3.97	1.8	-0.0059	0.0012	10.67	9.35	0.00003	T70	tmao	h	-7.04	1.9	-0.00	0.00	0.00009
T70	tmao	n	162.64	19.2	-0.1302	0.0214	112.21	32.52	0.00061	T70	tmao	n	54.05	22.7	-0.03	0.01	0.01305
T79	bet	c	-20.80	3.3	0.0052	0.0029	48.38	64.15	0.00004	T79	bet	c	-16.81	1.7	0.00	0.00	0.00007
T79	bet	h	-17.99	1.4	-0.0017	0.0009	2.77	13.50	0.00002	T79	bet	h	-18.61	1.1	-0.00	0.00	0.00003
T79	bet	n	-19.96	73.6	-0.0563	0.2382	998.97	3342.02	0.00013	T79	bet	n	-48.80	2.8	-0.00	0.00	0.00020
T79	ect	c	-14.30	14.1	0.0052	0.0215	251.86	1346.64	0.00011	T79	ect	c	-10.13	2.1	0.00	0.00	0.00011
T79	ect	h	-21.17	1.0	-0.0007	0.0007	15.59	63.66	0.00001	T79	ect	h	-21.59	0.6	-0.00	0.00	0.00001
T79	ect	n	-79.87	106.8	0.0664	0.3458	1000.00	4118.64	0.00028	T79	ect	n	-46.56	3.6	0.00	0.00	0.00034
T79	tmao	c	50.02	134.4	-0.1477	0.4352	999.97	2332.28	0.00044	T79	tmao	c	-25.58	6.1	-0.00	0.00	0.00093
T79	tmao	h	-39.77	3.5	-0.0472	0.0031	46.71	7.27	0.00004	T79	tmao	h	-75.50	10.4	-0.02	0.00	0.00277
T79	tmao	n	-104.72	72.5	-0.0862	0.2347	1000.00	2153.93	0.00013	T79	tmao	n	-151.56	4.0	-0.00	0.00	0.00041
T99	bet	c	4.64	1.6	0.0015	0.0011	9.20	30.90	0.00002	T99	bet	c	5.40	1.1	0.00	0.00	0.00003
T99	bet	h	3.43	0.9	-0.0010	0.0006	3.69	16.99	0.00001	T99	bet	h	3.04	0.7	-0.00	0.00	0.00001
T99	bet	n	-2.23	1.4	-0.0032	0.0008	1.30	5.68	0.00002	T99	bet	n	-3.27	1.4	-0.00	0.00	0.00005
T99	ect	c	-411178.14431609.5		337.5302	978.4487	556.78	1556.93	20294.78977	T99	ect	c	-182676.00	35966.4	16.23	16.47	32815.59658
T99	ect	h	8.62	0.8	0.0015	0.0006	25.84	30.40	0.00000	T99	ect	h	9.68	0.5	0.00	0.00	0.00001
T99	ect	n	-88.96	88.1	0.1323	0.2524	826.45	1325.61	0.00032	T99	ect	n	-12.42	6.9	0.00	0.00	0.00119
T99	tmao	c	44.73	6.4	0.0690	0.0051	27.82	5.98	0.00022	T99	tmao	c	92.22	17.4	0.03	0.01	0.00768
T99	tmao	h	2.46	59.7	0.0180	0.1935	1000.00	8480.64	0.00009	T99	tmao	h	13.76	1.8	-0.00	0.00	0.00009
T99	tmao	n	-2.39	9.7	-0.0841	0.0076	24.52	6.70	0.00054	T99	tmao	n	-57.84	21.2	-0.04	0.01	0.01135
V10	bet	c	20.30	43.5	-0.0315	0.1408	998.97	3532.72	0.00005	V10	bet	c	4.80	1.4	-0.00	0.00	0.00005
V10	bet	h	-0.25	0.6	-0.0018	0.0004	4.37	5.84	0.00000	V10	bet	h	-1.00	0.6	-0.00	0.00	0.00001
V10	bet	n	-9.04	4.0	-0.0074	0.0039	68.56	74.71	0.00004	V10	bet	n	-14.98	2.0	-0.00	0.00	0.00010
V10	ect	c	45.58	4.1	-0.0079	0.0029	11.15	16.94	0.00014	V10	ect	c	41.27	3.2	-0.00	0.00	0.00027
V10	ect	h	15.34	3.8	0.0024	0.0044	133.14	409.36	0.00002	V10	ect	h	17.35	0.9	0.00	0.00	0.00002
V10	ect	n	16.26	116.2	0.0256	0.3764	1000.00	11633.55	0.00033	V10	ect	n	31.85	3.6	-0.00	0.00	0.00034
V10	tmao	c	12.97	27.2	0.0531	0.0506	385.11	407.03	0.00018	V10	tmao	c	53.33	5.6	0.00	0.00	0.00079
V10	tmao	h	8.72	2.9	0.0215	0.0033	117.40	30.77	0.00001	V10	tmao	h	27.02	3.9	0.00	0.00	0.00038
V10	tmao	n	-2.77	9.5	0.0637	0.0098	85.15	25.47	0.00020	V10	tmao	n	49.69	12.4	0.02	0.01	0.00392
V36	bet	c	37.20	22.4	-0.0129	0.0135	1.00	22.15	0.00628	V36	bet	c	33.93	16.0	-0.01	0.01	0.00651
V36	bet	h	3.75	1.1	-0.0019	0.0007	1.00	7.49	0.00002	V36	bet	h	3.17	1.0	-0.00	0.00	0.00002
V36	bet	n	-21.18	76.7	0.0479	0.2484	999.16	4105.06	0.00014	V36	bet	n	5.35	3.1	0.00	0.00	0.00024
V36	ect	c	74.85	62.2	-0.0675	0.0845	191.87	347.14	0.00318	V36	ect	c	16.51	15.2	-0.01	0.01	0.00584
V36	ect	h	-6.41	29.2	-0.0016	0.0946	1000.00	48231.78	0.00002	V36	ect	h	-7.14	0.9	-0.00	0.00	0.00002
V36	ect	n	-113.20	148.3	0.1108	0.4801	999.32	3426.38	0.00053	V36	ect	n	-52.75	6.3	0.00	0.00	0.00102
V36	tmao	c	-10.37	7.7	-0.0601	0.0072	56.49	15.09	0.00019	V36	tmao	c	-57.50	13.1	-0.02	0.01	0.00436
V36	tmao	h	-38.03	5.0	-0.0627	0.0052	90.12	14.18	0.00005	V36	tmao	h	-89.51	11.5	-0.02	0.01	0.00336
V36	tmao	n	-206.24	22.6	-0.0463	0.0263	126.66	121.14	0.00073	V36	tmao	n	-243.80	8.6	-0.01	0.00	0.00186
V3	bet	c	-31.87	4.5	-0.0026	0.0038	38.01	143.50	0.00009	V3	bet	c	-34.13	2.0	-0.00	0.00	0.00011
V3	bet	h	31.45	1.3	-0.0016	0.0008	1.00	10.24	0.00002	V3	bet	h	30.96	1.0	-0.00	0.00	0.00003
V3	bet	n	79.80	54.3	-0.0312	0.1759	1000.00	4455.16	0.00007	V3	bet	n	63.13	2.0	-0.00	0.00	0.00011
V3	ect	c	-25.30	5.3	0.0203	0.0046	44.12	24.59	0.00011	V3	ect	c	-10.13	4.9	0.01	0.00	0.00062
V3	ect	h	52.54	5.3	-0.0068	0.0046	38.93	66.43	0.00012	V3	ect	h	47.23	2.8	-0.00	0.00	0.00020
V3	ect	n	78.49	7.1	-0.0032	0.0048	9.41	64.44	0.00044	V3	ect	n	76.82	4.3	-0.00	0.00	0.00046
V3	tmao	c	-15.10	7.7	-0.0101	0.0047	1.00	9.75	0.00075	V3	tmao	c	-18.18	6.2	-0.01	0.00	0.00099
V3	tmao	h	6.21	1.3	0.0100	0.0010	14.84	5.23	0.00001	V3	tmao	h	12.03	2.8	0.01	0.00	0.00019
V3	tmao	n	112.05	11.5	-0.0079	0.0129	113.07	322.98	0.00022	V3	tmao	n	104.80	3.4	-0.00	0.00	0.00029
V45	bet	c	-38.78	1.9	-0.0023	0.0011	1.00	10.38	0.00004	V45	bet	c	-39.49	1.5	-0.00	0.00	0.00006
V45	bet	h	3.69	0.8	-0.0022	0.0005	1.00	4.55	0.00001	V45	bet	h	3.03	0.9	-0.00	0.00	0.00002
V45	bet	n	-56.54	12.9	0.0025	0.0126	69.46	741.22	0.00045	V45	bet	n	-53.94	4.3	0.00	0.00	0.00046
V45	ect	c	-26.51	105.4	0.0165	0.3414	1000.00	16402.25	0.00027	V45	ect	c	-16.40	3.3	-0.00	0.00	0.00027
V45	ect	h	15.43	5.8	0.0114	0.0108	390.06	409.99	0.00001	V45	ect	h	23.96	1.2	0.00	0.00	0.00003
V45	ect	n	-42.42	71.9	0.0870	0.2327	1000.00	2117.37	0.00013	V45	ect	n	3.76	3.9	0.00	0.00	0.00038
V45	tmao	c	-28.20	15.4	0.0806	0.0166	99.90	37.82	0.00045	V45	tmao	c	40.48	16.0	0.02	0.01	0.00651
V45	tmao	h	24.34	3.5	0.0646	0.0036	84.23	9.16	0.00003	V45	tmao	h	77.88	12.6	0.02	0.01	0.00405

V45	tmao	n	-28.07	11.2	0.2559	0.0119	97.41	8.41	0.00024	V45	tmao	n	185.40	47.0	0.06	0.02	0.05601
W35	bet	c	-35.02	5.9	0.0049	0.0059	76.76	188.05	0.00009	W35	bet	c	-30.94	2.1	0.00	0.00	0.00011
W35	bet	h	9.70	0.8	-0.0013	0.0005	1.00	7.74	0.00001	W35	bet	h	9.31	0.7	-0.00	0.00	0.00001
W35	bet	n	33.89	78.3	0.0438	0.2537	999.75	4578.91	0.00015	W35	bet	n	58.12	3.0	0.00	0.00	0.00022
W35	ect	c	3.60	97.6	-0.0772	0.3162	1000.00	3243.06	0.00023	W35	ect	c	-36.73	4.0	-0.00	0.00	0.00041
W35	ect	h	9.26	1.3	0.0049	0.0013	75.56	39.70	0.00000	W35	ect	h	13.32	1.1	0.00	0.00	0.00003
W35	ect	n	46.54	105.6	0.1892	0.3308	949.58	1334.95	0.00031	W35	ect	n	146.61	7.2	0.01	0.00	0.00133
W35	tmao	c	-67.09	226.9	-0.0245	0.7349	1000.00	23703.62	0.00125	W35	tmao	c	-83.96	6.8	0.00	0.00	0.00118
W35	tmao	h	13.11	45.2	0.0167	0.1465	1000.00	6938.86	0.00005	W35	tmao	h	23.00	1.5	-0.00	0.00	0.00006
W35	tmao	n	135.11	13.1	0.4282	0.0137	89.21	5.44	0.00036	W35	tmao	n	489.90	80.8	0.11	0.04	0.16547
W71	bet	c	-0.99	4.3	-0.0050	0.0038	51.19	90.55	0.00006	W71	bet	c	-4.95	1.9	-0.00	0.00	0.00010
W71	bet	h	36.07	2.1	-0.0013	0.0013	1.66	23.00	0.00005	W71	bet	h	35.64	1.5	-0.00	0.00	0.00006
W71	bet	n	337.69	15.3	-0.0023	0.0107	11.33	214.03	0.00194	W71	bet	n	336.38	8.8	-0.00	0.00	0.00195
W71	ect	c	2.77	18.9	0.0217	0.0319	316.33	556.34	0.00013	W71	ect	c	19.11	2.9	0.00	0.00	0.00022
W71	ect	h	43.57	2.6	0.0007	0.0025	74.35	522.09	0.00002	W71	ect	h	44.31	0.8	0.00	0.00	0.00002
W71	ect	n	368.41	25.9	-0.0401	0.0327	158.99	201.04	0.00072	W71	ect	n	335.80	7.5	-0.01	0.00	0.00143
W71	tmao	c	0.00	0.0	-0.0000	0.0000	500.50	0.00	0.00000	W71	tmao	c	0.00	0.0	0.00	0.00	0.00000
W71	tmao	h	35.86	5.5	0.0184	0.0072	172.31	101.87	0.00003	W71	tmao	h	51.62	3.0	0.00	0.00	0.00023
W71	tmao	n	355.11	23.4	0.0464	0.0222	63.07	65.06	0.00159	W71	tmao	n	394.24	13.6	0.01	0.01	0.00469
W94	bet	c	47.63	3.7	-0.0024	0.0023	2.61	24.96	0.00016	W94	bet	c	46.76	2.6	-0.00	0.00	0.00018
W94	bet	h	-14.75	0.5	-0.0022	0.0003	1.00	2.95	0.00000	W94	bet	h	-15.44	0.8	-0.00	0.00	0.00002
W94	bet	n	-195.14	215.0	-0.1293	0.6962	1000.00	4260.32	0.00112	W94	bet	n	-263.51	8.1	-0.00	0.00	0.00167
W94	ect	c	68.74	7.1	-0.0036	0.0050	13.00	71.40	0.00039	W94	ect	c	66.66	4.1	-0.00	0.00	0.00042
W94	ect	h	1.59	1.5	-0.0011	0.0009	1.96	19.82	0.00003	W94	ect	h	1.21	1.1	-0.00	0.00	0.00003
W94	ect	n	-222.32	132.3	0.1205	0.4282	998.69	2809.18	0.00043	W94	ect	n	-157.13	6.4	0.00	0.00	0.00103
W94	tmao	c	30.17	25.5	0.0236	0.0372	227.73	485.21	0.00041	W94	tmao	c	51.84	5.5	0.00	0.00	0.00076
W94	tmao	h	-4.95	8.7	0.0154	0.0121	202.69	226.12	0.00006	W94	tmao	h	8.44	2.8	0.00	0.00	0.00019
W94	tmao	n	-292.01	87.1	0.2757	0.2822	1000.00	809.73	0.00018	W94	tmao	n	-148.59	9.6	0.01	0.00	0.00233
Y103	bet	c	-20.33	2.1	0.0034	0.0013	1.00	7.86	0.00005	Y103	bet	c	-19.27	1.8	0.00	0.00	0.00008
Y103	bet	h	1.70	1.0	-0.0021	0.0006	3.01	8.29	0.00001	Y103	bet	h	0.92	0.9	-0.00	0.00	0.00002
Y103	bet	n	-96.40	70.9	-0.0440	0.2295	1000.00	4127.97	0.00012	Y103	bet	n	-121.03	2.8	0.00	0.00	0.00020
Y103	ect	c	-16.95	5.3	-0.0055	0.0032	1.00	12.20	0.00035	Y103	ect	c	-18.62	4.1	-0.00	0.00	0.00042
Y103	ect	h	-0.50	1.7	0.0024	0.0015	50.10	76.93	0.00001	Y103	ect	h	1.37	0.9	0.00	0.00	0.00002
Y103	ect	n	-238.43	94.2	0.0150	0.3049	1000.00	16132.43	0.00021	Y103	ect	n	-228.97	2.9	-0.00	0.00	0.00021
Y103	tmao	c	10.97	8.5	0.0473	0.0097	117.99	41.90	0.00011	Y103	tmao	c	51.13	8.6	0.01	0.00	0.00186
Y103	tmao	h	7.36	2.0	-0.0132	0.0016	32.78	11.04	0.00002	Y103	tmao	h	-1.94	3.2	-0.01	0.00	0.00026
Y103	tmao	n	-107.88	5.4	-0.0726	0.0042	25.33	4.39	0.00016	Y103	tmao	n	-156.61	18.4	-0.03	0.01	0.00855
Y13	bet	c	-7.68	3.1	0.0030	0.0023	16.52	44.29	0.00007	Y13	bet	c	-5.92	1.8	0.00	0.00	0.00008
Y13	bet	h	-0.26	0.6	-0.0022	0.0004	1.00	3.61	0.00000	Y13	bet	h	-0.94	0.8	-0.00	0.00	0.00002
Y13	bet	n	44.74	2.6	-0.0020	0.0017	4.07	23.51	0.00007	Y13	bet	n	43.90	1.8	-0.00	0.00	0.00008
Y13	ect	c	-30.04	118.1	0.0828	0.3823	1000.00	3653.15	0.00034	Y13	ect	c	14.93	4.9	0.00	0.00	0.00060
Y13	ect	h	15.98	13.4	0.0058	0.0318	599.90	3085.37	0.00002	Y13	ect	h	19.65	0.9	0.00	0.00	0.00002
Y13	ect	n	11.25	118.1	0.1113	0.3825	1000.00	2719.79	0.00034	Y13	ect	n	71.34	5.6	0.00	0.00	0.00080
Y13	tmao	c	17.76	3.8	-0.0076	0.0027	14.96	19.77	0.00011	Y13	tmao	c	13.21	2.9	-0.00	0.00	0.00022
Y13	tmao	h	19.80	6.5	0.0148	0.0090	197.09	171.32	0.00003	Y13	tmao	h	32.66	2.5	0.00	0.00	0.00016
Y13	tmao	n	93.53	4.4	0.0062	0.0027	2.01	10.43	0.00024	Y13	tmao	n	95.69	3.6	0.00	0.00	0.00033
Y17	bet	c	-5.39	94.4	-0.0376	0.3056	1000.00	6429.58	0.00022	Y17	bet	c	-26.48	3.3	0.00	0.00	0.00027
Y17	bet	h	-17.07	1.1	-0.0023	0.0007	7.88	12.48	0.00001	Y17	bet	h	-18.20	0.9	-0.00	0.00	0.00002
Y17	bet	n	-139.36	159.3	-0.0632	0.5159	1000.00	6460.72	0.00061	Y17	bet	n	-171.91	5.3	-0.00	0.00	0.00071
Y17	ect	c	-12.86	163.7	-0.0657	0.5303	1000.00	6391.26	0.00065	Y17	ect	c	-49.54	5.7	0.00	0.00	0.00082
Y17	ect	h	-16.65	23.4	0.0115	0.0756	999.41	5200.99	0.00001	Y17	ect	h	-10.66	0.8	0.00	0.00	0.00002
Y17	ect	n	-205.42	144.8	0.0563	0.4690	1000.00	6591.16	0.00051	Y17	ect	n	-175.88	4.9	0.00	0.00	0.00061
Y17	tmao	c	-1.53	7.7	0.0036	0.0054	11.91	72.17	0.00049	Y17	tmao	c	0.56	4.5	0.00	0.00	0.00052
Y17	tmao	h	-11.98	50.5	0.0062	0.1634	1000.00	20944.76	0.00006	Y17	tmao	h	-7.44	1.4	-0.00	0.00	0.00005
Y17	tmao	n	-102.19	4.5	-0.0094	0.0027	1.00	6.15	0.00025	Y17	tmao	n	-105.03	4.3	-0.01	0.00	0.00046
Y24	bet	c	-57.45	3.1	-0.0023	0.0019	1.00	17.81	0.00012	Y24	bet	c	-58.13	2.3	-0.00	0.00	0.00014
Y24	bet	h	-5.25	0.8	-0.0016	0.0005	2.90	7.97	0.00001	Y24	bet	h	-5.84	0.7	-0.00	0.00	0.00001

Y24	bet	n	51.73	4.0	-0.0082	0.0038	57.29	58.32	0.00005	Y24	bet	n	45.41	2.2	-0.00	0.00	0.00012
Y24	ect	c	135.49	221.1	-0.2297	0.7162	1000.00	2467.14	0.00118	Y24	ect	c	13.98	10.7	-0.00	0.00	0.00293
Y24	ect	h	18.72	23.5	0.0051	0.0665	811.45	8980.47	0.00002	Y24	ect	h	21.55	1.0	0.00	0.00	0.00002
Y24	ect	n	33.42	114.6	0.0296	0.3706	998.11	9902.63	0.00032	Y24	ect	n	49.87	3.7	0.00	0.00	0.00036
Y24	tmao	c	44.89	5.6	0.1050	0.0050	51.78	5.74	0.00011	Y24	tmao	c	126.08	22.9	0.04	0.01	0.01328
Y24	tmao	h	3.79	4.4	0.0175	0.0059	184.84	91.64	0.00002	Y24	tmao	h	18.77	2.8	0.00	0.00	0.00019
Y24	tmao	n	15.42	6.1	-0.0418	0.0053	42.33	13.24	0.00015	Y24	tmao	n	-15.41	9.5	-0.02	0.00	0.00228
Y78	bet	c	11.41	3.0	-0.0045	0.0026	39.17	57.15	0.00004	Y78	bet	c	7.87	1.7	-0.00	0.00	0.00008
Y78	bet	h	5.79	1.1	-0.0019	0.0007	1.00	7.72	0.00002	Y78	bet	h	5.21	1.0	-0.00	0.00	0.00002
Y78	bet	n	31.15	3.5	-0.0027	0.0030	37.48	107.48	0.00005	Y78	bet	n	29.06	1.6	-0.00	0.00	0.00007
Y78	ect	c	10.46	126.5	0.0716	0.4095	1000.00	4523.77	0.00039	Y78	ect	c	50.32	4.8	-0.00	0.00	0.00059
Y78	ect	h	-17.69	17.0	0.0496	0.0524	925.41	792.04	0.00001	Y78	ect	h	8.91	1.8	0.00	0.00	0.00008
Y78	ect	n	11.05	99.2	0.0863	0.3209	998.86	2940.45	0.00024	Y78	ect	n	56.23	4.3	0.00	0.00	0.00047
Y78	tmao	c	48.10	14.5	0.0957	0.0151	88.17	26.62	0.00045	Y78	tmao	c	128.56	19.3	0.02	0.01	0.00950
Y78	tmao	h	-13.26	2.0	-0.0035	0.0012	1.00	7.19	0.00005	Y78	tmao	h	-14.32	1.7	-0.00	0.00	0.00008
Y78	tmao	n	-179.49	230.7	0.2182	0.7470	1000.00	2708.93	0.00129	Y78	tmao	n	-63.07	10.8	0.00	0.00	0.00296
Y90	bet	h	7.01	1.2	-0.0013	0.0007	1.00	11.92	0.00002	Y90	bet	h	6.63	0.9	-0.00	0.00	0.00002
Y90	bet	n	37.65	37.4	-0.0106	0.0983	717.78	5868.62	0.00008	Y90	bet	n	30.42	1.9	-0.00	0.00	0.00009
Y90	ect	c	0.79	48.9	0.0099	0.0995	456.64	4771.00	0.00041	Y90	ect	c	8.66	4.1	0.00	0.00	0.00043
Y90	ect	h	13.39	29.8	0.0026	0.0965	1000.00	29705.66	0.00002	Y90	ect	h	15.03	0.9	-0.00	0.00	0.00002
Y90	ect	n	13.26	174.3	0.0318	0.5645	1000.00	14061.01	0.00074	Y90	ect	n	34.23	5.2	-0.00	0.00	0.00068
Y90	tmao	c	27.26	46.7	-0.0437	0.1513	1000.00	2740.92	0.00005	Y90	tmao	c	3.77	2.2	-0.00	0.00	0.00012
Y90	tmao	h	13.61	4.7	0.0116	0.0059	158.48	125.91	0.00002	Y90	tmao	h	23.72	2.2	0.00	0.00	0.00012
Y90	tmao	n	31.86	8.1	0.0137	0.0082	82.99	97.76	0.00015	Y90	tmao	n	43.92	3.9	0.00	0.00	0.00039
Y97	bet	c	22.65	5.8	-0.0155	0.0097	308.02	233.40	0.00001	Y97	bet	c	10.60	1.7	-0.00	0.00	0.00007
Y97	bet	h	-4.31	1.0	-0.0015	0.0006	1.00	9.10	0.00001	Y97	bet	h	-4.76	0.9	-0.00	0.00	0.00002
Y97	bet	n	-20.84	47.3	0.0032	0.1533	1000.00	37814.73	0.00005	Y97	bet	n	-17.73	1.2	-0.00	0.00	0.00004
Y97	ect	c	23.78	4.3	-0.0033	0.0026	1.00	16.49	0.00023	Y97	ect	c	22.77	3.2	-0.00	0.00	0.00025
Y97	ect	h	-9.83	9.5	0.0160	0.0209	526.28	678.62	0.00001	Y97	ect	h	1.12	1.3	0.00	0.00	0.00004
Y97	ect	n	-73.47	69.6	0.1068	0.2253	998.79	1667.14	0.00012	Y97	ect	n	-16.92	4.5	0.00	0.00	0.00051
Y97	tmao	c	6.70	7.1	0.0058	0.0043	1.00	15.64	0.00064	Y97	tmao	c	8.45	5.3	0.00	0.00	0.00072
Y97	tmao	h	-3.24	5.1	0.0217	0.0059	129.28	59.03	0.00004	Y97	tmao	h	15.41	3.9	0.00	0.00	0.00040
Y97	tmao	n	-28.62	11.1	0.0534	0.0125	113.26	46.43	0.00020	Y97	tmao	n	17.15	10.2	0.01	0.00	0.00263
A102	clso4	c	-67.41	188.0	0.1413	0.6089	1000.00	3408.93	0.00085	A102	clso4	c	11.71	7.6	-0.00	0.00	0.00152
A102	clso4	h	21.30	35.6	0.0388	0.1152	1000.00	2348.39	0.00003	A102	clso4	h	40.81	1.3	0.00	0.00	0.00004
A102	clso4	n	70.73	12.8	0.1359	0.0087	11.03	2.56	0.00148	A102	clso4	n	151.96	35.4	0.07	0.02	0.03335
A102	scncl	c	-30.06	9.5	0.0073	0.0054	1.00	9.40	0.00134	A102	scncl	c	-28.14	7.3	0.01	0.00	0.00142
A102	scncl	h	10.37	6.2	0.0093	0.0056	53.24	72.98	0.00013	A102	scncl	h	18.71	3.2	0.00	0.00	0.00027
A102	scncl	n	101.86	13.1	0.1848	0.0126	67.02	9.54	0.00048	A102	scncl	n	259.02	36.0	0.05	0.02	0.03442
A102	scnso4	c	263.94	191.9	-0.5127	0.6215	1000.00	959.45	0.00088	A102	scnso4	c	-5.30	15.1	-0.01	0.01	0.00634
A102	scnso4	h	22.10	5.1	0.0239	0.0051	78.67	33.11	0.00006	A102	scnso4	h	43.60	4.4	0.01	0.00	0.00054
A102	scnso4	n	129.38	24.6	0.1766	0.0176	16.09	5.14	0.00467	A102	scnso4	n	256.86	43.9	0.08	0.02	0.05317
A11	cl	c	10.97	82.2	0.1225	0.2284	787.04	1253.32	0.00031	A11	cl	c	81.60	6.2	0.00	0.00	0.00097
A11	cl	h	10.16	10.1	0.0121	0.0112	109.48	180.07	0.00017	A11	cl	h	20.90	3.6	0.00	0.00	0.00033
A11	cl	n	-49.03	7.5	0.0158	0.0080	97.96	92.32	0.00011	A11	cl	n	-36.17	3.4	0.00	0.00	0.00029
A11	clso4	c	68.36	22.2	0.0990	0.0252	117.51	51.76	0.00078	A11	clso4	c	152.61	15.1	0.02	0.01	0.00609
A11	clso4	h	40.79	2.6	0.0120	0.0019	19.64	9.60	0.00005	A11	clso4	h	49.10	3.2	0.01	0.00	0.00027
A11	clso4	n	-68.76	37.9	-0.0249	0.0668	345.24	1071.41	0.00043	A11	clso4	n	-90.04	4.8	-0.00	0.00	0.00062
A11	scn	c	-36.99	33.0	0.2051	0.0875	698.50	269.38	0.00010	A11	scn	c	81.06	10.8	0.01	0.01	0.00336
A11	scncl	c	-75.51	73.8	0.2581	0.1789	625.68	401.01	0.00045	A11	scncl	c	93.06	14.3	0.01	0.01	0.00544
A11	scncl	h	4.40	3.9	0.0085	0.0028	18.57	19.51	0.00010	A11	scncl	h	10.43	3.0	0.00	0.00	0.00024
A11	scncl	n	-111.41	4.4	-0.0090	0.0025	1.00	3.53	0.00028	A11	scncl	n	-113.95	4.0	-0.01	0.00	0.00043
A11	scn	h	-10.06	2.5	0.0157	0.0024	54.50	21.40	0.00003	A11	scn	h	0.31	3.5	0.01	0.00	0.00035
A11	scn	n	-101.63	16.2	0.0053	0.0238	207.34	1353.15	0.00026	A11	scn	n	-96.44	3.2	0.00	0.00	0.00029
A11	scnso4	c	46.22	26.1	0.1079	0.0278	96.63	46.02	0.00134	A11	scnso4	c	142.12	17.8	0.02	0.01	0.00880
A11	scnso4	h	-0.28	6.5	0.0277	0.0062	66.68	31.21	0.00012	A11	scnso4	h	24.02	5.2	0.01	0.00	0.00076
A11	scnso4	n	-109.27	131.3	-0.0657	0.4253	1000.00	5125.02	0.00041	A11	scnso4	n	-147.51	4.3	0.00	0.00	0.00052

A11	so4	c	67.07	39.2	0.0947	0.0607	258.94	212.34	0.00078	A11	so4	c	138.76	11.3	0.01	0.01	0.00354
A11	so4	h	23.35	2.4	0.0096	0.0018	7.46	9.19	0.00006	A11	so4	h	27.47	3.0	0.01	0.00	0.00024
A11	so4	n	-64.42	143.8	-0.0151	0.4629	1000.00	24067.54	0.00052	A11	so4	n	-76.37	3.9	0.00	0.00	0.00043
A30	cl	c	16.27	16.0	0.0363	0.0205	165.33	142.78	0.00026	A30	cl	c	46.88	6.3	0.01	0.00	0.00101
A30	cl	h	9.29	10.5	0.0211	0.0108	86.23	85.58	0.00024	A30	cl	h	27.04	5.2	0.01	0.00	0.00070
A30	cl	n	32.02	11.0	0.1989	0.0138	154.70	16.83	0.00013	A30	cl	n	198.20	29.8	0.04	0.01	0.02254
A30	clso4	c	45.26	4.7	0.0428	0.0030	7.09	2.09	0.00023	A30	clso4	c	67.54	11.4	0.03	0.00	0.00344
A30	clso4	h	17.60	2.3	0.0453	0.0015	9.74	1.24	0.00005	A30	clso4	h	43.81	11.8	0.03	0.01	0.00369
A30	clso4	n	63.77	15.8	0.2518	0.0112	15.02	2.22	0.00197	A30	clso4	n	227.83	63.7	0.13	0.03	0.10788
A30	scn	c	-12.21	11.1	0.1110	0.0112	64.61	15.46	0.00047	A30	scn	c	62.21	22.1	0.04	0.01	0.01399
A30	scncl	c	14.65	8.8	0.0771	0.0067	22.13	5.74	0.00050	A30	scncl	c	69.65	19.2	0.03	0.01	0.00986
A30	scncl	h	-13.63	3.4	0.0478	0.0024	15.28	2.56	0.00009	A30	scncl	h	17.81	12.4	0.02	0.01	0.00412
A30	scncl	n	-74.39	18.4	0.2881	0.0139	22.07	3.20	0.00216	A30	scncl	n	130.66	70.4	0.13	0.03	0.13189
A30	scn	h	-21.07	1.3	0.0556	0.0011	29.40	1.91	0.00001	A30	scn	h	10.75	13.0	0.03	0.01	0.00482
A30	scn	n	-89.15	17.1	0.2971	0.0160	45.63	6.71	0.00148	A30	scn	n	97.84	63.8	0.13	0.03	0.11632
A30	scnso4	c	27.20	7.8	0.0894	0.0056	16.87	3.36	0.00046	A30	scnso4	c	93.72	22.6	0.04	0.01	0.01412
A30	scnso4	h	-19.19	3.9	0.0736	0.0028	14.87	1.83	0.00012	A30	scnso4	h	34.62	19.0	0.03	0.01	0.01000
A30	scnso4	n	-133.44	13.4	0.3213	0.0097	17.91	1.70	0.00130	A30	scnso4	n	111.22	81.7	0.14	0.03	0.18439
A30	so4	c	29.46	6.6	0.0609	0.0054	22.96	7.22	0.00028	A30	so4	c	64.05	14.7	0.03	0.01	0.00605
A30	so4	h	29.64	3.8	0.0614	0.0032	25.24	4.40	0.00009	A30	so4	h	65.35	14.4	0.03	0.01	0.00577
A30	so4	n	54.98	11.7	0.2898	0.0101	32.10	3.39	0.00070	A30	so4	n	233.71	64.8	0.14	0.03	0.11732
A32	cl	c	21.97	9.4	0.1073	0.0101	100.32	17.37	0.00016	A32	cl	c	110.68	19.1	0.03	0.01	0.00926
A32	cl	h	-12.50	6.8	0.0291	0.0064	61.10	29.24	0.00014	A32	cl	h	10.94	6.8	0.01	0.00	0.00117
A32	cl	n	48.40	6.7	0.0886	0.0064	60.75	9.55	0.00014	A32	cl	n	118.14	18.4	0.03	0.01	0.00863
A32	clso4	c	26.26	12.5	0.1436	0.0106	39.95	7.31	0.00067	A32	clso4	c	141.11	31.8	0.05	0.01	0.02682
A32	clso4	h	7.27	4.7	0.0338	0.0044	62.12	17.43	0.00007	A32	clso4	h	35.73	6.7	0.01	0.00	0.00120
A32	clso4	n	213.72	5.9	0.0631	0.0047	30.04	6.12	0.00019	A32	clso4	n	262.35	15.2	0.02	0.01	0.00615
A32	scn	c	-66.06	13.6	0.0859	0.0211	235.41	79.80	0.00015	A32	scn	c	-2.75	11.0	0.01	0.01	0.00346
A32	scncl	c	-41.07	10.6	0.0839	0.0076	15.81	4.74	0.00087	A32	scncl	c	14.37	22.1	0.04	0.01	0.01301
A32	scncl	h	-28.01	91.0	-0.0159	0.2946	1000.00	14689.26	0.00020	A32	scncl	h	-35.93	2.8	-0.00	0.00	0.00020
A32	scncl	n	212.63	258.7	-0.1939	0.8378	1000.00	3419.29	0.00162	A32	scncl	n	105.25	10.4	-0.00	0.00	0.00289
A32	scn	h	-31.08	1.6	-0.0059	0.0011	1.00	6.82	0.00003	A32	scn	h	-32.89	2.1	-0.00	0.00	0.00012
A32	scn	n	53.12	14.2	-0.0661	0.0130	41.78	23.36	0.00109	A32	scn	n	12.88	15.1	-0.03	0.01	0.00654
A32	scnso4	c	-40.63	5.7	0.0722	0.0040	15.08	2.75	0.00026	A32	scnso4	c	12.70	19.1	0.03	0.01	0.01007
A32	scnso4	h	-28.06	3.3	0.0090	0.0029	46.78	35.76	0.00004	A32	scnso4	h	-20.04	2.3	0.00	0.00	0.00015
A32	scnso4	n	415.57	144.9	-0.4551	0.4693	1000.00	816.22	0.00050	A32	scnso4	n	175.64	13.5	-0.01	0.01	0.00503
A32	so4	c	8.84	9.2	0.1065	0.0096	77.60	14.58	0.00021	A32	so4	c	87.46	19.9	0.04	0.01	0.01104
A32	so4	h	5.22	2.3	0.0016	0.0016	1.00	35.48	0.00006	A32	so4	h	5.72	1.6	0.00	0.00	0.00007
A32	so4	n	80.30	10.0	-0.0374	0.0113	106.33	58.66	0.00017	A32	so4	n	51.76	6.7	-0.01	0.00	0.00124
A37	cl	h	-28.48	12.0	-0.0290	0.0147	144.75	117.58	0.00017	A37	cl	h	-52.28	4.9	-0.01	0.00	0.00061
A37	cl	n	85.50	14.9	0.1583	0.0188	158.32	29.24	0.00024	A37	cl	n	218.82	24.2	0.03	0.01	0.01490
A37	clso4	h	16.85	4.4	-0.0905	0.0030	12.78	1.50	0.00017	A37	clso4	h	-39.96	23.4	-0.05	0.01	0.01455
A37	clso4	n	365.31	13.8	-0.0398	0.0083	3.29	4.02	0.00245	A37	clso4	n	348.76	14.5	-0.03	0.01	0.00561
A37	scn	c	210.85	431.5	-1.5963	0.4297	60.19	39.41	0.75473	A37	scn	c	-893.04	389.2	-0.61	0.20	4.32642
A37	scncl	c	-217.72	28.9	-1.1441	0.0193	9.73	0.63	0.00802	A37	scncl	c	-877.18	298.6	-0.65	0.13	2.37405
A37	scncl	h	-123.40	8.1	-0.1037	0.0054	10.16	2.01	0.00062	A37	scncl	h	-183.61	27.1	-0.06	0.01	0.01953
A37	scncl	n	132.65	15.7	0.4587	0.0110	13.36	1.12	0.00208	A37	scncl	n	421.59	117.5	0.24	0.05	0.36777
A37	scn	h	-98.46	4.6	-0.1034	0.0037	19.82	2.81	0.00017	A37	scn	h	-151.78	25.4	-0.06	0.01	0.01839
A37	scn	n	43.66	10.3	0.5295	0.0083	20.44	1.26	0.00086	A37	scn	n	319.22	129.3	0.30	0.07	0.47763
A37	scnso4	c	261.34	404.8	-1.9319	0.5269	146.27	72.29	0.13775	A37	scnso4	c	-1329.10	381.8	-0.40	0.20	3.33877
A37	scnso4	h	-125.98	2.8	-0.1103	0.0019	10.74	0.64	0.00007	A37	scnso4	h	-201.21	29.4	-0.05	0.01	0.02391
A37	scnso4	n	128.15	18.6	0.5306	0.0155	36.91	2.73	0.00157	A37	scnso4	n	582.38	119.2	0.17	0.05	0.39228
A37	so4	h	14.22	11.3	-0.1870	0.0125	97.04	12.30	0.00025	A37	so4	h	-126.30	31.4	-0.06	0.02	0.02749
A37	so4	n	46.44	22.9	-0.3681	0.0243	85.31	11.29	0.00117	A37	so4	n	-227.21	65.3	-0.12	0.03	0.11912
A43	cl	c	9.92	7.8	0.1081	0.0079	81.96	11.86	0.00014	A43	cl	c	99.12	21.3	0.03	0.01	0.01158
A43	cl	h	-33.80	11.1	-0.0681	0.0131	130.41	41.85	0.00017	A43	cl	h	-90.30	11.0	-0.01	0.01	0.00306
A43	cl	n	-42.50	9.2	-0.1795	0.0095	85.27	8.75	0.00019	A43	cl	n	-189.35	33.7	-0.05	0.02	0.02885

A43	clso4	c	-4.26	11.3	0.1455	0.0092	32.80	5.50	0.00064	A43	clso4	c	108.23	33.3	0.06	0.01	0.02949
A43	clso4	h	-42.49	6.3	-0.1008	0.0047	18.98	2.73	0.00028	A43	clso4	h	-111.92	24.8	-0.05	0.01	0.01632
A43	clso4	n	2.29	27.5	-0.2496	0.0232	38.75	9.02	0.00332	A43	clso4	n	-195.62	55.5	-0.09	0.02	0.08169
A43	scn	c	-38.97	4.8	0.1678	0.0041	27.19	2.25	0.00016	A43	scn	c	54.98	39.5	0.09	0.02	0.04453
A43	scncl	c	-42.74	6.9	0.1795	0.0047	11.87	1.13	0.00042	A43	scncl	c	66.92	46.6	0.10	0.02	0.05780
A43	scncl	h	-80.78	6.8	-0.1099	0.0046	9.83	1.56	0.00045	A43	scncl	h	-144.08	28.7	-0.06	0.01	0.02193
A43	scncl	n	-72.37	18.0	-0.3949	0.0119	8.95	1.06	0.00323	A43	scncl	n	-293.80	103.3	-0.23	0.04	0.28400
A43	scn	h	-57.81	2.0	-0.0917	0.0015	14.09	1.10	0.00004	A43	scn	h	-101.11	23.4	-0.06	0.01	0.01562
A43	scn	n	-66.50	6.0	-0.3754	0.0046	14.00	0.82	0.00033	A43	scn	n	-243.46	95.7	-0.23	0.05	0.26178
A43	scnso4	c	-108.22	18.2	0.2028	0.0137	22.27	4.45	0.00211	A43	scnso4	c	52.05	50.3	0.08	0.02	0.06982
A43	scnso4	h	-75.88	3.2	-0.1115	0.0022	12.02	0.81	0.00009	A43	scnso4	h	-153.84	29.4	-0.05	0.01	0.02390
A43	scnso4	n	-8.32	15.9	-0.4180	0.0116	18.28	1.58	0.00182	A43	scnso4	n	-326.73	105.1	-0.18	0.04	0.30518
A43	so4	c	-7.87	10.4	0.1029	0.0109	80.63	17.48	0.00026	A43	so4	c	67.62	18.4	0.03	0.01	0.00948
A43	so4	h	-14.33	3.4	-0.1135	0.0036	82.52	5.30	0.00003	A43	so4	h	-98.28	20.2	-0.04	0.01	0.01144
A43	so4	n	-3.95	6.4	-0.0692	0.0064	70.66	14.26	0.00011	A43	so4	n	-54.12	13.1	-0.02	0.01	0.00482
A46	cl	h	-1.02	8.1	0.0248	0.0083	83.46	54.33	0.00015	A46	cl	h	19.90	5.6	0.01	0.00	0.00081
A46	cl	n	2.45	17.1	0.1554	0.0254	237.46	51.73	0.00017	A46	cl	n	127.18	18.0	0.02	0.01	0.00822
A46	clso4	h	9.41	2.3	0.0363	0.0016	13.75	2.11	0.00004	A46	clso4	h	32.54	9.3	0.02	0.00	0.00229
A46	clso4	n	36.42	9.8	0.1552	0.0075	23.43	3.30	0.00059	A46	clso4	n	148.72	37.3	0.07	0.02	0.03699
A46	scncl	h	-28.62	3.3	0.0643	0.0023	11.72	1.49	0.00010	A46	scncl	h	10.60	16.8	0.03	0.01	0.00755
A46	scncl	n	-97.50	11.9	0.1926	0.0088	19.64	2.80	0.00097	A46	scncl	n	35.88	47.6	0.09	0.02	0.06039
A46	scn	h	-33.40	1.9	0.0835	0.0016	21.34	1.53	0.00003	A46	scn	h	10.56	20.3	0.05	0.01	0.01178
A46	scn	n	-114.46	9.9	0.2187	0.0086	30.81	3.91	0.00064	A46	scn	n	11.62	50.4	0.11	0.03	0.07262
A46	scnso4	h	-41.17	3.3	0.0884	0.0025	19.67	1.68	0.00008	A46	scnso4	h	27.50	22.3	0.04	0.01	0.01372
A46	scnso4	n	-179.45	18.0	0.2529	0.0137	22.99	3.63	0.00202	A46	scnso4	n	21.74	62.2	0.10	0.03	0.10684
A46	so4	h	16.77	2.2	0.0346	0.0018	19.96	3.93	0.00003	A46	so4	h	35.73	8.4	0.02	0.00	0.00198
A46	so4	n	57.16	14.4	0.2006	0.0137	53.39	8.88	0.00073	A46	so4	n	194.98	40.3	0.08	0.02	0.04528
A74	cl	c	-11.62	10.2	-0.0055	0.0099	69.69	265.10	0.00028	A74	cl	c	-15.76	3.4	-0.00	0.00	0.00030
A74	cl	h	-3.60	6.3	0.0038	0.0047	17.94	74.79	0.00027	A74	cl	h	-1.05	3.5	0.00	0.00	0.00030
A74	cl	n	73.44	40.9	-0.1488	0.0718	341.48	191.01	0.00051	A74	cl	n	-40.09	14.6	-0.01	0.01	0.00540
A74	clso4	c	28.63	72.1	-0.0872	0.2334	1000.00	2117.57	0.00013	A74	clso4	c	-19.04	3.8	-0.00	0.00	0.00037
A74	clso4	h	-12.54	2.7	-0.0051	0.0015	1.00	3.87	0.00011	A74	clso4	h	-13.98	2.4	-0.00	0.00	0.00016
A74	clso4	n	-74.42	19.8	-0.1026	0.0124	5.65	3.14	0.00446	A74	clso4	n	-124.26	29.7	-0.07	0.01	0.02342
A74	scn	c	-55.51	125.2	0.0329	0.4132	1000.00	10071.92	0.00059	A74	scn	c	-35.04	4.5	-0.00	0.00	0.00059
A74	scncl	c	-63.11	3.9	0.0068	0.0022	1.03	4.14	0.00022	A74	scncl	c	-61.06	3.5	0.01	0.00	0.00032
A74	scncl	h	-31.93	16.3	-0.0265	0.0201	148.38	178.34	0.00032	A74	scncl	h	-52.80	4.0	-0.01	0.00	0.00042
A74	scncl	n	-83.42	14.1	-0.1158	0.0081	1.33	0.96	0.00289	A74	scncl	n	-120.48	34.2	-0.09	0.01	0.03110
A74	scn	h	-26.83	46.1	0.0469	0.1521	1000.00	2604.58	0.00008	A74	scn	h	-2.36	2.6	0.00	0.00	0.00019
A74	scn	n	98.04	10.6	-0.0603	0.0073	2.06	4.79	0.00141	A74	scn	n	78.42	18.9	-0.05	0.01	0.01023
A74	scnso4	c	-24.32	186.8	-0.0234	0.6010	990.12	20204.52	0.00086	A74	scnso4	c	-40.68	5.3	0.00	0.00	0.00078
A74	scnso4	h	-36.87	2.6	-0.0062	0.0015	2.88	3.43	0.00009	A74	scnso4	h	-39.77	2.5	-0.00	0.00	0.00017
A74	scnso4	n	-31.01	11.6	-0.1162	0.0067	2.98	0.85	0.00189	A74	scnso4	n	-85.70	32.6	-0.08	0.01	0.02931
A74	so4	c	-27.47	5.0	0.0098	0.0039	16.82	28.17	0.00019	A74	so4	c	-22.36	3.5	0.01	0.00	0.00035
A74	so4	h	-27.36	25.8	0.0743	0.0829	999.66	876.38	0.00002	A74	so4	h	10.80	2.6	0.00	0.00	0.00019
A74	so4	n	-77.32	6.6	-0.0907	0.0045	1.46	1.85	0.00050	A74	so4	n	-107.56	27.1	-0.07	0.01	0.02057
D101	cl	c	64.86	198.6	-0.0612	0.6428	999.57	8305.15	0.00095	D101	cl	c	28.38	6.5	0.00	0.00	0.00106
D101	cl	h	-25.88	6.9	0.0100	0.0062	50.92	74.02	0.00017	D101	cl	h	-17.84	3.5	0.00	0.00	0.00032
D101	cl	n	-70.28	24.7	0.0982	0.0414	308.88	156.78	0.00023	D101	cl	n	7.10	10.7	0.01	0.00	0.00291
D101	clso4	c	69.24	6.4	-0.0304	0.0038	2.70	2.19	0.00054	D101	clso4	c	57.35	9.5	-0.02	0.00	0.00242
D101	clso4	h	-17.94	1.9	0.0124	0.0012	4.99	2.25	0.00004	D101	clso4	h	-12.07	3.6	0.01	0.00	0.00034
D101	clso4	n	-68.81	12.4	0.0534	0.0084	11.20	6.41	0.00138	D101	clso4	n	-36.37	15.7	0.03	0.01	0.00657
D101	scn	c	-29.78	2.6	-0.0231	0.0017	2.03	2.99	0.00008	D101	scn	c	-37.29	7.0	-0.02	0.00	0.00138
D101	scncl	c	-25.74	8.5	-0.0111	0.0059	12.83	24.12	0.00062	D101	scncl	c	-32.97	5.8	-0.01	0.00	0.00088
D101	scncl	h	-17.89	76.4	-0.0112	0.2473	1000.00	17396.97	0.00014	D101	scncl	h	-24.83	2.3	0.00	0.00	0.00014
D101	scncl	n	95.37	21.7	0.0550	0.0197	54.29	43.78	0.00158	D101	scncl	n	145.89	15.9	0.01	0.01	0.00677
D101	scn	h	-18.70	7.6	0.0093	0.0103	166.91	295.38	0.00008	D101	scn	h	-11.16	2.4	0.00	0.00	0.00016
D101	scn	n	84.69	14.5	0.0771	0.0169	108.29	45.38	0.00048	D101	scn	n	141.82	14.8	0.02	0.01	0.00623

D101	scnso4	c	-8.62	2.9	-0.0299	0.0017	4.98	1.23	0.00010	D101	scnso4	c	-25.04	8.3	-0.02	0.00	0.00189
D101	scnso4	h	-16.20	2.6	0.0161	0.0016	5.24	2.14	0.00008	D101	scnso4	h	-7.44	4.4	0.01	0.00	0.00054
D101	scnso4	n	124.35	13.7	0.0665	0.0090	8.98	4.48	0.00193	D101	scnso4	n	168.21	20.1	0.03	0.01	0.01119
D101	so4	c	36.30	7.9	-0.0223	0.0054	1.00	8.68	0.00072	D101	so4	c	29.09	8.4	-0.02	0.00	0.00195
D101	so4	h	-7.85	1.4	0.0091	0.0010	1.00	3.84	0.00002	D101	so4	h	-4.91	2.8	0.01	0.00	0.00023
D101	so4	n	-66.67	4.5	0.0907	0.0036	17.94	2.85	0.00015	D101	so4	n	-18.31	22.4	0.05	0.01	0.01394
D12	cl	c	-20.80	16.2	0.0065	0.0226	203.84	996.58	0.00020	D12	cl	c	-15.36	2.9	0.00	0.00	0.00022
D12	cl	h	-41.95	84.7	-0.0678	0.2742	1000.00	3196.48	0.00017	D12	cl	h	-77.60	3.5	-0.00	0.00	0.00032
D12	cl	n	-55.27	5.4	-0.0165	0.0053	72.85	48.50	0.00008	D12	cl	n	-68.38	3.5	-0.01	0.00	0.00032
D12	clso4	c	-25.14	5.0	-0.0193	0.0033	8.98	5.95	0.00024	D12	clso4	c	-36.26	6.1	-0.01	0.00	0.00097
D12	clso4	h	-74.87	5.2	-0.0576	0.0056	101.50	17.92	0.00005	D12	clso4	h	-125.84	10.1	-0.01	0.00	0.00273
D12	clso4	n	126.17	196.4	-0.3116	0.6358	1000.00	1614.41	0.00093	D12	clso4	n	-36.96	11.1	-0.01	0.00	0.00328
D12	scn	c	-56.78	2.9	-0.0036	0.0019	1.00	19.96	0.00011	D12	scn	c	-57.88	2.2	-0.00	0.00	0.00014
D12	scncl	c	-52.93	85.1	-0.0220	0.1960	572.18	4862.85	0.00074	D12	scncl	c	-71.47	5.3	0.00	0.00	0.00076
D12	scncl	h	-112.06	9.8	-0.0543	0.0110	111.59	39.63	0.00016	D12	scncl	h	-158.44	8.5	-0.01	0.00	0.00193
D12	scncl	n	59.81	6.0	-0.0420	0.0041	10.96	3.95	0.00033	D12	scncl	n	34.92	11.3	-0.02	0.00	0.00339
D12	scn	h	-92.75	5.7	-0.0354	0.0073	144.37	50.19	0.00005	D12	scn	h	-118.32	5.3	-0.01	0.00	0.00080
D12	scn	n	55.36	1.6	-0.0185	0.0012	5.51	3.00	0.00003	D12	scn	n	48.37	5.2	-0.01	0.00	0.00078
D12	scnso4	c	-14.48	17.5	-0.0661	0.0219	156.54	80.85	0.00034	D12	scnso4	c	-72.59	8.9	-0.01	0.00	0.00220
D12	scnso4	h	-131.88	3.2	-0.0499	0.0032	80.47	10.12	0.00002	D12	scnso4	h	-177.67	9.1	-0.01	0.00	0.00231
D12	scnso4	n	160.22	11.6	-0.0301	0.0093	29.23	24.49	0.00072	D12	scnso4	n	136.46	8.0	-0.01	0.00	0.00178
D12	so4	c	-44.36	6.4	-0.0182	0.0044	1.00	8.69	0.00048	D12	so4	c	-50.22	6.8	-0.01	0.00	0.00129
D12	so4	h	-33.15	7.4	-0.0981	0.0118	276.83	41.33	0.00002	D12	so4	h	-106.80	9.9	-0.01	0.01	0.00273
D12	so4	n	22.84	101.5	-0.0906	0.2719	749.03	1936.26	0.00056	D12	so4	n	-30.47	6.2	-0.00	0.00	0.00106
D22	cl	c	56.69	5.9	0.0095	0.0043	14.76	24.58	0.00026	D22	cl	c	62.11	4.0	0.01	0.00	0.00041
D22	cl	h	12.02	5.8	0.0057	0.0049	36.36	82.67	0.00015	D22	cl	h	16.28	2.8	0.00	0.00	0.00021
D22	cl	n	166.81	23.0	-0.0800	0.0382	304.59	175.88	0.00020	D22	cl	n	103.27	9.0	-0.01	0.00	0.00208
D22	clso4	c	105.64	6.7	0.0303	0.0056	37.24	17.46	0.00020	D22	clso4	c	129.35	7.1	0.01	0.00	0.00133
D22	clso4	h	-69.26	223.8	0.1431	0.7240	998.86	4000.39	0.00121	D22	clso4	h	13.83	8.4	-0.00	0.00	0.00187
D22	clso4	n	220.25	16.6	-0.0576	0.0101	4.03	3.75	0.00339	D22	clso4	n	194.50	19.5	-0.04	0.01	0.01014
D22	scn	c	-42.24	2.5	-0.0083	0.0018	5.81	10.44	0.00007	D22	scn	c	-45.42	2.8	-0.01	0.00	0.00022
D22	scncl	c	44.25	248.5	-0.1012	0.8049	1000.00	6289.90	0.00150	D22	scncl	c	-17.49	7.7	0.00	0.00	0.00159
D22	scncl	h	-50.26	4.3	-0.0076	0.0024	1.00	4.03	0.00027	D22	scncl	h	-52.41	3.7	-0.01	0.00	0.00037
D22	scncl	n	260.91	10.4	-0.0795	0.0062	3.03	1.45	0.00143	D22	scncl	n	229.07	22.8	-0.06	0.01	0.01382
D22	scn	h	-57.04	41.9	0.0366	0.1383	1000.00	3033.30	0.00007	D22	scn	h	-37.58	2.2	0.00	0.00	0.00014
D22	scn	n	129.12	19.1	-0.0469	0.0131	2.23	11.22	0.00456	D22	scn	n	113.74	18.6	-0.04	0.01	0.00985
D22	scnso4	c	-19.69	9.4	0.0101	0.0054	2.65	7.27	0.00127	D22	scnso4	c	-15.11	7.3	0.01	0.00	0.00148
D22	scnso4	h	-180.07	151.0	0.1975	0.4892	1000.00	1960.63	0.00055	D22	scnso4	h	-70.09	7.8	-0.00	0.00	0.00169
D22	scnso4	n	214.69	24.3	-0.0477	0.0143	3.54	4.97	0.00804	D22	scnso4	n	190.53	21.7	-0.03	0.01	0.01301
D22	so4	c	-36.23	46.0	0.2266	0.1480	999.29	512.79	0.00005	D22	so4	c	78.69	7.1	0.01	0.00	0.00141
D22	so4	h	-50.75	27.4	0.1508	0.0882	1000.00	459.33	0.00002	D22	so4	h	25.82	4.7	0.00	0.00	0.00062
D22	so4	n	102.93	8.6	-0.0364	0.0062	5.78	7.92	0.00075	D22	so4	n	88.17	11.3	-0.03	0.01	0.00357
D44	cl	c	-37.03	15.1	0.0643	0.0174	121.08	56.13	0.00035	D44	cl	c	16.63	11.3	0.01	0.01	0.00322
D44	cl	h	-14.47	9.3	0.0433	0.0098	89.57	38.61	0.00018	D44	cl	h	21.94	8.9	0.01	0.00	0.00204
D44	cl	n	74.30	8.5	0.1264	0.0093	106.23	14.13	0.00013	D44	cl	n	179.40	22.1	0.03	0.01	0.01247
D44	clso4	c	-38.47	8.7	0.0881	0.0070	31.60	6.73	0.00038	D44	clso4	c	28.77	20.1	0.03	0.01	0.01073
D44	clso4	h	6.54	3.4	0.0439	0.0024	17.41	3.10	0.00008	D44	clso4	h	36.20	11.0	0.02	0.00	0.00320
D44	clso4	n	141.24	9.3	0.2178	0.0065	13.72	1.40	0.00072	D44	clso4	n	280.02	55.5	0.11	0.02	0.08165
D44	scn	c	-139.58	5.3	0.0806	0.0047	34.00	6.13	0.00017	D44	scn	c	-91.96	18.4	0.04	0.01	0.00970
D44	scncl	c	-159.28	5.6	0.0853	0.0038	10.66	1.76	0.00029	D44	scncl	c	-108.60	22.6	0.05	0.01	0.01355
D44	scncl	h	-23.89	3.5	0.0670	0.0024	10.72	1.43	0.00012	D44	scncl	h	15.98	17.7	0.04	0.01	0.00829
D44	scncl	n	166.17	8.0	0.1344	0.0053	8.96	1.39	0.00063	D44	scncl	n	242.19	35.9	0.08	0.02	0.03434
D44	scn	h	-24.59	1.5	0.0732	0.0012	20.09	1.33	0.00002	D44	scn	h	13.36	18.0	0.04	0.01	0.00920
D44	scn	n	80.43	4.4	0.1258	0.0035	20.94	2.27	0.00015	D44	scn	n	146.47	30.8	0.07	0.02	0.02716
D44	scnso4	c	-161.65	4.9	0.0874	0.0034	13.40	1.75	0.00020	D44	scnso4	c	-99.05	22.9	0.04	0.01	0.01455
D44	scnso4	h	-22.34	4.2	0.0804	0.0030	16.23	1.95	0.00014	D44	scnso4	h	37.50	20.6	0.04	0.01	0.01170
D44	scnso4	n	156.53	8.5	0.1830	0.0054	7.84	0.89	0.00078	D44	scnso4	n	271.73	49.7	0.10	0.02	0.06830

D44	so4	c	-27.78	17.8	0.0779	0.0220	141.99	65.19	0.00039	D44	so4	c	32.00	11.6	0.02	0.01	0.00376
D44	so4	h	6.58	2.9	0.0230	0.0022	14.61	6.30	0.00006	D44	so4	h	18.22	6.0	0.01	0.00	0.00100
D44	so4	n	93.57	14.6	0.2965	0.0133	43.38	5.18	0.00088	D44	so4	n	289.13	62.4	0.13	0.03	0.10866
D54	cl	c	3.96	65.4	-0.1787	0.2117	999.54	936.66	0.00010	D54	cl	c	-88.75	6.3	-0.00	0.00	0.00100
D54	cl	h	-8.24	12.4	0.0744	0.0164	180.70	58.91	0.00014	D54	cl	h	53.86	10.7	0.01	0.00	0.00292
D54	cl	n	-79.91	46.1	0.4706	0.0851	377.09	76.00	0.00054	D54	cl	n	268.48	39.6	0.04	0.02	0.03983
D54	clso4	c	-61.41	7.7	-0.0601	0.0055	16.33	4.89	0.00045	D54	clso4	c	-101.62	15.7	-0.03	0.01	0.00653
D54	clso4	h	16.97	2.8	0.0977	0.0017	5.48	0.45	0.00009	D54	clso4	h	64.43	26.1	0.06	0.01	0.01804
D54	clso4	n	94.65	14.8	0.3169	0.0096	8.11	0.99	0.00223	D54	clso4	n	268.49	83.4	0.19	0.04	0.18485
D54	scn	c	-89.24	3.7	-0.0610	0.0033	41.17	6.45	0.00007	D54	scn	c	-126.84	13.4	-0.03	0.01	0.00514
D54	scncl	c	-106.22	9.9	-0.0623	0.0073	19.34	7.11	0.00068	D54	scncl	c	-149.36	16.2	-0.03	0.01	0.00702
D54	scncl	h	-16.78	10.8	0.0668	0.0098	54.92	18.11	0.00039	D54	scncl	h	38.62	14.0	0.02	0.01	0.00521
D54	scncl	n	17.18	29.9	0.2699	0.0249	36.39	8.61	0.00411	D54	scncl	n	228.08	60.9	0.10	0.03	0.09882
D54	scn	h	-27.41	4.6	0.0742	0.0060	148.68	20.12	0.00003	D54	scn	h	27.32	11.4	0.02	0.01	0.00371
D54	scn	n	-12.63	14.3	0.2532	0.0153	78.73	10.36	0.00065	D54	scn	n	162.77	47.6	0.09	0.02	0.06465
D54	scnso4	c	-80.85	5.4	-0.0905	0.0043	28.61	3.71	0.00016	D54	scnso4	c	-156.10	21.8	-0.03	0.01	0.01315
D54	scnso4	h	-48.83	7.0	0.0893	0.0045	7.75	1.48	0.00053	D54	scnso4	h	6.73	24.1	0.05	0.01	0.01606
D54	scnso4	n	-56.02	18.8	0.3101	0.0127	11.37	1.63	0.00327	D54	scnso4	n	156.46	81.4	0.15	0.03	0.18315
D54	so4	c	-56.67	6.3	-0.0642	0.0049	15.74	5.19	0.00030	D54	so4	c	-89.69	16.3	-0.04	0.01	0.00739
D54	so4	h	25.82	2.8	0.1093	0.0021	7.98	0.98	0.00008	D54	so4	h	73.31	29.3	0.07	0.02	0.02401
D54	so4	n	78.35	11.6	0.3483	0.0089	14.23	1.66	0.00107	D54	so4	n	253.21	88.1	0.21	0.05	0.21684
D75	cl	c	30.27	7.0	0.0482	0.0056	28.01	9.38	0.00026	D75	cl	c	62.78	11.8	0.02	0.01	0.00354
D75	cl	h	-20.67	7.5	0.0186	0.0072	65.85	54.45	0.00016	D75	cl	h	-5.19	4.9	0.01	0.00	0.00061
D75	cl	n	-42.67	7.9	0.0348	0.0109	198.20	88.51	0.00005	D75	cl	n	-14.39	4.6	0.01	0.00	0.00053
D75	clso4	c	7.26	6.4	0.1053	0.0043	10.66	1.60	0.00037	D75	clso4	c	69.88	27.4	0.06	0.01	0.01999
D75	clso4	h	-1.89	1.5	0.0216	0.0010	13.47	2.25	0.00002	D75	clso4	h	11.71	5.5	0.01	0.00	0.00080
D75	clso4	n	-6.65	6.7	0.0238	0.0038	1.00	2.03	0.00065	D75	clso4	n	0.51	8.4	0.02	0.00	0.00187
D75	scn	c	-27.03	3.9	0.1040	0.0038	54.69	5.06	0.00007	D75	scn	c	41.24	21.8	0.04	0.01	0.01353
D75	scncl	c	-39.93	5.3	0.1064	0.0041	26.87	2.94	0.00016	D75	scncl	c	39.89	25.9	0.04	0.01	0.01784
D75	scncl	h	-62.59	4.0	0.0050	0.0024	3.32	9.26	0.00020	D75	scncl	h	-60.48	3.1	0.00	0.00	0.00026
D75	scncl	n	-73.48	2.5	0.0273	0.0015	5.26	1.40	0.00007	D75	scncl	n	-60.44	7.5	0.02	0.00	0.00149
D75	scn	h	-46.32	32.8	-0.0121	0.1083	1000.00	7186.65	0.00004	D75	scn	h	-52.88	1.3	0.00	0.00	0.00005
D75	scn	n	-82.01	4.6	0.0410	0.0037	20.27	7.23	0.00017	D75	scn	n	-60.56	10.5	0.02	0.01	0.00312
D75	scnso4	c	-86.51	24.2	0.1354	0.0171	14.92	6.16	0.00471	D75	scnso4	c	11.25	36.2	0.06	0.01	0.03616
D75	scnso4	h	-57.70	1.9	0.0100	0.0011	5.02	2.45	0.00004	D75	scnso4	h	-52.04	3.1	0.01	0.00	0.00027
D75	scnso4	n	-63.17	6.0	0.0479	0.0038	7.12	2.21	0.00040	D75	scnso4	n	-33.59	13.7	0.03	0.01	0.00519
D75	so4	c	22.58	5.1	0.1082	0.0040	16.08	2.50	0.00019	D75	so4	c	78.56	26.9	0.06	0.01	0.02026
D75	so4	h	-1.38	1.5	0.0115	0.0011	6.94	4.75	0.00002	D75	so4	h	3.44	3.2	0.01	0.00	0.00029
D75	so4	n	-9.94	6.4	0.0423	0.0044	1.00	3.76	0.00048	D75	so4	n	3.75	13.3	0.03	0.01	0.00490
D86	cl	c	39.10	161.4	-0.2649	0.5218	998.03	1555.17	0.00063	D86	cl	c	-101.95	11.1	-0.00	0.01	0.00314
D86	cl	h	-16.33	8.8	0.0439	0.0092	89.42	35.69	0.00016	D86	cl	h	20.31	8.9	0.01	0.00	0.00199
D86	cl	n	102.39	13.7	0.0859	0.0183	184.25	57.49	0.00016	D86	cl	n	172.96	11.6	0.01	0.01	0.00345
D86	clso4	c	-58.05	7.0	-0.0964	0.0047	11.19	2.00	0.00044	D86	clso4	c	-116.18	25.2	-0.05	0.01	0.01681
D86	clso4	h	-17.17	2.2	0.0645	0.0016	13.47	1.12	0.00004	D86	clso4	h	23.89	16.6	0.03	0.01	0.00729
D86	clso4	n	174.64	19.8	0.1208	0.0178	51.61	17.38	0.00137	D86	clso4	n	272.97	24.6	0.04	0.01	0.01612
D86	scn	c	-119.20	5.2	-0.0677	0.0044	27.53	6.04	0.00018	D86	scn	c	-157.14	16.0	-0.04	0.01	0.00730
D86	scncl	c	-133.18	15.7	-0.1317	0.0136	44.78	11.11	0.00097	D86	scncl	c	-238.95	28.1	-0.05	0.01	0.02104
D86	scncl	h	-35.83	3.7	0.0716	0.0026	12.75	1.62	0.00012	D86	scncl	h	8.88	18.7	0.04	0.01	0.00927
D86	scncl	n	285.10	7.2	0.0169	0.0046	7.67	8.75	0.00054	D86	scncl	n	294.13	6.3	0.01	0.00	0.00106
D86	scn	h	-30.14	1.5	0.0620	0.0012	23.21	1.65	0.00002	D86	scn	h	3.26	14.9	0.03	0.01	0.00638
D86	scn	n	206.11	7.3	0.0816	0.0075	70.32	14.78	0.00019	D86	scn	n	261.85	16.1	0.03	0.01	0.00736
D86	scnso4	c	-52.14	20.4	-0.1548	0.0121	20.83	4.50	0.00124	D86	scnso4	c	-215.79	54.1	-0.06	0.02	0.04028
D86	scnso4	h	-68.81	3.2	0.0951	0.0022	14.60	1.13	0.00008	D86	scnso4	h	0.56	24.6	0.04	0.01	0.01677
D86	scnso4	n	379.06	6.7	0.0527	0.0044	8.48	2.63	0.00047	D86	scnso4	n	412.32	14.3	0.03	0.01	0.00562
D86	so4	c	-47.97	8.9	-0.0953	0.0071	19.08	5.50	0.00054	D86	so4	c	-99.61	23.6	-0.05	0.01	0.01553
D86	so4	h	4.47	2.6	0.0543	0.0021	21.96	3.07	0.00004	D86	so4	h	35.01	13.0	0.03	0.01	0.00472
D86	so4	n	58.00	19.8	0.1498	0.0217	95.43	26.50	0.00078	D86	so4	n	169.37	25.0	0.05	0.01	0.01747

D8	cl	c	154.74	23.1	0.0744	0.0312	187.58	114.69	0.00045	D8	cl	c	214.15	9.6	0.01	0.00	0.00236
D8	cl	h	-11.39	23.5	0.0291	0.0435	378.30	628.44	0.00014	D8	cl	h	11.18	3.7	0.00	0.00	0.00034
D8	cl	n	238.95	6.1	-0.0041	0.0040	5.81	32.89	0.00038	D8	cl	n	237.10	4.0	-0.00	0.00	0.00042
D8	clso4	c	242.67	19.9	0.1909	0.0208	90.50	18.66	0.00083	D8	clso4	c	408.22	33.5	0.04	0.01	0.02980
D8	clso4	h	-65.13	15.1	0.1924	0.0433	828.82	156.56	0.00001	D8	clso4	h	45.74	7.4	0.01	0.00	0.00146
D8	clso4	n	340.77	260.4	0.1720	0.8433	1000.00	3879.67	0.00163	D8	clso4	n	439.64	9.8	-0.00	0.00	0.00254
D8	scn	c	-10.89	15.3	0.2363	0.0309	425.02	60.49	0.00007	D8	scn	c	146.09	18.8	0.02	0.01	0.01011
D8	scncl	c	101.99	17.0	0.1232	0.0172	82.00	22.39	0.00067	D8	scncl	c	208.18	22.8	0.03	0.01	0.01378
D8	scncl	h	-141.05	73.6	0.0977	0.2381	998.57	1925.79	0.00013	D8	scncl	h	-88.75	4.0	0.00	0.00	0.00043
D8	scncl	n	283.07	22.7	-0.0744	0.0217	66.02	40.51	0.00147	D8	scncl	n	223.32	13.9	-0.02	0.01	0.00513
D8	scn	h	-106.49	34.3	0.0529	0.1132	999.50	1716.25	0.00004	D8	scn	h	-79.66	2.5	0.00	0.00	0.00017
D8	scn	n	148.40	18.9	-0.1269	0.0256	166.76	53.57	0.00048	D8	scn	n	56.29	17.8	-0.03	0.01	0.00906
D8	scnso4	c	78.89	14.2	0.2179	0.0160	114.58	14.65	0.00033	D8	scnso4	c	278.44	34.4	0.04	0.01	0.03271
D8	scnso4	h	-247.96	71.9	0.2923	0.2330	999.72	630.68	0.00012	D8	scnso4	h	-93.42	8.6	0.01	0.00	0.00206
D8	scnso4	n	303.08	56.0	-0.1745	0.1004	357.06	234.34	0.00088	D8	scnso4	n	170.66	12.7	-0.02	0.01	0.00448
D8	so4	c	191.33	19.3	0.2372	0.0279	219.60	35.26	0.00025	D8	so4	c	373.07	27.4	0.04	0.01	0.02101
D8	so4	h	-7.40	47.2	0.1709	0.1514	994.52	693.04	0.00006	D8	so4	h	78.42	4.9	0.01	0.00	0.00068
D8	so4	n	221.09	164.1	0.0913	0.5282	1000.00	4543.01	0.00068	D8	so4	n	270.19	6.0	0.00	0.00	0.00102
D93	cl	c	36.48	10.1	0.0386	0.0159	271.58	141.23	0.00005	D93	cl	c	67.65	4.6	0.00	0.00	0.00055
D93	cl	h	-20.34	7.2	0.0118	0.0068	62.56	78.24	0.00015	D93	cl	h	-10.52	3.7	0.00	0.00	0.00034
D93	cl	n	-12.21	101.7	-0.0587	0.3291	999.53	4428.10	0.00025	D93	cl	n	-43.73	3.9	-0.00	0.00	0.00038
D93	clso4	c	99.34	7.4	0.0157	0.0057	26.06	26.95	0.00032	D93	clso4	c	111.85	5.5	0.01	0.00	0.00081
D93	clso4	h	-9.15	2.3	0.0149	0.0016	11.02	4.20	0.00005	D93	clso4	h	-0.10	4.2	0.01	0.00	0.00046
D93	clso4	n	-46.97	28.4	-0.0938	0.0359	159.22	94.33	0.00086	D93	clso4	n	-126.15	12.9	-0.02	0.01	0.00444
D93	scn	c	-7.59	9.6	0.0820	0.0150	245.01	60.91	0.00007	D93	scn	c	52.37	10.0	0.01	0.01	0.00286
D93	scncl	c	58.38	4.5	0.0334	0.0031	13.67	4.45	0.00017	D93	scncl	c	79.79	9.1	0.02	0.00	0.00220
D93	scncl	h	2.63	6.7	0.0193	0.0056	36.25	26.97	0.00021	D93	scncl	h	17.95	5.3	0.01	0.00	0.00074
D93	scncl	n	199.68	51.5	-0.2434	0.0898	337.45	144.99	0.00084	D93	scncl	n	13.02	20.3	-0.02	0.01	0.01092
D93	scn	h	13.08	4.4	0.0310	0.0047	74.19	24.91	0.00007	D93	scn	h	34.72	6.3	0.01	0.00	0.00115
D93	scn	n	-25.28	10.0	-0.0323	0.0080	18.70	18.89	0.00084	D93	scn	n	-41.62	9.6	-0.02	0.00	0.00261
D93	scnso4	c	57.52	14.5	0.0410	0.0106	50.99	27.21	0.00030	D93	scnso4	c	108.58	13.9	0.01	0.00	0.00264
D93	scnso4	h	53.16	1.1	0.0184	0.0007	10.76	1.50	0.00001	D93	scnso4	h	65.75	5.0	0.01	0.00	0.00068
D93	scnso4	n	-74.40	13.3	-0.0588	0.0115	43.13	20.33	0.00072	D93	scnso4	n	-126.36	14.1	-0.02	0.01	0.00548
D93	so4	c	-15.51	99.5	0.1511	0.3201	1000.00	1664.55	0.00025	D93	so4	c	63.87	6.2	0.00	0.00	0.00108
D93	so4	h	-3.04	1.6	0.0157	0.0011	5.62	3.38	0.00003	D93	so4	h	3.30	4.5	0.01	0.00	0.00055
D93	so4	n	-112.84	13.7	-0.0802	0.0198	217.52	73.50	0.00013	D93	so4	n	-175.19	10.0	-0.01	0.01	0.00279
E29	cl	c	16.30	14.5	0.0061	0.0184	160.30	748.63	0.00022	E29	cl	c	21.25	3.1	0.00	0.00	0.00024
E29	cl	h	-24.96	9.4	0.0154	0.0099	91.67	111.60	0.00018	E29	cl	h	-11.59	4.2	0.00	0.00	0.00045
E29	cl	n	46.93	5.5	0.0047	0.0042	20.33	59.06	0.00019	E29	cl	n	50.05	3.1	0.00	0.00	0.00024
E29	clso4	c	-49.44	25.9	0.0806	0.0366	211.78	133.86	0.00047	E29	clso4	c	16.06	9.0	0.01	0.00	0.00213
E29	clso4	h	-32.68	79.7	0.0427	0.2580	1000.00	4782.95	0.00015	E29	clso4	h	-7.31	2.7	-0.00	0.00	0.00019
E29	clso4	n	132.19	8.5	-0.0199	0.0048	1.00	3.06	0.00105	E29	clso4	n	126.33	8.3	-0.02	0.00	0.00185
E29	scn	c	-108.98	13.1	0.0768	0.0207	248.08	90.36	0.00013	E29	scn	c	-53.87	8.9	0.01	0.00	0.00225
E29	scncl	c	-72.29	8.7	0.0217	0.0060	13.28	12.86	0.00063	E29	scncl	c	-58.99	7.1	0.01	0.00	0.00134
E29	scncl	h	-66.45	7.9	-0.0085	0.0077	71.57	133.80	0.00017	E29	scncl	h	-72.97	2.7	-0.00	0.00	0.00019
E29	scncl	n	178.08	9.3	-0.0746	0.0070	22.42	6.31	0.00054	E29	scncl	n	125.27	18.2	-0.03	0.01	0.00882
E29	scn	h	-57.31	1.3	-0.0072	0.0009	1.00	4.40	0.00002	E29	scn	h	-59.51	2.3	-0.01	0.00	0.00015
E29	scn	n	100.46	4.9	-0.0744	0.0041	25.80	5.03	0.00017	E29	scn	n	59.32	17.8	-0.04	0.01	0.00905
E29	scnso4	c	-171.26	19.4	0.0743	0.0201	88.61	45.68	0.00081	E29	scnso4	c	-104.71	13.2	0.02	0.01	0.00484
E29	scnso4	h	-70.20	2.9	-0.0033	0.0016	1.00	3.71	0.00014	E29	scnso4	h	-71.22	2.4	-0.00	0.00	0.00016
E29	scnso4	n	180.49	5.4	-0.0441	0.0034	6.30	1.94	0.00034	E29	scnso4	n	155.05	11.8	-0.03	0.00	0.00386
E29	so4	c	-5.47	18.4	0.0480	0.0239	162.58	124.70	0.00035	E29	so4	c	32.24	7.7	0.01	0.00	0.00166
E29	so4	h	9.13	5.9	-0.0136	0.0066	100.87	90.90	0.00006	E29	so4	h	-1.39	2.8	-0.00	0.00	0.00023
E29	so4	n	49.56	8.2	-0.0253	0.0060	8.62	12.64	0.00062	E29	so4	n	38.34	8.2	-0.02	0.00	0.00190
E60	cl	c	66.32	2.9	0.0023	0.0018	1.00	16.06	0.00011	E60	cl	c	67.02	2.2	0.00	0.00	0.00012
E60	cl	h	1.62	81.4	-0.0167	0.2636	1000.00	12465.37	0.00016	E60	cl	h	-7.58	2.6	-0.00	0.00	0.00017
E60	cl	n	17.81	124.5	0.0711	0.4031	1000.00	4480.93	0.00037	E60	cl	n	58.01	4.7	-0.00	0.00	0.00057

E60	clso4	c	13.38	112.8	0.2552	0.3651	999.54	1131.25	0.00031	E60	clso4	c	147.52	8.7	0.01	0.00	0.00203
E60	clso4	h	8.57	2.7	-0.0171	0.0016	2.60	1.64	0.00010	E60	clso4	h	1.99	5.1	-0.01	0.00	0.00068
E60	clso4	n	101.72	5.1	-0.0496	0.0034	9.03	2.39	0.00026	E60	clso4	n	73.58	13.3	-0.03	0.01	0.00472
E60	scn	c	92.02	6.0	-0.0048	0.0041	1.00	31.44	0.00047	E60	scn	c	90.64	4.3	-0.00	0.00	0.00052
E60	scncl	c	42.73	254.3	0.0884	0.8236	1000.00	7370.79	0.00157	E60	scncl	c	95.89	8.0	-0.00	0.00	0.00170
E60	scncl	h	-88.34	12.9	-0.0898	0.0117	53.87	15.82	0.00056	E60	scncl	h	-161.71	18.1	-0.03	0.01	0.00869
E60	scncl	n	-67.70	259.9	0.3126	0.8417	1000.00	2130.69	0.00164	E60	scncl	n	104.43	13.6	0.00	0.01	0.00491
E60	scn	h	-53.85	14.4	-0.1299	0.0172	115.99	28.45	0.00044	E60	scn	h	-147.15	21.3	-0.04	0.01	0.01301
E60	scn	n	51.84	74.5	0.0779	0.1033	177.99	365.78	0.00679	E60	scn	n	112.74	20.0	0.01	0.01	0.01142
E60	scnso4	c	27.86	137.1	0.2350	0.4439	999.54	1494.84	0.00045	E60	scnso4	c	150.26	7.4	0.01	0.00	0.00152
E60	scnso4	h	-131.16	7.5	-0.0649	0.0061	31.73	7.89	0.00029	E60	scnso4	h	-184.42	14.5	-0.02	0.01	0.00584
E60	scnso4	n	659.39	1218.2	-1.0117	3.9458	1000.00	3086.96	0.03559	E60	scnso4	n	130.47	44.8	-0.02	0.02	0.05548
E60	so4	c	95.30	219.7	0.0620	0.7072	1000.00	8963.07	0.00122	E60	so4	c	130.85	7.0	-0.00	0.00	0.00137
E60	so4	h	18.06	3.3	-0.0168	0.0025	13.81	9.66	0.00009	E60	so4	h	9.70	4.6	-0.01	0.00	0.00059
E60	so4	n	-28.60	4.8	-0.0580	0.0040	25.98	6.01	0.00014	E60	so4	n	-62.63	13.7	-0.03	0.01	0.00520
E73	cl	c	12.27	5.8	0.0072	0.0044	19.78	39.66	0.00022	E73	cl	c	16.99	3.6	0.00	0.00	0.00032
E73	cl	h	-13.67	8.1	0.0096	0.0073	52.02	91.51	0.00022	E73	cl	h	-5.72	3.8	0.00	0.00	0.00037
E73	cl	n	-23.32	5.2	0.0350	0.0048	57.57	17.67	0.00008	E73	cl	n	3.99	7.5	0.01	0.00	0.00144
E73	clso4	c	-55.94	194.2	0.1262	0.6288	1000.00	3942.38	0.00091	E73	clso4	c	15.04	7.4	-0.00	0.00	0.00144
E73	clso4	h	2.00	1.8	0.0136	0.0012	7.45	2.65	0.00003	E73	clso4	h	9.34	3.8	0.01	0.00	0.00039
E73	clso4	n	16.18	4.4	0.0645	0.0026	3.35	0.80	0.00025	E73	clso4	n	43.02	17.8	0.05	0.01	0.00839
E73	scn	c	-29.81	35.9	0.0708	0.0759	464.22	523.39	0.00030	E73	scn	c	18.29	6.9	0.01	0.00	0.00136
E73	scncl	c	-10.86	9.2	0.0205	0.0075	32.88	31.96	0.00042	E73	scncl	c	5.70	6.6	0.01	0.00	0.00115
E73	scncl	h	-32.75	4.4	0.0073	0.0027	4.76	8.84	0.00024	E73	scncl	h	-29.29	3.6	0.00	0.00	0.00035
E73	scncl	n	-73.67	9.3	0.0274	0.0055	3.19	3.87	0.00112	E73	scncl	n	-62.40	10.0	0.02	0.00	0.00264
E73	scn	h	-31.59	1.9	0.0089	0.0016	23.91	15.30	0.00003	E73	scn	h	-26.71	2.4	0.00	0.00	0.00016
E73	scn	n	-99.43	5.1	0.0343	0.0041	18.30	8.96	0.00022	E73	scn	n	-81.95	9.1	0.02	0.00	0.00235
E73	scnso4	c	-147.82	225.2	0.3183	0.7293	999.83	1813.38	0.00122	E73	scnso4	c	17.30	10.3	0.01	0.00	0.00294
E73	scnso4	h	-36.20	2.0	0.0202	0.0013	9.22	2.21	0.00004	E73	scnso4	h	-22.85	5.6	0.01	0.00	0.00088
E73	scnso4	n	-77.97	7.9	0.0497	0.0044	1.99	1.02	0.00096	E73	scnso4	n	-57.63	14.5	0.04	0.01	0.00581
E73	so4	c	-11.77	312.0	0.0405	1.0043	1000.00	19483.42	0.00247	E73	so4	c	18.96	8.4	-0.01	0.00	0.00197
E73	so4	h	8.12	1.9	0.0116	0.0013	2.49	4.56	0.00004	E73	so4	h	12.22	3.6	0.01	0.00	0.00036
E73	so4	n	25.03	4.1	0.0670	0.0028	2.37	1.65	0.00018	E73	so4	n	48.48	19.6	0.05	0.01	0.01075
F106	cl	c	-37.20	4.5	0.0116	0.0027	1.26	5.10	0.00025	F106	cl	c	-33.47	4.8	0.01	0.00	0.00058
F106	cl	h	-39.03	7.8	0.0112	0.0079	82.02	114.10	0.00014	F106	cl	h	-29.28	3.4	0.00	0.00	0.00030
F106	cl	n	51.47	46.4	0.4162	0.1209	708.68	182.19	0.00013	F106	cl	n	304.31	20.3	0.02	0.01	0.01045
F106	clso4	c	-38.08	7.9	0.0108	0.0045	1.00	5.31	0.00092	F106	clso4	c	-35.03	6.5	0.01	0.00	0.00113
F106	clso4	h	-49.90	16.6	0.0236	0.0331	441.24	650.98	0.00005	F106	clso4	h	-31.94	2.3	0.00	0.00	0.00015
F106	clso4	n	26.62	581.9	0.6255	1.8843	1000.00	2383.30	0.00815	F106	clso4	n	372.86	28.3	0.00	0.01	0.02128
F106	scn	c	-80.35	136.8	-0.0411	0.4515	1000.00	8814.28	0.00070	F106	scn	c	-103.00	5.3	0.00	0.00	0.00079
F106	scncl	c	-134.29	12.9	0.0125	0.0073	1.00	7.49	0.00246	F106	scncl	c	-130.77	10.2	0.01	0.00	0.00274
F106	scncl	h	-191.19	75.0	-0.1794	0.2429	999.62	1071.20	0.00014	F106	scncl	h	-284.46	5.7	-0.00	0.00	0.00086
F106	scncl	n	-283.10	348.8	1.0351	1.1295	1000.00	863.39	0.00295	F106	scncl	n	269.34	36.0	0.02	0.02	0.03444
F106	scn	h	-211.33	21.1	-0.0647	0.0408	387.61	275.35	0.00015	F106	scn	h	-253.94	5.3	-0.01	0.00	0.00079
F106	scn	n	8.19	383.6	0.3712	1.2653	999.01	2733.43	0.00553	F106	scn	n	202.96	21.3	0.01	0.01	0.01299
F106	scnso4	c	-137.52	10.1	0.0140	0.0054	1.00	3.07	0.00169	F106	scnso4	c	-132.93	8.7	0.01	0.00	0.00207
F106	scnso4	h	-271.09	53.4	-0.1080	0.1726	997.43	1262.37	0.00007	F106	scnso4	h	-328.79	3.6	-0.00	0.00	0.00037
F106	scnso4	n	146.55	244.2	0.1645	0.5428	537.55	1734.33	0.00699	F106	scnso4	n	277.20	18.9	-0.00	0.01	0.00991
F106	so4	c	-26.18	4.5	0.0144	0.0034	12.17	14.41	0.00017	F106	so4	c	-19.29	4.4	0.01	0.00	0.00054
F106	so4	h	-0.97	2.6	0.0065	0.0018	1.00	10.05	0.00008	F106	so4	h	1.11	2.6	0.00	0.00	0.00018
F106	so4	n	-125.29	209.6	0.4987	0.6743	998.91	1061.49	0.00112	F106	so4	n	133.20	18.3	0.01	0.01	0.00932
F56	cl	h	-6.91	76.0	-0.0771	0.2460	999.49	2522.82	0.00014	F56	cl	h	-46.47	3.4	-0.00	0.00	0.00029
F56	cl	n	-11.35	6.4	-0.1144	0.0100	270.61	30.06	0.00002	F56	cl	n	-102.04	12.4	-0.01	0.01	0.00389
F56	clso4	c	-68.55	265.4	-0.0591	0.8592	1000.00	11505.06	0.00170	F56	clso4	c	-109.47	6.9	0.01	0.00	0.00125
F56	clso4	h	-16.36	2.2	-0.0370	0.0014	10.39	1.51	0.00004	F56	clso4	h	-38.14	9.6	-0.02	0.00	0.00244
F56	clso4	n	-11.13	5.7	-0.1648	0.0036	6.10	0.59	0.00036	F56	clso4	n	-94.03	44.0	-0.10	0.02	0.05133
F56	scncl	c	38.03	221.7	-0.1049	0.7177	999.70	5411.21	0.00119	F56	scncl	c	-23.06	7.5	0.00	0.00	0.00151

F56	scncl	h	-54.31	8.2	-0.0350	0.0081	76.05	35.46	0.00017	F56	scncl	h	-83.90	6.7	-0.01	0.00	0.00118
F56	scncl	n	-172.94	12.5	-0.1211	0.0097	26.39	6.02	0.00089	F56	scncl	n	-261.67	28.5	-0.05	0.01	0.02168
F56	scn	h	-49.06	4.7	-0.0210	0.0059	131.31	65.17	0.00004	F56	scn	h	-64.19	3.4	-0.01	0.00	0.00033
F56	scn	n	-155.20	5.0	-0.0985	0.0045	39.35	5.21	0.00014	F56	scn	n	-215.35	21.8	-0.05	0.01	0.01360
F56	scnso4	c	-0.97	9.4	0.0153	0.0050	1.00	2.63	0.00148	F56	scnso4	c	3.72	8.2	0.01	0.00	0.00184
F56	scnso4	h	-40.68	2.3	-0.0353	0.0015	8.18	1.29	0.00006	F56	scnso4	h	-62.91	9.4	-0.02	0.00	0.00244
F56	scnso4	n	-69.07	8.5	-0.2050	0.0055	8.35	0.84	0.00076	F56	scnso4	n	-198.75	54.2	-0.11	0.02	0.08104
F56	so4	h	-9.60	2.5	-0.0395	0.0021	25.42	4.58	0.00004	F56	so4	h	-32.59	9.2	-0.02	0.00	0.00238
F56	so4	n	10.82	7.8	-0.1526	0.0059	11.94	2.31	0.00051	F56	so4	n	-62.39	39.5	-0.10	0.02	0.04360
F7	cl	c	-57.56	12.2	0.0422	0.0117	65.10	38.63	0.00043	F7	cl	c	-23.83	9.6	0.01	0.00	0.00235
F7	cl	h	-49.29	8.3	0.0119	0.0084	81.34	113.82	0.00016	F7	cl	h	-39.17	3.6	0.00	0.00	0.00032
F7	cl	n	13.77	64.1	0.2173	0.1964	918.00	673.60	0.00013	F7	cl	n	130.41	7.8	0.01	0.00	0.00156
F7	clso4	c	-27.63	135.1	0.0648	0.4352	991.60	5283.30	0.00045	F7	clso4	c	8.14	4.7	0.00	0.00	0.00059
F7	clso4	h	-127.75	58.1	0.2115	0.1879	999.53	702.86	0.00008	F7	clso4	h	-15.35	7.3	0.00	0.00	0.00140
F7	clso4	n	-22.05	81.3	0.3781	0.1661	460.90	209.36	0.00109	F7	clso4	n	249.29	26.0	0.02	0.01	0.01792
F7	scn	c	-321.73	523.3	-0.0500	1.7272	1000.00	27720.28	0.01027	F7	scn	c	-353.65	18.9	0.00	0.01	0.01021
F7	scncl	c	-204.96	167.0	-0.2885	0.5407	999.89	1482.83	0.00068	F7	scncl	c	-351.57	8.3	-0.01	0.00	0.00183
F7	scncl	h	-162.16	3.0	-0.0020	0.0017	1.00	10.51	0.00013	F7	scncl	h	-162.74	2.3	-0.00	0.00	0.00014
F7	scncl	n	-73.83	176.0	0.1390	0.5701	1000.00	3244.10	0.00075	F7	scncl	n	2.68	7.3	0.00	0.00	0.00140
F7	scn	h	-120.67	1.5	-0.0014	0.0010	1.00	27.50	0.00003	F7	scn	h	-121.08	1.1	-0.00	0.00	0.00003
F7	scn	n	-51.37	4.0	-0.0045	0.0027	1.00	22.48	0.00021	F7	scn	n	-52.70	3.0	-0.00	0.00	0.00025
F7	scnso4	c	-354.37	157.3	-0.0984	0.4004	680.15	2486.20	0.00165	F7	scnso4	c	-420.06	9.2	-0.00	0.00	0.00234
F7	scnso4	h	-294.20	32.0	0.2777	0.1037	999.76	295.35	0.00002	F7	scnso4	h	-146.99	8.1	0.01	0.00	0.00182
F7	scnso4	n	-143.84	17.3	0.1499	0.0219	160.58	36.17	0.00032	F7	scnso4	n	-10.30	19.7	0.02	0.01	0.01073
F7	so4	c	50.10	8.4	-0.0044	0.0057	1.00	47.32	0.00081	F7	so4	c	48.80	5.5	-0.00	0.00	0.00085
F7	so4	h	16.19	51.5	0.1018	0.1657	1000.00	1279.10	0.00007	F7	so4	h	68.26	3.6	0.00	0.00	0.00037
F7	so4	n	126.58	36.6	0.2096	0.0659	360.17	127.25	0.00038	F7	so4	n	275.82	17.0	0.02	0.01	0.00804
F82	cl	c	-40.84	110.7	0.2801	0.1725	264.85	208.40	0.00596	F82	cl	c	192.27	38.4	0.03	0.02	0.03749
F82	cl	h	-59.65	23.3	-0.1054	0.0283	142.73	61.93	0.00067	F82	cl	h	-146.83	16.5	-0.02	0.01	0.00692
F82	cl	n	68.22	16.0	-0.3776	0.0189	131.60	10.97	0.00035	F82	cl	n	-248.39	61.0	-0.08	0.03	0.09479
F82	clso4	c	86.45	12.9	0.2652	0.0093	8.35	1.08	0.00093	F82	clso4	c	301.01	89.2	0.10	0.05	0.17567
F82	clso4	h	-34.91	6.7	-0.1705	0.0048	15.71	1.44	0.00034	F82	clso4	h	-147.57	43.0	-0.08	0.02	0.04909
F82	clso4	n	112.72	36.2	-0.4288	0.0305	38.28	6.85	0.00582	F82	clso4	n	-226.56	94.7	-0.16	0.04	0.23808
F82	scn	c	-69.36	30.3	0.1144	0.0646	470.27	277.79	0.00021	F82	scn	c	5.19	9.0	0.01	0.00	0.00230
F82	scncl	c	2.18	4.9	0.1130	0.0032	8.90	1.01	0.00024	F82	scncl	c	65.61	29.7	0.07	0.01	0.02351
F82	scncl	h	-190.43	8.6	-0.1796	0.0058	10.56	1.27	0.00068	F82	scncl	h	-296.10	46.6	-0.10	0.02	0.05772
F82	scncl	n	52.36	48.8	-1.0132	0.0329	10.56	1.28	0.02217	F82	scncl	n	-543.91	262.8	-0.57	0.11	1.83859
F82	scn	h	14.83	1.1	-0.0497	0.0007	2.51	0.60	0.00001	F82	scn	h	-1.71	14.4	-0.04	0.01	0.00591
F82	scn	n	-151.06	7.9	-0.7105	0.0059	9.44	0.47	0.00065	F82	scn	n	-454.22	188.3	-0.47	0.10	1.01262
F82	scnso4	c	-23.16	16.0	0.1200	0.0099	6.23	2.08	0.00298	F82	scnso4	c	48.68	35.1	0.07	0.01	0.03400
F82	scnso4	h	-144.08	9.9	-0.2104	0.0069	13.86	1.52	0.00082	F82	scnso4	h	-296.00	55.0	-0.10	0.02	0.08364
F82	scnso4	n	23.63	36.5	-1.1156	0.0274	21.36	1.57	0.00873	F82	scnso4	n	-850.12	273.5	-0.45	0.11	2.06698
F82	so4	h	1.06	7.7	-0.2083	0.0074	55.95	4.73	0.00020	F82	so4	h	-143.59	41.3	-0.08	0.02	0.04761
F82	so4	n	140.98	17.2	-0.2059	0.0244	209.95	34.65	0.00021	F82	so4	n	-18.74	25.3	-0.04	0.01	0.01788
G34	cl	c	70.42	96.7	-0.3205	0.3129	999.78	772.04	0.00023	G34	cl	c	-96.00	11.0	-0.01	0.01	0.00310
G34	cl	h	-26.17	7.7	0.0054	0.0074	65.89	191.79	0.00017	G34	cl	h	-21.47	2.9	0.00	0.00	0.00021
G34	cl	n	-33.84	98.3	0.3268	0.3182	1000.00	769.93	0.00023	G34	cl	n	136.57	11.5	0.01	0.01	0.00336
G34	clso4	c	-69.88	5.1	-0.1026	0.0038	18.17	2.10	0.00019	G34	clso4	c	-140.46	25.8	-0.05	0.01	0.01770
G34	clso4	h	-122.21	134.4	0.1245	0.4352	1000.00	2766.70	0.00044	G34	clso4	h	-55.02	5.9	0.00	0.00	0.00092
G34	clso4	n	49.65	33.4	0.1293	0.0270	31.62	17.66	0.00569	G34	clso4	n	147.36	31.7	0.05	0.01	0.02674
G34	scn	c	-95.28	4.6	-0.0829	0.0036	16.17	3.08	0.00019	G34	scn	c	-135.79	20.9	-0.05	0.01	0.01246
G34	scncl	c	-118.30	7.9	-0.0905	0.0051	8.13	1.88	0.00064	G34	scncl	c	-167.62	24.2	-0.05	0.01	0.01553
G34	scncl	h	-41.15	2.7	-0.0060	0.0015	1.00	3.26	0.00011	G34	scncl	h	-42.90	2.6	-0.00	0.00	0.00018
G34	scncl	n	88.99	27.3	0.1798	0.0362	181.00	53.70	0.00067	G34	scncl	n	247.22	25.6	0.02	0.01	0.01744
G34	scn	h	-47.12	65.4	0.0330	0.2159	1000.00	5249.81	0.00016	G34	scn	h	-28.70	2.8	-0.00	0.00	0.00022
G34	scn	n	57.90	12.3	0.1924	0.0181	210.48	28.69	0.00014	G34	scn	n	199.77	25.2	0.04	0.01	0.01814
G34	scnso4	c	-114.40	8.8	-0.1130	0.0066	20.93	3.65	0.00051	G34	scnso4	c	-203.63	28.8	-0.04	0.01	0.02291

G34	scnso4	h	-254.14	163.8	0.2936	0.5297	997.62	1425.49	0.00065	G34	scnso4	h	-96.12	10.4	0.00	0.00	0.00299
G34	scnso4	n	-120.47	67.9	0.2746	0.0710	91.58	44.60	0.00957	G34	scnso4	n	121.73	45.6	0.06	0.02	0.05742
G34	so4	c	-90.18	11.2	-0.1306	0.0095	28.29	6.62	0.00071	G34	so4	c	-168.34	30.2	-0.07	0.02	0.02551
G34	so4	h	-17.27	4.5	0.0038	0.0030	1.00	28.75	0.00023	G34	so4	h	-16.09	3.1	0.00	0.00	0.00026
G34	so4	n	83.91	11.4	0.1353	0.0093	24.21	5.80	0.00079	G34	so4	n	161.61	32.0	0.07	0.02	0.02856
G40	cl	c	71.89	13.8	0.2566	0.0149	100.86	10.73	0.00035	G40	cl	c	285.81	46.5	0.06	0.02	0.05493
G40	cl	h	-7.24	12.7	0.0462	0.0155	145.09	78.26	0.00019	G40	cl	h	32.13	8.0	0.01	0.00	0.00163
G40	cl	n	97.71	20.3	0.6175	0.0234	122.11	7.90	0.00062	G40	cl	n	613.42	101.9	0.14	0.05	0.26419
G40	clso4	c	122.79	25.4	0.3269	0.0209	34.92	5.79	0.00306	G40	clso4	c	377.65	73.5	0.12	0.03	0.14332
G40	scn	c	20.81	8.5	0.4167	0.0070	21.74	1.38	0.00057	G40	scn	c	241.17	101.0	0.23	0.05	0.29130
G40	scncl	c	73.38	15.6	0.4408	0.0104	9.90	0.89	0.00233	G40	scncl	c	328.61	115.1	0.25	0.05	0.35250
G40	scncl	h	-7.42	4.2	0.1161	0.0030	13.62	1.21	0.00015	G40	scncl	h	66.36	30.0	0.06	0.01	0.02393
G40	scncl	n	25.03	56.7	1.5492	0.0386	11.41	1.04	0.02888	G40	scncl	n	958.94	400.8	0.85	0.17	4.27646
G40	scn	h	-14.59	1.8	0.1302	0.0015	23.20	0.98	0.00002	G40	scn	h	55.49	31.3	0.07	0.02	0.02802
G40	scn	n	-92.84	26.3	1.6354	0.0207	17.21	0.93	0.00595	G40	scn	n	720.86	407.6	0.97	0.21	4.74581
G40	scnso4	c	18.79	19.8	0.4827	0.0147	20.24	1.87	0.00266	G40	scnso4	c	394.44	120.4	0.20	0.05	0.40038
G40	scnso4	h	-92.95	12.6	0.2166	0.0078	6.11	0.90	0.00187	G40	scnso4	h	34.53	59.7	0.12	0.02	0.09831
G40	scnso4	n	-191.98	28.5	1.8767	0.0200	14.31	0.50	0.00668	G40	scnso4	n	1171.85	486.8	0.85	0.20	6.54758
G40	so4	c	45.52	31.1	0.1707	0.0348	102.61	38.90	0.00177	G40	so4	c	174.56	29.1	0.05	0.01	0.02364
G48	cl	c	22.95	11.5	-0.0350	0.0081	11.66	11.03	0.00110	G48	cl	c	4.07	11.4	-0.02	0.01	0.00333
G48	cl	h	-27.99	7.9	0.0075	0.0075	62.27	134.06	0.00018	G48	cl	h	-21.55	3.3	0.00	0.00	0.00027
G48	cl	n	50.20	8.5	0.0215	0.0107	155.11	121.03	0.00008	G48	cl	n	67.99	3.6	0.00	0.00	0.00033
G48	clso4	c	-19.74	4.4	-0.0316	0.0030	12.15	4.12	0.00017	G48	clso4	c	-39.22	8.5	-0.02	0.00	0.00191
G48	clso4	h	-27.54	2.3	-0.0080	0.0018	23.49	15.04	0.00003	G48	clso4	h	-33.39	2.3	-0.00	0.00	0.00013
G48	clso4	n	54.84	22.4	0.0348	0.0252	113.45	143.42	0.00082	G48	clso4	n	86.79	8.4	0.01	0.00	0.00189
G48	scn	c	-29.08	11.3	-0.0342	0.0077	1.00	8.34	0.00166	G48	scn	c	-39.49	12.6	-0.03	0.01	0.00456
G48	scncl	c	-40.49	12.9	-0.0560	0.0116	52.76	24.88	0.00057	G48	scncl	c	-86.92	12.5	-0.02	0.01	0.00414
G48	scncl	h	-63.61	2.4	-0.0040	0.0013	1.00	4.30	0.00008	G48	scncl	h	-64.77	2.1	-0.00	0.00	0.00012
G48	scncl	n	101.36	247.4	0.1527	0.8012	1000.00	4151.11	0.00149	G48	scncl	n	190.26	9.0	-0.00	0.00	0.00214
G48	scn	h	-54.59	6.1	0.0055	0.0075	128.07	311.17	0.00007	G48	scn	h	-50.10	1.9	0.00	0.00	0.00011
G48	scn	n	6.60	144.6	0.2734	0.4772	1000.00	1400.82	0.00078	G48	scn	n	146.71	12.3	0.01	0.01	0.00435
G48	scnso4	c	-77.17	9.4	-0.0283	0.0076	32.73	23.21	0.00044	G48	scnso4	c	-101.88	8.1	-0.01	0.00	0.00180
G48	scnso4	h	-82.05	45.3	0.0212	0.1468	1000.00	5482.10	0.00005	G48	scnso4	h	-69.58	1.5	-0.00	0.00	0.00006
G48	scnso4	n	68.32	69.8	0.1636	0.1403	446.15	400.82	0.00087	G48	scnso4	n	194.75	13.5	0.01	0.01	0.00504
G48	so4	c	47.43	10.3	-0.0315	0.0070	1.00	8.09	0.00124	G48	so4	c	37.33	11.4	-0.02	0.01	0.00364
G48	so4	h	-4.78	11.3	-0.0305	0.0179	274.27	201.44	0.00006	G48	so4	h	-28.30	3.7	-0.00	0.00	0.00038
G48	so4	n	49.33	3.9	0.0613	0.0032	23.21	4.29	0.00010	G48	so4	n	84.26	14.6	0.03	0.01	0.00594
G52	cl	c	27.84	8.6	0.0427	0.0084	68.95	28.49	0.00020	G52	cl	c	61.18	8.4	0.01	0.00	0.00178
G52	cl	h	-23.91	6.9	0.0141	0.0060	41.18	43.90	0.00020	G52	cl	h	-13.07	4.5	0.01	0.00	0.00051
G52	cl	n	10.15	9.0	0.0238	0.0093	84.96	64.65	0.00018	G52	cl	n	29.27	5.0	0.01	0.00	0.00063
G52	clso4	c	27.18	8.3	0.0766	0.0056	10.86	2.91	0.00063	G52	clso4	c	72.39	19.8	0.04	0.01	0.01037
G52	clso4	h	-21.86	9.5	0.0294	0.0176	386.76	256.21	0.00002	G52	clso4	h	1.01	2.7	0.00	0.00	0.00019
G52	clso4	n	19.42	4.6	0.0651	0.0027	3.13	0.79	0.00027	G52	clso4	n	45.92	18.0	0.05	0.01	0.00861
G52	scn	c	-33.80	16.1	0.0810	0.0244	221.56	94.33	0.00023	G52	scn	c	24.95	10.2	0.02	0.01	0.00298
G52	scncl	c	-20.32	12.8	0.0537	0.0092	16.47	9.23	0.00123	G52	scncl	c	14.62	14.5	0.03	0.01	0.00556
G52	scncl	h	-9.46	89.0	-0.0780	0.2882	1000.00	2922.76	0.00019	G52	scncl	h	-51.97	3.8	-0.00	0.00	0.00039
G52	scncl	n	9.74	3.9	-0.0341	0.0022	1.00	0.83	0.00023	G52	scncl	n	-0.31	9.9	-0.03	0.00	0.00261
G52	scn	h	-30.34	6.0	-0.0190	0.0084	180.53	122.91	0.00004	G52	scn	h	-43.89	2.6	-0.00	0.00	0.00020
G52	scn	n	-4.72	5.8	-0.0332	0.0039	1.00	4.38	0.00043	G52	scn	n	-14.81	10.5	-0.03	0.01	0.00316
G52	scnso4	c	-18.91	8.0	0.0736	0.0041	9.79	1.84	0.00030	G52	scnso4	c	48.33	28.6	0.04	0.01	0.01123
G52	scnso4	h	-44.09	43.7	0.0098	0.1414	1000.00	11427.31	0.00005	G52	scnso4	h	-38.60	1.3	-0.00	0.00	0.00005
G52	scnso4	n	-56.17	3.5	0.0289	0.0020	2.22	0.84	0.00019	G52	scnso4	n	-44.00	8.0	0.02	0.00	0.00178
G52	so4	c	29.26	9.2	0.0946	0.0080	32.56	8.29	0.00043	G52	so4	c	87.50	21.2	0.05	0.01	0.01251
G52	so4	h	29.27	17.7	-0.0449	0.0262	234.02	181.84	0.00019	G52	so4	h	-5.53	5.9	-0.01	0.00	0.00096
G52	so4	n	53.55	3.8	0.0798	0.0026	1.00	1.18	0.00017	G52	so4	n	79.41	23.9	0.06	0.01	0.01596
G53	cl	c	16.41	7.5	0.0108	0.0054	14.31	26.71	0.00042	G53	cl	c	22.69	5.0	0.01	0.00	0.00064
G53	cl	h	-6.04	7.3	0.0157	0.0070	64.76	62.10	0.00015	G53	cl	h	6.84	4.3	0.00	0.00	0.00046

G53	cl	n	36.17	75.4	0.1314	0.2439	999.31	1467.19	0.00014	G53	cl	n	103.23	4.5	0.00	0.00	0.00051
G53	clso4	c	-26.10	6.9	0.0608	0.0043	5.23	1.76	0.00056	G53	clso4	c	2.85	16.7	0.04	0.01	0.00741
G53	clso4	h	4.01	4.7	0.0162	0.0038	29.27	18.75	0.00012	G53	clso4	h	15.92	4.1	0.01	0.00	0.00044
G53	clso4	n	6.19	162.0	0.2733	0.5242	998.88	1516.14	0.00063	G53	clso4	n	151.56	10.2	0.00	0.00	0.00276
G53	scn	c	-132.24	3.6	0.0550	0.0041	107.48	15.51	0.00003	G53	scn	c	-92.50	9.5	0.02	0.00	0.00260
G53	sncl	c	-141.94	6.0	0.0437	0.0043	14.67	4.86	0.00029	G53	scncl	c	-113.64	11.7	0.02	0.01	0.00362
G53	scncl	h	-32.30	4.6	0.0098	0.0033	17.20	18.73	0.00015	G53	scncl	h	-25.35	3.6	0.00	0.00	0.00035
G53	scncl	n	196.30	331.5	0.0550	1.0735	1000.00	15433.58	0.00267	G53	scncl	n	237.86	8.1	-0.01	0.00	0.00173
G53	scn	h	-23.15	2.0	0.0173	0.0017	33.98	10.46	0.00002	G53	scn	h	-12.84	4.1	0.01	0.00	0.00048
G53	scn	n	155.96	222.5	0.0465	0.7344	1000.00	12676.03	0.00186	G53	scn	n	187.56	7.6	-0.00	0.00	0.00163
G53	scnso4	c	-182.15	7.8	0.0635	0.0046	4.02	1.32	0.00079	G53	scnso4	c	-149.79	17.5	0.04	0.01	0.00851
G53	scnso4	h	-34.30	7.7	0.0229	0.0080	85.91	57.37	0.00013	G53	scnso4	h	-14.02	4.2	0.01	0.00	0.00050
G53	scnso4	n	218.61	201.9	0.0762	0.6539	1000.00	6792.32	0.00098	G53	scnso4	n	264.75	6.1	-0.00	0.00	0.00102
G53	so4	c	-28.39	7.6	0.0715	0.0061	20.03	6.46	0.00039	G53	so4	c	10.92	17.7	0.04	0.01	0.00878
G53	so4	h	10.50	2.1	0.0097	0.0014	1.00	5.36	0.00005	G53	so4	h	13.64	3.2	0.01	0.00	0.00028
G53	so4	n	28.51	44.0	0.1143	0.0808	376.25	294.17	0.00050	G53	so4	n	108.63	9.4	0.01	0.00	0.00248
G61	cl	c	34.61	8.3	0.0248	0.0090	102.91	68.27	0.00012	G61	cl	c	55.27	5.0	0.01	0.00	0.00062
G61	cl	h	-5.78	10.7	0.0259	0.0123	121.25	98.62	0.00017	G61	cl	h	16.48	5.3	0.01	0.00	0.00072
G61	cl	n	189.00	21.2	0.2837	0.0336	275.65	41.05	0.00020	G61	cl	n	413.42	30.4	0.03	0.01	0.02353
G61	clso4	c	-48.96	76.7	0.2030	0.1824	605.55	509.14	0.00052	G61	clso4	c	83.23	11.2	0.01	0.00	0.00332
G61	clso4	h	5.32	2.5	0.0356	0.0017	12.09	2.10	0.00006	G61	clso4	h	27.29	9.3	0.02	0.00	0.00228
G61	clso4	n	354.99	17.6	0.2398	0.0126	15.86	2.73	0.00239	G61	clso4	n	513.17	60.4	0.12	0.03	0.09674
G61	scn	c	18.69	11.0	0.0723	0.0150	170.26	55.85	0.00016	G61	scn	c	72.49	10.9	0.02	0.01	0.00338
G61	scncl	c	-49.90	28.5	0.0912	0.0271	64.08	40.43	0.00238	G61	scncl	c	28.20	20.7	0.03	0.01	0.01142
G61	scncl	h	4.17	5.7	0.0317	0.0051	48.53	18.21	0.00012	G61	scncl	h	30.78	7.4	0.01	0.00	0.00146
G61	scncl	n	451.74	37.3	0.5423	0.0394	94.02	12.73	0.00281	G61	scncl	n	923.85	94.7	0.12	0.04	0.23871
G61	scn	h	-0.70	3.3	0.0443	0.0035	76.66	13.47	0.00004	G61	scn	h	30.10	8.6	0.02	0.00	0.00210
G61	scn	n	298.99	37.7	0.6009	0.0483	142.41	19.48	0.00235	G61	scn	n	737.81	91.4	0.15	0.05	0.23855
G61	scnso4	c	96.84	7.0	0.0384	0.0041	21.30	6.31	0.00014	G61	scnso4	c	137.06	13.2	0.01	0.00	0.00238
G61	scnso4	h	5.84	6.0	0.0446	0.0043	16.31	5.00	0.00027	G61	scnso4	h	38.63	11.4	0.02	0.00	0.00361
G61	scnso4	n	580.03	52.7	0.4711	0.0467	48.24	11.13	0.01032	G61	scnso4	n	988.64	96.5	0.14	0.04	0.25718
G61	so4	c	-2.17	72.3	0.1265	0.1600	538.33	658.20	0.00063	G61	so4	c	80.46	9.0	0.01	0.00	0.00227
G61	so4	h	12.11	2.6	0.0356	0.0020	12.75	3.47	0.00006	G61	so4	h	29.47	9.2	0.02	0.00	0.00236
G61	so4	n	145.50	13.4	0.2442	0.0111	24.82	3.85	0.00108	G61	so4	n	286.75	57.1	0.13	0.03	0.09109
G65	cl	c	23.61	96.4	-0.0577	0.3121	1000.00	4275.17	0.00022	G65	cl	c	-7.33	3.7	-0.00	0.00	0.00034
G65	cl	h	-66.21	95.9	-0.0271	0.3104	1000.00	9046.73	0.00022	G65	cl	h	-81.52	3.1	0.00	0.00	0.00025
G65	cl	n	28.73	6.1	0.0264	0.0050	32.56	17.00	0.00018	G65	cl	n	47.86	7.2	0.01	0.00	0.00131
G65	clso4	c	47.76	95.5	-0.0407	0.2328	632.67	3333.63	0.00073	G65	clso4	c	16.82	5.6	0.00	0.00	0.00085
G65	clso4	h	-67.64	3.9	-0.0237	0.0036	55.98	18.98	0.00005	G65	clso4	h	-87.18	4.8	-0.01	0.00	0.00060
G65	clso4	n	133.86	6.0	0.0300	0.0034	1.00	1.43	0.00052	G65	clso4	n	142.88	9.6	0.02	0.00	0.00245
G65	scn	c	-67.32	2.8	-0.0023	0.0019	1.00	30.69	0.00010	G65	scn	c	-68.03	2.0	-0.00	0.00	0.00012
G65	scncl	c	-77.23	6.0	0.0059	0.0034	1.00	7.28	0.00052	G65	scncl	c	-75.53	4.7	0.00	0.00	0.00059
G65	scncl	h	-124.72	16.2	-0.0829	0.0235	223.56	86.27	0.00017	G65	scncl	h	-193.20	9.3	-0.01	0.00	0.00229
G65	scncl	n	-107.08	243.6	-0.0757	0.7889	1000.00	8241.91	0.00144	G65	scncl	n	-155.52	7.0	0.00	0.00	0.00129
G65	scn	h	-117.61	7.5	-0.0524	0.0120	251.54	77.12	0.00004	G65	scn	h	-154.94	5.8	-0.01	0.00	0.00097
G65	scn	n	-203.66	4.6	0.0050	0.0033	4.91	30.25	0.00025	G65	scn	n	-201.78	3.3	0.00	0.00	0.00031
G65	scnso4	c	-12.21	131.5	-0.0670	0.3310	667.52	2984.25	0.00121	G65	scnso4	c	-61.65	7.2	0.00	0.00	0.00144
G65	scnso4	h	-163.60	3.2	-0.0254	0.0030	61.41	15.63	0.00003	G65	scnso4	h	-186.03	4.8	-0.01	0.00	0.00065
G65	scnso4	n	-234.59	3.6	0.0327	0.0020	2.49	0.81	0.00019	G65	scnso4	n	-220.22	9.1	0.02	0.00	0.00227
G65	so4	c	7.01	2.7	-0.0126	0.0023	26.73	15.77	0.00004	G65	so4	c	-0.50	3.2	-0.01	0.00	0.00029
G65	so4	h	14.34	15.6	-0.0549	0.0321	472.32	280.12	0.00004	G65	so4	h	-23.32	4.1	-0.00	0.00	0.00047
G65	so4	n	83.34	5.0	0.0454	0.0034	1.10	2.73	0.00029	G65	so4	n	98.14	13.9	0.03	0.01	0.00538
G68	cl	c	-1.60	3.7	0.0055	0.0022	1.00	8.51	0.00017	G68	cl	c	0.12	3.1	0.00	0.00	0.00025
G68	clso4	c	32.18	6.5	0.0117	0.0052	30.87	36.83	0.00022	G68	clso4	c	41.02	3.8	0.00	0.00	0.00039
G68	clso4	h	-74.18	12.6	0.0219	0.0191	251.08	286.46	0.00008	G68	clso4	h	-57.47	2.2	0.00	0.00	0.00013
G68	clso4	n	68.64	10.8	-0.0980	0.0095	46.14	10.60	0.00045	G68	clso4	n	-11.75	21.4	-0.03	0.01	0.01218
G68	scn	c	-1.17	57.2	0.0644	0.1888	999.33	2352.68	0.00012	G68	scn	c	31.94	3.4	0.00	0.00	0.00033

G68	scncl	c	20.68	6.2	0.0078	0.0037	3.17	9.02	0.00050	G68	scncl	c	23.90	4.9	0.01	0.00	0.00063
G68	scncl	h	-243.78	12.6	-0.0531	0.0159	158.87	73.39	0.00017	G68	scncl	h	-289.37	7.5	-0.01	0.00	0.00151
G68	scncl	n	-517.19	11.0	-0.0058	0.0081	19.31	84.98	0.00083	G68	scncl	n	-521.36	5.8	-0.00	0.00	0.00090
G68	scnso4	c	47.12	9.1	0.0166	0.0056	6.10	8.38	0.00096	G68	scnso4	c	56.62	7.3	0.01	0.00	0.00147
G68	scnso4	h	-281.43	16.0	-0.0119	0.0251	269.07	723.39	0.00012	G68	scnso4	h	-293.37	2.5	0.00	0.00	0.00018
G68	scnso4	n	-619.71	7.2	-0.0947	0.0056	27.72	4.56	0.00028	G68	scnso4	n	-698.22	23.1	-0.03	0.01	0.01478
G68	so4	c	0.10	119.1	0.0941	0.3834	1000.00	3200.18	0.00036	G68	so4	c	49.33	4.9	0.00	0.00	0.00067
G81	cl	c	-50.30	16.0	-0.2177	0.0138	40.34	6.46	0.00107	G81	cl	c	-210.10	49.4	-0.09	0.02	0.06221
G81	cl	h	-57.23	10.8	-0.0605	0.0125	122.17	43.02	0.00017	G81	cl	h	-107.24	10.0	-0.01	0.00	0.00253
G81	cl	n	107.69	11.5	0.2810	0.0132	122.58	9.85	0.00020	G81	cl	n	342.34	46.3	0.06	0.02	0.05456
G81	clso4	c	21.03	14.7	-0.3577	0.0105	15.53	1.50	0.00168	G81	clso4	c	-214.87	90.6	-0.18	0.04	0.21775
G81	clso4	h	-80.67	4.9	-0.0820	0.0037	22.43	3.03	0.00015	G81	clso4	h	-139.47	19.8	-0.04	0.01	0.01046
G81	clso4	n	203.52	16.5	0.3581	0.0122	19.82	2.07	0.00185	G81	clso4	n	453.87	88.3	0.17	0.04	0.20699
G81	scn	c	-111.44	4.4	0.0534	0.0030	1.00	2.09	0.00025	G81	scn	c	-95.14	16.1	0.04	0.01	0.00740
G81	scncl	c	-84.36	20.6	-0.4455	0.0142	12.18	1.39	0.00370	G81	scncl	c	-357.73	114.9	-0.24	0.05	0.35118
G81	scncl	h	-128.80	7.1	-0.0904	0.0048	11.25	2.20	0.00045	G81	scncl	h	-182.84	23.4	-0.05	0.01	0.01462
G81	scncl	n	310.36	12.8	0.4342	0.0088	11.77	0.86	0.00145	G81	scncl	n	574.70	112.3	0.24	0.05	0.33568
G81	scn	h	-88.53	2.6	-0.0738	0.0021	17.40	2.08	0.00006	G81	scn	h	-125.31	18.4	-0.04	0.01	0.00965
G81	scn	n	212.85	11.1	0.4201	0.0089	20.14	1.68	0.00099	G81	scn	n	430.61	102.8	0.24	0.05	0.30201
G81	scnso4	c	-48.04	20.6	-0.5079	0.0145	14.91	1.40	0.00341	G81	scnso4	c	-423.52	134.1	-0.23	0.06	0.49652
G81	scnso4	h	-125.02	2.7	-0.0899	0.0019	14.15	1.00	0.00006	G81	scnso4	h	-190.40	23.6	-0.04	0.01	0.01534
G81	scnso4	n	299.23	12.0	0.4803	0.0084	14.76	0.85	0.00116	G81	scnso4	n	650.22	124.0	0.22	0.05	0.42477
G81	so4	c	0.73	26.7	-0.4333	0.0244	44.24	6.59	0.00292	G81	so4	c	-285.79	90.6	-0.19	0.05	0.22918
G81	so4	h	-24.12	2.6	-0.0689	0.0028	83.88	6.76	0.00002	G81	so4	h	-75.33	12.3	-0.02	0.01	0.00423
G81	so4	n	83.32	16.4	0.3217	0.0152	46.52	5.66	0.00105	G81	so4	n	298.67	66.7	0.14	0.03	0.12421
G9	cl	c	-0.90	3.9	0.0191	0.0024	1.00	2.63	0.00019	G9	cl	c	5.04	6.6	0.01	0.00	0.00109
G9	cl	h	-20.84	6.2	0.0082	0.0053	40.95	66.66	0.00016	G9	cl	h	-14.40	3.3	0.00	0.00	0.00027
G9	cl	n	-6.39	33.5	0.1275	0.0614	373.34	201.43	0.00029	G9	cl	n	87.32	10.8	0.01	0.00	0.00297
G9	clso4	c	4.31	12.8	-0.0343	0.0128	76.56	57.12	0.00041	G9	clso4	c	-24.62	7.1	-0.01	0.00	0.00133
G9	clso4	h	3.45	3.9	0.0138	0.0030	26.25	16.33	0.00009	G9	clso4	h	13.73	3.8	0.01	0.00	0.00038
G9	clso4	n	-151.65	83.2	0.4068	0.1064	164.79	65.90	0.00706	G9	clso4	n	198.07	56.2	0.07	0.02	0.08387
G9	scn	c	-77.66	12.6	-0.4727	0.0097	14.18	1.37	0.00146	G9	scn	c	-301.11	120.4	-0.29	0.06	0.41408
G9	scncl	c	-15.42	94.4	-0.1789	0.2496	725.18	888.77	0.00051	G9	scncl	c	-120.27	7.1	-0.01	0.00	0.00133
G9	scncl	h	-40.45	88.7	-0.0229	0.2871	1000.00	9926.96	0.00019	G9	scncl	h	-52.97	2.8	-0.00	0.00	0.00021
G9	scncl	n	-297.50	29.3	0.4658	0.0554	395.81	51.52	0.00020	G9	scncl	n	51.93	35.7	0.03	0.02	0.03394
G9	scn	h	-44.79	2.3	0.0045	0.0020	29.92	44.17	0.00004	G9	scn	h	-42.17	1.6	0.00	0.00	0.00007
G9	scn	n	-336.91	65.4	0.8086	0.2011	894.31	184.95	0.00022	G9	scn	n	83.17	32.2	0.03	0.02	0.02959
G9	scnso4	c	-178.70	24.0	-0.0120	0.0174	17.31	79.15	0.00427	G9	scnso4	c	-186.03	12.4	-0.01	0.01	0.00428
G9	scnso4	h	-46.39	4.4	0.0170	0.0037	36.26	19.85	0.00009	G9	scnso4	h	-32.05	4.1	0.01	0.00	0.00047
G9	scnso4	n	-332.40	86.9	0.6138	0.1373	273.24	77.23	0.00350	G9	scnso4	n	173.58	57.3	0.06	0.02	0.09072
G9	so4	c	-11.36	22.1	-0.0469	0.0276	145.37	137.96	0.00059	G9	so4	c	-47.37	8.0	-0.01	0.00	0.00179
G9	so4	h	16.38	2.2	0.0118	0.0017	14.70	9.36	0.00004	G9	so4	h	22.37	3.2	0.01	0.00	0.00028
G9	so4	n	165.19	14.4	0.1444	0.0149	76.10	16.47	0.00052	G9	so4	n	269.94	26.1	0.05	0.01	0.01899
H102	cl	c	-1.12	12.2	0.0023	0.0120	72.49	796.71	0.00039	H102	cl	c	0.42	3.9	0.00	0.00	0.00039
H102	cl	h	7.07	6.7	0.0057	0.0061	54.38	132.00	0.00015	H102	cl	h	11.82	2.8	0.00	0.00	0.00020
H102	cl	n	36.01	14.4	0.1253	0.0211	228.92	52.04	0.00013	H102	cl	n	136.88	14.8	0.02	0.01	0.00556
H102	scn	c	-2.98	5.9	-0.0272	0.0059	59.27	31.36	0.00014	H102	scn	c	-20.87	5.8	-0.01	0.00	0.00096
H102	scn	h	-1.27	2.8	0.0187	0.0026	50.52	18.58	0.00004	H102	scn	h	10.96	4.2	0.01	0.00	0.00051
H102	scn	n	27.11	16.6	0.2419	0.0196	111.50	17.04	0.00061	H102	scn	n	200.98	40.4	0.07	0.02	0.04660
H102	so4	c	21.46	9.0	-0.0255	0.0061	1.00	8.69	0.00094	H102	so4	c	13.28	9.5	-0.02	0.00	0.00252
H102	so4	h	29.22	2.1	0.0099	0.0015	1.00	5.33	0.00005	H102	so4	h	32.44	3.3	0.01	0.00	0.00030
H102	so4	n	50.78	12.1	0.1566	0.0098	22.06	5.01	0.00094	H102	so4	n	138.58	37.5	0.09	0.02	0.03918
H18	cl	c	-9.84	198.5	-0.0637	0.6426	1000.00	7973.54	0.00095	H18	cl	c	-48.52	6.3	0.00	0.00	0.00102
H18	cl	h	-13.44	8.1	0.0130	0.0078	67.12	85.44	0.00018	H18	cl	h	-2.57	4.0	0.00	0.00	0.00040
H18	cl	n	60.44	8.1	0.0113	0.0067	32.58	52.58	0.00032	H18	cl	n	68.85	4.7	0.00	0.00	0.00055
H18	clso4	c	-9.50	10.6	-0.0252	0.0066	5.66	6.84	0.00127	H18	clso4	c	-22.17	9.9	-0.02	0.00	0.00258
H18	clso4	h	5.12	2.8	0.0217	0.0019	11.64	3.72	0.00007	H18	clso4	h	18.35	5.8	0.01	0.00	0.00089

H18	clso4	n	165.27	32.3	0.0569	0.0380	131.38	145.74	0.00143	H18	clso4	n	212.64	10.1	0.01	0.00	0.00273
H18	scn	c	-141.04	11.3	-0.0354	0.0117	70.55	53.29	0.00045	H18	scn	c	-165.55	8.2	-0.01	0.00	0.00190
H18	scncl	c	-112.46	21.7	-0.0910	0.0283	173.97	81.07	0.00045	H18	scncl	c	-188.84	11.6	-0.02	0.01	0.00358
H18	scncl	h	-43.48	3.8	0.0071	0.0026	11.91	15.97	0.00013	H18	scncl	h	-38.91	3.0	0.00	0.00	0.00024
H18	scncl	n	97.87	49.3	0.0402	0.0926	391.29	990.38	0.00057	H18	scncl	n	132.10	6.1	0.00	0.00	0.00098
H18	scn	h	-42.22	3.6	0.0126	0.0032	35.77	27.56	0.00008	H18	scn	h	-34.54	3.4	0.01	0.00	0.00033
H18	scn	n	-5.25	22.8	0.1035	0.0320	184.03	86.89	0.00061	H18	scn	n	69.86	14.2	0.02	0.01	0.00579
H18	scnso4	c	-138.61	12.2	-0.0259	0.0104	40.42	39.75	0.00063	H18	scnso4	c	-162.08	7.9	-0.01	0.00	0.00172
H18	scnso4	h	-46.64	3.3	0.0252	0.0023	14.00	4.27	0.00009	H18	scnso4	h	-28.34	6.9	0.01	0.00	0.00130
H18	scnso4	n	216.15	15.7	0.0290	0.0132	38.37	43.91	0.00109	H18	scnso4	n	243.20	9.9	0.01	0.00	0.00269
H18	so4	c	26.18	8.6	-0.0165	0.0059	1.00	12.93	0.00087	H18	so4	c	20.91	7.4	-0.01	0.00	0.00152
H18	so4	h	30.02	2.3	0.0203	0.0016	6.29	3.86	0.00005	H18	so4	h	38.41	5.7	0.01	0.00	0.00092
H18	so4	n	198.88	7.0	0.0406	0.0050	6.90	6.10	0.00047	H18	so4	n	215.92	11.7	0.03	0.01	0.00385
I109	cl	c	-30.76	258.7	-0.0699	0.8375	1000.00	9477.58	0.00162	I109	cl	c	-72.72	8.2	0.00	0.00	0.00173
I109	cl	h	-51.13	20.6	0.0437	0.0290	209.21	193.92	0.00030	I109	cl	h	-15.49	6.4	0.01	0.00	0.00105
I109	cl	n	389.23	144.2	0.2679	0.2018	205.97	218.25	0.01526	I109	cl	n	609.03	42.1	0.04	0.02	0.04513
I109	clso4	c	197.00	333.3	-0.7374	1.0792	1000.00	1157.83	0.00268	I109	clso4	c	-181.91	21.6	-0.02	0.01	0.01238
I109	clso4	h	-47.77	5.1	0.0150	0.0031	3.82	4.27	0.00032	I109	clso4	h	-41.22	5.4	0.01	0.00	0.00077
I109	clso4	n	1033.56	117.6	0.6134	0.1078	55.78	21.85	0.04556	I109	clso4	n	1539.75	126.0	0.19	0.05	0.42146
I109	scn	c	-57.52	15.7	-0.0381	0.0284	336.02	299.71	0.00011	I109	scn	c	-83.57	3.9	-0.01	0.00	0.00043
I109	scncl	c	45.04	223.9	-0.2901	0.7251	1000.00	1977.70	0.00122	I109	scncl	c	-113.98	12.3	-0.00	0.01	0.00401
I109	scncl	h	-80.65	5.1	0.0113	0.0031	4.20	6.08	0.00033	I109	scncl	h	-75.55	4.7	0.01	0.00	0.00059
I109	scncl	n	83.75	42.8	0.4522	0.0491	121.81	22.53	0.00278	I109	scncl	n	480.11	72.2	0.09	0.03	0.13872
I109	scn	h	-84.29	3.5	0.0233	0.0031	35.02	14.11	0.00007	I109	scn	h	-70.26	5.6	0.01	0.00	0.00090
I109	scn	n	34.05	13.1	0.2732	0.0164	134.05	14.04	0.00030	I109	scn	n	235.75	44.3	0.07	0.02	0.05613
I109	scnso4	c	-35.73	26.3	-0.1705	0.0367	204.80	62.13	0.00052	I109	scnso4	c	-183.48	19.6	-0.02	0.01	0.01061
I109	scnso4	h	-90.25	5.6	0.0142	0.0033	4.41	4.56	0.00040	I109	scnso4	h	-82.43	5.6	0.01	0.00	0.00087
I109	scnso4	n	32.35	119.0	0.8779	0.1212	83.41	22.37	0.03236	I109	scnso4	n	824.30	154.0	0.19	0.06	0.65483
I109	so4	c	-169.63	16.4	-0.0814	0.0173	82.69	35.73	0.00062	I109	so4	c	-228.87	14.6	-0.03	0.01	0.00591
I109	so4	h	-35.61	5.9	0.0134	0.0040	1.00	10.82	0.00040	I109	so4	h	-31.30	5.5	0.01	0.00	0.00084
I109	so4	n	1016.10	112.2	0.7666	0.1251	101.39	30.91	0.02337	I109	so4	n	1589.06	125.4	0.23	0.06	0.43905
I25	cl	c	52.77	90.5	-0.1291	0.2927	998.66	1791.19	0.00020	I25	cl	c	-16.10	5.6	-0.00	0.00	0.00079
I25	cl	h	-8.15	9.1	0.0427	0.0098	99.93	42.17	0.00015	I25	cl	h	27.70	8.3	0.01	0.00	0.00174
I25	cl	n	-131.17	70.7	0.0285	0.2287	1000.00	6334.60	0.00012	I25	cl	n	-114.85	2.4	-0.00	0.00	0.00015
I25	clso4	c	29.15	7.3	-0.0426	0.0044	3.51	2.05	0.00067	I25	clso4	c	11.09	12.7	-0.03	0.01	0.00426
I25	clso4	h	7.32	5.1	0.0499	0.0038	19.39	4.52	0.00018	I25	clso4	h	41.92	12.5	0.02	0.01	0.00412
I25	clso4	n	-221.97	19.7	0.0311	0.0112	1.00	4.57	0.00567	I25	clso4	n	-213.42	16.5	0.02	0.01	0.00725
I25	scn	c	3.72	7.5	-0.0849	0.0067	36.35	8.61	0.00033	I25	scn	c	-47.10	19.2	-0.04	0.01	0.01053
I25	scncl	c	31.26	17.7	-0.0952	0.0179	80.18	29.67	0.00075	I25	scncl	c	-50.41	17.9	-0.02	0.01	0.00855
I25	scncl	h	-15.14	3.6	0.0423	0.0026	16.36	3.27	0.00010	I25	scncl	h	13.09	10.9	0.02	0.00	0.00317
I25	scncl	n	-222.58	13.4	0.0989	0.0138	86.41	23.22	0.00040	I25	scncl	n	-135.11	19.1	0.02	0.01	0.00971
I25	scn	h	-18.77	2.7	0.0428	0.0025	41.79	6.86	0.00004	I25	scn	h	7.85	9.5	0.02	0.00	0.00259
I25	scn	n	-165.56	3.4	0.1856	0.0036	76.19	3.24	0.00004	I25	scn	n	-36.89	35.6	0.07	0.02	0.03612
I25	scnso4	c	14.80	6.7	-0.0707	0.0047	13.88	3.06	0.00038	I25	scnso4	c	-36.10	18.6	-0.03	0.01	0.00959
I25	scnso4	h	-19.27	5.3	0.0531	0.0039	17.78	4.07	0.00021	I25	scnso4	h	21.09	13.7	0.02	0.01	0.00520
I25	scnso4	n	-144.71	3.5	0.0802	0.0022	6.24	0.67	0.00014	I25	scnso4	n	-97.86	21.4	0.05	0.01	0.01262
I25	so4	c	9.99	4.6	-0.0590	0.0031	2.30	2.08	0.00023	I25	so4	c	-10.61	17.4	-0.04	0.01	0.00847
I25	so4	h	11.70	3.5	0.0377	0.0028	19.84	5.65	0.00008	I25	so4	h	32.31	9.2	0.02	0.00	0.00239
I25	so4	n	-151.29	16.4	0.0746	0.0112	1.00	5.44	0.00315	I25	so4	n	-127.27	24.5	0.06	0.01	0.01675
I4	cl	c	47.77	9.3	0.0031	0.0075	30.18	208.64	0.00044	I4	cl	c	50.62	4.3	0.00	0.00	0.00047
I4	cl	h	-96.77	6.3	0.0088	0.0054	39.62	62.35	0.00017	I4	cl	h	-89.92	3.5	0.00	0.00	0.00030
I4	cl	n	52.50	194.9	-0.1076	0.6308	1000.00	4637.52	0.00092	I4	cl	n	-9.13	7.3	0.00	0.00	0.00134
I4	clso4	c	148.19	118.6	-0.1394	0.3839	999.09	2177.43	0.00034	I4	clso4	c	75.47	5.5	-0.00	0.00	0.00081
I4	clso4	h	-127.57	1.8	0.0093	0.0013	19.61	8.58	0.00002	I4	clso4	h	-121.00	2.5	0.00	0.00	0.00017
I4	clso4	n	-43.71	120.9	0.0368	0.3915	1000.00	8407.52	0.00035	I4	clso4	n	-22.80	3.9	-0.00	0.00	0.00040
I4	scn	c	53.76	5.8	-0.0059	0.0039	1.00	24.76	0.00043	I4	scn	c	51.99	4.3	-0.00	0.00	0.00052
I4	scncl	c	69.08	7.6	0.0083	0.0053	14.02	30.90	0.00047	I4	scncl	c	74.60	4.8	0.00	0.00	0.00061

I4	scncl	h	-67.82	107.4	-0.1867	0.3477	999.74	1473.50	0.00028	I4	scncl	h	-167.01	6.9	-0.00	0.00	0.00128
I4	scncl	n	-353.21	20.5	-0.0888	0.0190	57.76	27.15	0.00135	I4	scncl	n	-425.51	17.8	-0.03	0.01	0.00843
I4	scn	h	-60.35	43.9	-0.1133	0.1448	997.97	1024.53	0.00007	I4	scn	h	-117.01	4.6	-0.00	0.00	0.00060
I4	scn	n	-331.22	35.8	-0.1643	0.0839	562.87	281.18	0.00020	I4	scn	n	-432.72	10.7	-0.01	0.01	0.00328
I4	scnso4	c	67.94	198.2	0.0191	0.6421	1000.00	26632.60	0.00094	I4	scnso4	c	76.55	5.8	0.00	0.00	0.00092
I4	scnso4	h	-190.48	61.3	0.0279	0.1987	1000.00	5629.32	0.00009	I4	scnso4	h	-177.06	1.6	0.00	0.00	0.00007
I4	scnso4	n	-98.64	654.4	-0.6037	2.1168	997.94	2771.46	0.01033	I4	scnso4	n	-421.77	27.4	-0.01	0.01	0.02074
I4	so4	c	2.90	9.5	-0.0186	0.0065	1.00	12.62	0.00106	I4	so4	c	-3.05	8.2	-0.01	0.00	0.00189
I4	so4	h	-48.46	2.5	0.0053	0.0017	1.24	11.80	0.00007	I4	so4	h	-46.73	2.2	0.00	0.00	0.00014
I4	so4	n	-43.77	7.3	0.0439	0.0056	13.61	8.04	0.00042	I4	so4	n	-22.08	11.7	0.03	0.01	0.00383
I51	cl	c	26.15	81.3	-0.1439	0.2633	1000.00	1447.18	0.00016	I51	cl	c	-48.88	5.5	-0.00	0.00	0.00077
I51	cl	h	-3.94	7.9	0.0668	0.0079	78.53	18.64	0.00015	I51	cl	h	50.93	13.5	0.02	0.01	0.00462
I51	cl	n	-4.71	10.1	0.2624	0.0120	131.33	9.98	0.00014	I51	cl	n	214.65	42.0	0.06	0.02	0.04491
I51	clso4	c	-58.10	10.5	-0.0395	0.0070	8.95	6.14	0.00110	I51	clso4	c	-80.78	12.4	-0.02	0.01	0.00411
I51	clso4	h	16.28	4.5	0.0812	0.0033	17.34	2.23	0.00015	I51	clso4	h	71.25	20.3	0.04	0.01	0.01097
I51	clso4	n	39.13	16.9	0.3701	0.0113	10.02	1.15	0.00268	I51	clso4	n	255.01	96.1	0.21	0.04	0.24506
I51	scn	c	-28.47	2.3	-0.0168	0.0017	11.45	6.26	0.00005	I51	scn	c	-36.04	4.6	-0.01	0.00	0.00061
I51	scncl	c	-32.76	13.9	-0.0439	0.0128	55.15	35.80	0.00065	I51	scncl	c	-68.05	9.4	-0.01	0.00	0.00233
I51	scncl	h	-19.01	2.4	0.0983	0.0016	9.59	0.60	0.00006	I51	scncl	h	37.59	25.9	0.06	0.01	0.01782
I51	scncl	n	-27.72	17.7	0.4398	0.0120	10.65	1.08	0.00292	I51	scncl	n	231.88	114.0	0.25	0.05	0.34590
I51	scn	h	-22.15	1.4	0.1055	0.0011	17.71	0.76	0.00002	I51	scn	h	30.75	26.2	0.06	0.01	0.01965
I51	scn	n	-48.95	9.6	0.4289	0.0079	22.98	1.57	0.00070	I51	scn	n	181.03	103.1	0.24	0.05	0.30380
I51	scnso4	c	-26.55	6.4	-0.0321	0.0046	15.59	7.21	0.00033	I51	scnso4	c	-49.85	8.6	-0.01	0.00	0.00205
I51	scnso4	h	-27.83	3.4	0.1162	0.0024	13.10	0.90	0.00010	I51	scnso4	h	54.60	30.1	0.05	0.01	0.02503
I51	scnso4	n	-37.22	14.7	0.4783	0.0096	9.00	0.67	0.00221	I51	scnso4	n	270.73	125.1	0.25	0.05	0.43235
I51	so4	c	-10.48	2.6	-0.0402	0.0019	7.85	2.42	0.00006	I51	so4	c	-27.85	10.9	-0.03	0.01	0.00330
I51	so4	h	15.64	3.3	0.0439	0.0026	17.57	4.19	0.00008	I51	so4	h	38.90	10.9	0.03	0.01	0.00331
I51	so4	n	26.54	16.0	0.3582	0.0134	26.54	3.28	0.00149	I51	so4	n	237.29	82.8	0.19	0.04	0.19121
I55	cl	c	30.88	222.4	-0.1016	0.7199	1000.00	5601.10	0.00119	I55	cl	c	-25.07	7.9	-0.00	0.00	0.00160
I55	cl	h	-29.22	85.7	-0.0455	0.2776	1000.00	4820.97	0.00018	I55	cl	h	-53.38	3.1	-0.00	0.00	0.00025
I55	cl	n	-18.67	7.6	-0.0217	0.0104	197.75	135.87	0.00004	I55	cl	n	-37.02	3.4	-0.00	0.00	0.00029
I55	clso4	c	-4.42	2.7	-0.0450	0.0017	7.40	1.17	0.00007	I55	clso4	c	-28.59	12.1	-0.03	0.01	0.00390
I55	clso4	h	-30.03	0.6	-0.0201	0.0004	8.66	0.67	0.00000	I55	clso4	h	-41.28	5.3	-0.01	0.00	0.00074
I55	clso4	n	31.57	5.6	-0.0825	0.0034	3.52	0.81	0.00040	I55	clso4	n	-3.24	22.7	-0.06	0.01	0.01362
I55	scn	c	-53.59	5.6	-0.0724	0.0053	49.07	9.52	0.00015	I55	scn	c	-99.67	15.3	-0.03	0.01	0.00665
I55	scncl	c	-26.44	12.9	-0.0925	0.0138	97.32	26.78	0.00033	I55	scncl	c	-107.05	16.2	-0.02	0.01	0.00701
I55	scncl	h	-118.29	11.3	-0.0391	0.0101	50.70	30.21	0.00046	I55	scncl	h	-148.90	8.0	-0.01	0.00	0.00169
I55	scncl	n	-49.42	18.4	-0.1071	0.0115	5.56	2.78	0.00390	I55	scncl	n	-100.90	30.4	-0.07	0.01	0.02466
I55	scn	h	-97.77	6.4	-0.0419	0.0084	152.75	50.44	0.00006	I55	scn	h	-128.20	6.2	-0.01	0.00	0.00109
I55	scn	n	-78.84	5.5	-0.0982	0.0048	33.84	5.17	0.00019	I55	scn	n	-136.67	22.3	-0.05	0.01	0.01420
I55	scnso4	c	-58.64	9.7	-0.0779	0.0053	14.65	3.08	0.00035	I55	scnso4	c	-136.31	29.1	-0.03	0.01	0.01163
I55	scnso4	h	-137.18	2.6	-0.0161	0.0017	8.38	3.24	0.00007	I55	scnso4	h	-147.26	4.4	-0.01	0.00	0.00054
I55	scnso4	n	-76.81	7.4	-0.0909	0.0044	4.61	0.98	0.00069	I55	scnso4	n	-124.80	24.1	-0.06	0.01	0.01603
I55	so4	c	18.07	16.8	-0.0581	0.0143	29.81	23.08	0.00153	I55	so4	c	-17.44	15.3	-0.03	0.01	0.00654
I55	so4	h	14.76	3.1	-0.0256	0.0032	74.15	19.42	0.00002	I55	so4	h	-3.89	4.8	-0.01	0.00	0.00064
I55	so4	n	16.27	4.9	-0.1047	0.0037	11.19	2.07	0.00021	I55	so4	n	-33.14	27.3	-0.07	0.01	0.02079
I76	cl	c	48.35	6.1	0.0117	0.0041	9.25	14.83	0.00033	I76	cl	c	54.21	4.8	0.01	0.00	0.00058
I76	cl	h	-20.49	5.9	0.0062	0.0048	32.12	68.53	0.00017	I76	cl	h	-15.80	3.1	0.00	0.00	0.00024
I76	cl	n	-23.20	11.9	-0.1321	0.0125	91.05	16.39	0.00029	I76	cl	n	-132.96	25.2	-0.03	0.01	0.01611
I76	clso4	c	68.04	3.5	0.0229	0.0024	13.52	4.90	0.00010	I76	clso4	c	82.61	6.2	0.01	0.00	0.00101
I76	clso4	h	-6.10	1.3	0.0038	0.0007	1.91	2.90	0.00002	I76	clso4	h	-4.74	1.4	0.00	0.00	0.00005
I76	clso4	n	-39.01	6.4	-0.1653	0.0046	18.13	1.61	0.00029	I76	clso4	n	-152.70	41.6	-0.08	0.02	0.04593
I76	scn	c	69.86	209.3	0.0273	0.6907	1000.00	20340.87	0.00164	I76	scn	c	91.61	6.7	-0.01	0.00	0.00127
I76	scncl	c	84.79	9.4	0.0341	0.0094	77.82	42.92	0.00022	I76	scncl	c	114.81	7.3	0.01	0.00	0.00142
I76	scncl	h	-16.15	88.7	-0.0385	0.2871	999.68	5902.10	0.00019	I76	scncl	h	-37.33	3.0	-0.00	0.00	0.00024
I76	scncl	n	-49.33	11.3	-0.1919	0.0074	8.68	1.34	0.00128	I76	scncl	n	-155.77	50.3	-0.11	0.02	0.06728
I76	scn	h	-32.18	3.1	0.0110	0.0029	42.18	31.12	0.00005	I76	scn	h	-25.17	2.9	0.00	0.00	0.00024

I76	scn	n	-52.37	5.9	-0.1078	0.0046	14.98	2.94	0.00032	I76	scn	n	-103.98	27.4	-0.07	0.01	0.02142
I76	scnso4	c	115.89	7.8	0.0183	0.0055	14.91	14.63	0.00049	I76	scnso4	c	129.57	6.5	0.01	0.00	0.00116
I76	scnso4	h	-316.09	146.8	0.4359	0.4754	1000.00	863.26	0.00052	I76	scnso4	h	-88.16	12.4	0.01	0.01	0.00422
I76	scnso4	n	-124.05	10.9	-0.1211	0.0064	4.05	0.97	0.00156	I76	scnso4	n	-185.96	32.8	-0.08	0.01	0.02976
I76	so4	c	7.80	23.4	0.0664	0.0406	333.82	236.30	0.00018	I76	so4	c	56.70	6.6	0.01	0.00	0.00121
I76	so4	h	9.61	1.6	0.0125	0.0011	1.00	3.07	0.00003	I76	so4	h	13.64	3.8	0.01	0.00	0.00041
I76	so4	n	99.24	19.1	-0.0947	0.0250	168.54	67.62	0.00036	I76	so4	n	24.63	13.8	-0.02	0.01	0.00533
I88	cl	c	-42.71	5.1	-0.0476	0.0060	132.13	27.81	0.00004	I88	cl	c	-82.46	7.6	-0.01	0.00	0.00149
I88	cl	h	0.51	7.3	0.0248	0.0068	59.85	36.42	0.00016	I88	cl	h	20.53	6.1	0.01	0.00	0.00094
I88	cl	n	27.72	8.3	0.0541	0.0098	134.43	40.39	0.00009	I88	cl	n	73.22	8.9	0.01	0.00	0.00203
I88	clso4	c	-51.84	13.0	-0.0713	0.0115	48.17	18.23	0.00063	I88	clso4	c	-109.96	15.4	-0.02	0.01	0.00629
I88	clso4	h	19.50	1.9	0.0240	0.0014	15.51	2.93	0.00003	I88	clso4	h	35.26	6.1	0.01	0.00	0.00098
I88	clso4	n	95.73	5.3	0.0693	0.0033	4.88	1.12	0.00033	I88	clso4	n	128.18	18.9	0.05	0.01	0.00944
I88	scn	c	-67.19	4.1	-0.0704	0.0032	14.24	3.04	0.00016	I88	scn	c	-100.54	18.1	-0.04	0.01	0.00935
I88	scncl	c	-108.84	9.4	-0.0692	0.0070	20.26	6.30	0.00059	I88	scncl	c	-156.71	17.2	-0.03	0.01	0.00789
I88	scncl	h	-23.74	3.5	0.0232	0.0023	6.79	2.87	0.00014	I88	scncl	h	-11.63	6.7	0.01	0.00	0.00119
I88	scncl	n	-15.64	6.4	0.0843	0.0049	21.54	3.74	0.00027	I88	scncl	n	44.66	21.3	0.04	0.01	0.01202
I88	scn	h	-31.72	1.9	0.0195	0.0015	13.46	4.99	0.00004	I88	scn	h	-22.60	5.1	0.01	0.00	0.00075
I88	scn	n	-47.47	3.5	0.0760	0.0031	33.42	4.22	0.00008	I88	scn	n	-2.77	17.3	0.04	0.01	0.00859
I88	scnso4	c	-91.76	14.4	-0.0722	0.0122	38.96	16.37	0.00091	I88	scnso4	c	-154.49	17.3	-0.02	0.01	0.00826
I88	scnso4	h	-35.84	1.3	0.0298	0.0009	11.60	1.22	0.00002	I88	scnso4	h	-15.14	7.9	0.01	0.00	0.00174
I88	scnso4	n	-31.95	7.6	0.1051	0.0052	12.42	2.12	0.00052	I88	scnso4	n	41.39	27.4	0.05	0.01	0.02071
I88	so4	c	-61.74	9.9	-0.0453	0.0075	13.29	10.44	0.00079	I88	so4	c	-83.92	12.5	-0.03	0.01	0.00439
I88	so4	h	15.87	2.2	0.0087	0.0015	1.00	6.25	0.00006	I88	so4	h	18.70	3.0	0.01	0.00	0.00025
I88	so4	n	52.76	9.6	0.0961	0.0078	21.77	6.40	0.00059	I88	so4	n	106.46	23.2	0.05	0.01	0.01504
I96	cl	c	1.03	9.8	0.0102	0.0087	47.89	97.29	0.00035	I96	cl	c	9.60	4.6	0.00	0.00	0.00055
I96	cl	h	-60.15	5.4	0.0024	0.0038	11.64	73.56	0.00024	I96	cl	h	-58.69	3.2	0.00	0.00	0.00025
I96	cl	n	-32.84	14.4	-0.0990	0.0217	245.70	70.86	0.00011	I96	cl	n	-112.12	11.4	-0.01	0.01	0.00330
I96	clso4	c	15.07	17.0	0.0244	0.0106	5.75	11.41	0.00326	I96	clso4	c	27.01	12.8	0.02	0.01	0.00433
I96	clso4	h	-26.75	2.4	0.0046	0.0016	7.91	10.89	0.00006	I96	clso4	h	-24.14	2.0	0.00	0.00	0.00011
I96	clso4	n	4.81	15.1	-0.0484	0.0104	12.90	9.67	0.00192	I96	clso4	n	-25.93	15.3	-0.03	0.01	0.00621
I96	scn	c	-57.64	9.4	0.0303	0.0125	159.74	107.10	0.00013	I96	scn	c	-35.15	5.0	0.01	0.00	0.00072
I96	scncl	c	-70.21	4.1	0.0362	0.0028	10.91	3.11	0.00015	I96	scncl	c	-48.64	9.7	0.02	0.00	0.00250
I96	scncl	h	-117.64	20.2	-0.0699	0.0349	330.26	193.43	0.00013	I96	scncl	h	-171.77	6.3	-0.01	0.00	0.00104
I96	scncl	n	-139.78	20.3	-0.1316	0.0193	65.03	20.23	0.00119	I96	scncl	n	-249.40	25.2	-0.04	0.01	0.01686
I96	scn	h	-91.17	26.9	-0.0894	0.0747	757.00	555.69	0.00006	I96	scn	h	-139.94	4.0	-0.01	0.00	0.00045
I96	scn	n	-165.85	5.2	-0.0828	0.0050	52.06	8.13	0.00012	I96	scn	n	-219.46	17.4	-0.04	0.01	0.00863
I96	scnso4	c	-70.16	19.2	0.0451	0.0127	9.70	9.92	0.00365	I96	scnso4	c	-40.32	16.6	0.02	0.01	0.00760
I96	scnso4	h	-146.93	2.5	0.0044	0.0016	6.79	9.49	0.00007	I96	scnso4	h	-144.29	2.0	0.00	0.00	0.00011
I96	scnso4	n	-154.21	5.8	-0.0556	0.0037	7.47	1.90	0.00036	I96	scnso4	n	-187.82	14.6	-0.03	0.01	0.00591
I96	so4	c	-9.34	7.3	0.0418	0.0061	26.83	12.96	0.00031	I96	so4	c	15.36	10.2	0.02	0.01	0.00290
I96	so4	h	22.62	1.5	0.0091	0.0010	1.00	4.12	0.00003	I96	so4	h	25.57	2.9	0.01	0.00	0.00023
I96	so4	n	83.86	5.5	-0.0241	0.0044	20.79	14.28	0.00020	I96	so4	n	70.48	6.4	-0.01	0.00	0.00115
K108	cl	c	-75.32	24.3	0.0342	0.0396	292.92	417.32	0.00024	K108	cl	c	-47.91	4.9	0.00	0.00	0.00061
K108	cl	h	-51.91	5.6	0.0058	0.0047	34.35	74.31	0.00015	K108	cl	h	-47.42	2.9	0.00	0.00	0.00021
K108	cl	n	146.78	15.1	-0.1097	0.0231	252.19	69.13	0.00012	K108	cl	n	59.03	12.5	-0.01	0.01	0.00397
K108	clso4	c	-148.62	172.1	0.0878	0.5572	999.68	5021.81	0.00071	K108	clso4	c	-98.48	6.0	-0.00	0.00	0.00096
K108	clso4	h	-60.03	0.5	0.0055	0.0003	6.02	1.59	0.00000	K108	clso4	h	-57.28	1.5	0.00	0.00	0.00006
K108	clso4	n	446.28	84.8	-0.4943	0.1289	250.78	85.33	0.00385	K108	clso4	n	42.14	51.7	-0.06	0.02	0.07104
K108	scn	c	-232.03	58.7	-0.0358	0.1318	520.57	1930.59	0.00063	K108	scn	c	-252.50	4.6	-0.00	0.00	0.00061
K108	scncl	c	-288.18	277.0	-0.0199	0.8970	1000.00	35739.95	0.00186	K108	scncl	c	-308.22	6.6	0.01	0.00	0.00117
K108	scncl	h	-176.80	12.7	-0.0500	0.0165	170.88	84.96	0.00016	K108	scncl	h	-218.88	6.5	-0.01	0.00	0.00114
K108	scncl	n	466.95	24.3	-0.2372	0.0275	117.13	23.43	0.00093	K108	scncl	n	261.32	37.2	-0.05	0.02	0.03686
K108	scn	h	-157.12	7.5	-0.0469	0.0108	193.58	66.44	0.00006	K108	scn	h	-190.98	6.0	-0.01	0.00	0.00104
K108	scn	n	193.36	6.8	-0.1224	0.0068	60.02	8.10	0.00019	K108	scn	n	112.10	24.8	-0.05	0.01	0.01750
K108	scnso4	c	-101.11	201.3	-0.3525	0.6519	1000.00	1463.68	0.00097	K108	scnso4	c	-288.72	12.0	-0.01	0.00	0.00398
K108	scnso4	h	-248.92	2.1	0.0025	0.0011	1.00	3.68	0.00008	K108	scnso4	h	-248.17	1.7	0.00	0.00	0.00008

K108	scnso4	n	559.09	59.0	-0.4771	0.0786	182.82	44.30	0.00308	K108	scnso4	n	138.48	58.9	-0.06	0.02	0.09575
K108	so4	c	-43.12	195.8	0.0103	0.6304	1000.00	48152.44	0.00097	K108	so4	c	-32.72	5.3	-0.00	0.00	0.00080
K108	so4	h	-3.31	2.8	0.0077	0.0019	1.00	8.89	0.00009	K108	so4	h	-0.86	2.9	0.01	0.00	0.00023
K108	so4	n	31.36	43.0	-0.3908	0.0691	282.13	61.68	0.00081	K108	so4	n	-258.79	37.7	-0.06	0.02	0.03967
K19	cl	c	40.38	5.3	0.0079	0.0039	16.71	29.17	0.00020	K19	cl	c	45.01	3.4	0.00	0.00	0.00030
K19	cl	h	-37.53	5.7	0.0034	0.0044	21.99	89.90	0.00020	K19	cl	h	-35.07	3.0	0.00	0.00	0.00023
K19	cl	n	-89.96	7.6	0.0178	0.0057	18.28	19.82	0.00039	K19	cl	n	-78.73	6.3	0.01	0.00	0.00099
K19	clso4	c	34.08	15.7	0.0403	0.0202	167.10	127.62	0.00025	K19	clso4	c	69.68	6.5	0.01	0.00	0.00114
K19	clso4	h	-36.73	26.7	-0.0086	0.0638	612.36	4246.11	0.00006	K19	clso4	h	-43.06	1.6	0.00	0.00	0.00007
K19	clso4	n	-91.14	5.9	0.0160	0.0033	1.00	2.66	0.00051	K19	clso4	n	-86.44	6.2	0.01	0.00	0.00102
K19	scn	c	-15.04	68.6	0.1427	0.2264	1000.00	1272.97	0.00018	K19	scn	c	55.84	5.7	0.00	0.00	0.00094
K19	scncl	c	49.78	7.5	0.0260	0.0064	41.92	25.32	0.00023	K19	scncl	c	70.71	6.4	0.01	0.00	0.00109
K19	scncl	h	-17.45	90.8	-0.0848	0.2539	794.74	2026.18	0.00037	K19	scncl	h	-68.24	5.3	-0.00	0.00	0.00074
K19	scncl	n	-248.19	332.5	-0.0807	1.0767	1000.00	10555.36	0.00268	K19	scncl	n	-300.56	9.5	0.01	0.00	0.00242
K19	scn	h	-47.44	33.1	-0.0106	0.1091	1000.00	8262.92	0.00004	K19	scn	h	-53.47	1.3	0.00	0.00	0.00005
K19	scn	n	-229.05	86.9	-0.0802	0.2867	1000.00	2870.15	0.00028	K19	scn	n	-270.45	4.6	-0.00	0.00	0.00060
K19	scnso4	c	53.95	6.6	0.0293	0.0062	60.72	27.69	0.00013	K19	scnso4	c	80.87	6.5	0.01	0.00	0.00116
K19	scnso4	h	-48.36	42.8	-0.0191	0.1128	722.43	3761.20	0.00010	K19	scnso4	h	-61.71	2.1	0.00	0.00	0.00012
K19	scnso4	n	-327.32	10.2	0.0161	0.0055	1.00	2.71	0.00174	K19	scnso4	n	-322.47	8.7	0.01	0.00	0.00211
K19	so4	c	-87.99	139.3	0.2746	0.4484	1000.00	1282.85	0.00049	K19	so4	c	53.41	10.1	0.01	0.01	0.00283
K19	so4	h	-19.42	86.1	-0.0178	0.2771	1000.00	12225.73	0.00019	K19	so4	h	-30.96	2.5	0.00	0.00	0.00018
K19	so4	n	-115.27	5.8	0.0215	0.0039	1.00	6.67	0.00039	K19	so4	n	-108.38	7.4	0.02	0.00	0.00151
K27	cl	c	2.34	11.8	0.0048	0.0152	167.80	803.52	0.00014	K27	cl	c	6.84	2.5	0.00	0.00	0.00016
K27	cl	h	4.81	10.3	0.0682	0.0117	116.90	34.67	0.00017	K27	cl	h	62.46	12.2	0.01	0.01	0.00377
K27	cl	n	2.11	20.5	0.2269	0.0248	140.46	24.95	0.00053	K27	cl	n	191.43	35.3	0.05	0.02	0.03171
K27	clso4	c	-22.12	3.8	0.0427	0.0024	5.72	1.44	0.00016	K27	clso4	c	-1.12	11.6	0.03	0.01	0.00356
K27	clso4	h	32.18	5.4	0.1239	0.0035	9.19	1.02	0.00028	K27	clso4	h	102.70	32.3	0.07	0.01	0.02778
K27	clso4	n	121.23	18.2	0.2853	0.0129	14.86	2.24	0.00263	K27	clso4	n	306.64	72.3	0.14	0.03	0.13885
K27	scn	c	-51.82	3.0	0.0175	0.0026	35.72	16.33	0.00005	K27	scn	c	-41.22	4.3	0.01	0.00	0.00052
K27	scncl	c	-52.83	4.9	0.0117	0.0035	13.86	14.14	0.00020	K27	scncl	c	-44.98	4.3	0.01	0.00	0.00050
K27	scncl	h	7.11	4.7	0.0956	0.0034	17.10	1.99	0.00017	K27	scncl	h	71.41	24.2	0.05	0.01	0.01556
K27	scncl	n	-7.19	13.6	0.1868	0.0116	41.73	6.37	0.00077	K27	scncl	n	143.37	41.5	0.06	0.02	0.04584
K27	scn	h	8.75	2.4	0.0900	0.0022	41.08	2.89	0.00003	K27	scn	h	64.30	19.8	0.04	0.01	0.01119
K27	scn	n	-122.03	87.9	0.3765	0.2153	609.36	330.84	0.00100	K27	scn	n	108.19	24.3	0.02	0.01	0.01691
K27	scnso4	c	-68.94	8.1	0.0448	0.0049	4.37	2.10	0.00085	K27	scnso4	c	-45.26	13.3	0.03	0.01	0.00487
K27	scnso4	h	6.53	5.9	0.1375	0.0040	11.44	1.17	0.00032	K27	scnso4	h	101.27	36.3	0.07	0.02	0.03632
K27	scnso4	n	39.82	14.2	0.1991	0.0099	13.92	2.32	0.00169	K27	scnso4	n	182.28	51.2	0.09	0.02	0.07255
K27	so4	c	3.32	7.4	0.0747	0.0068	44.34	10.65	0.00023	K27	so4	c	52.83	15.8	0.03	0.01	0.00700
K27	so4	h	30.26	5.6	0.1317	0.0045	19.81	2.59	0.00022	K27	so4	h	102.19	31.9	0.07	0.02	0.02833
K27	so4	n	74.25	13.2	0.2491	0.0108	24.04	3.63	0.00107	K27	so4	n	217.24	58.6	0.13	0.03	0.09591
K39	cl	c	-29.31	77.0	0.1482	0.1637	496.43	551.17	0.00083	K39	cl	c	77.20	12.9	0.01	0.01	0.00424
K39	cl	h	-23.49	9.4	0.0470	0.0100	95.92	38.06	0.00017	K39	cl	h	15.90	9.2	0.01	0.00	0.00216
K39	cl	n	-21.60	5.5	-0.1310	0.0061	108.04	9.02	0.00005	K39	cl	n	-131.31	23.2	-0.03	0.01	0.01368
K39	clso4	h	-72.68	4.5	0.1456	0.0029	8.48	0.68	0.00020	K39	clso4	h	8.35	38.2	0.09	0.02	0.03878
K39	clso4	n	-303.58	30.1	0.1371	0.0180	3.28	2.53	0.01161	K39	clso4	n	-246.52	43.1	0.10	0.02	0.04941
K39	scn	c	5.78	7.3	-0.0604	0.0049	1.00	3.04	0.00069	K39	scn	c	-12.66	18.5	-0.05	0.01	0.00983
K39	scncl	c	-15.95	4.9	-0.0156	0.0027	1.00	2.25	0.00035	K39	scncl	c	-20.37	5.5	-0.01	0.00	0.00079
K39	scncl	h	-39.10	3.3	0.0638	0.0023	12.86	1.61	0.00009	K39	scncl	h	0.93	16.7	0.03	0.01	0.00742
K39	scncl	n	-174.33	17.2	-0.4105	0.0116	10.29	1.09	0.00278	K39	scncl	n	-414.26	106.6	-0.23	0.05	0.30256
K39	scn	h	-32.47	1.6	0.0636	0.0014	28.12	2.05	0.00002	K39	scn	h	3.46	14.9	0.03	0.01	0.00635
K39	scn	n	-151.15	7.4	-0.4513	0.0058	15.04	0.88	0.00050	K39	scn	n	-367.90	114.2	-0.28	0.06	0.37255
K39	scnso4	c	-60.70	13.0	0.0224	0.0070	1.00	2.48	0.00282	K39	scnso4	c	-53.88	11.4	0.02	0.00	0.00357
K39	scnso4	h	-62.32	5.7	0.0997	0.0035	5.23	0.77	0.00040	K39	scnso4	h	-6.38	27.4	0.06	0.01	0.02072
K39	scnso4	n	-238.27	8.8	-0.4408	0.0079	50.89	2.09	0.00028	K39	scnso4	n	-632.56	93.4	-0.12	0.04	0.24114
K49	cl	c	18.92	12.1	-0.0510	0.0146	138.87	64.79	0.00019	K49	cl	c	-23.67	8.4	-0.01	0.00	0.00178
K49	cl	h	-21.82	5.1	0.0068	0.0039	20.14	37.49	0.00017	K49	cl	h	-17.26	3.2	0.00	0.00	0.00027
K49	cl	n	-222.52	99.7	0.4163	0.2196	529.43	274.16	0.00121	K49	cl	n	55.86	26.1	0.03	0.01	0.01738

K49	clso4	c	-0.42	3.2	-0.0527	0.0022	12.24	1.82	0.00009	K49	clso4	c	-33.32	13.9	-0.03	0.01	0.00513
K49	clso4	h	-19.22	3.3	0.0145	0.0020	2.95	2.49	0.00014	K49	clso4	h	-13.40	4.6	0.01	0.00	0.00056
K49	clso4	n	-42.56	28.0	0.1505	0.0198	15.00	6.58	0.00618	K49	clso4	n	55.12	40.4	0.08	0.02	0.04330
K49	scn	c	68.54	4.8	-0.0376	0.0034	3.83	3.94	0.00028	K49	scn	c	55.24	11.1	-0.03	0.01	0.00351
K49	scncl	c	80.24	22.4	-0.0459	0.0182	31.82	33.64	0.00254	K49	scncl	c	41.19	16.3	-0.02	0.01	0.00707
K49	scncl	h	-74.03	4.2	0.0079	0.0026	5.44	8.39	0.00020	K49	scncl	h	-70.10	3.5	0.01	0.00	0.00033
K49	scncl	n	-247.34	8.8	0.1304	0.0054	4.78	0.98	0.00092	K49	scncl	n	-186.99	35.4	0.09	0.02	0.03337
K49	scn	h	-59.43	2.1	0.0142	0.0016	10.42	6.54	0.00005	K49	scn	h	-53.21	3.9	0.01	0.00	0.00045
K49	scn	n	-228.87	10.2	0.1251	0.0074	8.60	3.26	0.00109	K49	scn	n	-176.56	34.1	0.08	0.02	0.03319
K49	scnso4	c	84.82	6.3	-0.0441	0.0040	7.23	2.56	0.00045	K49	scnso4	c	57.48	12.8	-0.02	0.01	0.00450
K49	scnso4	h	-63.86	2.2	0.0192	0.0013	4.31	1.30	0.00006	K49	scnso4	h	-53.84	5.3	0.01	0.00	0.00077
K49	scnso4	n	-309.14	13.4	0.1630	0.0084	6.45	1.32	0.00207	K49	scnso4	n	-210.83	46.1	0.09	0.02	0.05866
K49	so4	c	-27.60	5.1	-0.0413	0.0039	12.43	5.76	0.00022	K49	so4	c	-47.60	10.9	-0.03	0.01	0.00334
K49	so4	h	2.16	2.7	-0.0385	0.0030	105.05	15.05	0.00001	K49	so4	h	-27.06	6.3	-0.01	0.00	0.00112
K49	so4	n	186.50	13.7	-0.0834	0.0101	9.71	6.71	0.00167	K49	so4	n	148.47	23.3	-0.05	0.01	0.01517
K62	cl	c	194.66	32.9	-0.2288	0.0811	643.44	208.59	0.00008	K62	cl	c	50.33	12.3	-0.01	0.01	0.00385
K62	cl	h	-13.76	6.8	0.0087	0.0064	61.35	98.78	0.00014	K62	cl	h	-6.52	3.1	0.00	0.00	0.00025
K62	cl	n	138.09	22.2	0.0864	0.0439	434.96	233.56	0.00009	K62	cl	n	199.48	6.5	0.01	0.00	0.00109
K62	clso4	c	295.53	222.6	-0.3711	0.7205	999.53	1535.50	0.00119	K62	clso4	c	93.23	14.8	-0.00	0.01	0.00583
K62	clso4	h	-56.15	23.9	0.1113	0.0704	864.85	452.72	0.00002	K62	clso4	h	5.95	3.9	0.00	0.00	0.00040
K62	clso4	n	275.01	5.0	0.0371	0.0032	6.46	2.40	0.00027	K62	clso4	n	293.97	10.3	0.02	0.00	0.00282
K62	scn	c	33.79	5.1	-0.1304	0.0080	243.69	20.32	0.00002	K62	scn	c	-61.21	15.6	-0.02	0.01	0.00692
K62	scncl	c	237.31	192.7	-0.3367	0.4921	684.28	896.27	0.00246	K62	scncl	c	17.12	20.6	-0.01	0.01	0.01126
K62	scncl	h	-125.73	15.2	-0.0988	0.0184	141.44	42.55	0.00029	K62	scncl	h	-210.30	14.0	-0.02	0.01	0.00520
K62	scncl	n	393.23	19.0	0.1361	0.0223	131.11	35.68	0.00050	K62	scncl	n	513.15	21.7	0.02	0.01	0.01249
K62	scn	h	-109.26	10.3	-0.1026	0.0132	142.40	31.16	0.00018	K62	scn	h	-183.69	15.4	-0.03	0.01	0.00676
K62	scn	n	264.20	11.7	0.1669	0.0155	157.49	23.86	0.00020	K62	scn	n	386.97	24.7	0.04	0.01	0.01738
K62	scnso4	c	205.72	200.7	-0.3619	0.5174	694.76	885.96	0.00255	K62	scnso4	c	-32.24	20.3	-0.00	0.01	0.01136
K62	scnso4	h	-147.79	4.9	-0.1160	0.0084	328.88	27.96	0.00001	K62	scnso4	h	-240.88	9.8	-0.01	0.00	0.00264
K62	scnso4	n	462.59	18.4	0.1115	0.0163	47.88	16.30	0.00126	K62	scnso4	n	557.60	22.3	0.03	0.01	0.01379
K62	so4	c	7.01	34.4	-0.0584	0.0599	336.82	398.76	0.00038	K62	so4	c	-37.03	6.8	-0.01	0.00	0.00131
K62	so4	h	17.96	3.1	0.0107	0.0025	21.47	18.55	0.00006	K62	so4	h	23.88	2.9	0.01	0.00	0.00024
K62	so4	n	71.73	8.3	0.0513	0.0071	31.34	13.39	0.00036	K62	so4	n	102.90	11.8	0.03	0.01	0.00389
K66	cl	h	-20.92	7.1	0.0100	0.0065	51.28	76.93	0.00018	K66	cl	h	-12.79	3.6	0.00	0.00	0.00033
K66	cl	n	-69.95	71.7	0.2200	0.2321	999.86	834.16	0.00012	K66	cl	n	43.59	7.3	0.01	0.00	0.00137
K66	clso4	c	113.54	30.5	0.1154	0.0302	75.07	39.63	0.00234	K66	clso4	c	212.29	23.1	0.03	0.01	0.01417
K66	clso4	h	-14.05	1.4	0.0123	0.0008	1.31	0.91	0.00003	K66	clso4	h	-10.11	3.6	0.01	0.00	0.00035
K66	clso4	n	8.43	24.8	0.1622	0.0237	65.59	20.23	0.00176	K66	clso4	n	144.06	30.8	0.05	0.01	0.02522
K66	scncl	c	323.28	24.5	-0.0382	0.0275	113.98	143.00	0.00098	K66	scncl	c	290.93	8.2	-0.01	0.00	0.00178
K66	scncl	h	-176.22	9.9	-0.0148	0.0117	133.16	173.35	0.00013	K66	scncl	h	-188.80	3.0	-0.00	0.00	0.00024
K66	scncl	n	24.41	34.2	0.1357	0.0613	357.75	184.11	0.00033	K66	scncl	n	130.47	12.4	0.01	0.01	0.00411
K66	scn	h	-155.85	4.4	-0.0238	0.0056	134.42	54.81	0.00003	K66	scn	h	-173.22	3.9	-0.01	0.00	0.00043
K66	scn	n	14.74	14.8	0.1708	0.0335	528.83	103.74	0.00004	K66	scn	n	122.16	11.4	0.01	0.01	0.00372
K66	scnso4	c	352.06	27.2	0.0531	0.0212	26.08	29.29	0.00426	K66	scnso4	c	396.91	18.6	0.02	0.01	0.00952
K66	scnso4	h	-244.73	3.2	0.0107	0.0019	3.68	3.03	0.00014	K66	scnso4	h	-239.36	3.7	0.01	0.00	0.00037
K66	scnso4	n	74.90	22.0	0.1789	0.0214	70.85	17.39	0.00129	K66	scnso4	n	234.93	32.9	0.04	0.01	0.02988
K66	so4	h	-7.77	2.2	0.0152	0.0015	1.00	3.63	0.00006	K66	so4	h	-2.85	4.7	0.01	0.00	0.00063
K66	so4	n	124.29	17.7	0.1645	0.0176	65.73	15.71	0.00091	K66	so4	n	240.92	31.1	0.06	0.02	0.02692
K98	cl	c	5.20	107.5	-0.0732	0.3473	997.53	3745.25	0.00028	K98	cl	c	-33.55	4.4	-0.00	0.00	0.00048
K98	cl	h	-19.02	5.9	0.0008	0.0049	33.26	560.79	0.00017	K98	cl	h	-18.11	2.6	0.00	0.00	0.00017
K98	cl	n	-63.97	7.5	-0.0616	0.0074	73.71	18.25	0.00014	K98	cl	n	-113.54	12.2	-0.02	0.01	0.00380
K98	clso4	c	26.21	5.9	-0.0072	0.0037	6.12	14.04	0.00038	K98	clso4	c	22.46	4.3	-0.00	0.00	0.00049
K98	clso4	h	1.71	2.0	-0.0076	0.0012	2.27	2.50	0.00005	K98	clso4	h	-1.10	2.5	-0.01	0.00	0.00017
K98	clso4	n	-78.19	14.8	-0.1512	0.0103	13.44	3.15	0.00181	K98	clso4	n	-173.55	38.8	-0.08	0.02	0.04006
K98	scn	c	13.57	7.2	-0.0208	0.0050	2.74	9.88	0.00064	K98	scn	c	6.56	7.6	-0.02	0.00	0.00167
K98	scncl	c	23.64	91.9	-0.0667	0.2176	601.89	1842.19	0.00077	K98	scncl	c	-21.75	6.7	-0.00	0.00	0.00119
K98	scncl	h	-55.57	4.6	-0.0154	0.0031	11.13	8.31	0.00019	K98	scncl	h	-64.50	4.6	-0.01	0.00	0.00055

K98	scncl	n	-157.24	15.7	-0.1583	0.0117	19.61	4.48	0.00169	K98	scncl	n	-266.09	39.0	-0.07	0.02	0.04047
K98	scn	h	-55.04	1.8	-0.0063	0.0013	4.98	9.38	0.00004	K98	scn	h	-57.39	2.1	-0.00	0.00	0.00013
K98	scn	n	-125.92	6.7	-0.1503	0.0060	36.42	4.35	0.00026	K98	scn	n	-216.15	33.8	-0.07	0.02	0.03261
K98	scnso4	c	-97.16	302.1	0.2021	0.9786	1000.00	3831.90	0.00219	K98	scnso4	c	17.31	11.0	-0.00	0.00	0.00335
K98	scnso4	h	-57.19	2.7	-0.0049	0.0015	2.42	4.04	0.00011	K98	scnso4	h	-59.33	2.4	-0.00	0.00	0.00015
K98	scnso4	n	-145.48	16.4	-0.1869	0.0120	18.55	3.70	0.00193	K98	scnso4	n	-286.69	46.3	-0.08	0.02	0.05925
K98	so4	c	-13.02	9.0	-0.0073	0.0061	1.00	30.46	0.00093	K98	so4	c	-15.36	6.2	-0.01	0.00	0.00106
K98	so4	h	14.52	3.9	-0.0089	0.0038	56.70	57.14	0.00005	K98	so4	h	8.21	2.3	-0.00	0.00	0.00015
K98	so4	n	-104.66	11.1	-0.1596	0.0095	30.24	5.60	0.00066	K98	so4	n	-201.60	36.2	-0.08	0.02	0.03662
L14	cl	c	-17.56	46.0	0.0347	0.1012	527.25	1512.68	0.00026	L14	cl	c	6.11	3.9	0.00	0.00	0.00040
L14	cl	h	-39.82	8.3	0.0207	0.0099	136.62	107.26	0.00009	L14	cl	h	-22.09	4.0	0.00	0.00	0.00040
L14	cl	n	-99.86	8.9	-0.0193	0.0068	20.25	23.17	0.00051	L14	cl	n	-112.11	6.7	-0.01	0.00	0.00114
L14	clso4	c	-7.09	5.1	0.0051	0.0032	5.06	14.96	0.00030	L14	clso4	c	-4.66	3.6	0.00	0.00	0.00035
L14	clso4	h	3.01	4.1	0.0160	0.0030	20.73	11.92	0.00011	L14	clso4	h	14.54	4.6	0.01	0.00	0.00056
L14	clso4	n	-51.66	34.8	-0.0325	0.0406	127.51	267.34	0.00172	L14	clso4	n	-83.68	10.3	-0.00	0.00	0.00281
L14	scn	c	-59.03	93.5	0.0784	0.3083	998.62	3151.15	0.00033	L14	scn	c	-17.56	4.8	0.00	0.00	0.00066
L14	scncl	c	-37.66	9.0	0.0199	0.0065	17.42	18.39	0.00059	L14	scncl	c	-23.51	7.3	0.01	0.00	0.00142
L14	scncl	h	-13.79	4.0	0.0123	0.0028	13.85	10.75	0.00013	L14	scncl	h	-5.70	4.0	0.01	0.00	0.00043
L14	scncl	n	80.25	208.9	0.0380	0.6766	1000.00	14081.99	0.00106	L14	scncl	n	101.10	6.4	0.00	0.00	0.00111
L14	scn	h	-47.12	4.1	0.0065	0.0031	12.95	31.25	0.00016	L14	scn	h	-44.08	2.9	0.00	0.00	0.00025
L14	scn	n	-82.40	141.1	-0.0405	0.4657	1000.00	9220.33	0.00075	L14	scn	n	-106.89	5.2	0.00	0.00	0.00078
L14	scnso4	c	-8.49	6.5	0.0099	0.0036	1.62	3.64	0.00066	L14	scnso4	c	-4.73	5.5	0.01	0.00	0.00084
L14	scnso4	h	-20.14	4.6	0.0360	0.0044	65.63	16.83	0.00006	L14	scnso4	h	12.56	7.2	0.01	0.00	0.00143
L14	scnso4	n	47.79	7.4	0.0158	0.0045	4.97	6.03	0.00069	L14	scnso4	n	56.68	6.7	0.01	0.00	0.00123
L14	so4	c	-13.26	30.8	0.0125	0.0413	179.54	878.21	0.00085	L14	so4	c	-1.90	6.0	0.00	0.00	0.00099
L14	so4	h	6.51	8.7	-0.0327	0.0113	161.80	85.94	0.00008	L14	so4	h	-19.63	5.2	-0.01	0.00	0.00075
L14	so4	n	-0.10	16.7	-0.1211	0.0183	95.10	27.59	0.00056	L14	so4	n	-92.66	22.0	-0.04	0.01	0.01352
L20	cl	h	-36.85	9.2	0.0196	0.0100	99.43	93.10	0.00016	L20	cl	h	-20.12	4.6	0.00	0.00	0.00053
L20	cl	n	45.34	4.8	0.0054	0.0029	1.00	11.41	0.00029	L20	cl	n	47.01	3.8	0.00	0.00	0.00036
L20	clso4	h	-51.82	3.2	0.0196	0.0030	64.05	21.16	0.00003	L20	clso4	h	-35.31	3.9	0.01	0.00	0.00040
L20	clso4	n	215.79	395.1	-0.3022	1.2794	1000.00	3349.40	0.00376	L20	clso4	n	44.78	16.0	0.00	0.01	0.00676
L20	scncl	h	-194.98	5.6	0.0117	0.0050	47.66	47.32	0.00012	L20	scncl	h	-185.23	3.3	0.00	0.00	0.00030
L20	scncl	n	109.01	185.5	-0.2364	0.6007	1000.00	2010.78	0.00084	L20	scncl	n	-20.26	10.0	-0.00	0.00	0.00268
L20	scn	h	-172.69	9.3	0.0319	0.0164	318.77	199.98	0.00004	L20	scn	h	-149.97	3.5	0.00	0.00	0.00036
L20	scn	n	-96.80	10.8	0.0043	0.0089	23.88	180.03	0.00087	L20	scn	n	-94.16	5.7	0.00	0.00	0.00092
L20	scnso4	h	-312.35	26.7	0.1136	0.0486	365.89	176.76	0.00019	L20	scnso4	h	-225.44	8.4	0.01	0.00	0.00194
L20	scnso4	n	45.97	347.4	-0.2862	1.1251	1000.00	3111.69	0.00289	L20	scnso4	n	-118.05	13.6	0.00	0.01	0.00510
L20	so4	h	-6.27	2.5	0.0106	0.0017	1.00	5.87	0.00007	L20	so4	h	-2.87	3.5	0.01	0.00	0.00035
L20	so4	n	-127.42	9.0	0.0158	0.0062	1.00	14.08	0.00095	L20	so4	n	-122.39	7.4	0.01	0.00	0.00155
L33	cl	c	5.68	9.7	0.0557	0.0105	100.90	34.73	0.00017	L33	cl	c	51.72	10.1	0.01	0.00	0.00261
L33	cl	h	-18.21	9.8	0.0273	0.0103	89.82	64.61	0.00020	L33	cl	h	4.82	6.1	0.01	0.00	0.00095
L33	cl	n	-30.05	5.7	0.1204	0.0076	184.47	17.05	0.00003	L33	cl	n	69.70	16.4	0.02	0.01	0.00688
L33	clso4	c	37.86	11.7	0.0619	0.0092	28.75	11.82	0.00074	L33	clso4	c	84.24	15.0	0.03	0.01	0.00601
L33	clso4	h	44.16	3.3	0.0235	0.0025	23.59	7.37	0.00007	L33	clso4	h	61.46	6.1	0.01	0.00	0.00097
L33	clso4	n	73.17	9.0	0.0668	0.0075	37.05	10.58	0.00037	L33	clso4	n	127.28	16.2	0.02	0.01	0.00698
L33	scn	c	31.13	23.0	0.0928	0.0466	424.37	231.90	0.00015	L33	scn	c	93.61	8.1	0.01	0.00	0.00188
L33	scncl	c	102.75	6.4	0.0386	0.0042	8.47	3.71	0.00042	L33	scncl	c	124.43	11.2	0.02	0.00	0.00333
L33	scncl	h	-21.45	3.3	0.0194	0.0023	12.16	5.12	0.00010	L33	scncl	h	-9.35	5.5	0.01	0.00	0.00081
L33	scncl	n	-2.91	15.3	0.0758	0.0144	61.62	25.20	0.00071	L33	scncl	n	61.90	16.4	0.02	0.01	0.00715
L33	scn	h	-22.47	2.5	0.0234	0.0026	72.69	17.92	0.00002	L33	scn	h	-6.17	4.8	0.01	0.00	0.00065
L33	scn	n	-41.23	11.9	0.1150	0.0180	223.62	49.27	0.00012	L33	scn	n	42.98	14.5	0.02	0.01	0.00601
L33	scnso4	c	74.46	24.1	0.0585	0.0177	18.84	17.56	0.00409	L33	scnso4	c	117.83	18.1	0.03	0.01	0.00909
L33	scnso4	h	-2.39	3.1	0.0221	0.0023	18.04	5.78	0.00007	L33	scnso4	h	14.86	6.1	0.01	0.00	0.00103
L33	scnso4	n	33.82	12.4	0.0625	0.0100	32.32	13.71	0.00076	L33	scnso4	n	87.68	16.1	0.02	0.01	0.00717
L33	so4	c	-44.25	62.0	0.1441	0.1025	300.36	257.66	0.00151	L33	so4	c	63.82	16.0	0.02	0.01	0.00716
L33	so4	h	1.09	1.7	0.0217	0.0013	16.22	4.19	0.00002	L33	so4	h	12.35	5.5	0.01	0.00	0.00083
L33	so4	n	-15.42	7.2	0.0568	0.0071	63.02	17.82	0.00015	L33	so4	n	24.55	10.9	0.02	0.01	0.00333

L42	cl	c	6.43	12.5	0.2752	0.0138	108.08	9.71	0.00027	L42	cl	c	235.57	47.8	0.07	0.02	0.05820
L42	cl	h	72.43	15.4	0.5861	0.0167	101.27	5.28	0.00044	L42	cl	h	559.73	104.7	0.14	0.05	0.27912
L42	cl	n	158.22	28.6	1.1630	0.0314	104.84	5.10	0.00146	L42	cl	n	1126.04	204.7	0.28	0.09	1.06564
L42	clso4	c	28.78	21.9	0.3515	0.0165	21.40	3.01	0.00313	L42	clso4	c	278.13	85.6	0.16	0.04	0.19440
L42	clso4	h	180.62	47.7	0.7321	0.0371	25.90	3.72	0.01319	L42	clso4	h	721.26	173.4	0.31	0.08	0.79852
L42	clso4	n	442.25	89.8	1.4071	0.0689	24.01	3.41	0.04915	L42	clso4	n	1465.45	337.2	0.61	0.15	3.01928
L42	scn	c	-49.29	22.3	0.5858	0.0182	22.09	2.59	0.00386	L42	scn	c	261.42	141.7	0.33	0.07	0.57322
L42	sncl	c	-17.73	17.3	0.5538	0.0117	11.06	0.86	0.00271	L42	sncl	c	313.47	143.7	0.31	0.06	0.54971
L42	sncl	h	94.20	31.2	1.1421	0.0210	10.25	0.71	0.00917	L42	sncl	h	762.47	297.4	0.64	0.13	2.35391
L42	sncl	n	325.66	69.9	2.1757	0.0471	10.50	0.85	0.04547	L42	sncl	n	1606.85	565.4	1.22	0.25	8.51075
L42	scn	h	29.01	14.4	1.1217	0.0112	16.42	0.72	0.00181	L42	scn	h	580.33	281.1	0.67	0.14	2.25752
L42	scn	n	176.37	36.1	2.1150	0.0284	17.32	0.99	0.01117	L42	scn	n	1230.38	526.8	1.26	0.27	7.92823
L42	scnso4	c	-35.44	11.6	0.6004	0.0083	16.18	0.72	0.00104	L42	scnso4	c	412.54	154.1	0.26	0.06	0.65580
L42	scnso4	h	15.49	25.9	1.2532	0.0188	17.54	0.83	0.00494	L42	scnso4	h	966.72	319.3	0.53	0.13	2.81618
L42	scnso4	n	219.14	48.7	2.3595	0.0352	17.37	0.82	0.01753	L42	scnso4	n	2005.97	601.3	1.00	0.25	9.98827
L42	so4	c	-8.33	17.5	0.3062	0.0170	60.01	7.75	0.00096	L42	so4	c	206.97	59.8	0.12	0.03	0.09977
L63	cl	h	-5.05	6.4	0.0128	0.0059	55.41	57.63	0.00013	L63	cl	h	5.27	3.7	0.00	0.00	0.00036
L63	cl	n	-34.39	12.9	0.0242	0.0186	219.81	231.88	0.00011	L63	cl	n	-15.02	3.5	0.00	0.00	0.00031
L63	clso4	h	2.69	1.1	0.0183	0.0007	4.77	0.89	0.00002	L63	clso4	h	11.23	5.0	0.01	0.00	0.00066
L63	clso4	n	-45.57	6.3	0.0228	0.0039	4.41	3.82	0.00048	L63	clso4	n	-35.28	7.4	0.02	0.00	0.00146
L63	sncl	h	36.97	5.6	0.0486	0.0051	51.11	12.23	0.00011	L63	sncl	h	77.56	10.6	0.02	0.00	0.00301
L63	sncl	n	-201.96	10.4	0.0602	0.0099	64.12	22.42	0.00032	L63	sncl	n	-150.11	12.8	0.02	0.01	0.00438
L63	scn	h	15.81	2.6	0.0778	0.0029	92.05	7.00	0.00002	L63	scn	h	71.17	14.1	0.03	0.01	0.00572
L63	scn	n	-197.30	4.6	0.0832	0.0048	73.49	9.54	0.00007	L63	scn	n	-139.74	16.3	0.03	0.01	0.00760
L63	scnso4	h	42.07	10.4	0.0649	0.0092	47.20	15.65	0.00041	L63	scnso4	h	97.66	13.3	0.02	0.01	0.00488
L63	scnso4	n	-261.57	12.8	0.0694	0.0107	37.27	14.50	0.00074	L63	scnso4	n	-203.13	15.7	0.02	0.01	0.00680
L63	so4	h	-5.46	1.7	0.0182	0.0012	2.91	2.66	0.00003	L63	so4	h	1.08	5.3	0.01	0.00	0.00080
L63	so4	n	-12.48	4.0	0.0336	0.0029	5.15	3.86	0.00016	L63	so4	n	0.80	9.6	0.02	0.00	0.00259
L89	cl	c	-0.58	17.6	0.0218	0.0204	123.52	196.77	0.00046	L89	cl	c	18.83	6.0	0.00	0.00	0.00091
L89	cl	h	4.13	93.7	-0.0303	0.3035	1000.00	7929.56	0.00021	L89	cl	h	-12.19	3.1	-0.00	0.00	0.00025
L89	cl	n	-62.17	4.9	-0.0486	0.0053	95.74	19.33	0.00005	L89	cl	n	-102.90	9.2	-0.01	0.00	0.00218
L89	clso4	c	26.81	7.0	0.0219	0.0046	9.33	7.57	0.00047	L89	clso4	c	39.32	7.1	0.01	0.00	0.00133
L89	clso4	h	6.15	1.6	-0.0059	0.0010	5.48	4.26	0.00003	L89	clso4	h	3.31	1.9	-0.00	0.00	0.00009
L89	clso4	n	-82.13	7.9	-0.1302	0.0048	4.20	0.81	0.00077	L89	clso4	n	-140.33	35.5	-0.09	0.02	0.03346
L89	scn	c	-29.13	8.7	0.0601	0.0124	189.53	59.11	0.00009	L89	scn	c	15.19	8.3	0.01	0.00	0.00199
L89	sncl	c	-18.34	7.5	0.0364	0.0053	14.19	7.02	0.00045	L89	sncl	c	5.01	10.2	0.02	0.00	0.00277
L89	sncl	h	-13.25	6.4	-0.0226	0.0054	39.69	23.56	0.00017	L89	sncl	h	-30.58	5.1	-0.01	0.00	0.00069
L89	sncl	n	-153.65	8.3	-0.1191	0.0057	10.92	1.92	0.00064	L89	sncl	n	-224.44	31.1	-0.07	0.01	0.02569
L89	scn	h	-22.97	1.9	-0.0087	0.0014	6.16	7.79	0.00004	L89	scn	h	-26.33	2.7	-0.01	0.00	0.00020
L89	scn	n	-114.61	4.5	-0.1003	0.0037	23.02	3.13	0.00015	L89	scn	n	-168.35	24.1	-0.06	0.01	0.01663
L89	scnso4	c	-3.67	6.6	0.0334	0.0045	12.17	5.65	0.00039	L89	scnso4	c	20.08	9.8	0.02	0.00	0.00266
L89	scnso4	h	-21.33	2.2	-0.0047	0.0013	4.20	5.14	0.00006	L89	scnso4	h	-23.83	2.0	-0.00	0.00	0.00011
L89	scnso4	n	-131.62	10.1	-0.1497	0.0063	6.05	1.03	0.00120	L89	scnso4	n	-217.62	39.6	-0.09	0.02	0.04329
L89	so4	c	-42.82	63.9	0.0986	0.1106	332.74	432.69	0.00133	L89	so4	c	28.99	11.0	0.01	0.01	0.00339
L89	so4	h	19.57	6.0	-0.0090	0.0064	85.47	121.07	0.00008	L89	so4	h	12.67	2.4	-0.00	0.00	0.00016
L89	so4	n	-70.07	8.1	-0.1168	0.0061	10.68	2.99	0.00058	L89	so4	n	-124.49	30.7	-0.07	0.02	0.02637
L95	cl	c	-92.40	6.8	0.0065	0.0061	51.27	112.93	0.00016	L95	cl	c	-87.30	2.9	0.00	0.00	0.00022
L95	cl	h	-18.63	7.1	0.0067	0.0064	49.88	111.92	0.00018	L95	cl	h	-13.04	3.2	0.00	0.00	0.00026
L95	cl	n	38.23	8.5	0.0292	0.0082	68.16	40.42	0.00020	L95	cl	n	61.30	6.3	0.01	0.00	0.00100
L95	clso4	c	-123.78	33.8	-0.0563	0.0526	264.51	316.05	0.00056	L95	clso4	c	-168.81	6.9	-0.01	0.00	0.00127
L95	clso4	h	-21.21	1.7	0.0052	0.0011	3.68	4.12	0.00004	L95	clso4	h	-18.99	1.8	0.00	0.00	0.00009
L95	clso4	n	39.53	25.9	0.0607	0.0329	161.46	134.95	0.00071	L95	clso4	n	90.31	8.8	0.01	0.00	0.00208
L95	scn	c	-109.05	6.3	-0.0106	0.0052	23.79	42.18	0.00029	L95	scn	c	-114.83	4.1	-0.01	0.00	0.00048
L95	sncl	c	-52.93	143.6	-0.2071	0.4646	998.69	1773.58	0.00050	L95	sncl	c	-163.44	8.2	-0.00	0.00	0.00180
L95	sncl	h	-21.83	52.7	-0.0522	0.1707	999.20	2583.12	0.00007	L95	sncl	h	-49.50	2.3	-0.00	0.00	0.00014
L95	sncl	n	37.70	8.8	0.0131	0.0070	29.11	42.50	0.00041	L95	sncl	n	48.15	5.2	0.00	0.00	0.00073
L95	scn	h	-38.80	4.8	0.0013	0.0062	149.48	1200.32	0.00004	L95	scn	h	-37.48	1.1	0.00	0.00	0.00004

L95	scn	n	-20.67	95.5	0.0785	0.3152	999.34	3220.06	0.00034	L95	scn	n	20.61	4.9	0.00	0.00	0.00068
L95	scnso4	c	-55.22	86.7	-0.1962	0.1986	567.33	550.54	0.00078	L95	scnso4	c	-190.74	12.2	-0.01	0.01	0.00411
L95	scnso4	h	-68.30	5.1	0.0116	0.0049	68.84	60.73	0.00007	L95	scnso4	h	-58.06	2.6	0.00	0.00	0.00019
L95	scnso4	n	-121.85	55.1	0.1784	0.1062	412.19	265.02	0.00064	L95	scnso4	n	9.86	12.1	0.01	0.00	0.00403
L95	so4	c	-111.29	23.1	-0.0503	0.0379	297.33	271.44	0.00021	L95	so4	c	-147.89	5.1	-0.01	0.00	0.00073
L95	so4	h	-14.36	3.0	0.0067	0.0020	1.00	11.01	0.00010	L95	so4	h	-12.21	2.8	0.01	0.00	0.00021
L95	so4	n	14.96	6.6	0.0452	0.0055	26.35	10.66	0.00025	L95	so4	n	41.43	10.8	0.02	0.01	0.00324
N23	cl	c	-52.20	4.0	0.0110	0.0029	16.18	15.28	0.00011	N23	cl	c	-45.56	3.7	0.01	0.00	0.00034
N23	cl	h	-14.17	8.7	0.0223	0.0088	79.34	62.28	0.00018	N23	cl	h	4.62	5.4	0.01	0.00	0.00074
N23	cl	n	-28.62	11.3	0.0847	0.0146	167.89	44.06	0.00013	N23	cl	n	41.79	12.3	0.01	0.01	0.00383
N23	clso4	c	-5.75	272.2	-0.0645	0.8814	1000.00	10819.27	0.00178	N23	clso4	c	-47.73	7.8	0.00	0.00	0.00160
N23	clso4	h	-7.84	2.4	0.0376	0.0020	33.81	4.60	0.00003	N23	clso4	h	21.58	8.7	0.01	0.00	0.00200
N23	clso4	n	-63.27	6.4	0.0937	0.0050	26.01	3.92	0.00024	N23	clso4	n	7.08	23.1	0.04	0.01	0.01413
N23	scn	c	-11.20	72.5	-0.0526	0.2392	1000.00	3648.90	0.00020	N23	scn	c	-35.88	2.8	-0.00	0.00	0.00022
N23	scncl	c	-53.41	10.5	0.0115	0.0060	1.21	7.00	0.00162	N23	scncl	c	-49.80	8.4	0.01	0.00	0.00190
N23	scncl	h	89.54	5.9	0.0245	0.0044	20.14	11.16	0.00024	N23	scncl	h	106.73	6.8	0.01	0.00	0.00122
N23	scncl	n	183.96	7.5	0.0458	0.0057	23.97	8.77	0.00034	N23	scncl	n	218.24	12.4	0.02	0.01	0.00407
N23	scn	h	63.11	9.7	0.0592	0.0146	218.17	76.59	0.00009	N23	scn	h	106.00	7.4	0.01	0.00	0.00156
N23	scn	n	153.90	38.0	0.0921	0.0817	478.00	440.61	0.00032	N23	scn	n	215.10	8.1	0.01	0.00	0.00189
N23	scnso4	c	-29.13	7.8	0.0096	0.0042	1.00	3.46	0.00101	N23	scnso4	c	-26.10	6.5	0.01	0.00	0.00117
N23	scnso4	h	121.12	6.0	0.0432	0.0049	34.42	10.15	0.00017	N23	scnso4	h	157.34	9.9	0.01	0.00	0.00269
N23	scnso4	n	197.14	8.6	0.0735	0.0066	23.25	6.08	0.00046	N23	scnso4	n	255.93	18.5	0.03	0.01	0.00945
N23	so4	c	-30.01	4.1	-0.0170	0.0028	1.00	5.95	0.00020	N23	so4	c	-35.51	5.7	-0.01	0.00	0.00091
N23	so4	h	22.70	3.9	0.0299	0.0038	61.29	17.99	0.00005	N23	so4	h	43.77	5.9	0.01	0.00	0.00097
N23	so4	n	-49.32	6.9	0.0889	0.0065	50.83	9.28	0.00018	N23	so4	n	11.57	18.4	0.04	0.01	0.00944
N41	cl	c	91.40	21.3	0.0135	0.0165	22.69	86.39	0.00273	N41	cl	c	100.52	11.0	0.01	0.01	0.00308
N41	cl	h	-16.11	9.8	0.0366	0.0107	101.99	54.33	0.00018	N41	cl	h	15.02	7.4	0.01	0.00	0.00141
N41	cl	n	50.84	10.5	0.1642	0.0140	183.82	22.97	0.00010	N41	cl	n	186.82	22.5	0.03	0.01	0.01283
N41	clso4	c	69.80	9.3	0.0597	0.0062	10.16	3.96	0.00081	N41	clso4	c	104.37	16.0	0.03	0.01	0.00678
N41	clso4	h	-40.71	3.5	0.0973	0.0023	9.60	0.86	0.00011	N41	clso4	h	15.61	25.5	0.06	0.01	0.01731
N41	clso4	n	44.25	13.8	0.2979	0.0098	15.01	1.65	0.00151	N41	clso4	n	238.83	75.6	0.15	0.03	0.15159
N41	scn	c	-21.58	4.0	0.1160	0.0032	21.40	2.28	0.00012	N41	scn	c	39.52	28.2	0.07	0.01	0.02274
N41	scncl	c	-21.89	10.3	0.1183	0.0069	10.64	2.33	0.00098	N41	scncl	c	48.38	31.6	0.07	0.01	0.02654
N41	scncl	h	-22.32	4.2	0.0041	0.0030	13.13	33.28	0.00015	N41	scncl	h	-19.47	2.7	0.00	0.00	0.00020
N41	scncl	n	154.41	8.6	0.2701	0.0059	12.47	0.98	0.00064	N41	scncl	n	321.52	69.7	0.14	0.03	0.12920
N41	scn	h	-14.76	1.5	-0.0107	0.0010	1.00	3.57	0.00003	N41	scn	h	-18.00	3.3	-0.01	0.00	0.00031
N41	scn	n	94.33	6.7	0.2915	0.0055	21.56	1.55	0.00036	N41	scn	n	248.19	70.8	0.16	0.04	0.14306
N41	scnso4	c	-6.25	2.8	0.1238	0.0019	12.54	0.68	0.00007	N41	scnso4	c	81.20	32.6	0.06	0.01	0.02930
N41	scnso4	h	-40.97	6.6	0.0274	0.0036	1.72	1.39	0.00068	N41	scnso4	h	-30.22	8.9	0.02	0.00	0.00217
N41	scnso4	n	99.99	8.9	0.3660	0.0062	13.40	0.76	0.00067	N41	scnso4	n	362.25	95.7	0.17	0.04	0.25285
N41	so4	h	14.24	12.3	0.1684	0.0112	42.06	7.53	0.00065	N41	so4	h	124.48	35.8	0.07	0.02	0.03570
N41	so4	n	53.77	32.3	0.5038	0.0311	56.22	8.27	0.00349	N41	so4	n	402.87	99.4	0.20	0.05	0.27578
N58	cl	c	37.24	6.5	0.0177	0.0052	28.85	24.28	0.00022	N58	cl	c	49.38	5.2	0.01	0.00	0.00068
N58	cl	h	-91.80	24.4	0.1277	0.0364	240.65	90.95	0.00034	N58	cl	h	12.02	15.7	0.02	0.01	0.00631
N58	cl	n	-335.02	106.1	1.4074	0.2300	513.37	83.28	0.00147	N58	cl	n	625.18	92.7	0.09	0.04	0.21847
N58	clso4	c	45.69	9.3	0.0534	0.0057	4.90	2.56	0.00101	N58	clso4	c	70.47	15.3	0.04	0.01	0.00620
N58	clso4	h	-241.50	795.4	0.4028	2.5756	1000.00	5059.24	0.01524	N58	clso4	h	0.49	26.0	-0.01	0.01	0.01792
N58	clso4	n	-993.95	927.0	2.8780	2.9942	996.34	821.03	0.02091	N58	clso4	n	542.81	99.3	0.05	0.04	0.26183
N58	scn	c	118.06	20.3	0.2329	0.0276	167.24	31.50	0.00055	N58	scn	c	288.89	33.2	0.05	0.02	0.03152
N58	scncl	c	200.32	19.3	0.1512	0.0185	66.19	16.98	0.00106	N58	scncl	c	327.99	29.4	0.04	0.01	0.02309
N58	scncl	h	-253.55	6.0	-0.0174	0.0034	1.00	2.48	0.00052	N58	scncl	h	-258.52	6.4	-0.01	0.00	0.00109
N58	scncl	n	-572.21	45.1	0.7481	0.0499	109.02	12.89	0.00351	N58	scncl	n	95.50	131.0	0.15	0.06	0.45660
N58	scn	h	-188.35	4.5	-0.0173	0.0031	1.27	6.77	0.00027	N58	scn	h	-193.73	5.9	-0.01	0.00	0.00101
N58	scn	n	-375.60	48.2	0.6387	0.0638	156.62	25.56	0.00338	N58	scn	n	99.99	98.4	0.15	0.05	0.27660
N58	scnso4	c	265.12	19.1	0.1388	0.0155	32.59	9.60	0.00181	N58	scnso4	c	379.15	31.0	0.05	0.01	0.02657
N58	scnso4	h	-233.94	6.4	-0.0348	0.0034	1.00	0.79	0.00069	N58	scnso4	h	-245.19	10.3	-0.03	0.00	0.00294
N58	scnso4	n	-514.16	201.1	0.6758	0.3003	240.80	141.95	0.02329	N58	scnso4	n	94.72	86.5	0.04	0.04	0.20658

N58	so4	c	24.12	11.6	0.0733	0.0096	24.52	11.00	0.00082	N58	so4	c	66.26	17.8	0.04	0.01	0.00885
N58	so4	h	-12.91	621.9	0.1145	2.0018	1000.00	13729.45	0.00980	N58	so4	h	66.52	17.2	-0.01	0.01	0.00824
N58	so4	n	110.77	1048.2	0.3442	3.3741	1000.00	7699.59	0.02783	N58	so4	n	317.22	33.1	-0.01	0.02	0.03061
N5	cl	c	-0.93	85.8	-0.0885	0.2777	1000.00	2482.04	0.00018	N5	cl	c	-46.80	3.9	-0.00	0.00	0.00039
N5	cl	h	-24.28	5.7	0.0029	0.0045	27.24	124.33	0.00018	N5	cl	h	-22.03	2.8	0.00	0.00	0.00020
N5	cl	n	144.08	10.9	-0.0364	0.0108	73.58	44.78	0.00030	N5	cl	n	114.37	8.2	-0.01	0.00	0.00169
N5	clso4	c	-57.43	6.9	-0.0223	0.0043	5.29	4.83	0.00055	N5	clso4	c	-68.05	7.4	-0.01	0.00	0.00146
N5	clso4	h	-1.59	5.2	-0.0065	0.0050	64.29	104.16	0.00008	N5	clso4	h	-7.26	2.2	-0.00	0.00	0.00013
N5	clso4	n	231.12	9.9	-0.0751	0.0075	22.03	6.56	0.00063	N5	clso4	n	178.17	18.1	-0.03	0.01	0.00874
N5	scn	c	-56.53	1.9	-0.0178	0.0016	25.89	7.90	0.00002	N5	scn	c	-66.32	4.3	-0.01	0.00	0.00052
N5	scncl	c	12.35	60.1	-0.1766	0.1583	720.83	568.84	0.00021	N5	scncl	c	-95.89	8.4	-0.01	0.00	0.00187
N5	scncl	h	-61.85	10.2	-0.0103	0.0124	144.22	277.63	0.00013	N5	scncl	h	-70.00	2.3	-0.00	0.00	0.00014
N5	scncl	n	46.14	2.6	-0.0569	0.0015	2.04	0.41	0.00009	N5	scncl	n	25.83	15.8	-0.04	0.01	0.00667
N5	scn	h	-54.18	2.2	-0.0010	0.0015	1.00	56.51	0.00006	N5	scn	h	-54.45	1.5	-0.00	0.00	0.00006
N5	scn	n	-27.18	6.8	-0.0349	0.0046	1.00	4.93	0.00060	N5	scn	n	-37.81	11.3	-0.03	0.01	0.00364
N5	scnso4	c	-92.47	12.6	-0.0220	0.0083	9.40	12.96	0.00160	N5	scnso4	c	-107.18	9.7	-0.01	0.00	0.00261
N5	scnso4	h	-94.16	82.0	0.0486	0.2657	1000.00	4325.25	0.00016	N5	scnso4	h	-67.94	2.9	0.00	0.00	0.00022
N5	scnso4	n	-37.32	5.0	-0.0449	0.0029	3.03	0.96	0.00035	N5	scnso4	n	-58.21	12.3	-0.03	0.01	0.00419
N5	so4	c	-43.72	6.7	-0.0163	0.0046	1.00	10.20	0.00053	N5	so4	c	-48.99	6.5	-0.01	0.00	0.00118
N5	so4	h	23.60	7.1	-0.0187	0.0086	133.10	101.95	0.00007	N5	so4	h	8.79	3.4	-0.00	0.00	0.00032
N5	so4	n	44.22	10.8	-0.0134	0.0102	53.02	98.95	0.00041	N5	so4	n	34.58	4.9	-0.01	0.00	0.00066
N77	cl	c	38.24	9.3	0.0539	0.0086	56.37	20.18	0.00028	N77	cl	c	79.60	11.3	0.02	0.01	0.00325
N77	cl	h	-18.99	5.7	0.0033	0.0045	26.02	104.39	0.00018	N77	cl	h	-16.47	2.9	0.00	0.00	0.00021
N77	cl	n	83.41	11.9	-0.0759	0.0145	144.07	44.15	0.00017	N77	cl	n	18.59	12.7	-0.01	0.01	0.00410
N77	clso4	c	39.10	15.2	0.0818	0.0130	40.84	15.95	0.00098	N77	clso4	c	104.41	18.6	0.03	0.01	0.00914
N77	clso4	h	-4.17	1.7	-0.0020	0.0010	1.00	6.15	0.00004	N77	clso4	h	-4.70	1.4	-0.00	0.00	0.00005
N77	clso4	n	163.16	17.2	-0.1222	0.0113	9.09	3.27	0.00289	N77	clso4	n	94.48	32.8	-0.07	0.01	0.02856
N77	scn	c	-3.74	19.7	0.0981	0.0311	247.00	106.00	0.00029	N77	scn	c	67.22	11.8	0.02	0.01	0.00399
N77	scncl	c	40.29	8.0	0.0582	0.0062	24.16	7.44	0.00039	N77	scncl	c	82.79	14.6	0.03	0.01	0.00571
N77	scncl	h	-49.56	3.6	-0.0019	0.0021	1.00	14.02	0.00019	N77	scncl	h	-50.02	2.7	-0.00	0.00	0.00020
N77	scncl	n	345.83	15.7	-0.0676	0.0097	4.72	3.37	0.00296	N77	scncl	n	314.80	20.8	-0.04	0.01	0.01155
N77	scn	h	-49.56	2.1	0.0094	0.0021	66.74	35.24	0.00002	N77	scn	h	-43.13	2.0	0.00	0.00	0.00012
N77	scn	n	216.77	2.9	-0.0588	0.0021	5.77	1.71	0.00010	N77	scn	n	194.30	16.3	-0.04	0.01	0.00761
N77	scnso4	c	68.25	6.8	0.0591	0.0039	17.17	3.28	0.00016	N77	scnso4	c	129.25	21.8	0.02	0.01	0.00650
N77	scnso4	h	-123.07	43.5	0.1502	0.1409	1000.00	742.68	0.00005	N77	scnso4	h	-43.28	4.6	0.00	0.00	0.00058
N77	scnso4	n	384.40	7.1	-0.0686	0.0043	5.61	1.47	0.00060	N77	scnso4	n	345.57	18.8	-0.04	0.01	0.00972
N77	so4	c	-18.99	33.9	0.1133	0.0449	173.28	103.02	0.00108	N77	so4	c	68.13	15.8	0.02	0.01	0.00696
N77	so4	h	-0.33	34.3	0.0142	0.1104	1000.00	6103.70	0.00003	N77	so4	h	6.47	1.0	0.00	0.00	0.00003
N77	so4	n	28.94	7.1	-0.0917	0.0059	27.80	5.84	0.00028	N77	so4	n	-25.77	21.3	-0.05	0.01	0.01265
N84	cl	c	5.41	30.2	-0.1285	0.0440	227.37	105.53	0.00057	N84	cl	c	-98.66	16.1	-0.02	0.01	0.00656
N84	cl	h	0.58	12.0	-0.0408	0.0141	130.02	75.34	0.00020	N84	cl	h	-32.89	6.7	-0.01	0.00	0.00116
N84	cl	n	-69.47	11.0	-0.4174	0.0114	87.45	4.61	0.00026	N84	cl	n	-413.09	78.5	-0.11	0.04	0.15681
N84	clso4	c	21.02	4.3	-0.1182	0.0029	9.47	0.88	0.00018	N84	clso4	c	-46.94	30.9	-0.07	0.01	0.02529
N84	clso4	h	-0.51	2.4	-0.0426	0.0021	45.17	5.41	0.00002	N84	clso4	h	-35.48	9.3	-0.01	0.00	0.00231
N84	clso4	n	-155.75	25.6	-0.4690	0.0197	24.39	2.96	0.00396	N84	clso4	n	-498.76	112.6	-0.20	0.05	0.33668
N84	scn	c	-200.84	4.8	-0.0922	0.0044	42.74	5.79	0.00012	N84	scn	c	-258.20	20.1	-0.04	0.01	0.01159
N84	scncl	c	-163.17	24.1	-0.1425	0.0217	51.58	17.92	0.00204	N84	scncl	c	-277.93	28.6	-0.05	0.01	0.02180
N84	scncl	h	13.99	3.1	-0.0551	0.0019	5.35	0.88	0.00011	N84	scncl	h	-12.40	14.8	-0.04	0.01	0.00582
N84	scncl	n	-154.88	13.5	-0.6938	0.0090	9.31	0.47	0.00179	N84	scncl	n	-550.38	182.1	-0.40	0.08	0.88265
N84	scn	h	-156.68	2.8	-0.1513	0.0023	19.19	1.16	0.00007	N84	scn	h	-234.17	37.3	-0.09	0.02	0.03966
N84	scn	n	11.56	14.9	-0.9582	0.0120	20.39	1.00	0.00177	N84	scn	n	-486.97	234.2	-0.55	0.12	1.56671
N84	scnso4	c	-228.91	10.7	-0.1014	0.0072	11.62	2.89	0.00104	N84	scnso4	c	-298.13	26.5	-0.05	0.01	0.01946
N84	scnso4	h	14.58	2.3	-0.0415	0.0015	9.88	1.32	0.00005	N84	scnso4	h	-12.95	11.0	-0.02	0.00	0.00336
N84	scnso4	n	-96.66	24.3	-0.7147	0.0173	15.90	1.24	0.00458	N84	scnso4	n	-632.72	187.6	-0.31	0.08	0.97285
N84	so4	c	71.82	10.4	-0.1084	0.0087	25.22	6.84	0.00065	N84	so4	c	8.91	25.6	-0.06	0.01	0.01825
N84	so4	h	9.68	4.1	0.0071	0.0028	1.00	14.12	0.00019	N84	so4	h	11.93	3.3	0.01	0.00	0.00031
N84	so4	n	-13.67	5.6	-0.1946	0.0049	34.63	2.55	0.00015	N84	so4	n	-136.10	43.1	-0.09	0.02	0.05177

P21	cl	c	20.38	62.7	0.0654	0.1510	619.72	1327.64	0.00033	P21	cl	c	64.87	5.6	0.00	0.00	0.00081
P21	clso4	c	-84.12	27.8	0.2472	0.0580	478.04	114.42	0.00012	P21	clso4	c	93.65	17.0	0.01	0.01	0.00768
P21	scn	c	-17.78	138.7	0.0505	0.4579	1000.00	7281.22	0.00072	P21	scn	c	11.99	5.3	-0.00	0.00	0.00081
P21	scncl	c	6.93	11.5	0.0242	0.0093	30.62	31.71	0.00069	P21	scncl	c	26.01	7.9	0.01	0.00	0.00167
P21	scnso4	c	-43.56	19.8	0.0816	0.0224	117.52	55.67	0.00062	P21	scnso4	c	29.50	12.7	0.01	0.01	0.00448
P21	so4	c	-59.69	62.9	0.2991	0.1641	716.81	343.87	0.00024	P21	so4	c	116.68	13.9	0.01	0.01	0.00537
P47	cl	c	-40.91	5.8	0.0119	0.0050	40.94	43.65	0.00014	P47	cl	c	-32.22	3.5	0.00	0.00	0.00032
P47	clso4	c	-73.15	4.8	0.0313	0.0047	74.56	22.73	0.00006	P47	clso4	c	-46.32	6.0	0.01	0.00	0.00095
P47	scn	c	-220.64	5.4	0.0070	0.0036	1.00	19.23	0.00037	P47	scn	c	-218.54	4.1	0.01	0.00	0.00049
P47	scncl	c	-274.10	10.7	0.0223	0.0061	1.00	3.49	0.00170	P47	scncl	c	-267.46	10.1	0.02	0.00	0.00274
P47	scnso4	c	-306.08	6.9	0.0159	0.0040	3.38	4.09	0.00066	P47	scnso4	c	-298.31	6.5	0.01	0.00	0.00117
P47	so4	c	-27.49	36.4	0.0362	0.0598	297.16	594.76	0.00053	P47	so4	c	1.39	6.1	0.00	0.00	0.00104
P64	cl	c	-19.51	224.6	-0.0867	0.7267	999.39	6627.60	0.00122	P64	cl	c	-71.07	7.5	0.00	0.00	0.00145
P64	clso4	c	-72.19	12.8	-0.0324	0.0094	18.09	16.50	0.00118	P64	clso4	c	-95.17	11.0	-0.01	0.00	0.00320
P64	scn	c	-65.98	29.5	-0.0684	0.0294	60.67	63.26	0.00350	P64	scn	c	-112.68	18.4	-0.03	0.01	0.00962
P64	scncl	c	-156.64	20.5	-0.0255	0.0161	27.58	48.90	0.00233	P64	scncl	c	-177.66	11.9	-0.01	0.01	0.00377
P64	scnso4	c	-127.42	15.5	-0.0374	0.0117	21.64	20.04	0.00156	P64	scnso4	c	-158.90	12.9	-0.01	0.01	0.00461
P64	so4	c	-11.62	18.4	-0.0490	0.0171	47.42	42.44	0.00131	P64	so4	c	-44.34	12.0	-0.02	0.01	0.00405
Q104	cl	c	-19.22	13.4	0.0543	0.0160	136.38	66.14	0.00023	Q104	cl	c	26.31	9.1	0.01	0.00	0.00213
Q104	cl	h	-12.28	6.0	0.0029	0.0048	28.44	137.02	0.00019	Q104	cl	h	-10.00	2.9	0.00	0.00	0.00021
Q104	cl	n	169.55	16.4	-0.1506	0.0221	186.45	39.94	0.00023	Q104	cl	n	44.66	20.8	-0.02	0.01	0.01097
Q104	clso4	c	-63.36	14.5	0.0871	0.0096	9.65	4.06	0.00201	Q104	clso4	c	-13.40	23.9	0.05	0.01	0.01513
Q104	clso4	h	1.88	2.5	0.0024	0.0014	1.00	7.45	0.00009	Q104	clso4	h	2.61	2.0	0.00	0.00	0.00010
Q104	clso4	n	250.56	13.6	-0.0980	0.0098	16.42	5.33	0.00140	Q104	clso4	n	185.13	25.5	-0.05	0.01	0.01731
Q104	scn	c	-54.49	7.4	0.1032	0.0070	47.84	8.71	0.00027	Q104	scn	c	11.11	22.0	0.05	0.01	0.01388
Q104	scncl	c	-35.66	5.1	0.0878	0.0037	15.93	2.21	0.00020	Q104	scncl	c	22.31	22.3	0.04	0.01	0.01329
Q104	scncl	h	-12.20	10.1	-0.0202	0.0120	133.77	130.99	0.00014	Q104	scncl	h	-29.40	3.6	-0.00	0.00	0.00034
Q104	scncl	n	275.51	28.1	-0.2054	0.0225	29.45	8.82	0.00421	Q104	scncl	n	123.38	46.9	-0.09	0.02	0.05846
Q104	scn	h	-26.35	1.7	-0.0050	0.0011	1.00	8.43	0.00004	Q104	scn	h	-27.89	1.9	-0.00	0.00	0.00010
Q104	scn	n	140.59	14.1	-0.2051	0.0142	63.01	10.44	0.00078	Q104	scn	n	3.54	40.9	-0.08	0.02	0.04774
Q104	scnso4	c	-75.23	13.9	0.1070	0.0093	10.93	3.37	0.00181	Q104	scnso4	c	-2.61	29.1	0.05	0.01	0.02333
Q104	scnso4	h	-35.78	102.3	0.0231	0.3312	1000.00	11344.31	0.00025	Q104	scnso4	h	-23.62	3.1	0.00	0.00	0.00026
Q104	scnso4	n	173.95	11.5	-0.1098	0.0079	12.29	3.04	0.00118	Q104	scnso4	n	98.95	27.8	-0.05	0.01	0.02133
Q104	so4	c	-9.47	5.4	0.0772	0.0041	12.63	3.25	0.00024	Q104	so4	c	28.11	20.0	0.05	0.01	0.01114
Q104	so4	h	-0.60	2.3	0.0068	0.0016	1.00	8.31	0.00006	Q104	so4	h	1.60	2.5	0.01	0.00	0.00018
Q104	so4	n	146.37	6.0	-0.0520	0.0046	14.99	5.88	0.00028	Q104	so4	n	119.83	13.5	-0.03	0.01	0.00508
Q15	cl	c	47.01	4.3	0.0068	0.0027	4.23	11.92	0.00020	Q15	cl	c	49.80	3.5	0.00	0.00	0.00030
Q15	cl	h	-28.06	5.6	0.0019	0.0050	45.08	279.94	0.00012	Q15	cl	h	-26.30	2.3	0.00	0.00	0.00013
Q15	cl	n	-97.39	4.3	-0.0467	0.0043	78.35	14.60	0.00004	Q15	cl	n	-135.52	9.2	-0.01	0.00	0.00217
Q15	clso4	c	27.79	21.3	0.1004	0.0276	169.72	70.43	0.00044	Q15	clso4	c	113.28	13.3	0.02	0.01	0.00471
Q15	clso4	h	-30.65	2.5	0.0026	0.0014	1.00	7.17	0.00009	Q15	clso4	h	-29.90	2.0	0.00	0.00	0.00011
Q15	clso4	n	159.83	84.8	-0.3760	0.1555	373.85	173.11	0.00184	Q15	clso4	n	-126.02	30.9	-0.03	0.01	0.02529
Q15	scn	c	-77.71	43.9	0.0996	0.1047	579.84	588.97	0.00028	Q15	scn	c	-15.59	7.3	0.01	0.00	0.00153
Q15	scncl	c	-10.78	11.4	0.0227	0.0095	37.65	40.04	0.00058	Q15	scncl	c	7.27	6.9	0.01	0.00	0.00128
Q15	scncl	h	44.39	129.2	-0.2009	0.4185	1000.00	1647.73	0.00041	Q15	scncl	h	-63.04	7.8	-0.00	0.00	0.00161
Q15	scncl	n	141.36	1059.2	-1.1037	3.4282	999.10	2456.07	0.02730	Q15	scncl	n	-428.28	44.6	-0.03	0.02	0.05294
Q15	scn	h	-63.22	2.3	-0.0024	0.0016	1.00	23.91	0.00007	Q15	scn	h	-63.95	1.7	-0.00	0.00	0.00008
Q15	scn	n	-159.23	24.3	-0.1415	0.0256	75.01	30.12	0.00197	Q15	scn	n	-255.56	27.3	-0.05	0.01	0.02129
Q15	scnso4	c	-69.09	21.4	0.1026	0.0242	116.61	47.57	0.00073	Q15	scnso4	c	21.10	14.8	0.02	0.01	0.00604
Q15	scnso4	h	-96.22	1.5	0.0046	0.0010	10.13	7.73	0.00002	Q15	scnso4	h	-93.00	1.6	0.00	0.00	0.00007
Q15	scnso4	n	236.04	420.2	-1.0485	1.3608	999.75	1027.05	0.00424	Q15	scnso4	n	-321.97	33.4	-0.02	0.01	0.03089
Q15	so4	c	35.82	38.5	0.1231	0.0770	445.17	288.87	0.00027	Q15	so4	c	121.12	9.5	0.01	0.00	0.00254
Q15	so4	h	4.50	106.1	-0.0247	0.3415	1000.00	10874.16	0.00029	Q15	so4	h	-11.17	3.1	0.00	0.00	0.00028
Q15	so4	n	237.68	96.4	-0.6267	0.3102	999.85	388.79	0.00024	Q15	so4	n	-81.69	20.0	-0.02	0.01	0.01113
Q2	clso4	c	-279.34	937.8	1.0123	3.0366	1000.00	2373.22	0.02118	Q2	clso4	c	262.28	44.1	0.02	0.02	0.05156
Q2	scn	c	-152.77	3.3	-0.0035	0.0022	1.00	23.76	0.00014	Q2	scn	c	-153.78	2.4	-0.00	0.00	0.00017
Q2	scncl	c	-203.38	25.3	-0.0494	0.0175	12.26	15.50	0.00560	Q2	scncl	c	-233.86	19.3	-0.03	0.01	0.00996

Q2	scnso4	c	599.15	1146.2	-0.7165	3.3785	998.41	3412.87	0.00696	Q2	scnso4	c	157.94	32.6	-0.00	0.01	0.01461
Q31	cl	c	47.58	13.9	0.1803	0.0171	147.19	22.31	0.00023	Q31	cl	c	198.50	27.8	0.03	0.01	0.01960
Q31	cl	h	-1.51	10.0	0.0569	0.0112	111.48	38.66	0.00017	Q31	cl	h	46.32	10.3	0.01	0.00	0.00271
Q31	cl	n	29.47	24.9	0.1702	0.0423	320.18	94.61	0.00021	Q31	cl	n	161.30	17.0	0.02	0.01	0.00735
Q31	clso4	c	99.00	17.8	0.2165	0.0132	19.50	3.66	0.00218	Q31	clso4	c	248.77	53.1	0.10	0.02	0.07493
Q31	clso4	h	1.25	4.6	0.0762	0.0032	12.15	1.78	0.00018	Q31	clso4	h	48.12	19.6	0.04	0.01	0.01015
Q31	clso4	n	8.18	7.3	0.1905	0.0049	9.62	0.93	0.00051	Q31	clso4	n	118.05	49.6	0.11	0.02	0.06523
Q31	scn	c	39.61	10.7	0.2125	0.0101	47.36	6.04	0.00056	Q31	scn	c	174.44	45.3	0.09	0.02	0.05854
Q31	scncl	c	70.54	13.5	0.2245	0.0098	17.11	2.41	0.00135	Q31	scncl	c	221.07	56.5	0.11	0.02	0.08483
Q31	scncl	h	-13.93	5.6	0.1029	0.0040	15.59	2.01	0.00024	Q31	scncl	h	53.61	26.2	0.05	0.01	0.01824
Q31	scncl	n	70.80	22.4	0.3426	0.0179	29.35	4.21	0.00269	Q31	scncl	n	329.73	80.5	0.14	0.03	0.17239
Q31	scn	h	-18.63	4.1	0.1214	0.0035	31.22	2.93	0.00011	Q31	scn	h	51.65	28.0	0.06	0.01	0.02233
Q31	scn	n	50.83	21.0	0.3987	0.0185	34.55	4.94	0.00268	Q31	scn	n	286.80	90.2	0.20	0.05	0.23225
Q31	scnso4	c	71.45	14.0	0.2293	0.0099	15.58	2.19	0.00154	Q31	scnso4	c	240.42	58.9	0.10	0.02	0.09577
Q31	scnso4	h	-35.66	7.5	0.1288	0.0054	16.27	2.18	0.00043	Q31	scnso4	h	60.07	32.9	0.06	0.01	0.02985
Q31	scnso4	n	19.21	21.6	0.3886	0.0158	18.26	2.31	0.00337	Q31	scnso4	n	313.74	97.0	0.16	0.04	0.25971
Q31	so4	c	55.55	13.3	0.2019	0.0121	42.89	6.89	0.00074	Q31	so4	c	188.40	42.7	0.09	0.02	0.05096
Q31	so4	h	18.35	3.1	0.0726	0.0025	21.56	2.76	0.00006	Q31	so4	h	58.95	17.4	0.04	0.01	0.00844
Q31	so4	n	72.30	16.1	0.3022	0.0130	21.53	3.40	0.00169	Q31	so4	n	240.89	72.3	0.17	0.04	0.14579
R110	cl	h	-7.25	7.8	0.0171	0.0074	63.58	59.34	0.00018	R110	cl	h	6.80	4.7	0.01	0.00	0.00055
R110	cl	n	289.91	183.0	0.3727	0.5918	998.37	1254.24	0.00081	R110	cl	n	485.42	14.4	0.01	0.01	0.00526
R110	clso4	h	5.77	1.7	0.0105	0.0011	6.63	2.90	0.00003	R110	clso4	h	11.30	3.1	0.01	0.00	0.00025
R110	clso4	n	-169.36	76.9	1.5027	0.1721	544.04	60.60	0.00068	R110	clso4	n	853.84	87.0	0.08	0.04	0.20085
R110	scncl	h	-33.98	3.8	0.0099	0.0026	11.68	11.18	0.00013	R110	scncl	h	-27.79	3.5	0.01	0.00	0.00033
R110	scncl	n	117.53	410.6	0.8193	1.3276	997.52	1279.91	0.00412	R110	scncl	n	560.21	31.2	0.01	0.01	0.02588
R110	scn	h	-37.17	2.7	0.0151	0.0025	45.46	20.82	0.00004	R110	scn	h	-27.42	3.6	0.01	0.00	0.00037
R110	scn	n	255.70	189.1	0.2167	0.6243	1000.00	2312.33	0.00134	R110	scn	n	368.04	11.4	0.00	0.01	0.00371
R110	scnso4	h	-48.44	2.4	0.0164	0.0016	11.98	4.11	0.00005	R110	scnso4	h	-36.89	4.6	0.01	0.00	0.00059
R110	scnso4	n	-207.07	57.7	1.0291	0.1096	400.81	46.59	0.00074	R110	scnso4	n	576.49	73.9	0.06	0.03	0.15070
R110	so4	h	7.44	3.0	0.0108	0.0020	1.00	6.75	0.00010	R110	so4	h	10.93	3.7	0.01	0.00	0.00039
R110	so4	n	-52.27	87.9	1.4486	0.2162	648.31	87.56	0.00060	R110	so4	n	828.54	70.0	0.08	0.04	0.13672
R59	cl	c	117.22	37.1	0.0879	0.0706	402.61	351.95	0.00030	R59	cl	c	180.97	7.6	0.01	0.00	0.00149
R59	cl	h	-10.10	7.1	0.0041	0.0048	8.67	47.58	0.00046	R59	cl	h	-7.92	4.4	0.00	0.00	0.00050
R59	cl	n	357.36	609.1	-0.1561	1.9717	1000.00	9986.22	0.00896	R59	cl	n	261.34	19.0	0.01	0.01	0.00919
R59	clso4	c	171.75	17.7	0.1097	0.0146	34.70	11.98	0.00150	R59	clso4	c	255.61	24.3	0.04	0.01	0.01570
R59	clso4	h	66.38	49.5	-0.0629	0.0916	381.25	616.82	0.00060	R59	clso4	h	15.16	7.7	-0.00	0.00	0.00156
R59	clso4	n	451.87	104.7	-0.0683	0.1060	81.40	248.31	0.02555	R59	clso4	n	386.99	34.6	-0.01	0.01	0.03177
R59	scn	c	-180.57	347.8	0.4556	1.1480	1000.00	2022.31	0.00454	R59	scn	c	49.54	21.8	0.01	0.01	0.01355
R59	scncl	c	31.57	11.3	0.0889	0.0116	86.36	21.71	0.00028	R59	scncl	c	108.62	16.2	0.02	0.01	0.00699
R59	scncl	h	-37.74	7.5	-0.0907	0.0082	101.52	16.60	0.00011	R59	scncl	h	-117.31	15.7	-0.02	0.01	0.00655
R59	scncl	n	126.02	24.5	-0.2009	0.0312	163.24	38.81	0.00062	R59	scncl	n	-47.17	27.5	-0.03	0.01	0.02016
R59	scn	h	-66.91	8.4	-0.0902	0.0079	47.74	11.24	0.00034	R59	scn	h	-123.86	19.1	-0.04	0.01	0.01042
R59	scn	n	97.91	60.1	-0.3737	0.1074	328.48	113.84	0.00166	R59	scn	n	-167.92	39.3	-0.05	0.02	0.04402
R59	scnso4	c	6.94	14.3	0.1085	0.0113	27.88	8.04	0.00114	R59	scnso4	c	95.16	25.8	0.04	0.01	0.01838
R59	scnso4	h	-60.70	13.3	-0.0919	0.0163	147.68	41.56	0.00021	R59	scnso4	h	-146.72	14.3	-0.01	0.01	0.00562
R59	scnso4	n	-46.50	24.9	-0.1538	0.0196	27.18	9.62	0.00349	R59	scnso4	n	-179.13	42.3	-0.05	0.02	0.04938
R59	so4	c	85.86	12.3	0.1336	0.0124	70.06	14.16	0.00041	R59	so4	c	181.64	24.7	0.05	0.01	0.01706
R59	so4	h	2.42	1.4	0.0167	0.0010	9.82	3.46	0.00002	R59	so4	h	10.06	4.5	0.01	0.00	0.00056
R59	so4	n	52.46	7.3	0.1370	0.0062	29.79	4.26	0.00029	R59	so4	n	135.46	31.1	0.07	0.02	0.02705
R69	cl	c	-44.86	112.0	-0.1520	0.3625	999.89	1885.50	0.00030	R69	cl	c	-123.43	6.0	-0.00	0.00	0.00091
R69	cl	h	-60.33	7.3	0.0088	0.0065	48.12	84.66	0.00019	R69	cl	h	-53.17	3.6	0.00	0.00	0.00032
R69	cl	n	-73.08	31.2	0.1323	0.0618	433.77	214.23	0.00018	R69	cl	n	20.86	9.9	0.01	0.00	0.00251
R69	clso4	c	-235.42	5.3	-0.0454	0.0035	10.26	2.99	0.00026	R69	clso4	c	-262.46	12.5	-0.03	0.01	0.00414
R69	clso4	h	-91.58	1.9	0.0028	0.0011	1.00	4.97	0.00005	R69	clso4	h	-90.77	1.6	0.00	0.00	0.00007
R69	clso4	n	-42.01	8.2	0.0276	0.0055	10.81	7.93	0.00061	R69	clso4	n	-25.12	8.9	0.01	0.00	0.00212
R69	scn	c	-312.27	27.6	-0.0718	0.0219	18.23	23.04	0.00640	R69	scn	c	-348.71	23.3	-0.04	0.01	0.01557
R69	scncl	c	-433.53	10.9	-0.0467	0.0085	26.03	13.52	0.00069	R69	scncl	c	-469.10	13.0	-0.02	0.01	0.00450

R69	scncl	h	-211.58	5.0	-0.0079	0.0041	34.80	47.54	0.00012	R69	scncl	h	-217.30	2.5	-0.00	0.00	0.00017
R69	scncl	n	219.36	8.0	0.0790	0.0069	44.16	9.29	0.00025	R69	scncl	n	283.98	17.8	0.03	0.01	0.00840
R69	scn	h	-155.64	3.5	-0.0122	0.0037	71.23	48.39	0.00004	R69	scn	h	-163.96	2.6	-0.00	0.00	0.00020
R69	scn	n	174.27	9.3	0.1423	0.0106	98.08	14.45	0.00022	R69	scn	n	275.97	25.3	0.04	0.01	0.01831
R69	scnso4	c	-563.14	10.3	-0.0658	0.0068	9.74	3.65	0.00105	R69	scnso4	c	-606.17	18.0	-0.03	0.01	0.00897
R69	scnso4	h	-270.06	80.7	0.0499	0.2615	1000.00	4149.76	0.00016	R69	scnso4	h	-244.01	2.7	0.00	0.00	0.00020
R69	scnso4	n	337.41	7.2	0.0523	0.0054	21.17	6.57	0.00035	R69	scnso4	n	379.29	14.0	0.02	0.01	0.00540
R69	so4	c	-163.88	8.0	-0.0505	0.0061	14.42	7.89	0.00050	R69	so4	c	-189.20	13.3	-0.03	0.01	0.00493
R69	so4	h	-27.33	93.7	-0.0396	0.3015	999.71	5985.68	0.00022	R69	so4	h	-50.00	3.2	0.00	0.00	0.00028
R69	so4	n	-71.41	5.8	0.0275	0.0041	5.67	6.97	0.00033	R69	so4	n	-60.33	8.3	0.02	0.00	0.00194
R72	cl	c	24.37	78.9	-0.0093	0.2553	1000.00	21655.63	0.00015	R72	cl	c	19.09	2.5	0.00	0.00	0.00015
R72	cl	h	11.00	122.9	-0.0413	0.3977	1000.00	7607.46	0.00036	R72	cl	h	-10.97	4.1	-0.00	0.00	0.00042
R72	cl	n	211.04	186.5	-0.5173	0.6036	999.77	922.40	0.00084	R72	cl	n	-56.93	18.0	-0.01	0.01	0.00820
R72	clso4	c	17.17	9.6	0.0117	0.0082	40.50	70.28	0.00040	R72	clso4	c	27.11	4.8	0.00	0.00	0.00062
R72	clso4	h	2.38	66.1	0.0258	0.2141	1000.00	6573.91	0.00011	R72	clso4	h	17.82	2.1	-0.00	0.00	0.00012
R72	clso4	n	103.53	20.8	-0.0779	0.0145	13.16	8.46	0.00365	R72	clso4	n	52.74	24.4	-0.04	0.01	0.01577
R72	scn	c	-37.45	106.4	0.0958	0.3507	998.24	2933.66	0.00043	R72	scn	c	12.91	5.7	0.00	0.00	0.00092
R72	scncl	c	-7.15	9.8	0.0147	0.0076	25.51	37.84	0.00056	R72	scncl	c	4.73	6.3	0.01	0.00	0.00104
R72	scncl	h	4.44	12.6	-0.0169	0.0177	208.84	306.82	0.00012	R72	scncl	h	-9.23	2.7	-0.00	0.00	0.00019
R72	scncl	n	30.38	21.2	-0.1446	0.0291	197.40	56.73	0.00036	R72	scncl	n	-92.20	17.9	-0.02	0.01	0.00853
R72	scn	h	-5.46	3.5	0.0011	0.0036	69.45	512.71	0.00004	R72	scn	h	-4.43	1.3	0.00	0.00	0.00005
R72	scn	n	-75.68	6.0	-0.0616	0.0057	50.29	12.29	0.00017	R72	scn	n	-115.43	13.3	-0.03	0.01	0.00503
R72	scnso4	c	-360.33	362.3	0.6253	1.1733	999.55	1484.60	0.00315	R72	scnso4	c	-32.39	20.4	0.01	0.01	0.01153
R72	scnso4	h	-84.53	112.0	0.1079	0.3626	1000.00	2659.20	0.00030	R72	scnso4	h	-26.42	4.7	0.00	0.00	0.00061
R72	scnso4	n	30.47	32.5	-0.1004	0.0252	25.07	17.90	0.00628	R72	scnso4	n	-52.30	29.4	-0.04	0.01	0.02393
R72	so4	c	-1.80	18.9	0.0285	0.0219	114.22	155.77	0.00058	R72	so4	c	20.82	6.8	0.01	0.00	0.00128
R72	so4	h	3.30	2.4	0.0027	0.0017	1.00	22.37	0.00007	R72	so4	h	4.15	1.7	0.00	0.00	0.00009
R72	so4	n	22.57	5.1	-0.0212	0.0035	2.09	6.34	0.00029	R72	so4	n	15.25	7.0	-0.02	0.00	0.00136
R83	cl	c	22.76	9.5	0.1159	0.0098	85.28	14.06	0.00020	R83	cl	c	118.06	22.2	0.03	0.01	0.01252
R83	cl	h	87.89	15.2	0.5405	0.0166	104.30	5.79	0.00041	R83	cl	h	537.66	95.4	0.13	0.04	0.23147
R83	cl	n	368.05	47.4	1.8740	0.0525	107.99	5.41	0.00388	R83	cl	n	1928.83	325.6	0.45	0.15	2.69763
R83	clso4	c	-9.45	12.6	0.2275	0.0096	23.15	2.86	0.00098	R83	clso4	c	154.83	54.8	0.10	0.02	0.07969
R83	clso4	h	123.32	18.8	0.7827	0.0145	21.29	1.19	0.00171	R83	clso4	h	817.49	211.2	0.24	0.11	1.07999
R83	clso4	n	697.87	116.7	2.4121	0.0906	23.15	2.66	0.06398	R83	clso4	n	2852.29	645.4	0.71	0.33	10.08632
R83	scn	c	-60.90	5.4	0.0970	0.0052	51.64	7.13	0.00013	R83	scn	c	1.76	20.3	0.04	0.01	0.01183
R83	scncl	c	-66.10	9.8	0.1374	0.0068	12.95	2.26	0.00082	R83	scncl	c	19.74	35.6	0.07	0.02	0.03379
R83	scncl	h	71.95	12.0	0.5222	0.0081	10.78	0.63	0.00134	R83	scncl	h	382.16	135.7	0.29	0.06	0.49031
R83	scncl	n	358.42	50.8	1.6274	0.0344	10.75	0.84	0.02385	R83	scncl	n	1323.60	422.8	0.90	0.18	4.75770
R83	scn	h	34.74	5.8	0.3703	0.0047	20.60	1.01	0.00027	R83	scn	h	227.89	90.3	0.21	0.05	0.23315
R83	scn	n	195.15	26.5	0.9857	0.0218	23.39	1.90	0.00528	R83	scn	n	726.08	236.5	0.54	0.12	1.59733
R83	scnso4	c	-97.29	8.4	0.1306	0.0056	10.55	1.61	0.00067	R83	scnso4	c	-7.80	35.8	0.06	0.01	0.03546
R83	scnso4	h	-15.28	7.6	0.5456	0.0052	11.93	0.39	0.00052	R83	scnso4	h	366.43	144.5	0.26	0.06	0.57709
R83	scnso4	n	123.00	25.8	1.5776	0.0178	12.86	0.49	0.00578	R83	scnso4	n	1242.98	413.3	0.74	0.17	4.71988
R83	so4	c	6.67	14.5	0.3454	0.0155	85.70	7.69	0.00047	R83	so4	c	263.04	60.7	0.11	0.03	0.10270
R87	cl	c	-86.29	9.1	0.0025	0.0080	45.97	358.33	0.00031	R87	cl	c	-83.54	3.6	0.00	0.00	0.00033
R87	cl	h	1.91	82.2	-0.0441	0.2661	1000.00	4773.92	0.00016	R87	cl	h	-20.08	2.7	-0.00	0.00	0.00019
R87	cl	n	43.52	5.0	-0.0055	0.0039	23.03	50.54	0.00015	R87	cl	n	39.72	2.9	-0.00	0.00	0.00021
R87	clso4	c	-111.79	237.7	-0.0194	0.7696	1000.00	31391.90	0.00136	R87	clso4	c	-128.94	6.2	0.00	0.00	0.00102
R87	clso4	h	3.73	1.9	-0.0213	0.0012	7.33	1.75	0.00004	R87	clso4	h	-7.60	5.7	-0.01	0.00	0.00087
R87	clso4	n	35.51	170.1	0.0750	0.5506	1000.00	5810.86	0.00070	R87	clso4	n	77.91	5.8	-0.00	0.00	0.00088
R87	scn	c	-99.94	3.6	-0.0174	0.0024	1.00	5.19	0.00017	R87	scn	c	-105.26	5.7	-0.01	0.00	0.00093
R87	scncl	c	-164.80	4.7	-0.0241	0.0031	8.91	4.54	0.00022	R87	scncl	c	-178.14	6.7	-0.01	0.00	0.00121
R87	scncl	h	-32.10	4.1	-0.0179	0.0029	12.79	7.24	0.00015	R87	scncl	h	-43.02	5.0	-0.01	0.00	0.00066
R87	scncl	n	-9.72	182.3	0.2622	0.5903	1000.00	1780.95	0.00081	R87	scncl	n	133.67	10.8	0.00	0.00	0.00308
R87	scn	h	-33.32	1.7	-0.0074	0.0012	2.24	6.43	0.00004	R87	scn	h	-35.74	2.4	-0.01	0.00	0.00017
R87	scn	n	-7.18	72.8	0.2162	0.2402	1000.00	891.48	0.00020	R87	scn	n	100.64	8.4	0.01	0.00	0.00202
R87	scnso4	c	-95.46	29.5	-0.0564	0.0339	123.41	126.06	0.00129	R87	scnso4	c	-149.67	11.9	-0.01	0.00	0.00389

R87	scnso4	h	-22.76	2.6	-0.0169	0.0016	4.67	1.87	0.00009	R87	scnso4	h	-31.89	4.9	-0.01	0.00	0.00066
R87	scnso4	n	25.72	28.3	0.1186	0.0380	186.06	87.14	0.00069	R87	scnso4	n	133.28	16.4	0.01	0.01	0.00743
R87	so4	c	-96.87	6.8	0.0164	0.0046	1.00	10.27	0.00054	R87	so4	c	-91.58	6.6	0.01	0.00	0.00120
R87	so4	h	9.24	3.7	-0.0218	0.0035	49.47	19.95	0.00005	R87	so4	h	-5.64	4.7	-0.01	0.00	0.00063
R87	so4	n	25.26	31.2	0.0759	0.0535	325.04	267.69	0.00033	R87	so4	n	80.17	7.3	0.01	0.00	0.00147
S28	cl	c	29.44	11.6	0.1222	0.0144	149.09	27.86	0.00016	S28	cl	c	132.23	19.0	0.02	0.01	0.00923
S28	cl	h	-6.51	9.2	0.0362	0.0100	102.59	51.48	0.00015	S28	cl	h	24.05	7.1	0.01	0.00	0.00130
S28	cl	n	9.01	6.4	-0.1019	0.0077	141.32	17.30	0.00005	S28	cl	n	-76.39	16.0	-0.02	0.01	0.00651
S28	clso4	c	28.45	20.4	0.1664	0.0160	27.19	7.31	0.00234	S28	clso4	c	151.28	38.9	0.07	0.02	0.04013
S28	clso4	h	5.02	4.1	0.0510	0.0028	10.45	2.10	0.00016	S28	clso4	h	35.05	13.3	0.03	0.01	0.00469
S28	clso4	n	15.33	9.0	-0.1053	0.0063	14.96	3.01	0.00063	S28	clso4	n	-53.15	26.8	-0.05	0.01	0.01910
S28	scn	c	-86.00	17.8	0.1903	0.0189	77.85	16.91	0.00102	S28	scn	c	45.21	35.9	0.07	0.02	0.03676
S28	scncl	c	-82.18	16.1	0.1876	0.0130	31.39	5.84	0.00132	S28	scncl	c	60.74	43.3	0.07	0.02	0.04984
S28	scncl	h	-61.86	3.2	0.0305	0.0025	28.21	6.47	0.00006	S28	scncl	h	-38.68	7.5	0.01	0.00	0.00151
S28	scncl	n	-2.57	20.7	-0.1924	0.0148	15.86	4.05	0.00329	S28	scncl	n	-127.69	48.2	-0.10	0.02	0.06196
S28	scn	h	-55.68	1.7	0.0347	0.0017	71.81	8.15	0.00001	S28	scn	h	-31.79	6.8	0.01	0.00	0.00133
S28	scn	n	-36.73	13.1	-0.1728	0.0115	34.05	7.04	0.00105	S28	scn	n	-138.51	39.3	-0.09	0.02	0.04410
S28	scnso4	c	-100.11	19.6	0.1869	0.0149	22.66	5.29	0.00242	S28	scnso4	c	46.90	45.6	0.07	0.02	0.05748
S28	scnso4	h	-84.84	4.2	0.0518	0.0028	11.33	2.15	0.00016	S28	scnso4	h	-49.48	13.6	0.03	0.01	0.00510
S28	scnso4	n	-51.04	10.6	-0.1391	0.0073	13.28	2.35	0.00095	S28	scnso4	n	-149.26	35.8	-0.07	0.01	0.03535
S28	so4	c	62.18	14.3	0.1472	0.0127	39.03	9.43	0.00092	S28	so4	c	156.96	32.0	0.07	0.02	0.02854
S28	so4	h	21.91	4.5	0.0505	0.0034	12.88	4.16	0.00016	S28	so4	h	46.56	13.1	0.03	0.01	0.00477
S28	so4	n	64.53	6.4	-0.0523	0.0054	27.87	9.31	0.00023	S28	so4	n	33.08	12.5	-0.03	0.01	0.00438
S38	cl	c	32.75	6.8	0.2363	0.0074	102.19	5.83	0.00008	S38	cl	c	229.44	42.2	0.06	0.02	0.04530
S38	cl	h	-44.71	7.9	-0.0686	0.0074	59.89	14.24	0.00019	S38	cl	h	-98.44	14.3	-0.02	0.01	0.00522
S38	cl	n	-10.93	21.1	-0.3309	0.0166	25.44	3.82	0.00251	S38	cl	n	-230.84	81.4	-0.16	0.04	0.16857
S38	clso4	c	88.66	25.2	0.2892	0.0209	35.50	6.61	0.00299	S38	clso4	c	314.53	64.8	0.11	0.03	0.11141
S38	scn	c	-112.04	14.0	0.5328	0.0111	19.08	1.62	0.00161	S38	scn	c	160.25	131.2	0.31	0.07	0.49204
S38	scncl	c	-81.64	23.7	0.5282	0.0161	11.08	1.25	0.00513	S38	scncl	c	234.20	137.2	0.29	0.06	0.50120
S38	scncl	h	-138.50	9.4	-0.2152	0.0062	9.62	1.07	0.00085	S38	scncl	h	-261.89	56.2	-0.12	0.02	0.08393
S38	scncl	n	80.63	22.3	-1.0624	0.0148	9.06	0.49	0.00493	S38	scncl	n	-519.10	278.3	-0.62	0.12	2.06155
S38	scn	h	-108.24	3.9	-0.2286	0.0031	15.66	0.94	0.00014	S38	scn	h	-219.20	57.6	-0.14	0.03	0.09478
S38	scn	n	80.64	7.9	-1.1822	0.0061	13.33	0.33	0.00059	S38	scn	n	-469.70	303.1	-0.74	0.15	2.62339
S38	scnso4	c	-152.33	25.4	0.6113	0.0193	22.93	2.12	0.00404	S38	scnso4	c	333.85	149.5	0.24	0.06	0.61731
S38	scnso4	h	-104.89	292.6	-0.3277	0.5479	324.78	730.17	0.01237	S38	scnso4	h	-392.34	49.7	-0.01	0.03	0.06009
S38	scnso4	n	548.12	1295.1	-2.0106	2.4994	350.27	562.21	0.21509	S38	scnso4	n	-1142.86	270.0	-0.06	0.14	1.77713
S50	cl	c	-14.25	7.8	0.0140	0.0067	40.11	48.76	0.00026	S50	cl	c	-3.67	4.7	0.01	0.00	0.00055
S50	cl	h	-21.03	6.3	0.0063	0.0051	31.26	70.25	0.00020	S50	cl	h	-16.20	3.3	0.00	0.00	0.00028
S50	cl	n	-39.71	6.4	0.0517	0.0066	87.45	21.60	0.00009	S50	cl	n	2.60	9.7	0.01	0.00	0.00241
S50	clso4	c	-76.43	118.1	0.1086	0.3821	998.89	2780.32	0.00034	S50	clso4	c	-16.33	5.3	0.00	0.00	0.00075
S50	clso4	h	-20.17	38.3	0.0225	0.1239	999.37	4361.29	0.00004	S50	clso4	h	-8.48	1.3	0.00	0.00	0.00005
S50	clso4	n	-42.40	11.0	0.0540	0.0093	40.14	17.22	0.00052	S50	clso4	n	1.30	12.9	0.02	0.01	0.00444
S50	scn	c	-57.91	4.9	0.0256	0.0042	27.81	15.27	0.00017	S50	scn	c	-43.41	6.5	0.01	0.00	0.00122
S50	scncl	c	-80.87	6.2	0.0400	0.0038	4.60	2.22	0.00047	S50	scncl	c	-62.48	11.6	0.03	0.01	0.00360
S50	scncl	h	11.55	68.1	-0.0668	0.2023	875.36	2188.01	0.00016	S50	scncl	h	-25.30	3.3	-0.00	0.00	0.00029
S50	scncl	n	-62.67	9.7	0.0046	0.0067	11.74	61.18	0.00084	S50	scncl	n	-59.38	5.8	0.00	0.00	0.00091
S50	scn	h	-13.74	34.9	-0.0076	0.1151	1000.00	12229.03	0.00005	S50	scn	h	-17.08	1.2	-0.00	0.00	0.00004
S50	scn	n	-56.12	5.2	-0.0066	0.0035	1.00	19.78	0.00035	S50	scn	n	-58.06	4.0	-0.01	0.00	0.00045
S50	scnso4	c	37.77	424.9	-0.0777	1.3763	1000.00	14022.71	0.00433	S50	scnso4	c	-16.12	11.3	0.01	0.00	0.00350
S50	scnso4	h	-65.03	43.9	0.0915	0.1421	999.82	1228.98	0.00005	S50	scnso4	h	-17.25	2.7	0.00	0.00	0.00021
S50	scnso4	n	-100.57	6.8	0.0273	0.0046	11.10	6.52	0.00043	S50	scnso4	n	-81.37	8.6	0.01	0.00	0.00202
S50	so4	c	-2.17	7.5	-0.0240	0.0051	1.00	7.68	0.00065	S50	so4	c	-9.86	8.6	-0.02	0.00	0.00204
S50	so4	h	5.59	16.5	-0.0192	0.0299	364.25	635.63	0.00008	S50	so4	h	-8.86	2.5	-0.00	0.00	0.00017
S50	so4	n	-41.69	7.3	0.0208	0.0049	1.00	8.62	0.00061	S50	so4	n	-34.98	7.8	0.02	0.00	0.00168
S57	clso4	c	-216.48	19.7	0.0541	0.0116	2.62	3.72	0.00513	S57	clso4	c	-195.65	20.3	0.04	0.01	0.01094
S57	clso4	h	-71.04	2.7	0.0313	0.0016	2.15	0.82	0.00010	S57	clso4	h	-59.65	8.9	0.02	0.00	0.00209
S57	clso4	n	24.49	12.0	0.0909	0.0070	2.02	1.20	0.00199	S57	clso4	n	57.03	26.7	0.07	0.01	0.01888

S57	scn	c	-172.35	17.5	-0.0999	0.0150	29.92	14.75	0.00202	S57	scn	c	-229.58	24.6	-0.05	0.01	0.01724
S57	scncl	c	-241.37	29.7	-0.1074	0.0327	107.28	58.20	0.00155	S57	scncl	c	-338.01	20.7	-0.02	0.01	0.01141
S57	scncl	h	-104.80	8.3	-0.0807	0.0098	132.58	26.65	0.00009	S57	scncl	h	-175.79	12.6	-0.01	0.01	0.00423
S57	scncl	n	-49.76	15.7	-0.0953	0.0179	118.81	38.37	0.00039	S57	scncl	n	-134.57	16.4	-0.02	0.01	0.00713
S57	scnso4	c	-292.79	21.7	0.0341	0.0116	1.00	2.71	0.00779	S57	scnso4	c	-281.86	18.9	0.03	0.01	0.00990
S57	scnso4	h	-205.17	5.4	0.0174	0.0029	1.00	1.32	0.00048	S57	scnso4	h	-199.50	6.2	0.01	0.00	0.00106
S57	scnso4	n	-213.95	17.1	0.0459	0.0093	1.46	1.95	0.00464	S57	scnso4	n	-196.74	18.0	0.03	0.01	0.00898
S67	clso4	c	28.87	7.3	0.0424	0.0050	12.59	5.22	0.00045	S67	clso4	c	54.86	11.2	0.02	0.00	0.00333
S67	clso4	h	-23.08	9.0	0.0084	0.0093	85.21	181.91	0.00018	S67	clso4	h	-14.65	3.3	0.00	0.00	0.00029
S67	clso4	n	221.20	22.4	-0.0508	0.0137	4.57	6.19	0.00599	S67	clso4	n	197.75	20.4	-0.03	0.01	0.01103
S67	scn	c	-443.73	59.6	0.1303	0.0498	25.58	34.42	0.02556	S67	scn	c	-370.69	43.8	0.07	0.02	0.05471
S67	scncl	c	-372.45	688.8	-0.0764	2.2307	1000.00	23094.28	0.01152	S67	scncl	c	-438.58	16.1	0.02	0.01	0.00687
S67	scncl	h	-104.19	11.1	-0.0909	0.0146	177.59	42.25	0.00011	S67	scncl	h	-181.88	11.8	-0.01	0.01	0.00371
S67	scncl	n	324.94	35.9	-0.2234	0.0412	121.70	38.23	0.00196	S67	scncl	n	133.78	33.6	-0.05	0.01	0.03004
S67	scnso4	c	-361.34	257.9	-0.1770	0.7610	999.79	3114.61	0.00035	S67	scnso4	c	-471.41	7.7	0.00	0.00	0.00081
S67	scnso4	h	-175.96	59.3	0.0797	0.1920	998.04	1902.82	0.00008	S67	scnso4	h	-133.95	2.9	0.00	0.00	0.00023
S67	scnso4	n	250.32	17.1	-0.0932	0.0125	18.29	7.63	0.00211	S67	scnso4	n	181.03	23.7	-0.04	0.01	0.01547
S67	so4	c	26.69	46.6	0.1223	0.0457	61.70	52.93	0.00667	S67	so4	c	115.78	29.9	0.04	0.02	0.02488
S80	cl	c	9.94	14.0	0.0393	0.0190	190.53	133.40	0.00016	S80	cl	c	41.97	5.6	0.01	0.00	0.00080
S80	cl	h	-35.82	8.8	0.0255	0.0086	72.21	50.31	0.00020	S80	cl	h	-14.67	6.1	0.01	0.00	0.00095
S80	cl	n	-128.34	6.3	0.0496	0.0065	83.33	21.31	0.00009	S80	cl	n	-87.70	9.6	0.01	0.00	0.00236
S80	clso4	c	23.88	214.0	0.1066	0.6929	1000.00	5141.09	0.00110	S80	clso4	c	87.37	7.1	-0.00	0.00	0.00134
S80	clso4	h	-35.68	2.4	0.0272	0.0016	12.96	2.73	0.00005	S80	clso4	h	-18.55	7.1	0.01	0.00	0.00134
S80	clso4	n	-120.51	10.2	0.0370	0.0063	4.26	3.73	0.00128	S80	clso4	n	-104.09	12.0	0.02	0.01	0.00380
S80	scn	c	-42.14	34.6	0.1739	0.0945	735.85	355.11	0.00010	S80	scn	c	54.88	8.3	0.01	0.00	0.00196
S80	scncl	c	29.98	8.6	0.0339	0.0071	34.29	18.70	0.00035	S80	scncl	c	56.30	8.5	0.01	0.00	0.00192
S80	scncl	h	-95.78	3.6	0.0292	0.0024	8.54	2.76	0.00013	S80	scncl	h	-79.40	8.1	0.02	0.00	0.00176
S80	scncl	n	-566.51	7.9	0.0458	0.0048	3.90	2.20	0.00078	S80	scncl	n	-546.48	13.6	0.03	0.01	0.00495
S80	scn	h	-75.55	1.7	0.0312	0.0014	23.52	3.96	0.00002	S80	scn	h	-58.67	7.6	0.02	0.00	0.00164
S80	scn	n	-468.75	7.9	0.0382	0.0055	3.94	6.38	0.00075	S80	scn	n	-455.14	12.0	0.03	0.01	0.00409
S80	scnso4	c	0.67	34.6	0.1375	0.0616	351.32	180.73	0.00035	S80	scnso4	c	109.67	11.7	0.01	0.00	0.00379
S80	scnso4	h	-105.95	4.8	0.0393	0.0034	15.18	4.27	0.00018	S80	scnso4	h	-77.15	10.4	0.02	0.00	0.00297
S80	scnso4	n	-659.38	5.7	0.0634	0.0036	6.44	1.45	0.00038	S80	scnso4	n	-622.18	17.0	0.04	0.01	0.00800
S80	so4	c	47.81	15.8	-0.0121	0.0108	1.00	32.24	0.00292	S80	so4	c	44.05	10.8	-0.01	0.01	0.00325
S80	so4	h	-7.04	2.2	0.0167	0.0016	7.84	4.94	0.00005	S80	so4	h	0.21	4.7	0.01	0.00	0.00061
S80	so4	n	26.71	4.7	0.0265	0.0032	1.00	4.35	0.00025	S80	so4	n	35.30	8.5	0.02	0.00	0.00200
S85	cl	c	45.05	7.0	0.0431	0.0064	53.34	18.17	0.00017	S85	cl	c	78.58	9.7	0.02	0.00	0.00239
S85	cl	h	-90.44	13.5	-0.0610	0.0175	168.45	73.27	0.00018	S85	cl	h	-140.56	8.7	-0.01	0.00	0.00193
S85	cl	n	-134.51	13.2	-0.3098	0.0162	146.47	12.22	0.00021	S85	cl	n	-392.97	47.0	-0.06	0.02	0.05632
S85	clso4	c	59.47	6.4	0.0654	0.0046	16.65	3.81	0.00031	S85	clso4	c	102.87	16.3	0.03	0.01	0.00706
S85	clso4	h	-120.47	4.9	-0.0784	0.0035	15.38	2.26	0.00019	S85	clso4	h	-171.75	19.7	-0.04	0.01	0.01031
S85	clso4	n	-226.40	23.1	-0.3560	0.0169	18.15	2.71	0.00382	S85	clso4	n	-469.25	88.2	-0.17	0.04	0.20658
S85	scn	c	130.14	4.7	0.0635	0.0039	24.00	5.29	0.00016	S85	scn	c	164.61	15.4	0.03	0.01	0.00677
S85	scncl	c	159.04	5.6	0.0900	0.0037	8.44	1.39	0.00032	S85	scncl	c	209.07	24.2	0.05	0.01	0.01557
S85	scncl	h	-242.98	8.4	-0.0904	0.0060	15.90	3.51	0.00054	S85	scncl	h	-302.09	22.8	-0.05	0.01	0.01380
S85	scncl	n	-628.75	30.1	-0.4134	0.0215	15.56	2.70	0.00705	S85	scncl	n	-898.09	104.0	-0.21	0.05	0.28820
S85	scn	h	-183.37	4.5	-0.0700	0.0037	23.35	4.54	0.00015	S85	scn	h	-220.99	16.9	-0.04	0.01	0.00811
S85	scn	n	-549.83	14.0	-0.3512	0.0119	28.56	3.24	0.00132	S85	scn	n	-748.69	82.0	-0.18	0.04	0.19198
S85	scnso4	c	154.31	11.8	0.0895	0.0082	13.35	4.10	0.00118	S85	scnso4	c	217.32	23.4	0.04	0.01	0.01517
S85	scnso4	h	-237.89	2.8	-0.0852	0.0020	13.90	1.07	0.00007	S85	scnso4	h	-299.32	22.1	-0.04	0.01	0.01353
S85	scnso4	n	-753.97	7.6	-0.3874	0.0053	13.95	0.64	0.00048	S85	scnso4	n	-1033.88	100.7	-0.18	0.04	0.28008
S85	so4	c	-44.69	39.7	0.1389	0.0689	333.54	191.48	0.00051	S85	so4	c	55.02	12.1	0.02	0.01	0.00407
S85	so4	h	-34.69	4.3	-0.0748	0.0040	49.85	6.76	0.00007	S85	so4	h	-85.42	15.2	-0.03	0.01	0.00648
S85	so4	n	-107.05	18.5	-0.2798	0.0165	38.36	6.34	0.00156	S85	so4	n	-286.48	60.4	-0.13	0.03	0.10197
S91	cl	c	-33.57	19.3	0.0096	0.0217	113.88	449.38	0.00060	S91	cl	c	-26.28	5.0	0.00	0.00	0.00063
S91	cl	h	-28.64	5.3	0.0050	0.0040	18.96	49.96	0.00018	S91	cl	h	-25.32	3.1	0.00	0.00	0.00024
S91	cl	n	-116.78	78.6	0.0461	0.2542	998.83	4355.89	0.00015	S91	cl	n	-92.85	2.9	0.00	0.00	0.00021

S91	clso4	c	-15.06	4.7	0.0305	0.0030	8.23	3.30	0.00022	S91	clso4	c	1.68	8.4	0.02	0.00	0.00188
S91	clso4	h	-24.68	2.8	0.0046	0.0020	15.55	22.29	0.00006	S91	clso4	h	-21.53	2.0	0.00	0.00	0.00010
S91	clso4	n	-313.50	286.7	0.2093	0.9284	1000.00	3508.99	0.00198	S91	clso4	n	-194.96	11.3	-0.00	0.00	0.00342
S91	scn	c	-106.02	81.7	0.2033	0.2698	1000.00	1065.06	0.00025	S91	scn	c	-3.87	8.3	0.01	0.00	0.00197
S91	scncl	c	-53.54	6.1	0.0300	0.0038	5.86	3.40	0.00042	S91	scncl	c	-38.65	9.0	0.02	0.00	0.00215
S91	scncl	h	-40.84	24.2	-0.0423	0.0487	448.78	539.89	0.00010	S91	scncl	h	-71.19	3.4	-0.00	0.00	0.00031
S91	scncl	n	-334.30	18.3	-0.0577	0.0167	55.00	35.64	0.00112	S91	scncl	n	-379.83	11.7	-0.02	0.01	0.00363
S91	scn	h	-34.56	33.3	-0.0362	0.1098	1000.00	2433.42	0.00004	S91	scn	h	-52.54	1.8	-0.00	0.00	0.00009
S91	scn	n	-276.92	4.4	-0.0493	0.0043	56.35	12.37	0.00008	S91	scn	n	-309.13	10.1	-0.02	0.01	0.00291
S91	scnso4	c	-22.72	8.7	0.0404	0.0056	8.11	4.28	0.00081	S91	scnso4	c	2.87	12.1	0.02	0.01	0.00406
S91	scnso4	h	-79.34	3.3	0.0074	0.0024	18.01	18.33	0.00008	S91	scnso4	h	-73.80	2.5	0.00	0.00	0.00017
S91	scnso4	n	-495.17	6.7	-0.0032	0.0036	1.00	8.94	0.00076	S91	scnso4	n	-496.13	5.3	-0.00	0.00	0.00077
S91	so4	c	3.15	10.6	0.0500	0.0117	100.30	44.19	0.00021	S91	so4	c	41.16	8.9	0.01	0.00	0.00221
S91	so4	h	-12.77	2.1	0.0051	0.0014	1.00	10.17	0.00005	S91	so4	h	-11.11	2.1	0.00	0.00	0.00012
S91	so4	n	-94.14	6.0	0.0233	0.0046	15.25	13.25	0.00027	S91	so4	n	-82.33	6.5	0.01	0.00	0.00119
S92	cl	c	-44.56	18.5	0.0164	0.0279	244.92	546.98	0.00019	S92	cl	c	-30.83	3.5	0.00	0.00	0.00031
S92	cl	h	-49.73	6.3	0.0055	0.0053	36.73	92.82	0.00018	S92	cl	h	-45.39	3.1	0.00	0.00	0.00024
S92	cl	n	-128.44	10.5	0.0520	0.0133	161.53	63.85	0.00011	S92	cl	n	-85.92	7.3	0.01	0.00	0.00137
S92	clso4	c	-206.48	75.8	0.1308	0.2453	999.27	1482.35	0.00014	S92	clso4	c	-137.14	4.9	0.00	0.00	0.00063
S92	clso4	h	-91.65	48.8	0.0245	0.1578	997.99	5088.85	0.00006	S92	clso4	h	-79.40	1.5	0.00	0.00	0.00006
S92	clso4	n	-242.62	8.7	0.0406	0.0066	22.64	10.81	0.00047	S92	clso4	n	-213.48	10.6	0.02	0.00	0.00297
S92	scn	c	-102.60	3.4	-0.0100	0.0023	1.00	8.60	0.00015	S92	scn	c	-105.63	3.7	-0.01	0.00	0.00040
S92	scncl	c	-160.93	6.8	0.0094	0.0041	4.57	10.17	0.00055	S92	scncl	c	-156.55	5.3	0.01	0.00	0.00074
S92	scncl	h	-64.99	22.6	-0.0361	0.0427	397.69	513.48	0.00012	S92	scncl	h	-91.77	3.3	-0.00	0.00	0.00030
S92	scncl	n	-248.62	174.1	0.0519	0.5638	1000.00	8591.57	0.00074	S92	scncl	n	-216.99	5.3	-0.00	0.00	0.00076
S92	scn	h	-40.02	19.5	-0.0282	0.0644	1000.00	1835.27	0.00001	S92	scn	h	-54.06	1.3	-0.00	0.00	0.00004
S92	scn	n	-113.22	3.4	-0.0026	0.0023	1.00	33.81	0.00015	S92	scn	n	-113.96	2.4	-0.00	0.00	0.00017
S92	scnso4	c	-146.50	77.4	-0.0730	0.1698	525.00	1204.75	0.00074	S92	scnso4	c	-199.73	7.0	-0.00	0.00	0.00135
S92	scnso4	h	-91.50	4.6	-0.0042	0.0048	93.41	201.69	0.00004	S92	scnso4	h	-95.36	1.4	-0.00	0.00	0.00006
S92	scnso4	n	-243.94	3.8	0.0229	0.0023	4.83	2.10	0.00018	S92	scnso4	n	-231.72	6.4	0.01	0.00	0.00114
S92	so4	c	-124.26	108.9	0.0554	0.3504	1000.00	4965.30	0.00030	S92	so4	c	-93.76	3.9	0.00	0.00	0.00043
S92	so4	h	-39.98	40.7	-0.0129	0.1065	720.35	5194.80	0.00010	S92	so4	h	-48.83	2.0	0.00	0.00	0.00011
S92	so4	n	-158.24	9.0	0.0476	0.0081	41.87	19.32	0.00034	S92	so4	n	-127.11	10.6	0.02	0.01	0.00315
T100	cl	c	-13.73	10.0	0.0553	0.0095	61.96	23.11	0.00030	T100	cl	c	29.55	11.6	0.02	0.01	0.00341
T100	cl	h	-4.79	84.9	-0.0037	0.2748	1000.00	58940.21	0.00017	T100	cl	h	-6.68	2.6	-0.00	0.00	0.00017
T100	cl	n	21.09	15.4	-0.0661	0.0216	206.23	94.78	0.00017	T100	cl	n	-34.40	9.5	-0.01	0.00	0.00228
T100	clso4	c	-73.45	12.4	0.1050	0.0105	39.25	9.76	0.00067	T100	clso4	c	9.77	23.2	0.04	0.01	0.01428
T100	clso4	h	11.63	1.0	-0.0065	0.0006	4.37	2.14	0.00001	T100	clso4	h	8.73	1.8	-0.00	0.00	0.00009
T100	clso4	n	103.98	8.7	-0.1000	0.0059	11.70	2.50	0.00067	T100	clso4	n	43.47	25.6	-0.05	0.01	0.01744
T100	scn	c	-51.75	7.9	0.1203	0.0098	131.21	18.88	0.00011	T100	scn	c	36.65	19.5	0.03	0.01	0.01088
T100	scncl	c	-42.53	16.0	0.0993	0.0141	47.06	15.76	0.00097	T100	scncl	c	39.62	22.6	0.03	0.01	0.01361
T100	scncl	h	-67.95	6.1	-0.0156	0.0053	42.31	34.88	0.00015	T100	scncl	h	-79.92	3.8	-0.01	0.00	0.00038
T100	scncl	n	-20.06	13.7	-0.1141	0.0112	33.65	8.70	0.00091	T100	scncl	n	-107.16	25.6	-0.04	0.01	0.01741
T100	scn	h	-75.57	3.0	-0.0121	0.0025	24.92	18.13	0.00006	T100	scn	h	-82.08	3.1	-0.01	0.00	0.00028
T100	scn	n	-69.35	11.8	-0.0942	0.0105	34.92	11.88	0.00084	T100	scn	n	-124.86	21.5	-0.05	0.01	0.01320
T100	scnso4	c	-21.97	12.6	0.1058	0.0093	19.74	5.29	0.00109	T100	scnso4	c	58.57	26.1	0.04	0.01	0.01876
T100	scnso4	h	-97.36	2.5	-0.0058	0.0013	1.00	1.79	0.00010	T100	scnso4	h	-99.24	2.4	-0.00	0.00	0.00016
T100	scnso4	n	-13.39	6.8	-0.0971	0.0046	12.22	2.00	0.00041	T100	scnso4	n	-80.83	25.2	-0.05	0.01	0.01761
T100	so4	c	-35.49	13.9	0.1136	0.0145	78.32	20.75	0.00047	T100	so4	c	47.64	20.8	0.04	0.01	0.01203
T100	so4	h	15.14	4.6	-0.0082	0.0044	54.26	69.92	0.00007	T100	so4	h	9.39	2.3	-0.00	0.00	0.00015
T100	so4	n	57.25	5.2	-0.0796	0.0043	23.78	4.47	0.00017	T100	so4	n	11.47	19.0	-0.04	0.01	0.01007
T105	cl	c	28.20	109.3	-0.0772	0.3534	998.16	3617.68	0.00029	T105	cl	c	-11.37	4.1	-0.00	0.00	0.00044
T105	cl	h	-23.29	5.5	0.0069	0.0043	25.61	47.70	0.00017	T105	cl	h	-18.42	3.2	0.00	0.00	0.00026
T105	cl	n	-202.36	110.6	0.4928	0.3581	1000.00	574.65	0.00030	T105	cl	n	55.65	17.4	0.01	0.01	0.00769
T105	clso4	c	-7.66	4.9	-0.0320	0.0033	11.56	4.33	0.00021	T105	clso4	c	-27.22	8.8	-0.02	0.00	0.00205
T105	clso4	h	-22.81	2.7	0.0113	0.0019	14.77	8.27	0.00006	T105	clso4	h	-15.53	3.1	0.01	0.00	0.00026
T105	clso4	n	-466.71	422.5	0.9306	1.3680	1000.00	1162.98	0.00430	T105	clso4	n	34.29	34.1	0.01	0.01	0.03096

T105	scn	c	-152.02	4.8	-0.0259	0.0046	49.14	22.89	0.00011	T105	scn	c	-168.36	5.6	-0.01	0.00	0.00090
T105	scncl	c	-105.05	55.9	-0.1090	0.1112	438.11	470.88	0.00058	T105	scncl	c	-185.99	9.4	-0.01	0.00	0.00238
T105	scncl	h	-21.33	72.7	-0.0175	0.2354	1000.00	10669.28	0.00013	T105	scncl	h	-31.39	2.3	0.00	0.00	0.00014
T105	scncl	n	-322.86	335.1	0.3833	1.0851	1000.00	2240.03	0.00272	T105	scncl	n	-109.68	17.0	0.00	0.01	0.00769
T105	scn	h	-22.71	2.3	0.0114	0.0021	39.22	21.16	0.00003	T105	scn	h	-15.64	2.8	0.01	0.00	0.00023
T105	scn	n	-82.35	280.9	0.0359	0.9273	1000.00	20716.71	0.00296	T105	scn	n	-53.84	9.0	-0.01	0.00	0.00233
T105	scnso4	c	-174.17	24.5	-0.0531	0.0194	68.03	46.25	0.00065	T105	scnso4	c	-234.25	13.0	-0.01	0.00	0.00233
T105	scnso4	h	-31.10	2.8	0.0157	0.0021	20.28	8.20	0.00005	T105	scnso4	h	-18.82	4.2	0.01	0.00	0.00048
T105	scnso4	n	-140.38	501.3	0.1082	1.6236	1000.00	11877.58	0.00603	T105	scnso4	n	-65.13	12.6	-0.01	0.01	0.00437
T105	so4	c	-27.28	2.3	-0.0334	0.0017	12.28	3.12	0.00004	T105	so4	c	-43.42	8.7	-0.02	0.00	0.00209
T105	so4	h	-2.35	1.8	0.0103	0.0012	1.00	4.28	0.00004	T105	so4	h	0.99	3.3	0.01	0.00	0.00030
T105	so4	n	-192.27	227.7	0.5260	0.7329	1000.00	1094.50	0.00131	T105	so4	n	80.82	19.4	0.01	0.01	0.01055
T107	cl	c	4.16	9.1	0.0144	0.0084	56.89	74.64	0.00026	T107	cl	c	14.71	4.0	0.01	0.00	0.00041
T107	cl	h	-26.38	5.3	0.0028	0.0041	23.97	109.69	0.00017	T107	cl	h	-24.29	2.7	0.00	0.00	0.00019
T107	cl	n	39.28	16.3	-0.1714	0.0279	322.92	62.11	0.00009	T107	cl	n	-93.22	16.8	-0.02	0.01	0.00722
T107	clso4	c	-7.88	7.8	0.0017	0.0081	88.38	830.82	0.00013	T107	clso4	c	-6.98	2.2	0.00	0.00	0.00013
T107	clso4	h	-8.67	2.1	-0.0039	0.0013	2.87	5.88	0.00006	T107	clso4	h	-10.21	1.8	-0.00	0.00	0.00009
T107	clso4	n	163.56	14.3	-0.1288	0.0110	24.64	6.06	0.00122	T107	clso4	n	69.74	31.0	-0.06	0.01	0.02547
T107	scn	c	-91.51	2.2	-0.0087	0.0016	8.40	9.93	0.00005	T107	scn	c	-95.13	2.7	-0.01	0.00	0.00021
T107	scncl	c	-116.33	16.9	-0.0032	0.0189	112.96	1158.38	0.00047	T107	scncl	c	-118.03	4.1	-0.00	0.00	0.00045
T107	scncl	h	-37.71	14.3	-0.0268	0.0205	216.93	228.30	0.00014	T107	scncl	h	-59.86	3.7	-0.00	0.00	0.00036
T107	scncl	n	102.29	29.7	-0.3073	0.0333	113.51	21.46	0.00145	T107	scncl	n	-165.23	49.5	-0.06	0.02	0.06518
T107	scn	h	-35.66	24.8	-0.0344	0.0818	997.63	1904.83	0.00002	T107	scn	h	-52.80	1.6	-0.00	0.00	0.00007
T107	scn	n	-44.84	15.6	-0.2537	0.0188	119.44	16.20	0.00050	T107	scn	n	-228.32	41.3	-0.07	0.02	0.04884
T107	scnso4	c	-121.07	117.9	-0.0112	0.3819	1000.00	27020.00	0.00033	T107	scnso4	c	-130.04	3.1	0.00	0.00	0.00027
T107	scnso4	h	-55.45	59.8	0.0096	0.1938	1000.00	15925.75	0.00009	T107	scnso4	h	-49.20	1.7	-0.00	0.00	0.00008
T107	scnso4	n	70.54	18.4	-0.2215	0.0172	60.84	10.22	0.00104	T107	scnso4	n	-128.60	44.0	-0.06	0.02	0.05344
T107	so4	c	8.73	219.0	-0.0549	0.7049	1000.00	10087.62	0.00121	T107	so4	c	-17.45	6.6	-0.00	0.00	0.00121
T107	so4	h	8.80	5.7	-0.0022	0.0060	81.36	454.78	0.00008	T107	so4	h	6.88	1.7	-0.00	0.00	0.00008
T107	so4	n	135.43	8.8	-0.0819	0.0072	23.74	7.31	0.00048	T107	so4	n	88.13	20.0	-0.04	0.01	0.01118
T16	cl	c	-8.05	74.6	-0.0147	0.2415	1000.00	13009.45	0.00013	T16	cl	c	-16.39	2.4	0.00	0.00	0.00014
T16	cl	h	-3.93	6.3	0.0039	0.0054	38.96	137.43	0.00017	T16	cl	h	-0.75	2.8	0.00	0.00	0.00020
T16	cl	n	161.22	4.5	-0.0063	0.0032	12.88	25.28	0.00016	T16	cl	n	157.58	3.1	-0.00	0.00	0.00024
T16	clso4	c	-14.73	10.0	-0.0208	0.0081	31.65	32.78	0.00050	T16	clso4	c	-30.76	6.5	-0.01	0.00	0.00112
T16	clso4	h	5.63	41.7	0.0248	0.1349	997.43	4303.47	0.00004	T16	clso4	h	18.99	1.5	0.00	0.00	0.00006
T16	clso4	n	155.41	274.6	0.2720	0.8877	997.77	2577.93	0.00183	T16	clso4	n	305.97	12.8	0.00	0.01	0.00438
T16	scn	c	-70.62	5.0	-0.0198	0.0049	56.43	35.26	0.00011	T16	scn	c	-83.44	4.4	-0.01	0.00	0.00054
T16	scncl	c	-64.01	15.0	-0.0395	0.0247	297.76	227.12	0.00009	T16	scncl	c	-97.08	4.7	-0.00	0.00	0.00058
T16	scncl	h	-5.31	14.5	-0.0138	0.0187	169.81	346.68	0.00021	T16	scncl	h	-16.24	3.0	-0.00	0.00	0.00024
T16	scncl	n	388.69	597.1	0.1274	1.9337	1000.00	12008.13	0.00865	T16	scncl	n	477.40	15.6	-0.01	0.01	0.00644
T16	scn	h	-17.95	10.2	0.0040	0.0171	287.54	1585.33	0.00006	T16	scn	h	-14.43	1.6	0.00	0.00	0.00007
T16	scn	n	296.73	359.7	0.1166	1.1873	1000.00	8171.45	0.00485	T16	scn	n	367.71	13.4	-0.01	0.01	0.00510
T16	scnso4	c	-19.11	95.8	-0.1579	0.3104	1000.00	1555.71	0.00022	T16	scnso4	c	-104.03	5.6	-0.00	0.00	0.00088
T16	scnso4	h	-58.92	45.9	0.1122	0.1485	1000.00	1048.01	0.00005	T16	scnso4	h	-0.02	3.4	0.00	0.00	0.00031
T16	scnso4	n	239.47	345.2	0.6118	1.1181	1000.00	1446.38	0.00286	T16	scnso4	n	574.95	22.2	0.00	0.01	0.01365
T16	so4	c	-15.99	4.3	-0.0097	0.0030	3.57	13.13	0.00020	T16	so4	c	-19.58	3.9	-0.01	0.00	0.00041
T16	so4	h	19.54	1.7	0.0045	0.0012	1.67	9.72	0.00003	T16	so4	h	21.05	1.7	0.00	0.00	0.00008
T16	so4	n	61.46	60.7	0.2719	0.1683	792.77	414.91	0.00017	T16	so4	n	215.26	11.3	0.01	0.01	0.00358
T26	cl	c	-17.13	3.8	0.0067	0.0024	2.79	9.00	0.00017	T26	cl	c	-14.62	3.3	0.00	0.00	0.00027
T26	cl	h	4.26	6.9	0.0190	0.0064	55.89	42.37	0.00015	T26	cl	h	19.55	5.0	0.01	0.00	0.00064
T26	cl	n	-25.51	6.5	-0.1276	0.0072	109.37	10.99	0.00007	T26	cl	n	-132.51	22.5	-0.03	0.01	0.01291
T26	clso4	c	-7.36	12.1	0.0073	0.0068	1.00	11.88	0.00213	T26	clso4	c	-5.20	9.2	0.01	0.00	0.00224
T26	clso4	h	-39.26	100.7	0.1665	0.3258	999.38	1547.27	0.00024	T26	clso4	h	51.62	6.7	0.00	0.00	0.00118
T26	clso4	n	-41.36	13.2	-0.2022	0.0101	22.91	3.36	0.00110	T26	clso4	n	-187.15	49.0	-0.09	0.02	0.06370
T26	scn	c	-56.97	12.1	0.0342	0.0205	291.93	221.08	0.00008	T26	scn	c	-32.06	4.2	0.00	0.00	0.00051
T26	scncl	c	-57.35	4.4	0.0177	0.0027	5.79	4.12	0.00022	T26	scncl	c	-48.60	5.5	0.01	0.00	0.00082
T26	scncl	h	31.17	7.4	-0.0294	0.0071	63.40	32.41	0.00016	T26	scncl	h	5.95	6.5	-0.01	0.00	0.00112

T26	scncl	n	-113.29	8.5	-0.1378	0.0054	7.29	1.22	0.00076	T26	scncl	n	-185.93	36.6	-0.08	0.02	0.03559
T26	scn	h	9.82	3.4	-0.0290	0.0034	61.36	17.57	0.00005	T26	scn	h	-9.40	5.9	-0.01	0.00	0.00098
T26	scn	n	-133.68	4.3	-0.0512	0.0030	5.51	2.84	0.00021	T26	scn	n	-153.02	14.4	-0.04	0.01	0.00592
T26	scnso4	c	-33.00	11.6	0.0104	0.0054	5.48	11.20	0.00078	T26	scnso4	c	-24.18	8.9	0.01	0.00	0.00109
T26	scnso4	h	29.50	3.9	-0.0117	0.0024	5.60	4.70	0.00018	T26	scnso4	h	22.81	4.1	-0.01	0.00	0.00046
T26	scnso4	n	-112.87	6.9	-0.1466	0.0047	11.46	1.28	0.00044	T26	scnso4	n	-214.74	39.4	-0.07	0.02	0.04278
T26	so4	c	-122.06	81.6	0.2036	0.2625	999.35	1012.32	0.00017	T26	so4	c	-18.95	6.7	0.01	0.00	0.00124
T26	so4	h	46.67	3.7	-0.0051	0.0025	1.00	17.89	0.00016	T26	so4	h	45.03	2.8	-0.00	0.00	0.00022
T26	so4	n	-30.79	15.8	-0.1384	0.0192	133.46	30.87	0.00034	T26	so4	n	-138.15	21.2	-0.03	0.01	0.01259
T6	cl	c	-12.17	106.8	-0.0057	0.3458	1000.00	47952.20	0.00028	T6	cl	c	-15.42	3.3	0.00	0.00	0.00028
T6	cl	h	17.66	5.9	0.0091	0.0049	35.61	51.04	0.00016	T6	cl	h	24.48	3.4	0.00	0.00	0.00030
T6	cl	n	306.46	74.5	0.0474	0.2413	1000.00	4029.38	0.00013	T6	cl	n	332.75	3.0	0.00	0.00	0.00022
T6	clso4	c	-90.60	109.1	0.1096	0.3533	999.53	2549.14	0.00029	T6	clso4	c	-33.60	4.6	0.00	0.00	0.00057
T6	clso4	h	36.85	2.1	0.0065	0.0013	7.74	6.52	0.00004	T6	clso4	h	40.50	2.2	0.00	0.00	0.00013
T6	clso4	n	477.53	7.3	-0.0188	0.0041	1.00	2.80	0.00078	T6	clso4	n	472.08	7.4	-0.01	0.00	0.00146
T6	scn	c	-57.12	144.5	0.0243	0.4769	1000.00	15720.35	0.00078	T6	scn	c	-39.80	4.8	-0.00	0.00	0.00067
T6	scncl	c	-82.29	11.6	0.0124	0.0123	95.15	175.79	0.00027	T6	scncl	c	-70.24	4.2	0.00	0.00	0.00046
T6	scncl	h	-1.10	4.6	0.0033	0.0036	26.78	82.45	0.00012	T6	scncl	h	1.77	2.4	0.00	0.00	0.00015
T6	scncl	n	236.73	276.3	0.2595	0.8938	998.52	2722.64	0.00186	T6	scncl	n	382.44	12.5	-0.00	0.01	0.00415
T6	scn	h	-19.03	4.5	0.0105	0.0055	123.42	115.94	0.00004	T6	scn	h	-10.96	2.3	0.00	0.00	0.00015
T6	scn	n	72.06	162.8	0.1940	0.5372	1000.00	2222.54	0.00099	T6	scn	n	173.29	10.1	0.00	0.01	0.00294
T6	scnso4	c	-127.28	183.1	0.1395	0.5929	999.24	3363.03	0.00081	T6	scnso4	c	-53.28	6.8	0.00	0.00	0.00128
T6	scnso4	h	-19.56	4.2	0.0149	0.0034	32.62	19.57	0.00009	T6	scnso4	h	-6.90	3.9	0.00	0.00	0.00042
T6	scnso4	n	100.24	326.2	0.3008	1.0564	1000.00	2779.42	0.00255	T6	scnso4	n	270.13	13.7	-0.00	0.01	0.00516
T6	so4	c	-16.75	10.2	-0.0117	0.0070	1.00	21.52	0.00122	T6	so4	c	-20.46	7.4	-0.01	0.00	0.00155
T6	so4	h	-6.00	1.8	0.0060	0.0012	4.94	9.32	0.00003	T6	so4	h	-3.64	2.0	0.00	0.00	0.00011
T6	so4	n	84.39	7.1	-0.0142	0.0049	1.00	12.37	0.00059	T6	so4	n	79.85	6.2	-0.01	0.00	0.00108
T70	cl	c	55.56	11.2	0.0451	0.0139	150.26	73.23	0.00014	T70	cl	c	93.42	7.3	0.01	0.00	0.00135
T70	cl	h	-20.07	97.2	-0.0261	0.3145	1000.00	9532.20	0.00023	T70	cl	h	-33.77	3.1	-0.00	0.00	0.00025
T70	cl	n	330.67	17.5	-0.2165	0.0269	257.27	41.33	0.00016	T70	cl	n	157.30	24.5	-0.03	0.01	0.01524
T70	clso4	c	141.67	6.1	0.0509	0.0043	15.95	4.44	0.00028	T70	clso4	c	175.21	13.0	0.03	0.01	0.00447
T70	clso4	h	-24.81	42.9	0.0158	0.1390	1000.00	6936.31	0.00004	T70	clso4	h	-15.03	1.3	-0.00	0.00	0.00004
T70	clso4	n	780.41	9.9	-0.0345	0.0069	14.02	9.55	0.00079	T70	clso4	n	758.65	10.0	-0.02	0.00	0.00266
T70	scn	c	34.28	8.9	0.0945	0.0100	97.25	20.59	0.00020	T70	scn	c	102.62	17.6	0.03	0.01	0.00881
T70	scncl	c	99.84	7.3	0.0598	0.0056	24.18	6.61	0.00032	T70	scncl	c	143.88	15.2	0.03	0.01	0.00619
T70	scncl	h	-104.86	5.9	-0.0229	0.0052	46.89	25.22	0.00013	T70	scncl	h	-123.22	5.1	-0.01	0.00	0.00070
T70	scncl	n	-38.65	15.7	-0.2749	0.0129	34.49	4.22	0.00117	T70	scncl	n	-252.45	62.5	-0.10	0.03	0.10401
T70	scn	h	-95.87	1.9	-0.0177	0.0016	22.18	7.32	0.00003	T70	scn	h	-105.28	4.4	-0.01	0.00	0.00055
T70	scn	n	-265.73	6.6	-0.3200	0.0063	49.35	2.56	0.00021	T70	scn	n	-471.38	68.1	-0.14	0.03	0.13243
T70	scnso4	c	147.45	7.5	0.0645	0.0054	16.97	4.49	0.00042	T70	scnso4	c	196.29	17.1	0.03	0.01	0.00808
T70	scnso4	h	-169.50	86.8	0.0685	0.2812	1000.00	3246.59	0.00018	T70	scnso4	h	-130.71	3.4	-0.00	0.00	0.00032
T70	scnso4	n	-246.00	22.4	-0.0627	0.0142	7.24	6.39	0.00558	T70	scnso4	n	-285.21	22.5	-0.03	0.01	0.01404
T70	so4	c	83.84	6.7	0.0692	0.0062	44.64	10.44	0.00018	T70	so4	c	129.95	14.8	0.03	0.01	0.00610
T70	so4	h	23.65	2.4	0.0036	0.0017	4.83	20.59	0.00006	T70	so4	h	25.07	1.8	0.00	0.00	0.00009
T70	so4	n	530.14	282.9	0.2960	0.9106	999.69	2416.11	0.00203	T70	so4	n	687.92	14.1	0.00	0.01	0.00556
T79	cl	c	-26.07	11.6	-0.0099	0.0120	85.41	201.15	0.00030	T79	cl	c	-33.77	3.8	-0.00	0.00	0.00036
T79	cl	h	-47.75	18.6	-0.0777	0.0286	257.10	122.31	0.00018	T79	cl	h	-109.13	8.7	-0.01	0.00	0.00191
T79	cl	n	100.87	43.6	-0.5744	0.1322	904.79	169.93	0.00006	T79	cl	n	-210.96	20.8	-0.02	0.01	0.01106
T79	clso4	c	-14.30	5.0	-0.0124	0.0033	9.38	9.57	0.00024	T79	clso4	c	-21.75	4.6	-0.01	0.00	0.00057
T79	clso4	h	-75.62	4.3	-0.0711	0.0035	34.27	4.42	0.00009	T79	clso4	h	-131.07	16.1	-0.03	0.01	0.00691
T79	clso4	n	88.92	52.2	-0.4793	0.1050	447.77	102.58	0.00048	T79	clso4	n	-262.63	34.8	-0.03	0.02	0.03222
T79	scn	c	-155.44	2.6	-0.0504	0.0019	4.84	1.71	0.00008	T79	scn	c	-174.03	14.2	-0.04	0.01	0.00572
T79	scncl	c	-191.52	3.2	-0.0460	0.0021	8.35	1.55	0.00011	T79	scncl	c	-216.75	12.1	-0.03	0.01	0.00392
T79	scncl	h	-169.49	9.1	-0.0949	0.0066	16.88	3.81	0.00062	T79	scncl	h	-232.42	23.7	-0.05	0.01	0.01499
T79	scncl	n	243.06	66.5	-0.6474	0.1027	260.10	53.04	0.00224	T79	scncl	n	-285.79	66.7	-0.07	0.03	0.11855
T79	scn	h	-137.38	5.1	-0.0799	0.0043	25.07	4.74	0.00019	T79	scn	h	-181.07	19.0	-0.04	0.01	0.01033
T79	scn	n	-25.87	42.2	-0.4007	0.0639	224.14	50.38	0.00155	T79	scn	n	-314.83	47.8	-0.08	0.02	0.06524

T79	scnso4	c	-182.76	7.5	-0.0522	0.0059	26.74	8.46	0.00032	T79	scnso4	c	-226.63	13.7	-0.02	0.01	0.00518
T79	scnso4	h	-177.07	2.0	-0.0916	0.0015	21.97	1.09	0.00003	T79	scnso4	h	-249.62	22.7	-0.04	0.01	0.01420
T79	scnso4	n	-44.61	36.8	-0.4247	0.0490	183.18	31.09	0.00119	T79	scnso4	n	-424.79	54.5	-0.05	0.02	0.08220
T79	so4	c	45.72	3.3	-0.0131	0.0022	1.00	6.17	0.00012	T79	so4	c	41.48	4.4	-0.01	0.00	0.00055
T79	so4	h	6.31	8.8	-0.0794	0.0119	184.49	40.62	0.00007	T79	so4	h	-55.71	10.6	-0.01	0.01	0.00313
T79	so4	n	-23.48	371.0	-0.2347	1.1943	1000.00	3997.99	0.00349	T79	so4	n	-154.84	14.4	0.00	0.01	0.00577
T99	cl	c	59.91	6.2	0.0068	0.0049	25.12	53.91	0.00022	T99	cl	c	64.68	3.5	0.00	0.00	0.00031
T99	cl	h	-27.34	6.4	0.0095	0.0055	40.90	59.15	0.00017	T99	cl	h	-19.91	3.5	0.00	0.00	0.00032
T99	cl	n	-115.07	6.3	0.0362	0.0063	76.05	26.66	0.00010	T99	cl	n	-85.57	7.4	0.01	0.00	0.00141
T99	clso4	c	134.17	293.2	-0.0834	0.9495	1000.00	9006.24	0.00207	T99	clso4	c	95.87	8.1	-0.01	0.00	0.00173
T99	clso4	h	-17.37	2.5	0.0109	0.0017	9.07	5.33	0.00006	T99	clso4	h	-11.15	3.2	0.01	0.00	0.00028
T99	clso4	n	-142.08	12.1	0.0185	0.0069	1.00	4.73	0.00214	T99	clso4	n	-136.70	10.3	0.01	0.00	0.00281
T99	scn	c	73.51	212.2	0.0115	0.7002	1000.00	49073.92	0.00169	T99	scn	c	87.40	6.5	-0.01	0.00	0.00119
T99	scncl	c	97.68	7.6	0.0233	0.0067	48.81	32.86	0.00021	T99	scncl	c	117.90	6.3	0.01	0.00	0.00105
T99	scncl	h	-63.79	3.9	0.0048	0.0024	5.86	13.57	0.00017	T99	scncl	h	-61.30	2.9	0.00	0.00	0.00022
T99	scncl	n	-248.73	6.5	0.0108	0.0037	1.88	5.28	0.00058	T99	scncl	n	-244.92	5.6	0.01	0.00	0.00083
T99	scn	h	-49.55	1.6	0.0091	0.0013	26.12	13.08	0.00002	T99	scn	h	-44.45	2.3	0.00	0.00	0.00016
T99	scn	n	-166.64	4.3	0.0364	0.0034	21.01	7.68	0.00014	T99	scn	n	-147.48	9.2	0.02	0.00	0.00241
T99	scnso4	c	10.09	318.4	0.1800	1.0313	1000.00	4535.61	0.00243	T99	scnso4	c	114.59	10.8	-0.00	0.00	0.00322
T99	scnso4	h	-58.61	3.6	0.0134	0.0024	11.02	6.94	0.00012	T99	scnso4	h	-49.38	4.1	0.01	0.00	0.00048
T99	scnso4	n	-228.83	9.1	0.0173	0.0051	1.82	3.18	0.00128	T99	scnso4	n	-221.82	8.3	0.01	0.00	0.00191
T99	so4	c	48.45	7.8	-0.0171	0.0053	1.00	11.33	0.00072	T99	so4	c	42.99	7.1	-0.01	0.00	0.00142
T99	so4	h	-8.16	2.6	0.0079	0.0018	1.00	8.23	0.00008	T99	so4	h	-5.61	2.9	0.01	0.00	0.00023
T99	so4	n	-105.81	12.6	0.0156	0.0086	1.00	19.85	0.00184	T99	so4	n	-100.87	9.3	0.01	0.00	0.00241
V10	cl	c	33.75	117.1	0.0053	0.3789	1000.00	56604.54	0.00033	V10	cl	c	38.01	3.5	-0.00	0.00	0.00032
V10	cl	h	10.93	9.7	0.0192	0.0105	102.86	102.72	0.00017	V10	cl	h	27.40	4.5	0.00	0.00	0.00052
V10	cl	n	30.92	26.3	0.1529	0.0494	391.35	139.02	0.00016	V10	cl	n	144.32	13.2	0.01	0.01	0.00445
V10	clso4	c	49.33	51.4	0.0379	0.1170	561.82	1668.28	0.00028	V10	clso4	c	75.30	4.0	0.00	0.00	0.00042
V10	clso4	h	27.27	5.1	0.0370	0.0047	58.53	16.40	0.00008	V10	clso4	h	58.34	7.5	0.01	0.00	0.00151
V10	clso4	n	182.49	17.0	0.1279	0.0141	35.22	10.01	0.00137	V10	clso4	n	281.43	28.6	0.05	0.01	0.02165
V10	scn	c	-33.78	103.5	0.1137	0.3411	997.68	2403.19	0.00040	V10	scn	c	25.57	6.2	0.00	0.00	0.00109
V10	scncl	c	7.49	10.8	0.0278	0.0074	12.22	11.67	0.00101	V10	scncl	c	24.38	9.3	0.02	0.00	0.00230
V10	scncl	h	-18.94	3.8	0.0168	0.0029	22.45	11.63	0.00009	V10	scncl	h	-6.65	4.7	0.01	0.00	0.00059
V10	scncl	n	20.74	15.3	0.1109	0.0161	91.75	25.01	0.00049	V10	scncl	n	116.11	19.2	0.03	0.01	0.00983
V10	scn	h	-26.37	2.0	0.0224	0.0020	68.14	14.14	0.00001	V10	scn	h	-11.02	4.5	0.01	0.00	0.00059
V10	scn	n	-12.70	9.6	0.1207	0.0136	187.30	31.99	0.00011	V10	scn	n	76.43	16.7	0.02	0.01	0.00797
V10	scnso4	c	-143.14	175.0	0.3604	0.5666	999.85	1244.27	0.00073	V10	scnso4	c	43.52	10.2	0.01	0.00	0.00286
V10	scnso4	h	-46.96	6.4	0.0499	0.0061	65.36	16.75	0.00012	V10	scnso4	h	-2.16	9.7	0.01	0.00	0.00257
V10	scnso4	n	14.33	15.5	0.1604	0.0137	48.27	9.59	0.00089	V10	scnso4	n	155.79	34.2	0.05	0.01	0.03224
V10	so4	c	16.45	172.0	0.0677	0.5537	1000.00	6423.18	0.00075	V10	so4	c	55.63	5.7	-0.00	0.00	0.00092
V10	so4	h	47.41	6.3	0.0306	0.0057	41.20	20.77	0.00017	V10	so4	h	67.22	6.8	0.01	0.00	0.00130
V10	so4	n	169.71	18.2	0.1479	0.0185	71.23	19.26	0.00089	V10	so4	n	275.57	27.2	0.05	0.01	0.02061
V36	cl	c	-15.93	13.6	-0.0275	0.0118	42.28	45.26	0.00075	V36	cl	c	-35.74	7.8	-0.01	0.00	0.00155
V36	cl	h	-42.57	18.1	-0.0967	0.0287	274.06	102.51	0.00015	V36	cl	h	-118.66	10.4	-0.01	0.00	0.00276
V36	cl	n	-157.26	16.5	-0.1651	0.0299	364.09	74.43	0.00007	V36	cl	n	-281.35	14.7	-0.01	0.01	0.00551
V36	clso4	c	179.19	15.9	-0.3523	0.0105	9.00	1.04	0.00248	V36	clso4	c	-21.04	93.1	-0.20	0.04	0.23014
V36	clso4	h	-42.46	4.6	-0.1351	0.0031	10.25	0.87	0.00020	V36	clso4	h	-121.88	35.1	-0.08	0.02	0.03264
V36	clso4	n	-318.29	7.9	-0.0535	0.0048	3.79	1.85	0.00078	V36	clso4	n	-341.45	15.5	-0.04	0.01	0.00636
V36	scn	c	-22.00	26.3	-0.1117	0.0197	11.49	10.84	0.00681	V36	scn	c	-72.21	33.0	-0.07	0.02	0.03118
V36	scncl	c	-35.04	9.2	-0.1484	0.0062	11.18	1.73	0.00077	V36	scncl	c	-123.95	38.6	-0.08	0.02	0.03976
V36	scncl	h	-73.56	17.7	-0.1129	0.0298	313.55	99.05	0.00011	V36	scncl	h	-164.26	11.0	-0.01	0.00	0.00325
V36	scncl	n	-349.42	48.7	-0.3331	0.0815	310.21	91.24	0.00088	V36	scncl	n	-620.27	33.8	-0.03	0.01	0.03032
V36	scn	h	-122.87	9.5	0.0115	0.0064	1.00	20.86	0.00116	V36	scn	h	-119.48	7.2	0.01	0.00	0.00147
V36	scn	n	-132.20	274.1	-0.7051	0.9044	999.78	1029.31	0.00282	V36	scn	n	-487.54	29.1	-0.02	0.01	0.02422
V36	scnso4	c	114.35	14.2	-0.2807	0.0091	7.51	0.93	0.00221	V36	scnso4	c	-59.84	76.1	-0.15	0.03	0.15987
V36	scnso4	h	-114.16	6.2	-0.0344	0.0035	2.14	1.21	0.00058	V36	scnso4	h	-128.68	10.3	-0.02	0.00	0.00296
V36	scnso4	n	-682.17	73.7	-0.0124	0.2386	1000.00	15274.71	0.00013	V36	scnso4	n	-689.66	2.2	0.00	0.00	0.00013

V36	so4	h	-21.40	17.2	-0.2363	0.0162	51.11	8.71	0.00107	V36	so4	h	-182.33	47.9	-0.10	0.02	0.06417
V36	so4	n	-48.18	7.0	-0.0435	0.0049	4.79	5.05	0.00050	V36	so4	n	-65.19	12.9	-0.03	0.01	0.00464
V3	cl	c	-106.90	38.1	0.0478	0.0346	52.15	87.45	0.00500	V3	cl	c	-67.89	18.3	0.02	0.01	0.00855
V3	clso4	c	-182.36	172.5	0.2108	0.5585	1000.00	2095.77	0.00072	V3	clso4	c	-67.44	9.0	0.00	0.00	0.00216
V3	clso4	h	-45.96	16.7	0.0328	0.0263	269.55	274.05	0.00013	V3	clso4	h	-18.07	4.4	0.00	0.00	0.00051
V3	clso4	n	179.32	561.6	0.8141	1.8184	1000.00	1767.08	0.00759	V3	clso4	n	620.96	32.9	0.01	0.01	0.02876
V3	scn	c	-187.70	400.9	0.1427	1.3232	1000.00	7440.56	0.00603	V3	scn	c	-103.72	15.4	-0.00	0.01	0.00679
V3	scncl	c	-16.99	281.0	-0.2812	0.9100	1000.00	2560.81	0.00192	V3	scncl	c	-172.33	13.1	-0.00	0.01	0.00458
V3	scncl	h	-141.26	17.7	-0.0572	0.0295	304.31	189.70	0.00012	V3	scncl	h	-186.51	5.6	-0.01	0.00	0.00083
V3	scncl	n	83.82	11.4	-0.0222	0.0064	1.00	3.71	0.00191	V3	scncl	n	77.48	10.3	-0.02	0.00	0.00283
V3	scnso4	c	-140.81	289.8	-0.0326	0.9386	1000.00	22801.93	0.00201	V3	scnso4	c	-167.63	7.1	0.01	0.00	0.00138
V3	scnso4	h	-345.28	74.5	0.2283	0.2411	999.39	835.49	0.00013	V3	scnso4	h	-224.30	7.1	0.00	0.00	0.00139
V3	scnso4	n	-639.23	378.3	1.1534	1.2253	1000.00	840.78	0.00343	V3	scnso4	n	-16.56	37.7	0.01	0.02	0.03932
V3	so4	c	-81.65	12.5	0.0658	0.0093	9.87	7.86	0.00140	V3	so4	c	-51.52	18.7	0.04	0.01	0.00977
V45	cl	c	37.14	7.6	0.0633	0.0082	99.31	23.76	0.00011	V45	cl	c	90.32	11.9	0.02	0.01	0.00363
V45	cl	h	6.80	9.8	0.0603	0.0103	89.46	29.13	0.00020	V45	cl	h	56.95	11.9	0.02	0.01	0.00358
V45	cl	n	8.67	12.0	0.2463	0.0134	110.16	10.63	0.00024	V45	cl	n	213.81	42.4	0.06	0.02	0.04582
V45	clso4	c	36.21	13.5	0.0987	0.0118	45.49	12.91	0.00071	V45	clso4	c	115.88	21.0	0.03	0.01	0.01172
V45	clso4	h	40.98	5.1	0.0690	0.0038	19.76	3.35	0.00018	V45	clso4	h	89.03	17.0	0.03	0.01	0.00767
V45	clso4	n	146.35	23.9	0.2716	0.0177	19.19	3.87	0.00398	V45	clso4	n	333.66	67.0	0.13	0.03	0.11918
V45	scn	c	-54.70	4.1	0.0926	0.0036	31.56	3.89	0.00011	V45	scn	c	-0.96	21.3	0.05	0.01	0.01297
V45	scncl	c	-38.52	4.4	0.0943	0.0029	9.98	1.18	0.00018	V45	scncl	c	16.60	25.0	0.05	0.01	0.01667
V45	scncl	h	9.80	3.2	0.0826	0.0022	10.36	1.03	0.00010	V45	scncl	h	58.47	21.7	0.05	0.01	0.01256
V45	scncl	n	76.00	9.0	0.3592	0.0061	10.80	0.68	0.00074	V45	scncl	n	289.79	93.6	0.20	0.04	0.23347
V45	scn	h	-6.17	1.8	0.0832	0.0015	21.81	1.47	0.00003	V45	scn	h	37.92	20.2	0.05	0.01	0.01165
V45	scn	n	13.39	8.1	0.3660	0.0066	21.19	1.46	0.00052	V45	scn	n	205.69	89.0	0.21	0.05	0.22636
V45	scnso4	c	-57.17	6.2	0.0963	0.0044	16.67	2.45	0.00029	V45	scnso4	c	15.53	25.1	0.04	0.01	0.01743
V45	scnso4	h	10.62	3.6	0.0961	0.0026	16.64	1.44	0.00010	V45	scnso4	h	82.81	24.7	0.04	0.01	0.01685
V45	scnso4	n	89.21	11.5	0.4097	0.0082	15.49	1.00	0.00104	V45	scnso4	n	391.91	105.5	0.18	0.04	0.30752
V45	so4	c	-19.20	13.5	0.1321	0.0188	200.16	40.45	0.00014	V45	so4	c	82.62	16.2	0.02	0.01	0.00733
V45	so4	h	21.88	4.2	0.0429	0.0035	25.83	7.03	0.00010	V45	so4	h	46.96	10.1	0.02	0.01	0.00285
V45	so4	n	54.81	14.1	0.2252	0.0128	41.43	6.38	0.00086	V45	so4	n	201.70	47.8	0.10	0.02	0.06384
W35	cl	c	-196.39	168.7	0.0886	0.5463	1000.00	4872.78	0.00069	W35	cl	c	-148.21	6.2	0.00	0.00	0.00099
W35	cl	h	-13.61	7.6	0.0074	0.0068	49.56	106.95	0.00020	W35	cl	h	-7.46	3.4	0.00	0.00	0.00030
W35	cl	n	111.90	11.8	0.4076	0.0133	115.44	6.57	0.00022	W35	cl	n	452.81	69.4	0.09	0.03	0.12260
W35	clso4	c	-79.50	22.9	-0.0791	0.0194	39.51	24.19	0.00228	W35	clso4	c	-141.39	18.8	-0.03	0.01	0.00936
W35	clso4	h	1.23	2.0	0.0030	0.0011	1.15	5.07	0.00006	W35	clso4	h	2.16	1.7	0.00	0.00	0.00008
W35	clso4	n	196.20	16.4	0.5574	0.0116	15.01	1.04	0.00212	W35	clso4	n	560.57	141.2	0.28	0.06	0.52940
W35	scn	c	-101.55	4.2	-0.0741	0.0033	18.08	3.37	0.00015	W35	scn	c	-138.81	18.4	-0.04	0.01	0.00969
W35	scncl	c	-118.85	9.7	-0.0927	0.0079	32.05	7.29	0.00048	W35	scncl	c	-191.20	22.5	-0.04	0.01	0.01353
W35	scncl	h	-31.69	4.8	-0.0301	0.0032	9.03	3.72	0.00023	W35	scncl	h	-48.45	8.2	-0.02	0.00	0.00179
W35	scncl	n	215.09	22.8	0.5277	0.0159	13.49	1.41	0.00433	W35	scncl	n	547.97	135.0	0.28	0.06	0.48483
W35	scn	h	-30.75	1.9	-0.0321	0.0014	10.11	2.53	0.00004	W35	scn	h	-44.68	8.5	-0.02	0.00	0.00207
W35	scn	n	114.20	14.2	0.4735	0.0119	26.69	2.32	0.00141	W35	scn	n	377.91	111.6	0.25	0.06	0.35550
W35	scnso4	c	-120.70	15.3	-0.0839	0.0117	22.95	9.33	0.00147	W35	scnso4	c	-189.94	23.3	-0.03	0.01	0.01503
W35	scnso4	h	-15.69	3.8	-0.0388	0.0032	40.72	8.26	0.00006	W35	scnso4	h	-48.98	8.4	-0.01	0.00	0.00196
W35	scnso4	n	181.30	8.1	0.5544	0.0055	11.47	0.40	0.00060	W35	scnso4	n	564.73	146.6	0.27	0.06	0.59370
W35	so4	h	-9.66	3.3	0.0050	0.0022	1.00	16.14	0.00012	W35	so4	h	-8.09	2.6	0.00	0.00	0.00018
W35	so4	n	116.59	27.4	0.6087	0.0247	40.89	4.53	0.00326	W35	so4	n	513.09	129.7	0.27	0.07	0.46964
W71	cl	c	1.55	104.1	-0.0090	0.3368	1000.00	29578.40	0.00026	W71	cl	c	-5.45	3.0	0.00	0.00	0.00023
W71	cl	h	-6.67	9.2	0.0247	0.0098	94.41	70.04	0.00017	W71	cl	h	14.35	5.5	0.01	0.00	0.00078
W71	cl	n	437.44	7.6	0.0510	0.0070	56.15	17.48	0.00019	W71	cl	n	477.73	11.5	0.02	0.01	0.00337
W71	clso4	c	-30.09	4.4	-0.0126	0.0025	1.00	2.53	0.00029	W71	clso4	c	-33.90	4.9	-0.01	0.00	0.00063
W71	clso4	h	26.96	2.3	0.0223	0.0016	12.37	3.04	0.00004	W71	clso4	h	40.84	5.9	0.01	0.00	0.00092
W71	clso4	n	989.32	13.7	0.1262	0.0085	5.09	1.64	0.00219	W71	clso4	n	1049.36	35.1	0.08	0.02	0.03266
W71	scn	c	19.06	80.3	0.0071	0.2649	1000.00	29970.01	0.00024	W71	scn	c	25.58	2.5	-0.00	0.00	0.00018
W71	scncl	c	35.72	121.4	-0.0346	0.3932	1000.00	8986.10	0.00036	W71	scncl	c	15.83	3.9	0.00	0.00	0.00039

W71	scncl	h	-12.57	4.9	0.0187	0.0036	17.80	10.97	0.00018	W71	scncl	h	0.60	5.7	0.01	0.00	0.00086
W71	scncl	n	1020.38	16.6	0.1033	0.0118	15.46	5.90	0.00214	W71	scncl	n	1089.36	28.8	0.05	0.01	0.02200
W71	scn	h	-24.33	3.8	0.0288	0.0039	67.48	21.01	0.00005	W71	scn	h	-4.44	6.1	0.01	0.00	0.00106
W71	scn	n	540.49	13.0	0.1780	0.0136	72.70	12.46	0.00058	W71	scn	n	663.85	35.4	0.06	0.02	0.03587
W71	scnso4	c	27.24	8.0	-0.0098	0.0053	9.92	19.41	0.00063	W71	scnso4	c	20.72	5.4	-0.00	0.00	0.00081
W71	scnso4	h	-3.51	2.9	0.0267	0.0020	13.18	3.37	0.00007	W71	scnso4	h	15.57	7.2	0.01	0.00	0.00143
W71	scnso4	n	1174.77	10.7	0.1605	0.0071	10.65	1.68	0.00108	W71	scnso4	n	1284.52	43.6	0.08	0.02	0.05258
W71	so4	c	-42.32	5.8	-0.0242	0.0039	1.00	5.88	0.00039	W71	so4	c	-50.13	8.1	-0.02	0.00	0.00183
W71	so4	h	19.06	1.5	0.0213	0.0011	6.26	2.50	0.00002	W71	so4	h	27.81	5.9	0.01	0.00	0.00097
W71	so4	n	569.45	13.3	0.1952	0.0105	18.73	3.98	0.00123	W71	so4	n	674.41	47.7	0.11	0.02	0.06363
W94	cl	c	89.22	3.2	0.0228	0.0021	5.44	3.00	0.00011	W94	cl	c	99.21	6.9	0.02	0.00	0.00121
W94	cl	h	-14.52	7.3	0.0086	0.0064	44.29	80.20	0.00021	W94	cl	h	-7.50	3.7	0.00	0.00	0.00034
W94	cl	n	98.08	4.0	0.0128	0.0029	14.78	12.32	0.00012	W94	cl	n	105.35	3.9	0.01	0.00	0.00038
W94	clso4	c	112.16	25.2	0.0638	0.0314	153.18	118.42	0.00072	W94	clso4	c	165.92	9.5	0.01	0.00	0.00238
W94	clso4	h	-14.13	2.9	0.0150	0.0019	8.81	4.42	0.00008	W94	clso4	h	-5.69	4.3	0.01	0.00	0.00049
W94	clso4	n	256.68	4.0	0.0289	0.0027	9.54	3.36	0.00015	W94	clso4	n	273.25	7.8	0.02	0.00	0.00161
W94	scn	c	71.53	29.2	0.0529	0.0348	113.58	139.46	0.00185	W94	scn	c	111.76	12.9	0.01	0.01	0.00476
W94	scncl	c	139.11	24.4	0.0327	0.0202	35.64	56.94	0.00278	W94	scncl	c	166.74	13.4	0.01	0.01	0.00480
W94	scncl	h	-2.67	3.3	0.0078	0.0023	13.16	13.40	0.00009	W94	scncl	h	2.47	2.9	0.00	0.00	0.00022
W94	scncl	n	190.45	6.3	0.0137	0.0038	3.95	5.95	0.00049	W94	scncl	n	196.54	5.8	0.01	0.00	0.00089
W94	scn	h	-3.60	3.4	0.0200	0.0039	92.54	36.34	0.00003	W94	scn	h	10.76	3.9	0.01	0.00	0.00043
W94	scn	n	76.47	2.8	0.0243	0.0025	35.78	10.97	0.00005	W94	scn	n	91.16	5.8	0.01	0.00	0.00094
W94	scnso4	c	191.12	8.8	0.0453	0.0072	35.32	14.51	0.00036	W94	scnso4	c	230.34	11.3	0.01	0.00	0.00350
W94	scnso4	h	5.17	3.2	0.0164	0.0021	10.93	5.05	0.00010	W94	scnso4	h	16.36	4.7	0.01	0.00	0.00061
W94	scnso4	n	276.02	7.1	0.0291	0.0045	6.75	4.09	0.00057	W94	scnso4	n	293.33	8.9	0.02	0.00	0.00218
W94	so4	c	25.49	45.6	0.1899	0.1071	600.02	314.69	0.00019	W94	so4	c	144.24	10.3	0.01	0.01	0.00295
W94	so4	h	-3.63	2.2	0.0117	0.0015	1.00	4.72	0.00006	W94	so4	h	0.17	3.8	0.01	0.00	0.00040
W94	so4	n	189.74	11.4	0.0446	0.0094	23.26	17.25	0.00082	W94	so4	n	214.77	11.5	0.02	0.01	0.00371
Y103	cl	c	18.34	8.0	0.0248	0.0065	30.98	22.63	0.00032	Y103	cl	c	35.97	7.1	0.01	0.00	0.00130
Y103	cl	h	11.91	79.0	-0.0686	0.2556	999.45	2946.56	0.00015	Y103	cl	h	-23.38	3.3	-0.00	0.00	0.00027
Y103	cl	n	60.55	5.1	-0.0079	0.0036	13.38	23.64	0.00020	Y103	cl	n	55.88	3.6	-0.00	0.00	0.00034
Y103	clso4	c	11.73	4.6	0.0653	0.0031	11.01	1.93	0.00020	Y103	clso4	c	50.71	16.9	0.04	0.01	0.00754
Y103	clso4	h	25.81	11.2	-0.0367	0.0190	321.53	197.62	0.00004	Y103	clso4	h	-2.96	3.4	-0.00	0.00	0.00031
Y103	clso4	n	133.78	13.0	-0.0712	0.0079	4.17	2.42	0.00205	Y103	clso4	n	101.86	21.3	-0.05	0.01	0.01204
Y103	scn	c	-15.58	2.9	0.0165	0.0022	12.20	8.26	0.00008	Y103	scn	c	-8.10	4.6	0.01	0.00	0.00059
Y103	scncl	c	-21.43	7.0	0.0293	0.0043	4.84	3.52	0.00058	Y103	scncl	c	-7.79	9.2	0.02	0.00	0.00224
Y103	scncl	h	-20.76	6.3	-0.0282	0.0048	22.62	11.34	0.00025	Y103	scncl	h	-40.41	7.0	-0.01	0.00	0.00131
Y103	scncl	n	79.74	17.9	-0.0341	0.0101	1.00	3.80	0.00472	Y103	scncl	n	69.81	16.2	-0.03	0.01	0.00701
Y103	scn	h	-32.59	2.7	-0.0138	0.0020	11.13	8.79	0.00007	Y103	scn	h	-38.71	3.9	-0.01	0.00	0.00044
Y103	scn	n	-5.80	490.3	0.0680	1.6182	1000.00	19093.17	0.00902	Y103	scn	n	47.52	15.6	-0.01	0.01	0.00696
Y103	scnso4	c	-21.74	6.2	0.0610	0.0038	4.62	1.24	0.00050	Y103	scnso4	c	10.95	16.8	0.04	0.01	0.00784
Y103	scnso4	h	-29.71	2.1	-0.0037	0.0014	11.67	15.34	0.00004	Y103	scnso4	h	-32.18	1.5	-0.00	0.00	0.00006
Y103	scnso4	n	122.54	20.7	-0.0763	0.0116	2.09	1.79	0.00644	Y103	scnso4	n	90.57	25.6	-0.05	0.01	0.01818
Y103	so4	c	3.52	6.6	0.0756	0.0059	38.65	8.39	0.00020	Y103	so4	c	52.22	16.5	0.03	0.01	0.00760
Y103	so4	h	15.32	69.1	-0.0102	0.2224	1000.00	17162.44	0.00012	Y103	so4	h	8.40	2.0	0.00	0.00	0.00011
Y103	so4	n	39.65	12.8	-0.0403	0.0087	1.00	7.83	0.00190	Y103	so4	n	26.65	14.5	-0.03	0.01	0.00589
Y13	cl	c	21.49	73.7	-0.0372	0.2386	1000.00	5078.89	0.00013	Y13	cl	c	0.08	2.7	0.00	0.00	0.00018
Y13	cl	h	-6.87	6.8	0.0108	0.0064	57.64	76.13	0.00015	Y13	cl	h	1.82	3.4	0.00	0.00	0.00030
Y13	cl	n	-56.36	11.4	0.0390	0.0174	252.89	146.83	0.00007	Y13	cl	n	-25.06	4.7	0.00	0.00	0.00056
Y13	clso4	c	-8.92	3.2	-0.0047	0.0019	2.00	6.27	0.00014	Y13	clso4	c	-10.62	2.7	-0.00	0.00	0.00019
Y13	clso4	h	11.21	3.8	0.0130	0.0030	26.94	17.34	0.00008	Y13	clso4	h	20.89	3.5	0.01	0.00	0.00033
Y13	clso4	n	-73.65	10.1	-0.0072	0.0088	45.38	132.49	0.00040	Y13	clso4	n	-80.72	4.4	-0.00	0.00	0.00052
Y13	scn	c	-11.82	140.4	0.0631	0.4633	1000.00	5895.09	0.00074	Y13	scn	c	24.06	5.7	-0.00	0.00	0.00094
Y13	scncl	c	-27.92	104.5	0.0757	0.3384	999.57	3536.19	0.00027	Y13	scncl	c	12.40	4.1	0.00	0.00	0.00044
Y13	scncl	h	10.03	21.0	0.0231	0.0353	311.34	570.97	0.00016	Y13	scncl	h	29.14	3.5	0.00	0.00	0.00032
Y13	scncl	n	44.80	166.8	0.2407	0.5399	998.75	1773.05	0.00068	Y13	scncl	n	176.45	10.0	0.00	0.00	0.00265
Y13	scn	h	-2.73	10.3	0.0473	0.0184	329.65	154.14	0.00005	Y13	scn	h	30.75	5.0	0.01	0.00	0.00070

Y13	scn	n	31.76	111.6	0.2988	0.3680	998.04	987.13	0.00047	Y13	scn	n	182.01	12.3	0.01	0.01	0.00431
Y13	scnso4	c	-77.07	283.2	0.1674	0.9173	1000.00	4336.99	0.00192	Y13	scnso4	c	15.12	10.0	0.00	0.00	0.00274
Y13	scnso4	h	-1.26	17.5	0.0640	0.0249	215.67	115.87	0.00021	Y13	scnso4	h	52.91	7.0	0.01	0.00	0.00137
Y13	scnso4	n	79.48	160.1	0.2497	0.5181	998.63	1640.65	0.00062	Y13	scnso4	n	211.92	8.8	0.00	0.00	0.00212
Y13	so4	c	-15.01	5.2	-0.0099	0.0036	1.00	12.96	0.00032	Y13	so4	c	-18.18	4.5	-0.01	0.00	0.00055
Y13	so4	h	15.37	2.5	0.0139	0.0020	16.85	9.95	0.00005	Y13	so4	h	22.65	3.7	0.01	0.00	0.00037
Y13	so4	n	-89.74	10.7	0.0061	0.0120	102.08	370.68	0.00021	Y13	so4	n	-84.89	3.0	0.00	0.00	0.00025
Y17	cl	c	-23.56	4.5	0.0113	0.0027	1.57	5.47	0.00024	Y17	cl	c	-19.81	4.7	0.01	0.00	0.00055
Y17	cl	h	-22.75	6.1	0.0048	0.0050	33.20	95.24	0.00018	Y17	cl	h	-19.04	3.0	0.00	0.00	0.00022
Y17	cl	n	-3.16	9.1	0.0255	0.0107	130.43	91.01	0.00011	Y17	cl	n	18.01	4.5	0.01	0.00	0.00052
Y17	clso4	c	-20.92	10.4	-0.0348	0.0078	22.14	14.83	0.00069	Y17	clso4	c	-45.60	9.6	-0.02	0.00	0.00247
Y17	clso4	h	-18.40	1.7	0.0027	0.0009	1.00	4.48	0.00004	Y17	clso4	h	-17.65	1.4	0.00	0.00	0.00005
Y17	clso4	n	54.54	6.3	0.0099	0.0041	7.87	13.26	0.00041	Y17	clso4	n	60.03	4.8	0.01	0.00	0.00060
Y17	scn	c	-61.97	5.0	0.0060	0.0034	1.00	20.73	0.00032	Y17	scn	c	-60.12	3.8	0.00	0.00	0.00041
Y17	scncl	c	-49.57	25.7	-0.0477	0.0403	268.95	288.40	0.00032	Y17	scncl	c	-88.46	5.9	-0.01	0.00	0.00094
Y17	scncl	h	-19.92	84.8	-0.0370	0.2746	999.70	5871.46	0.00017	Y17	scncl	h	-40.95	2.9	0.00	0.00	0.00022
Y17	scncl	n	-45.07	18.5	0.0429	0.0240	170.02	143.62	0.00034	Y17	scncl	n	-6.49	7.4	0.01	0.00	0.00145
Y17	scn	h	-34.35	2.4	0.0083	0.0020	21.38	19.42	0.00005	Y17	scn	h	-29.93	2.4	0.00	0.00	0.00017
Y17	scn	n	-55.74	7.2	0.0588	0.0089	131.85	35.14	0.00009	Y17	scn	n	-13.36	9.1	0.02	0.00	0.00234
Y17	scnso4	c	-2.15	21.3	-0.0771	0.0291	194.07	105.46	0.00037	Y17	scnso4	c	-69.38	9.6	-0.01	0.00	0.00257
Y17	scnso4	h	-35.77	2.5	0.0079	0.0017	13.56	9.94	0.00005	Y17	scnso4	h	-30.00	2.5	0.00	0.00	0.00018
Y17	scnso4	n	32.54	4.6	0.0172	0.0035	21.09	12.70	0.00014	Y17	scnso4	n	46.36	5.0	0.01	0.00	0.00070
Y17	so4	c	-23.35	13.2	-0.0035	0.0150	109.44	861.65	0.00030	Y17	so4	c	-26.46	3.3	-0.00	0.00	0.00031
Y17	so4	h	-8.27	3.3	0.0039	0.0023	1.00	20.85	0.00013	Y17	so4	h	-7.04	2.4	0.00	0.00	0.00016
Y17	so4	n	20.89	6.4	0.0147	0.0048	12.35	20.04	0.00034	Y17	so4	n	27.93	5.1	0.01	0.00	0.00072
Y24	cl	c	24.87	21.6	0.0977	0.0226	90.99	40.06	0.00096	Y24	cl	c	104.16	18.2	0.03	0.01	0.00843
Y24	cl	h	-36.19	7.1	0.0084	0.0061	41.59	76.34	0.00021	Y24	cl	h	-29.50	3.6	0.00	0.00	0.00033
Y24	cl	n	-96.05	8.3	0.0081	0.0078	62.97	131.97	0.00020	Y24	cl	n	-89.27	3.4	0.00	0.00	0.00029
Y24	clso4	c	106.30	25.8	0.1033	0.0190	18.90	10.80	0.00465	Y24	clso4	c	178.58	29.4	0.05	0.01	0.02292
Y24	clso4	h	-25.22	3.3	0.0058	0.0019	2.52	5.76	0.00015	Y24	clso4	h	-23.01	2.8	0.00	0.00	0.00021
Y24	clso4	n	-67.34	151.1	-0.0815	0.3863	686.08	2911.63	0.00149	Y24	clso4	n	-124.57	8.7	0.00	0.00	0.00200
Y24	scn	c	11.83	14.6	0.1390	0.0157	80.78	19.59	0.00066	Y24	scn	c	108.15	26.0	0.05	0.01	0.01928
Y24	scncl	c	72.26	6.8	0.0986	0.0045	9.08	1.62	0.00045	Y24	scncl	c	127.73	25.9	0.06	0.01	0.01783
Y24	scncl	h	-38.89	138.7	-0.0701	0.4493	1000.00	5073.92	0.00047	Y24	scncl	h	-78.88	4.8	0.00	0.00	0.00062
Y24	scncl	n	-169.58	54.2	-0.1313	0.0927	324.45	270.54	0.00100	Y24	scncl	n	-268.96	11.3	-0.01	0.00	0.00338
Y24	scn	h	-65.10	3.1	0.0015	0.0021	2.13	57.30	0.00012	Y24	scn	h	-64.61	2.1	0.00	0.00	0.00013
Y24	scn	n	-191.42	35.2	-0.0519	0.0653	356.36	522.65	0.00049	Y24	scn	n	-224.78	5.0	-0.01	0.00	0.00072
Y24	scnso4	c	81.19	14.5	0.1324	0.0098	11.54	2.98	0.00192	Y24	scnso4	c	171.66	34.9	0.06	0.01	0.03374
Y24	scnso4	h	-74.40	3.5	0.0079	0.0021	4.69	5.37	0.00015	Y24	scnso4	h	-69.80	3.4	0.00	0.00	0.00032
Y24	scnso4	n	-211.65	153.5	-0.1707	0.4971	1000.00	2305.16	0.00056	Y24	scnso4	n	-305.08	7.1	-0.00	0.00	0.00140
Y24	so4	c	-13.83	27.4	0.1558	0.0376	190.78	66.43	0.00062	Y24	so4	c	107.68	20.7	0.03	0.01	0.01192
Y24	so4	h	-8.87	2.3	0.0103	0.0016	5.69	7.33	0.00005	Y24	so4	h	-4.69	3.2	0.01	0.00	0.00028
Y24	so4	n	-115.72	8.6	0.0177	0.0058	1.00	11.94	0.00086	Y24	so4	n	-110.08	7.6	0.01	0.00	0.00160
Y78	cl	c	3.60	12.3	0.0864	0.0129	89.95	25.57	0.00032	Y78	cl	c	74.97	16.5	0.02	0.01	0.00693
Y78	cl	h	-14.57	7.9	0.0039	0.0065	31.89	146.85	0.00031	Y78	cl	h	-11.47	3.7	0.00	0.00	0.00034
Y78	cl	n	-15.69	32.9	0.1046	0.0339	85.72	53.97	0.00238	Y78	cl	n	70.86	22.4	0.03	0.01	0.01273
Y78	clso4	c	28.15	13.2	0.1224	0.0101	24.23	5.81	0.00105	Y78	clso4	c	116.56	29.1	0.05	0.01	0.02246
Y78	clso4	h	-14.12	2.3	0.0084	0.0016	14.67	9.51	0.00004	Y78	clso4	h	-8.78	2.4	0.00	0.00	0.00015
Y78	clso4	n	-76.39	19.0	0.1877	0.0138	17.45	4.09	0.00264	Y78	clso4	n	49.98	46.9	0.09	0.02	0.05840
Y78	scn	c	-2.83	2.4	0.0976	0.0022	38.57	2.50	0.00003	Y78	scn	c	56.80	21.9	0.05	0.01	0.01368
Y78	scncl	c	27.89	4.0	0.1201	0.0026	8.12	0.72	0.00017	Y78	scncl	c	93.77	32.0	0.07	0.01	0.02720
Y78	scncl	h	-24.91	2.9	-0.0044	0.0017	1.00	4.85	0.00013	Y78	scncl	h	-26.17	2.5	-0.00	0.00	0.00016
Y78	scncl	n	-139.19	21.0	0.1972	0.0158	21.68	5.24	0.00284	Y78	scncl	n	0.62	49.0	0.09	0.02	0.06391
Y78	scn	h	-43.97	54.4	0.0468	0.1794	1000.00	3077.06	0.00011	Y78	scn	h	-19.06	2.8	0.00	0.00	0.00023
Y78	scn	n	-81.42	17.7	0.1976	0.0165	45.71	10.42	0.00157	Y78	scn	n	42.64	42.5	0.09	0.02	0.05159
Y78	scnso4	c	-7.06	15.8	0.1447	0.0116	18.68	4.63	0.00178	Y78	scnso4	c	104.07	37.4	0.06	0.02	0.03862
Y78	scnso4	h	-83.99	29.9	0.1105	0.0968	999.89	693.21	0.00002	Y78	scnso4	h	-26.56	2.9	0.00	0.00	0.00024

Y78	scnso4	n	-132.96	25.3	0.2165	0.0186	19.03	5.03	0.00449	Y78	scnso4	n	31.69	54.3	0.09	0.02	0.08147
Y78	so4	c	31.01	9.6	0.0621	0.0085	37.89	14.62	0.00042	Y78	so4	c	70.86	14.0	0.03	0.01	0.00547
Y78	so4	h	11.24	2.8	0.0208	0.0021	10.64	5.70	0.00007	Y78	so4	h	20.94	5.6	0.01	0.00	0.00089
Y78	so4	n	-14.76	18.7	0.1648	0.0155	25.30	8.06	0.00208	Y78	so4	n	80.73	38.9	0.09	0.02	0.04223
Y90	cl	c	-28.03	5.5	-0.0154	0.0038	10.21	10.88	0.00026	Y90	cl	c	-36.09	5.3	-0.01	0.00	0.00072
Y90	cl	h	-14.38	8.8	0.0153	0.0085	66.45	77.85	0.00022	Y90	cl	h	-1.66	4.5	0.00	0.00	0.00052
Y90	cl	n	-30.45	5.4	0.0227	0.0050	57.29	28.44	0.00009	Y90	cl	n	-12.56	5.2	0.01	0.00	0.00070
Y90	clso4	c	-19.04	152.9	-0.2213	0.4950	1000.00	1769.38	0.00056	Y90	clso4	c	-138.31	8.8	-0.00	0.00	0.00208
Y90	clso4	h	-6.37	2.2	0.0107	0.0014	5.98	3.48	0.00005	Y90	clso4	h	-1.04	3.2	0.01	0.00	0.00027
Y90	clso4	n	6.95	176.1	-0.0844	0.5126	847.25	4291.87	0.00118	Y90	clso4	n	-44.05	7.6	-0.00	0.00	0.00153
Y90	scn	c	-196.48	44.6	0.0400	0.0727	266.82	635.52	0.00131	Y90	scn	c	-165.68	8.6	0.00	0.00	0.00214
Y90	scncl	c	-221.51	42.3	0.0269	0.0768	365.97	1180.22	0.00048	Y90	scncl	c	-200.60	4.9	0.00	0.00	0.00063
Y90	scncl	h	-43.25	4.1	0.0043	0.0029	13.25	31.23	0.00014	Y90	scncl	h	-40.30	2.7	0.00	0.00	0.00019
Y90	scncl	n	-12.72	219.1	-0.0701	0.7095	1000.00	8003.87	0.00117	Y90	scncl	n	-44.84	5.9	-0.00	0.00	0.00092
Y90	scn	h	-37.38	3.4	0.0118	0.0033	52.87	38.17	0.00005	Y90	scn	h	-29.54	2.9	0.00	0.00	0.00025
Y90	scn	n	-37.05	3.8	-0.0062	0.0026	1.00	15.62	0.00019	Y90	scn	n	-38.88	3.1	-0.00	0.00	0.00028
Y90	scnso4	c	-219.79	19.0	-0.0161	0.0102	1.00	5.05	0.00601	Y90	scnso4	c	-224.18	15.0	-0.01	0.01	0.00623
Y90	scnso4	h	-41.84	3.1	0.0062	0.0019	5.17	6.67	0.00012	Y90	scnso4	h	-38.25	2.8	0.00	0.00	0.00021
Y90	scnso4	n	-20.77	11.7	-0.0435	0.0110	61.35	33.28	0.00042	Y90	scnso4	n	-57.96	8.2	-0.01	0.00	0.00186
Y90	so4	c	-14.74	8.7	-0.0087	0.0059	1.00	24.79	0.00088	Y90	so4	c	-17.49	6.2	-0.01	0.00	0.00106
Y90	so4	h	-6.95	2.2	0.0096	0.0015	1.00	5.76	0.00006	Y90	so4	h	-3.86	3.2	0.01	0.00	0.00028
Y90	so4	n	-88.62	6.1	0.0075	0.0042	1.00	20.03	0.00043	Y90	so4	n	-86.26	4.5	0.01	0.00	0.00057
Y97	cl	c	-43.04	5.8	0.0049	0.0046	27.12	74.46	0.00018	Y97	cl	c	-39.55	3.0	0.00	0.00	0.00023
Y97	cl	h	-23.28	8.2	0.0164	0.0075	53.76	56.02	0.00022	Y97	cl	h	-9.94	4.9	0.01	0.00	0.00062
Y97	cl	n	-108.50	7.6	0.0648	0.0074	70.17	16.74	0.00015	Y97	cl	n	-56.25	13.3	0.02	0.01	0.00450
Y97	clso4	c	-59.45	6.3	-0.0106	0.0041	9.36	14.10	0.00038	Y97	clso4	c	-65.76	4.8	-0.01	0.00	0.00062
Y97	clso4	h	-20.63	2.2	0.0091	0.0013	2.64	2.45	0.00006	Y97	clso4	h	-17.08	3.0	0.01	0.00	0.00023
Y97	clso4	n	-48.85	324.0	-0.2353	1.0491	1000.00	3527.38	0.00253	Y97	clso4	n	-183.67	12.7	0.00	0.01	0.00426
Y97	scn	c	-59.22	5.1	-0.0087	0.0035	1.00	14.85	0.00034	Y97	scn	c	-61.82	4.3	-0.01	0.00	0.00052
Y97	scncl	c	-71.54	40.5	-0.0231	0.0752	383.87	1383.35	0.00040	Y97	scncl	c	-89.43	4.4	-0.00	0.00	0.00050
Y97	scncl	h	-42.87	4.1	0.0128	0.0027	8.02	6.85	0.00017	Y97	scncl	h	-35.74	4.4	0.01	0.00	0.00051
Y97	scncl	n	-180.50	16.7	0.0344	0.0107	6.80	9.14	0.00305	Y97	scncl	n	-162.01	14.5	0.02	0.01	0.00562
Y97	scn	h	-42.49	2.4	0.0178	0.0020	24.19	9.64	0.00004	Y97	scn	h	-32.72	4.5	0.01	0.00	0.00058
Y97	scn	n	-171.33	8.5	0.0462	0.0064	11.37	8.38	0.00071	Y97	scn	n	-150.59	13.1	0.03	0.01	0.00490
Y97	scnso4	c	-58.53	20.6	-0.0247	0.0195	64.57	107.94	0.00123	Y97	scnso4	c	-79.42	7.7	-0.01	0.00	0.00162
Y97	scnso4	h	-40.02	2.4	0.0107	0.0014	3.14	2.00	0.00008	Y97	scnso4	h	-34.88	3.4	0.01	0.00	0.00032
Y97	scnso4	n	72.80	390.0	-0.4752	1.2633	1000.00	2103.96	0.00365	Y97	scnso4	n	-191.16	19.3	-0.00	0.01	0.01029
Y97	so4	c	-44.47	4.5	-0.0142	0.0031	1.00	7.83	0.00024	Y97	so4	c	-49.03	5.1	-0.01	0.00	0.00073
Y97	so4	h	-23.01	3.4	0.0113	0.0023	1.00	7.37	0.00013	Y97	so4	h	-19.39	4.0	0.01	0.00	0.00044
Y97	so4	n	-123.95	305.7	-0.2012	0.9839	1000.00	3841.44	0.00237	Y97	so4	n	-235.99	12.1	0.00	0.01	0.00405
